# Rehabilitation technology for self-care: Customised foot and ankle exercise software for people with diabetes

**DOI:** 10.1371/journal.pone.0218560

**Published:** 2019-06-20

**Authors:** Jane S. S. P. Ferreira, Isabel C. N. Sacco, Alisson A. Siqueira, Maria H. M. Almeida, Cristina D. Sartor

**Affiliations:** 1 Departamento Fisioterapia, Fonoaudiologia e Terapia Ocupacional, Faculdade de Medicina FMUSP, Universidade de São Paulo, São Paulo, São Paulo, Brazil; 2 Universidade Federal do Vale de São Francisco, Juazeiro, Pernanbuco, Brazil; 3 Universidade Ibirapuera, São Paulo, São Paulo, Brazil; Baylor College of Medicine, UNITED STATES

## Abstract

**Aims:**

To develop and validate the content of a free web-based software (desktop and mobile applications) for the self-management of and customised foot-ankle exercises for people with diabetes and diabetic neuropathy.

**Methods:**

The development of the programme was based on gamification principles and addressed three main areas: foot care recommendations; self-assessment of feet according to the main complications of diabetic neuropathy; and customised foot-ankle exercises to strengthen muscles, increase the range of motion and improve functionality. The content was validated using the Delphi methodology and a quantitative approach in two rounds with diabetes specialists (n = 9) and users with diabetes (n = 20). A 70% approval rate was considered sufficient in the second round for final validation purposes. The data analysis was conducted using descriptive statistics, absolute and relative frequencies and the content-validity index (CVI).

**Results:**

Among specialists, the CVI was 0.812 after the first round, and final approval was 100% after the second round. Among users, the CVI was 0.902 in the first round, and the final approval was 97%.

**Conclusion:**

This free access web software was developed based on the high agreement rating between specialists and users and has the potential to prevent complications arising from diabetic polyneuropathy. It allows for self-monitoring and promotes personalised exercises, following a preventive model that can be applied in primary and secondary care services as a complementary treatment for chronic complications. However, further steps to validate the software in a larger population are recommended.

## Introduction

Foot and ankle function are compromised when an individual suffers from diabetes mellitus (DM), and foot and ankle functioning can have costly outcomes if not prevented or treated[1,2]. According to the International Working Group on the Diabetic Foot[2], strategies have been suggested to encourage foot care and self-management, in addition to using therapeutic footwear when severe diabetic polyneuropathy (DPN) is present. However, although it is believed that muscle weakness and joint limitations in DM and DPN patients are irreversible, specific therapeutic foot and ankle exercises may contribute to preventing and controlling musculoskeletal and structural deficits that may affect foot function and balance and increase the risk for ulcers if not treated[3–5]. Up to now, there have been 10 clinical trials–some of them with low risk of bias–that have demonstrated the beneficial effects of foot and ankle–related exercises for improving DPN symptoms and sensory deficits[6,7] and for reducing peak plantar pressure[6–11], in addition, these studies have showed the ability for patients to improve foot–ankle range of motion[6,8,9,12–14] and foot–ankle muscle strength and functioning[12,14,15].

Supervised and unsupervised therapeutic foot-related exercises performed by patients with low and moderate neuropathy have been shown to reduce plantar pressure distribution during gait[6,8–11]. Likewise, in one study, a personalised therapeutic exercise protocol was followed for 12 weeks to rehabilitate small joints and foot–ankle muscles[7,9,16]. The results showed satisfactory changes in gait biomechanics with an improved distribution of plantar pressure, resulting in a better physiological pattern in foot–ankle rollover[7].

Besides exercises, educational and self-care actions are essential for preventing late consequences and help patients identify clinical situations earlier before a late diagnosis with complications, such as DPN [17]^,^[18]. The use of technology by health providers has not only improved patient monitoring and adherence, but has also reduced the demands on healthcare facilities[19]. A number of studies with DM patients have been conducted using e-health technologies that allow people to engage in activities in their preferred environment, thereby taking up less of the health professional’s time and decreasing demands on health centres[20]; they provide a means for people to better monitor themselves, having them depend less on face-to-face care and reducing human and financial costs.

So far, e-health technologies used for diabetes have focused on general, whole-body exercises or have had other purposes, such as glucose monitoring. Software for specific foot and ankle exercises is not available, and mostly, these programmes have not been able to personalise the exercises progression following the user’s individual physical capacities. In this context, the current study aimed to develop and validate free web software that can be accessed through computers or smartphones, here targeting people with DM and low or moderate DPN [21]; this had the potential of enabling self-management and customised care through a personalised foot–ankle exercise routine.

## Methods

### Software development and structure

We used an application layer that provides online services accessed through the World Wide Web or through mobile applications (Android and iOS). The system was developed using hypertext markup language and JavaScript for the interface and for the usability of the hypertext preprocessor tool employed to analyse user data. Structured query language (SQL) was used for the database and MySQL version 5.0.51, as well as SQLITE for systems management because it requires few hardware resources.

For the user requirements, an HTML 5/CSS 3 compatible browser is required and must be able to navigate in a web environment with a minimum resolution of 1200 x 780 pixels. To use this web software version, no installation is required: the user only has to enter the link www.usp.br/labimph/soped. To run the app, the user needs to have Android 4.3 or higher and to download the application from the same link, in the ‘download our application’. No operating system requirement is made. In both cases, users need Internet access. The software is available in English and Portuguese, but it can be translated into any language. The software was created with the intention of being used independently by a person with DM at his or her own convenience, but also has the potential to be a tool that facilitates primary and secondary health services worldwide.

The software is in its first version. We intend to revise it when including other languages while keeping the software free and public. All access codes and algorithms used in the software are available as supplemental material (S1 Appendix, S2 Appendix).

**Fig 1:**
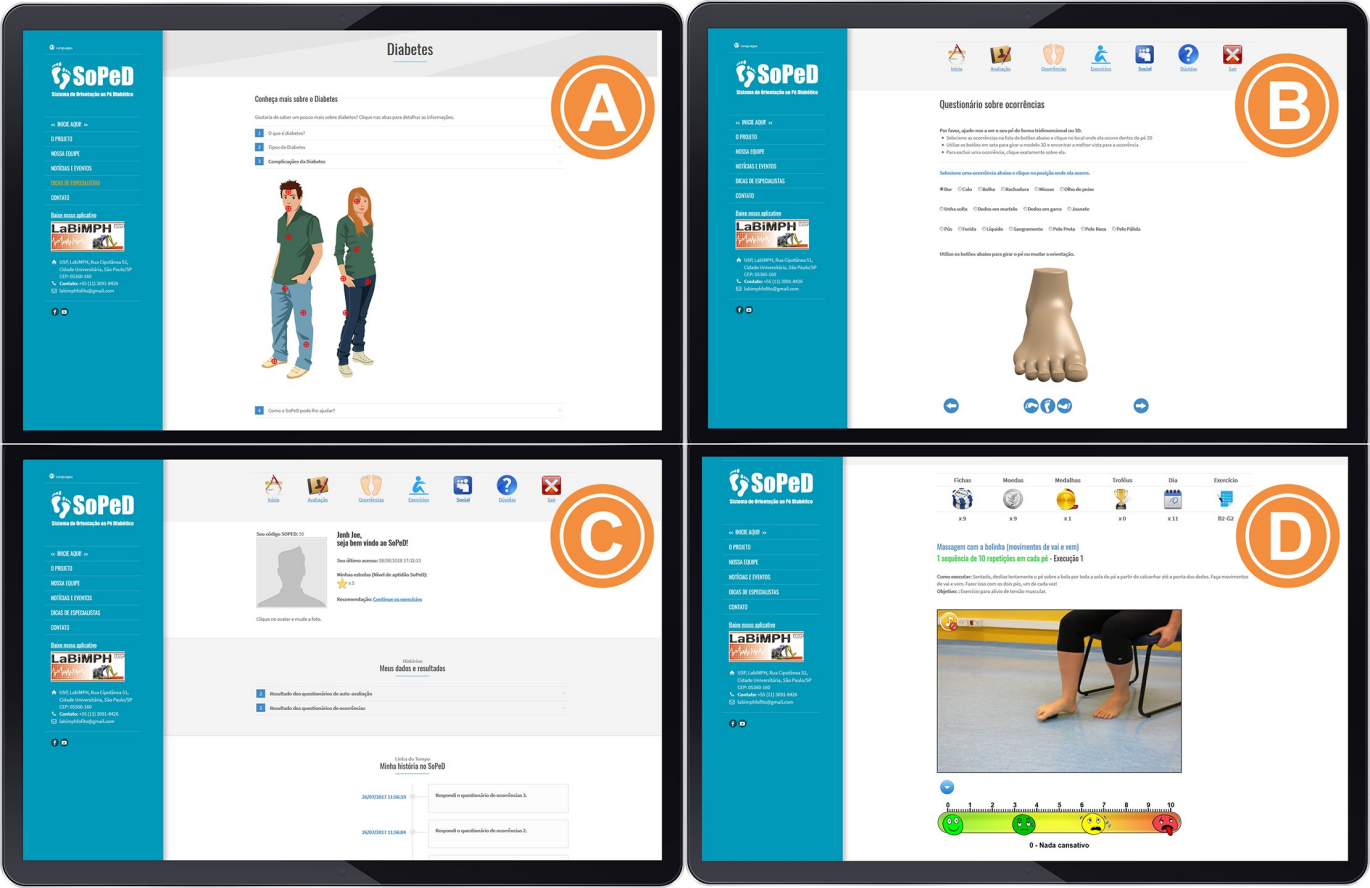
Layout of the three main aspects of the software: (a) Information about DM and DPN, (b) self-assessment of common foot problems with DM and DPN, (c) user profile and (d) exercises and methods of performance with the perceived effort scale.

**Fig 2:**
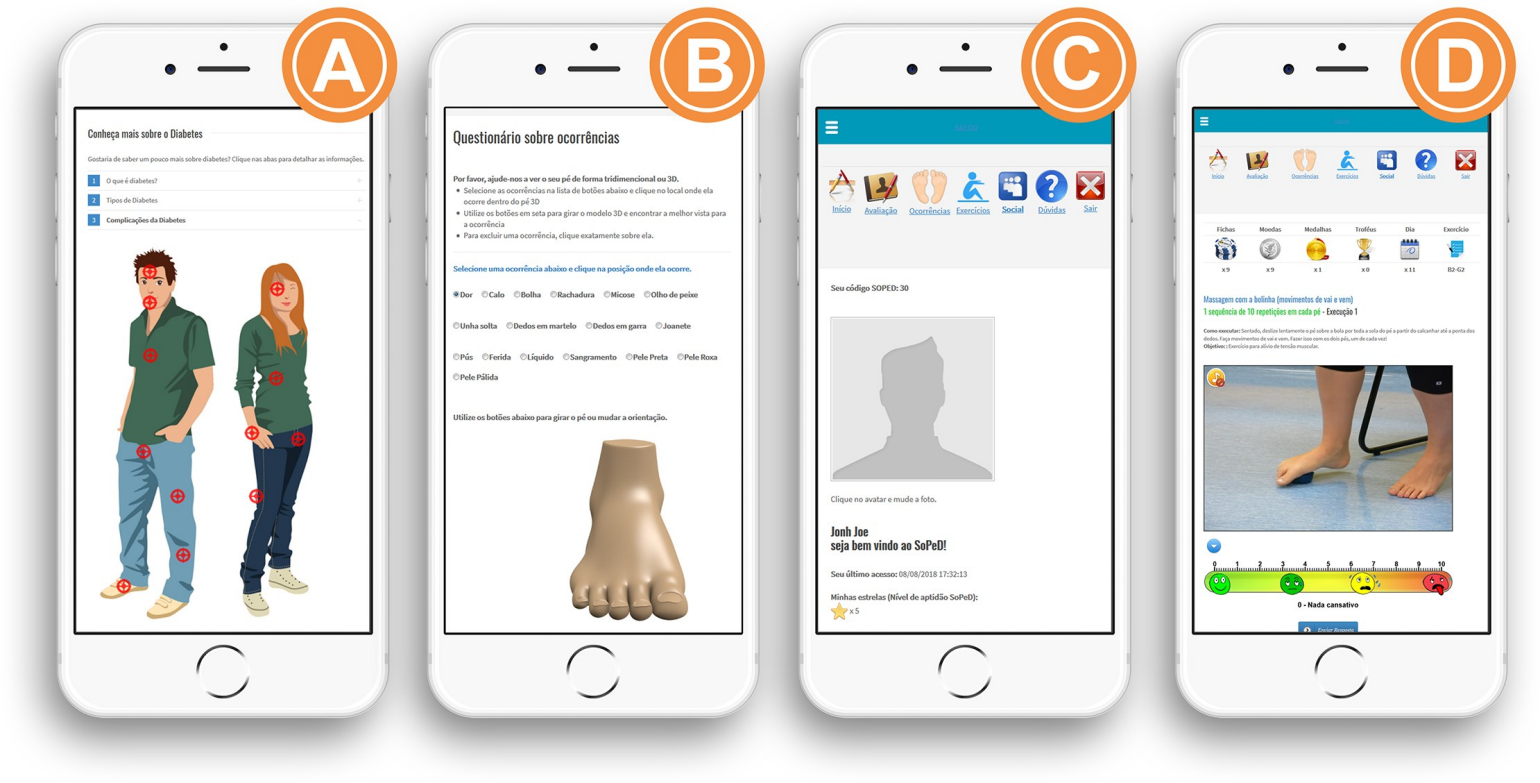
Mobile version of the same layout presented in the desktop: (a) Information about DM and DPN, (b) self-assessment of common foot problems with DM and DPN, (c) user profile and (d) exercises and methods of performance with the perceived effort scale.

In the development of this software (**Figs 1 and 2**), three main aspects were considered: (i) foot care recommendations and information about DM and DPN; (ii) self-assessment of feet according to the main foot alterations of DM and DPN (calluses, cracks, deformities and soft-tissue lesions, among others); and (iii) customised foot–ankle exercises to strengthen muscles, increase range of motion and improve functionality.

### Website sections and features

**Fig 3:**
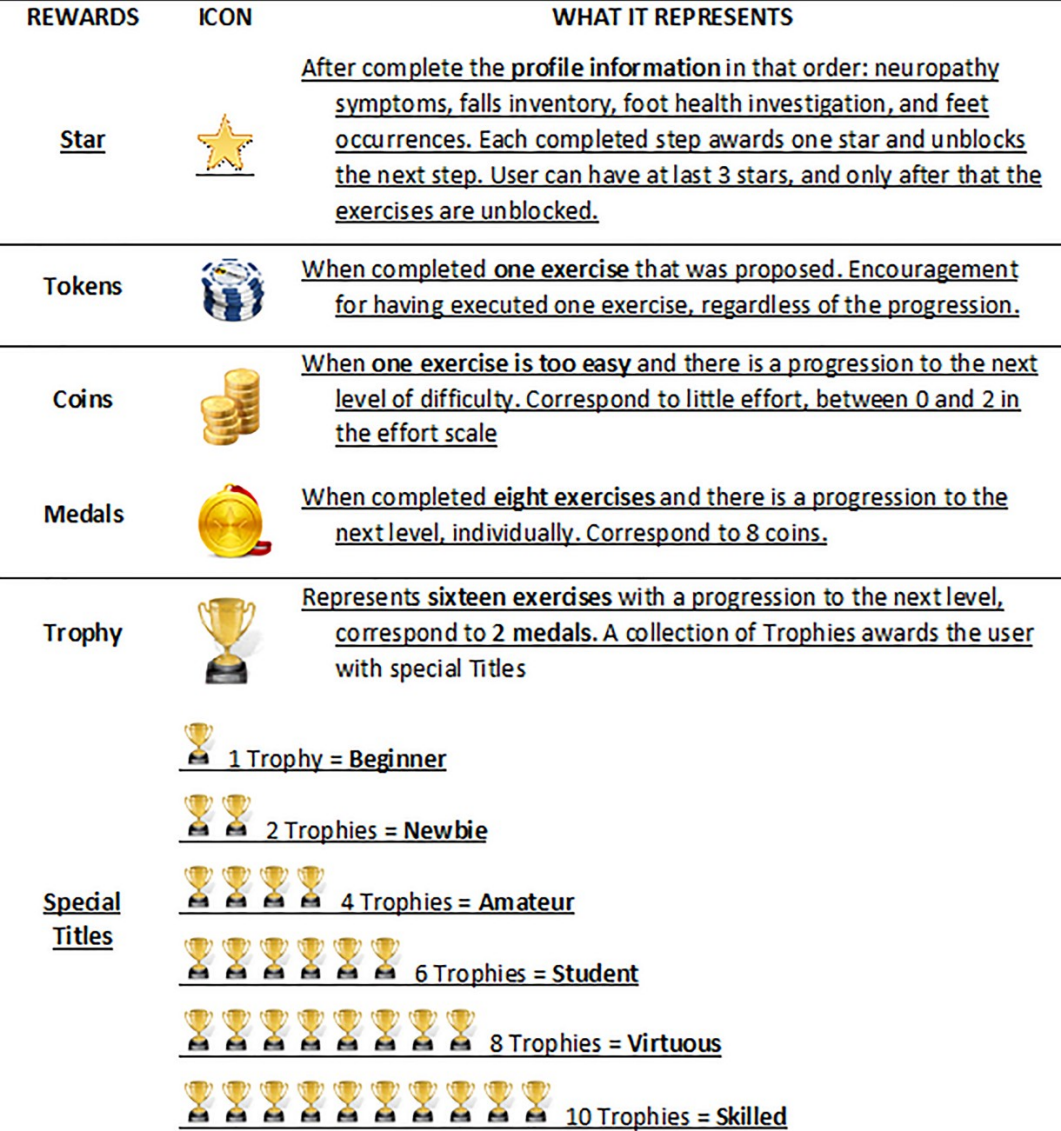
Details on the reward system and what each icon represents.

The main sections and features of the website include the following:

- Informative webpages about foot care recommendations and the disease’s main complications (Fig 1A).
- Self-assessment of the feet, which is included to encourage users to assess their feet regularly and stimulate an investigation of their health conditions. Some validated instruments were included: Michigan Neuropathy Screening Instrument[22,23] for the self-assessment of DPN signs and symptoms; Foot Health Status Questionnaire[24]; and a brief investigation of fall occurrences. To guarantee the users’ physical integrity, a physical examination of the user’s feet was included to check for the presence of foot alterations/deformations, such as calluses, cracks, mycoses, deformed toes, ulcers and amputations (Fig 1B). A general feedback of the user’s health status is provided, based on the answers given. If there are any signs or symptoms of severe health conditions, a clear recommendation is made to seek for medical assistance, such as severe polyneuropathy, increased risk for falls and bad foot health status. Users with preulcerative lesions are referred to contact a foot care specialist.
- A custom exercises section was made available only after the user had responded to all self-assessments in the software. The programme will personalise the exercises progression, according to each individual physical capacity. An effort scale that the user fulfil will determine the progression or not to other levels of difficulties.
- Because of its potential to increase engagement, gamification[25] components were employed throughout the user environment to encourage and motivate the patients to use the tool[26,27]. The user’s panel was designed with dynamic features and with game functions to stimulate the users to practice the exercises and navigate throughout the software. Information about diabetes and a physical examination of the feet come with a 2D animation and interactive menu. We inserted a reward system for the completion of each step of the software and after the sessions and progression of the exercises. Even if real progression in exercise difficulty had not occurred, users would be rewarded for their dedication and persistence, not just for their physical ability. Details of the reward system is presented in Fig 3.
- Possibility of interactions between users and researchers. A forum section was included to facilitate the exchange of information.

### Exercise protocol

A therapeutic exercise protocol was developed (S3 Appendix) to provide autonomy for the individuals during exercise without the need for professional supervision. The protocol is simple, contains clear written instructions (as well as video and audio) and preserves the safety of the target population during exercise. Furthermore, it establishes the training volume, progression criteria and guidelines for discontinuing the protocol.

A singular feature of this tool is that it personalises the progress of a foot–ankle exercise programme based on individual capabilities, which is similar to conventional physiotherapy. To include this feature, we incorporated a visual analogue scale (Fig 1D), which is represented by a ruler that quantifies the level of effort required to perform each exercise so that daily progress can be customised based on one’s results.

To personalise the exercises, a progression algorithm was created from the perceived effort of each user, who could progress in the exercise programme’s difficulty, maintain it or return to the previous stage in accordance with the following criteria: with a 0.0 to 2.0 score in the visual scale, the user progresses to the next level of effort on the following day; from 2.1 to 7.0, the user advances to the next level after 2 days at the present level; from 7.1 to 10, the user returns to the previous level.

The physiotherapeutic foot–ankle exercise protocol is based on previous clinical trials [7,16]. It was designed following three criteria established in a supervised, face-to-face intervention: (a) muscle stretching (20 exercises); (b) strengthening of the intrinsic foot muscles (40 exercises); and (c) strengthening of the extrinsic foot–ankle muscles and functional exercises such as balance and gait training (44 exercises). In total, 39 different exercises were chosen, and when including their sublevels of progression, a total of 104 different exercises can be completed. For each session, only eight exercises are combined to provide the three previously described criteria. The exercises are recommended to be performed twice or three times weekly. To avoid monotony and enhance motivation, the exercises always change from session to session, and the maximum duration of a session is no longer than 20 minutes. Those features also prevent the users from doing excessive effort, because they also limit the uncontrolled progression, as explained: the exercises should only be done twice or three times a week; no more than eight exercises each day is allowed; and the individual difficulty is regulated by the effort scale.

Some exercises have sublevels that correspond to increases in the load, number of repetitions or time duration. However, each exercise is different and may contain only one level or up to five levels of difficulty. For each exercise, the user attributes the effort, and the progression is made for this exercise. So if a session is composed of eight exercises, the user may progress in two of these exercises but may stay at the same level for six of the exercises in the next session. Therefore, individual physical capacities are respected, and one exercise will not block the progression of other exercises that are easier to perform. Therefore, the user’s overall progression is not classified as levels but rather follows the reward system of trophies and items presented in Fig 3.

The following muscle groups are targeted in the protocol: medial-plantar aspect: abductor hallucis, flexor halluces brevis and adductor hallucis; lateral plantar aspect: abductor digiti minimi, flexor digiti minimi brevis and opponens digiti minimi; middle-plantar aspect: flexor digitorum brevis, quadratus plantae, lumbrical muscles, plantar interosseous and dorsal interosseous muscles; dorsal-foot aspect: extensor digitorum brevis and extensor halluces brevis. The following joints are targeted in the protocol: talocrural, tarsometatarsals, interphalangeals and metatarsophalangeals.

### Tool validation

**Fig 4:**
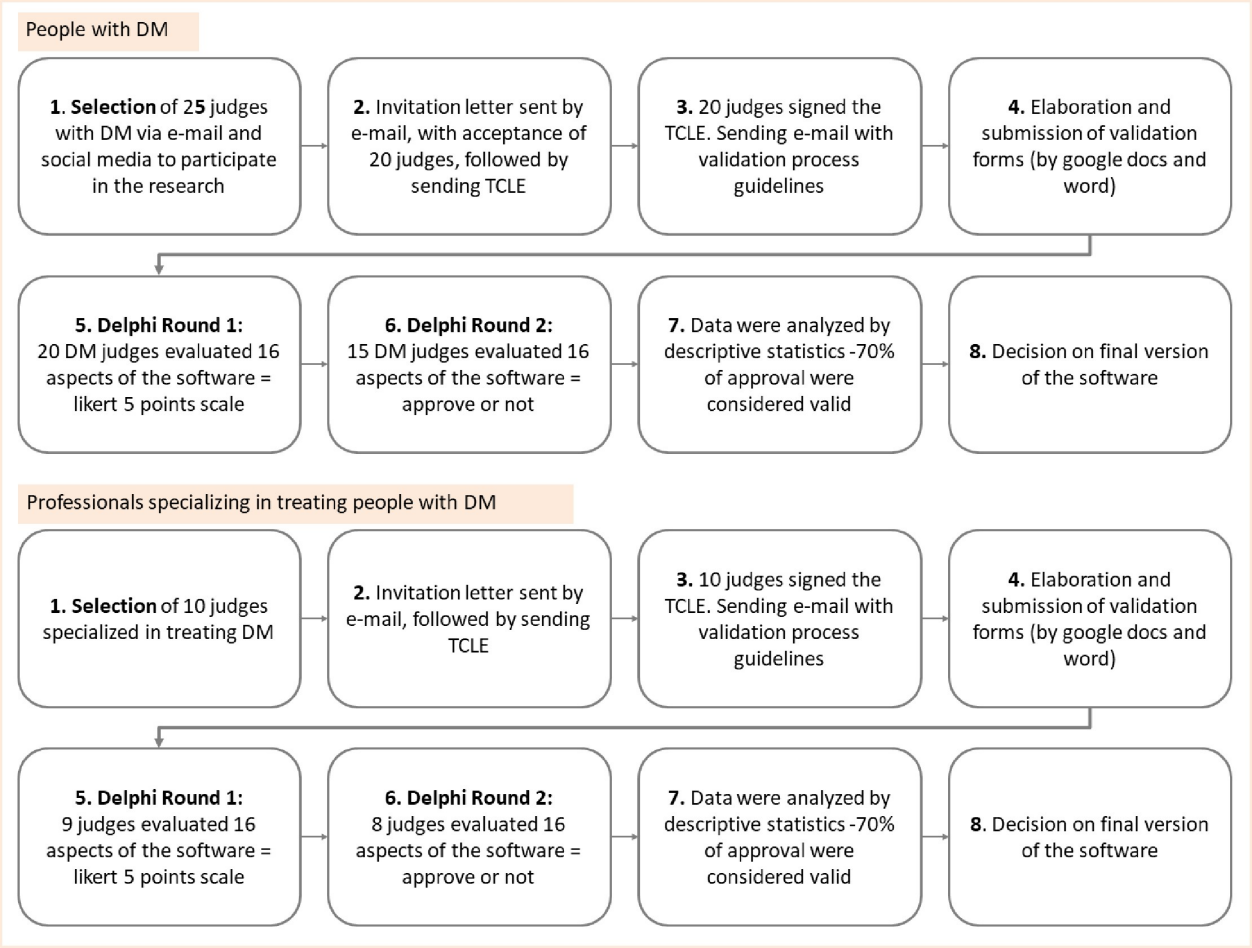
Flowchart of the software content showing the panel of health professional specialists and one person with diabetes who used the tool.

The Delphi method was used for validation[28]. The process occurred concurrently with a judging panel of 20 people with DM (Fig 4) and another panel of nine health professionals (Fig 4) specialising in treating people with DM and DPN. The judges had access to the desktop and mobile versions of the software, and their responses were given for both applications. The judges used the software for a period between 30 and 45 days, twice or three times a week. This time period was chosen because at least a usage of 30 days represent one third of the entire protocol, and is representative enough of the functionality of the software. During this time, judges could properly verify all sessions and features of the SOPED that are: fulfill personal information; read all the instructions and information about the disease; fulfill the feet physical inspection; read and practice many exercises; use the effort scale in different situations and with different exercises; verify the progression, maintenance or regression of the exercises according to their individual capacities; use the forum; receive the rewards; verify the safety information; and navigate through different sessions of the SOPED. In addition, all the judges received an attached file with detailed descriptions of all the exercises included in the software, whit their respective training volume and progression, and therefore could analyze all the protocol without the need to perform every exercises for three months.

The panel of users comprised six men (30%) and 14 women (70%) with a mean age of 41.4 years (21–65 years), mean DM diagnosis of 14 years (1–33 years) and normal cognitive performance as assessed by the Mini Mental State Examination (score media of 28.5). Depression level was assessed using Beck’s Depression Inventory, in which one of 20 subjects exhibited a score of 27 (moderate level), six a score of more than 4 (minimum level) and four a score of more than 12 (mild level). Educational level was 20% (4/20) for high school and 80% (16/20) for university level. In addition, 80% (16/20) of the patients were working. There were 12 patients with Diabetes but without neuropathy, confirmed by the Michigan Neuropathy Screening Instrument (MNSI) and 8 patients with previous confirmed diagnostic of neuropathy (MNSI and physical examination from the database of the research center). Other eligibility criteria for users were the following: both sexes: having type 1 or type 2 DM; 18 years old or more: free of tissue injury at the time of execution of the exercises; be able to use the software alone; or have someone to help at all times; and having completed schooling equal to or higher than the fourth year of elementary school.

The panel of specialists comprised nine women, including one psychologist, three physiotherapists (two specialised in clinical diabetes care, one specialised in biomechanics and musculoskeletal function of diabetic neuropathy), one podiatrist nurse (specialised in podiatric care and diabetic foot), one physician/endocrinologist (specialised in diabetic foot), one occupational therapist and two physical education professionals (one specialised in clinical and gait analysis of diabetic foot). The mean age was 45.44 years (35–59 years), and the mean experience in the treatment of people with DM was 18.8 years. For specialist selection, résumés were assessed using an adaptation of the Fehring criteria[29] (Table 1), which allows for a minimum score of 5 points. The mean adapted Fehring score was 9.7 out of 14. The other eligibility criteria for the specialists are described in Table 1 and are based on adapted Fehring criteria.

**Table 1:** Adaptation of the specialist scoring system according to the adapted Fehring criteria.

Fehring criteria (1994)	Score	Adapted criteria	Adapted Score
Master’s degree in nursing	4	Masters, courses or experience in continuous education related to diabetes	2
Master’s degree in nursing: dissertation with supplementary material content relevant in the area	1	Dissertation with relevant content in the area	1
Research (published articles in the area of diagnostics)	2	Studies published on diabetes and/or its complications or relevant content	2
Article published in the area of diagnostics in a reference journal	2	Article published on diabetes and/or its complications in an indexed journal	1
Doctorate in diagnostics	2	Doctorate related to the issue or medical area	2
Clinical practice of at least 1 year in the field of clinical nursing	1	Experience of at least 1 year in caring for patients with diabetes and/or with a focus on prevention or foot care	4
Certified in clinical medicine with proven clinical experience	2	Experience (clinical, teaching or research) with a focus on rehabilitation, exclusive or not, of diabetes	2
**Maximum Score**	**14**	**Maximum Score**	**14**

The first round of assessment consisted of a questionnaire containing 16 items that was based on a 5-point primar scale (I completely agree, I agree, I neither agree nor disagree, I disagree and I totally disagree) in which the comments considered important to each member of the panel were obtained for each item. The instrument that was built to discern the specialists’ opinions addressed the following matters: objective of the web software; fitness of the language to the population; amount and quality of information; contribution of exercises to decreasing foot deficits caused by DM; and whether the tool promoted daily exercise. The instrument that was built to elicit the user’s opinion addressed the same matters, but also aimed to determine whether the user correctly understood the exercise performance.

For the first round, both suggestions from specialists and users were evaluated only by the researchers, and their suggestions and recommended changes were incorporated into the software by the researchers. After incorporating the suggestions, the new version of the software was submitted again, in a second round, to the same panel members who evaluated the modifications in the software. At this stage, the changes should be approved or not, until a minimum of 70% was reached, and then the final version of the web software was determined.

### Statistical analysis

The data were analysed using descriptive statistics, means, relative and absolute frequencies and the content validation index (CVI). The CVI measures the proportion of items that the judges are in agreement. The content validity is determined by the proportion of judges that score items as being relevant or representative[30]. That correspond to a score of 4 or 5 (‘agree’ and ‘strongly agree’) on a Likert scale out of all possible answers (the others being ‘disagree’, ‘strongly disagree’, and ‘don’t agree nor disagree’). The score is calculated by the sum of the agreement of the items marked 4 or 5[31]. The CVI was calculated only for the first round. For the final validation, after the second round, we used a 70% approval consensus criterion for all modifications implemented in the software.

### Ethical approval

This research was approved by the Research Ethics Committee of the School of Medicine of the University of São Paulo (Approval No. 2.262.357). Informed consent was obtained from all participants included in the design process.

## Results

In the first round of the Delphi technique, we obtained an accurate and satisfactory result regarding all aspects of the web software that were queried, all of which showed a high degree of agreement.

Table 2 shows that 90.3% of the specialists agreed with the web software particulars, 5.6% neither agreed nor disagreed, and only 4.2% disagreed with some aspects. The results indicate that the initial version achieved satisfactory CVIs, given that all the CVIs of the individual items (n = 16) obtained values of more than 0.78, except for item 13, the question on accessibility (which was influenced by the presence of retinopathy in users, a common complication of persons with DM). We included an explanatory audio during the exercise-video demonstration to facilitate accessibility. The overall CVI of the first round was 0.902[32].

**Table 2:** Overall results (in percentage of the number of judges) of the Likert scale applied to the two panels (P–health professionals and U–software users with DM) in the first assessment round.

	QUESTIONS	*CA (%)	*A (%)	*NAND (%)	*D (%)	*CD (%)
1	**P**–Way of individualising exercises	55.6	44.4	0.0	0.0	0.0
	**U**–Informative content	65.0	30.0	5.0	0.0	0.0
2	**P**–It is easy, clear and intuitive to navigate using the software and/or application	66.7	22.2	0.0	11.1	0.0
	**U**–Promotes awareness and better foot care	65.0	35.0	0.0	0.0	0.0
3	**P**–The language is adequate for the general population	55.6	44.4	0.0	0.0	0.0
	**U**–Exercises can be performed in any environment	65.0	25.0	0.0	10.0	0.0
4	**P**–Suitability of the layout	66.7	22.2	0.0	11.1	0.0
	**U**–It is easy, clear and intuitive to navigate using the software	40.0	50.0	5.0	5.0	0.0
5	**P**–Motivation to exercise daily	33.3	66.7	0	0.0	0.0
	**U**–Suitability of the layout	70.0	25.0	5.0	0.0	0.0
6	**P**–Awareness about the importance of exercise	33.3	66.7	0	0.0	0.0
	**U**–Motivation to exercise daily	55.0	20.0	20.0	5.0	0.0
7	**P**–Questionnaires are adequate and important for foot problems	55.6	44.4	0.0	0.0	0.0
	**U**–Information on foot problems	60.0	35.0	5.0	0.0	0.0
8	**P**–Adequate self-assessment and self-examination	55.6	33.3	11.1	0.0	0.0
	**U**–The section on physical self-examination of the feet is clear	65.0	25.0	5.0	5.0	0.0
9	**P**–Appropriate exercises	55.6	22.2	22.2	0.0	0.0
	**U**–The material available is sufficient to perform the exercises alone	70.0	20.0	5.0	5.0	0.0
10	**P**–The software is self-informative, clear and self-sufficient	55.6	44.4	0.0	0.0	0.0
	**U**–Provides support in the case of doubt	60.0	25.0	15.0	0.0	0.0
11	**P**–Feedback after the exercises is provided and is essential for continued assessment and progress	100.0	0.0	0.0	0.0	0.0
	**U**–Feedback is motivational for questionnaire periodicity	70.0	20.0	5.0	5.0	0.0
12	**P**–Personalised training available	66.7	33.3	0.0	0.0	0.0
	**U**–Audio recordings help in exercise execution	50.0	20.0	15.0	5.0	10.0
13	**P**–The software contains questions on accessibility	33.3	0.0	44.4	0.0	22.2
	**U**–Weekly reminders are useful in maintaining frequency in the software	65.0	15.0	10.0	0.0	10.0
14	**P**–Serves as interdisciplinary support and can be included in primary and secondary health care	88.9	11.1	0.0	0.0	0.0
	**U**–Confidentiality of personal data is clearly explained	80.0	20.0	0.0	0.0	0.0
15	**P**–Confidentiality of personal data is clearly explained	77.8	0.0	11.1	0.0	11.1
	**U**–Safety emphasised during exercises	85.0	15.0	0.0	0.0	0.0
16	**P**–Safety emphasised during exercises	55.6	33.3	0.0	11.1	0.0
	**U**–The objective and importance of correctly completing the exertion scale is clearly explained	65.0	25.0	0.0	5.0	5.0
Total	**PROFESSIONALS**	59.7	30.6	5.6	2.1	2.1
	**USERS WITH DIABETES**	64.4	25.3	5.9	2.8	1.6

*CA = I completely agree; A = I agree; NAND = I neither agree nor disagree; D = I disagree; CD = I completely disagree

A similar result in the first round was observed in the assessment conducted by patients with DM (Table 2), in which 89.7% agreed, 5.9% neither agreed nor disagreed, and only 4.4% disagreed with some aspects. The CVI results of the individual items showed values higher than 0.78 for 14 of the 16 items in the instrument, and the overall CVI was 0.903[32].

Although there was a high degree of agreement in the first round, the panels suggested a number of changes that were incorporated, producing the first version of SOPED–Educational Diabetic Foot Software (Table 3).

**Table 3:** Final approval of the changes made to the software based on suggestions.

**SUGGESTIONS PRESENTED TO THE PANEL OF DIABETES SPECIALISTS**	**SUGGESTIONS APPROVED/JUSTIFIED**	**APPROVAL**
Review the numerical sequence of each exercise because it is not sequential.	The numerical sequence was corrected.	100%
Include alternative exercises to benefit users with reduced mobility.	The exercises included in the protocol were initially designed for most people with DM. Currently, the protocol cannot be changed, but this will be considered in future revisions.	100%
Include a note instructing users to ask for help in using the tool and improve the explanation on how to use the exertion scale.	Additional information explaining how to use the exertion scale was included.	100%
It was pointed out that the use of technology is not equal for everyone, especially in low-income countries, including Brazil.	The American Diabetes Association (ADA, 2016) has recommended that technologies that help in treating chronic diseases be developed because interventions using devices such as smartphones and computers may be more effective than conventional interventions. Moreover, access to these technologies has been growing.	100%
Difficulty navigating the software and accessing for the first time/registering.	Clearer information was provided on how to use the software for the first time.	100%
Review skin colours in the complications field and include a description of each complication.	Skin colours were changed from ‘dark, white and red’ to ‘black, purple and pale’.	100%
Some exercises, notably with the fingers, may be more difficult to perform without prior training. This condition could contribute to not repeating some exercises (limited joint mobility (LJM)–associated with neuropathy and obesity, which contributes to this difficulty).	Sartor et al. (2014) applied these exercises in individuals with severe neuropathies and limited mobility, and even those who experienced some difficulty showed significant improvements after a training period. At any rate, after the software is concluded, its effectiveness and applications will be tested in future studies.	100%
Include information that deals specifically with awareness of the importance of exercise, emphasising limited joint mobility, which is common in people with DM, and underscoring the importance of exercises for preserving foot health.	The information suggested was inserted into the first page of the software.	100%
Include a note underscoring that the assessment and exercises suggested in the software do not replace assessments by a health professional.	This information is found in some areas throughout the tool and can be viewed immediately after the user’s first assessment.	100%
Review the training volume described in the exercises, forms of execution and absence of explanatory audio.	Problems playing the audio and with exercise descriptions were corrected.	100%
Review the criterion for classifying subjects as ‘fallers’.	The criterion was revised. We consider recurrent fallers those who fell two or more times in a 6-month period.	100%
Review the criteria and correct functionality in blocking exercise access (which should be unblocked after completing assessments).	Tests were redone, and the problem was solved.	100%
Review the scale of difficulty because easy and difficult are in different categories of slightly, very and moderately tired.	The scale was revised, and the suggestion was included.	100%
Include information on safety while performing exercises, preventing hypoglycaemia, food tips before exercise and insulin application for those who use it.	The suggestions were included throughout the software, especially in the tutorial before the exercise protocol.	100%
**SUGGESTIONS FROM PANEL MEMBERS WITH DIABETES**	**SUGGESTIONS APPROVED / JUSTIFICATIONS**	**APPROVAL**
Review the numerical description of each exercise because a nonsequential emergence may cause insecurity with respect to completing the weekly programme.	The numerical sequence was corrected.	100%
Include a step-by-step description of the stages that precede the exercises.	A tutorial was included to make each stage that precedes the exercises clearer.	100%
Difficulty navigating the software and accessing/registering for the first time.	Clearer information was provided on how to use the programme for the first time.	100%
Because software functioning depends on Internet access (it does not function offline), it cannot be used in any environment.	An offline application would be interesting but is not feasible at the moment because the functions contained in the software (such as exercises, sending questions to the specialists and interaction with other social media platforms) require an Internet connection.	86.87%
Sending an SMS or other more direct means could be more practical than sending emails.	Sending an SMS is a paid service that we cannot afford at the moment.	100%
Changing the chat feature to a forum could be much more useful.	The chat session was changed to a forum.	100%
Some exercises that require forcing the fingers open were impossible to perform.	Each user has different limitations. However, the fact that a user is unable to perform an exercise is no reason to skip it in the ‘game’. Assessments at the end of the exercise and the system will send exercises to train the affected region. With persistent training, it is possible to improve the specific exercise and train ‘forgotten’ regions. For this reason, we did not include the option of skipping an exercise. By trying to perform any movement, muscle strength and mobility in the region will improve, and this is the primary objective of the movements.	93.33%

All the suggestions made by specialists and users were analysed by the researchers and were incorporated and implemented in the software (Table 3). This new version of the software was presented once again to the panels in the second round of the validation process. Some of the suggestions could not be implemented but were justified and resubmitted to the panel for their approval. In this second round, the judges could only ‘approve’ or ‘disapprove’ of the changes.

In general, the main suggestions involved an indication of the sequence of steps on the homepage. The starting point was unclear, so we made it more evident where to begin. Also, the health professionals questioned the usability and correct interpretation of the effort scale. We added a clearer explanation in the tutorial and also next to the scale icon.

In the second round of the web software assessment, one health professional and five users with DM dropped out of the study, but we still had a sufficient number of judges. Therefore, eight diabetes specialists and 15 anonymous panel members with diabetes approved or disapproved of the changes. None of the items were rejected by either group (given the approval index of 70%). We obtained an approval rate of 100% from the DM specialists and 97% from the software users with DM, as detailed in Table 3. Consequently, no further rounds were needed.

## Discussion

SOPED was developed and validated with a high degree of agreement between DM specialists and people with DM at 100% and 97%, respectively. The software allows self-management and personalised care for patients, which is recommended by international consensus. The main feature and innovation of the software is the customised treatment that respects the physical capacities of each user[33] by ensuring that the software users recognise the purpose and importance of completing the appropriate effort scale.

One concern that had an average disapproval rating from health professionals of 15% and from people with diabetes of 22% was the lack of audio in the videos, which made it difficult or impossible for people with visual problems to use the software (42.9% of people with DM have some type of retinopathy)[34]. It was important to make the software intuitive and easy to use because diabetes is prevalent in people who are 20–79 years old [1].

SOPED encompasses some recommendations given by the International Consensus on the Diabetic Foot (2015)[2] for the care and prevention of diabetic foot complications: (1) inspect and examine the affected foot; (2) identify the affected foot; and (3) educate people, family and/or caregivers and health professionals. In accordance with previous recommendations for the prevention of foot ulcers, as a safety function, we added in the periodic examination (every 30 days) of feet to assess tissue integrity. It is mandatory for the continued use of the tool, and access is blocked if the user exhibits any preulcerative signs, such as sores, blisters or a developing ulcer. Similarly, users are not allowed upon the first use of the software to see the exercise instructions if they present any sign of tissue damage. If preulcerative signs are present, a clear recommendation is made to seek urgent medical care. For future versions of SOPED, daily inspection will be recommended in a more evident way, besides the already given recommendation that is included in the instructions. Since many aspects of the feet can change after initiating the exercises, a quick questionnaire can be included just before starting the next session.

To avoid a repetitive and monotonous exercise sequence, each session was designed to be conducted in a short period of time. The variation in exercises was also planned with gamification concepts[25,35]. The main component of the gamification aspects was the system created to reward each successful exercise execution, regardless of individual physical capacity.

Despite the questions raised and discussed in an attempt to increase adherence to the software, it will be important to conduct an intervention with the target population to analyse whether the stimuli will be effective for improving foot–ankle mobility and functionality and strengthening foot–ankle muscles. There is also a need to verify the long-term effectiveness of the proposed exercises in a controlled, randomised clinical trial. Nevertheless, positive results are expected because the effect of this type of intervention has already been proven to be efficient in promoting changes in DPN-related deficits [7,9,36,37].

This tool complements the traditional recommended interventions of foot inspection, podiatric care, shoes and prescriptions and can be suggested by ay health professional because of its multiprofessional characteristic. The software was designed to be used at health centres as a self-explanatory tool validated by professionals from various areas, hence making its use interdisciplinary. The final version of SOPED is available at the following link: <http://www.usp.br/labimph/soped/> for the desktop and also to download the mobile application.

## Conclusion

SOPED was developed based on scientific evidence and on a high level of agreement between health experts and users with diabetes. SOPED can be recommended by an interdisciplinary team and is a free preventive model that can be implemented in primary and secondary care as a complementary treatment for DPN. Further steps to validate the software in a larger population are recommended.

## Supporting information

S1 Appendix Algorithms used in the software. (PDF)

S2 Appendix Codes used in the software. (PDF)

S3 Appendix Therapeutic exercise protocol included in the software. (XLSX)
