# Supplementary material for: Rehabilitation technology for self-care: Customised foot and ankle exercise software for people with diabetes
Source: PLoS One. 2019 Jun 20;14(6):e0218560. doi: 10.1371/journal.pone.0218560 (PMC6586406; doi:10.1371/journal.pone.0218560)
Supplement: S1 Appendix — (PDF) [file pone.0218560.s001.pdf]

```
var resources = [
        "ruler", "images/ruler.png",
        "cursor", "images/cursor.png",
        "bt_normal", "images/bt_normal.png",
        "bt_hover", "images/bt_hover.png",
        "sound", "images/sound_bt.png",
        "soundover", "images/sound_over.png",
        "nosound", "images/nosound_bt.png",
        "","",
        "","",
];

var GAME_WIDTH  = 720;
var GAME_HEIGHT = 800;
var ALLOW_SOUND = true;

var renderer;
var stage;
var background;
var state;
var allSoundsDecoded = false;

var loadingText;
var titletext;

var soundSprite = [];


var FRAMERATE = 0.1;

var ruler, cursor, button, grade, dbGrade;
var frametime, lasttime, frameindex;
var framepos, direction;

var rect;

var lastVideo = "";
var videoSprite = null;
var texture = null;
var soundButton;

var CurrentTime, BreakTime;

function DoNothing() {}

function PauseGameSound()
{
        soundSprite["audio"].pause();
        /*
        for(i=0; i<Object.keys(soundSprite).length; i++)
           if(soundSprite[Object.keys(soundSprite)[i]].loop)
            soundSprite[Object.keys(soundSprite)[i]].pause();
        */
}

function PlayGameSound(SoundName, IsLoop)
{
        if(!ALLOW_SOUND || !allSoundsDecoded)
          return;

        soundSprite[SoundName].loop = IsLoop;

        if(IsLoop || SoundName == "applause")
          soundSprite[SoundName].volume = 0.5;
```

```
		if(SoundName == "boss" || SoundName == "harpa")
		  soundSprite[SoundName].volume = 0.25;

		soundSprite[SoundName].playFrom(0);
	}

	function Preload(vid)
	{
		resources[resources.length-3] = vid;
		resources[resources.length-4] = "video";

		resources[resources.length-1] = 
vid.replace(".mp4","").replace(".",",").replace("videos","audio")+".mp3";
		resources[resources.length-2] = "audio";
		//alert(resources[resources.length-1]);
		CreateRenderer();

		var Resource = PIXI.loaders.Resource;
		Resource.setExtensionLoadType("wav", 
Resource.LOAD_TYPE.XHR);
		Resource.setExtensionLoadType("mp3", 
Resource.LOAD_TYPE.XHR);
		Resource.setExtensionLoadType("ogg", 
Resource.LOAD_TYPE.XHR);
		Resource.setExtensionLoadType("webm", 
Resource.LOAD_TYPE.XHR);

		Resource.setExtensionXhrType("wav", 
Resource.XHR_RESPONSE_TYPE.BUFFER);
		Resource.setExtensionXhrType("mp3", 
Resource.XHR_RESPONSE_TYPE.BUFFER);
		Resource.setExtensionXhrType("ogg", 
Resource.XHR_RESPONSE_TYPE.BUFFER);
		Resource.setExtensionXhrType("webm", 
Resource.XHR_RESPONSE_TYPE.BUFFER);

		var loader = PIXI.loader;

		for(i=0; i<resources.length; i+=2)
			loader.add(resources[i], resources[i+1]);

		loader.on("progress", LoadProgressHandler);
		loader.on("complete", DecodeAudioFiles);

		loader.load();
	}

	function DecodeAudioFiles()
	{
		var soundsToDecode = 0, soundsDecoded = 0;
		var soundExtensions = ["wav", "mp3", "ogg", "webm"];

		try
		{
			var decodeHandler = function() {
				soundsDecoded += 1;

				loadingText.text = "Iniciando som 
"+soundsDecoded + " de "+soundsToDecode;
				loadingText.x = 
loadingText.width*0.5+(GAME_WIDTH-loadingText.width)*0.5;
				renderer.render(stage);

				if(soundsToDecode == soundsDecoded)
				{
```

```
                                        //alert("Audio decodificado com 
sucesso!");
                                        allSoundsDecoded = true;
                                        InitAll();
                                }
                        };


        Object.keys(PIXI.loader.resources).forEach(function(resource) {

                                var extension = 
PIXI.loader.resources[resource].url.split(".").pop();
                                if(soundExtensions.indexOf(extension) !== 
-1)
                                {
                                                soundsToDecode += 1;
                                                var xhr = 
PIXI.loader.resources[resource].xhr,
                                                url = 
PIXI.loader.resources[resource].url;

        soundSprite[PIXI.loader.resources[resource].name] = 
makeSound(url, decodeHandler.bind(this), false, xhr);
                                }
                        });
                }
                catch(err)
                {
                        alert("Falha ao decodificar audio: "+err);
                        allSoundsDecoded = true;
                        InitAll();
                }
        }


        function loadResource(resourceName, resourcePath) {

                var loader = PIXI.loader;
                loadingText.visible = true;

                loader.add(resourceName, resourcePath);

                loader.on("progress", LoadProgressHandler);
                loader.on("complete", PlayGameVideo);

                loader.load();
        }

        function LoadProgressHandler(loader, resource)
        {
                loadingText.text = loader.progress.toFixed(2) + "%";
                loadingText.x = loadingText.width*0.5+(GAME_WIDTH-
loadingText.width)*0.5;
                renderer.render(stage);
        }

        function loadVideo(res) {
                lastVideo = res;
                //loadResource(res, res);
        }


        function InitAll()
        {
```

```
                loadingText.visible = false;

                AllocateAll();


                CreateScene();
                Animate();
        }

        function CreateRenderer()
        {
                state = DoNothing;
                dbGrade = 0;

                var rendererOptions = {antialiasing: true, transparent: 
true, resolution: window.devicePixelRatio, autoResize: false, view : 
document.getElementById("game"), backgroundColor : 0xD7D7D7};
                renderer = PIXI.autoDetectRenderer(GAME_WIDTH, 
GAME_HEIGHT, rendererOptions);

                stage = new PIXI.Container();
                ResizeRenderer();

                window.addEventListener("resize", ResizeRenderer);
                window.addEventListener("focus", ResizeRenderer);

                var style = {font : 'bold  20px Arial', fill : 
'#000000'};
                loadingText = new PIXI.Text('0.00 %',style);
                loadingText.anchor.x = 0.5;
                loadingText.anchor.y = 0.5;
                loadingText.x = loadingText.width*0.5+(GAME_WIDTH-
loadingText.width)*0.5;
                loadingText.y = loadingText.height*2;

                loadingText.visible = true;
                stage.addChild(loadingText);

                renderer.render(stage);
        }

        function Animate()
        {
                state();

                renderer.render(stage);
                requestAnimationFrame(Animate);
        }


        function ResizeRenderer()
        {
                if(window.innerHeight > window.innerWidth && 
window.innerWidth < GAME_WIDTH)
                {

                        renderer.view.style.width = window.innerWidth + 
"px";
                        renderer.view.style.height = 
(renderer.view.style.width*GAME_HEIGHT/GAME_WIDTH) + "px";
                        renderer.view.style.display = "block";
                }
        }
```

```
function CreateScene()
{
        lasttime = new Date().getTime();
        frameindex = 0;
        framepos = 0;
        frametime = FRAMERATE;

        offy = 500;

        ruler.position.x = 360;
        ruler.anchor.set(0.5);
        ruler.position.y = 115+offy;
        ruler.alpha = 1;
        ruler.interactive = true;
        ruler.buttonMode = true;
        ruler.mousedown = ObjectClick;
        ruler.touchstart = ObjectClick;

        cursor.anchor.set(0.5);
        cursor.position.x = cursor.width*0.5+8.0;
        cursor.position.y = cursor.height*0.5+offy;
        cursor.initialPosition = cursor.position.x;
        cursor.endPosition = ruler.width-cursor.width*0.5-8;
        cursor.alpha = 1;
        MakeMoveble(cursor);

        grade.anchor.set(0.5);
        grade.x = grade.width*0.5+(GAME_WIDTH-grade.width)*0.5;
        grade.y = ruler.height+4*grade.height+offy;

        button.interactive = true;
        button.buttonMode = true;
        button.anchor.set(0.5);
        button.x = button.width*0.5+(GAME_WIDTH-
button.width)*0.5;
        button.y = ruler.y+150;

        button.mouseover = ButtonOver;
        button.mouseout = ButtonOut;
        button.mousedown = ButtonClick;
        button.touchstart = ButtonClick;

        soundButton = new
PIXI.Sprite(PIXI.loader.resources.sound.texture);


        soundButton.position.x = soundButton.width*0.5;
        soundButton.position.y = soundButton.height*0.5;
        soundButton.scale.set(0.8);
        soundButton.anchor.x = 0.5;
        soundButton.anchor.y = 0.5;
        soundButton.interactive = true;
        soundButton.buttonMode = true;

        soundButton.mousedown = SoundClick;
        soundButton.mouseover = ChangeSoundButton;
        soundButton.mouseout = ChangeSoundButtonOut;
        soundButton.touchstart = SoundClick;


        stage.addChild(ruler);
        stage.addChild(cursor);
        stage.addChild(grade);
        stage.addChild(button);
```

```
		renderer.plugins.interaction.autoPreventDefault = false;
		StartTimeSync();
	}

	function SoundClick(event)
	{
			this.toggle = !this.toggle;
			if(this.toggle)
			{
				this.texture =
PIXI.loader.resources.nosound.texture;
				ALLOW_SOUND = false;
				PauseGameSound();
			}
			else
			{
				this.texture =
PIXI.loader.resources.sound.texture;
				ALLOW_SOUND = true;
				soundSprite["audio"].play();
			}
	}


	function ChangeSoundButton(event)
	{
		if(!this.toggle)
			this.texture =
PIXI.loader.resources.soundover.texture;
	}

	function ChangeSoundButtonOut(event)
	{
		if(this.toggle)
			this.texture =
PIXI.loader.resources.nosound.texture;
		else
			this.texture =
PIXI.loader.resources.sound.texture;
	}

	function PlayGameVideo()
	{
		var texture =
PIXI.Texture.fromVideo(resources[resources.length-3]);
		var videoSprite = new PIXI.Sprite(texture);

		videoSprite.width = 716;
		videoSprite.height = 476;
		videoSprite.anchor.set(0.5);

		videoSprite.x = 360;
		videoSprite.y = 240;

		stage.addChild(videoSprite);

		texture.baseTexture.source.volume = 0;
		texture.baseTexture.source.currentTime = 0;
		texture.baseTexture.source.play();
		texture.baseTexture.source.loop = true;
	}

	function MakePauseBreak() {
```

```
                    if(!BreakTime)
                    {
                         if(GetTimeDiff() > 5)
                         {
                            BreakTime = true;
                            loadingText.text = '';
                            PlayGameVideo();
                            PlayGameSound("audio", false);
                            stage.addChild(soundButton);
                         }
                         else
                         {
                            loadingText.text =
jQuery('#program_time').html()+Math.round(5-GetTimeDiff());
                         }

                         loadingText.x = loadingText.width*0.5+(GAME_WIDTH-
loadingText.width)*0.5;
                      }

        }

        function StartTimeSync()
        {
                stage.removeChild(loadingText);

                var style = {font : 'bold  20px Arial', fill :
'#ffffff'};
                loadingText = new PIXI.Text('0.00 %',style);
                loadingText.anchor.x = 0.5;
                loadingText.anchor.y = 0.5;
                loadingText.x = (GAME_WIDTH-loadingText.width)*0.5;
                loadingText.y = 100+loadingText.height*2;
                loadingText.visible = true;

                stage.addChild(loadingText);

                BreakTime = false;
                loadingText.visible = true;
                LastTime = new Date().getTime();
                state = MakePauseBreak;
        }

        function GetTimeDiff()
        {
                return 0.001*(new Date().getTime()-LastTime);
        }

        function ButtonClick(event)
        {
                SetGrade(dbGrade.toFixed(1));
        }

        function ButtonOver(event)
        {
                this.texture = PIXI.loader.resources.bt_hover.texture;
        }

        function ButtonOut(event)
        {
                this.texture = PIXI.loader.resources.bt_normal.texture;
        }

        function ObjectClick(event)
        {
```

```
                        var newPosition = 
event.data.getLocalPosition(this.parent);

                        if(newPosition.x < cursor.initialPosition)
                          cursor.position.x = cursor.initialPosition;
                        else if(newPosition.x > cursor.endPosition)
                          cursor.position.x = cursor.endPosition;
                        else
                          cursor.position.x = newPosition.x;

                        dbGrade =  ((cursor.position.x-
cursor.initialPosition)*10.0/(cursor.endPosition - 
cursor.initialPosition));
                        grade.text = dbGrade.toFixed(1);

                        if(dbGrade < 2.5)
                          grade.text += " - 
"+jQuery('#program_status1').html();
                        else if(dbGrade < 5.0)
                          grade.text += " - 
"+jQuery('#program_status2').html();
                        else if(dbGrade < 7.5)
                          grade.text += " - 
"+jQuery('#program_status3').html();
                        else
                          grade.text += " - 
"+jQuery('#program_status4').html();

                        grade.x = grade.width*0.5+(GAME_WIDTH-
grade.width)*0.5;
        }

        function MakeMoveble(obj)
        {
                obj.interactive = true;
                obj.buttonMode = true;

                obj.mousedown = ObjectDragStart;
                obj.touchstart = ObjectDragStart;
                obj.mouseup = ObjectDragEnd;
                obj.touchend = ObjectDragEnd;
                obj.mouseupoutside = ObjectDragEnd;
                obj.touchendoutside = ObjectDragEnd;
                obj.mousemove = ObjectDragMove;
                obj.touchmove = ObjectDragMove;
        }

        function ObjectDragStart(event)
        {
                this.alpha = 1;
                this.dragging = true;
        }

        function ObjectDragMove(event)
        {
                if (this.dragging)
                {
                        var newPosition = 
event.data.getLocalPosition(this.parent);

                        if(newPosition.x < this.initialPosition)
                          this.position.x = this.initialPosition;
                        else if(newPosition.x > this.endPosition)
                          this.position.x = this.endPosition;
                        else
```

```
                        this.position.x = newPosition.x;

                      dbGrade =  ((this.position.x-
this.initialPosition)*10.0/(this.endPosition - this.initialPosition));
                      grade.text = dbGrade.toFixed(1);

                      if(dbGrade < 2.5)
                        grade.text += " -
"+jQuery('#program_status1').html();
                      else if(dbGrade < 5.0)
                        grade.text += " -
"+jQuery('#program_status2').html();
                      else if(dbGrade < 7.5)
                        grade.text += " -
"+jQuery('#program_status3').html();
                      else
                        grade.text += " -
"+jQuery('#program_status4').html();

                      grade.x = grade.width*0.5+(GAME_WIDTH-
grade.width)*0.5;
                }
        }

        function ObjectDragEnd(event)
        {
                this.alpha = 1;
                this.dragging = false;
        }

        function AllocateAll()
        {
                ruler = new
PIXI.Sprite(PIXI.loader.resources.ruler.texture);
                cursor = new
PIXI.Sprite(PIXI.loader.resources.cursor.texture);
                button = new
PIXI.Sprite(PIXI.loader.resources.bt_normal.texture);
                grade = new PIXI.Text('0 -
'+jQuery('#program_status1').html(),{font : 'bold  20px Arial', fill :
'#000000'});

                rect = new PIXI.Graphics();

                rect.beginFill(0x333333);
                rect.lineStyle(2, 0x333333);

                rect.drawRect(0, 0, 720, 479);
                stage.addChild(rect);
        }


<?php session_start(); if(isset($_GET['page'])) $page=$_GET['page']; else
if(isset($_POST['page'])) $page = $_POST['page']; else $page =
'apresentacao'; ?>

<?php

        if(isset($_SESSION['email']) && $page == "start")
        {
           if(isset($_GET['action']) && $_GET['action']  == "logout")
             session_destroy();
           else
             $page = "user_start";
        }
```

```
        if(!isset($_SESSION["language_code"]))
                $_SESSION["language_code"] = "pt_br";

        if(isset($_GET["lc"]))
                $_SESSION["language_code"] = $_GET["lc"];

        $langcode = $_SESSION["language_code"];

        function getLanguage($str, $defaut = "") {

                $language = "pt_br";

                if(isset($_SESSION["language_code"]))
                        $language = $_SESSION["language_code"];
                else
                        $_SESSION["language_code"] = $language;

                if($language == "pt_br")
                        return $defaut;

                include("php/languages/".$language.".php");

                return $translations[trim($str)];
        }
?>


<!doctype html>
<html lang="en-US" class="no-js">
<head>
        <?php include('php/head.php'); ?>
</head>

<body class="page page-id-2566 page-template-default rt-loading centered-
contents layout1 wpb-js-composer js-comp-ver-4.11.1 vc_responsive">

        <div id="loader-wrapper">
                <div id="loader"></div>
        </div>

        <div id="container">

                <div id="left_side" class="fixed_position scroll classic
active shadow" data-parallax-effect="1">

                        <div class="left-side-background-holder">
                                <div class="left-side-background"></div>
                        </div>

                        <div id="side_content" class="centered-contents"
data-position-y="0">

                                <div id="logo" class="site-logo"
style='margin-top: 30px;'>

                                        <?php
include('php/top_content.php'); ?><br/>
                                         <a href="index.php"
title="SAEDD"><img src="images/logos/logo_<?php echo $langcode; ?>.png"
alt="SAEDD" style='width: 250px;' /></a>
                                </div>


                                <?php include('php/menu.php'); ?>
```

```
				</div>

			</div>

			<div id="right_side" data-scrool-top="">

				<?php include('php/responsive-menu.php'); ?>

				<div id="main_content">
					<?php include('php/pages/'.$page.'.php');
?>
				</div>

				<?php include('php/footer.php'); ?>
			</div>

		</div>

		<script type='text/javascript'
src='js/bootstrap.min.js?ver=4.3.3'></script>
		<script type='text/javascript'
src='js/isotope.pkgd.min.js?ver=4.3.3'></script>
		<script type='text/javascript'
src='js/imagesloaded.min.js?ver=4.3.3'></script>
		<script type='text/javascript'
src='js/owl.carousel.min.js?ver=4.3.3'></script>
		<script type='text/javascript'
src='js/jflickrfeed.min.js?ver=4.3.3'></script>
		<script type='text/javascript'
src='js/customselect.min.js?ver=4.3.3'></script>

		<script type='text/javascript'
src='js/placeholders.min.js?ver=4.3.3'></script>
		<script type='text/javascript'
src='plugins/js_composer/assets/lib/waypoints/waypoints.min.js?ver=4.11.1
'></script>
		<script type='text/javascript'
src='js/jquery.vide.min.js?ver=4.3.3'></script>
		<script type='text/javascript'>
			/* <![CDATA[ */
				var mejsL10n = {"language":"en-
US","strings":{"Close":"Close","Fullscreen":"Fullscreen","Download
File":"Download File","Download Video":"Download
Video","Play\/Pause":"Play\/Pause","Mute Toggle":"Mute
Toggle","None":"None","Turn off Fullscreen":"Turn off Fullscreen","Go
Fullscreen":"Go
Fullscreen","Unmute":"Unmute","Mute":"Mute","Captions\/Subtitles":"Captio
ns\/Subtitles"}};
				var _wpmejsSettings = {"pluginPath":"\/wp-
includes\/js\/mediaelement\/"};
			/* ]]> */
		</script>
		<script type='text/javascript' src='js/mediaelement-and-
player.min.js?ver=2.17.0'></script>
		<script type='text/javascript'
src='js/scripts.js?ver=4.3.3'></script>
		<script type='text/javascript' src='js/comment-
reply.min.js?ver=4.3.3'></script>
		<script type='text/javascript'
src='plugins/js_composer/assets/js/dist/js_composer_front.min.js?ver=4.11
.1'></script>
```

```
        <?php
                if($langcode != "pt_br")
                        echo "
                                <script type='text/javascript'
src='js/language/$langcode.js'></script>

                <script>

        if(usMessages !== null && usMessages !== undefined) {

                var keys = Object.keys(usMessages);
                var values = Object.keys(usMessages).map(function(key)
{return usMessages[key];});

                for(i=0; i<keys.length; i++)
                        if(document.getElementById(keys[i]) !== null &&
document.getElementById(keys[i]) !== 'undefined')

        document.getElementById(keys[i]).innerHTML = values[i];


        }
                </script>

                ";
        ?>

</body>
</html>
<footer id="footer" class="clearfix footer" data-footer="">

        <div class="content_row row clearfix footer_contents
footer_info_bar fullwidth">
                <div class="content_row_wrapper default clearfix">
                        <div class="col col-sm-12">
                                <div class="copyright"
id='footer_copy'>Copyright &copy; LaBiMPH - USP | Todos os direitos
reservados</div>
                                <ul id="footer-navigation" class="menu">
                                        <li id="menu-item-2824"
class="menu-item menu-item-type-custom menu-item-object-custom menu-item-
2824"><a href="index.php?page=terms" id="footer_terms">Termos de uso do
SoPed</a></li>
                                        <li id="menu-item-2825"
class="menu-item menu-item-type-custom menu-item-object-custom menu-item-
2825"><a href="index.php?page=privacy" id="footer_privacy">Política de
Privacidade</a></li>
                                </ul>
                        </div>
                </div>
        </div>
</footer>
<?php
        function check($test, $page)
        {
                if($test == $page)
                  echo " current-menu-ancestor current-menu-parent
current_page_parent current_page_ancestor ";
        }
?>

<div class="navigation_holder side-element" style="display: block;
height: 100%; background: #0099B9;">
```

```
        <ul id="navigation" class="menu">

                <li id='menu-item-2836'  class="menu-item menu-item-type-
post_type <?php check("start", $page); ?> menu-item-object-page" data-
depth='0'>
                        <a  href="index.php?page=start" id='menu_i1'>
&lt;&lt;  INICIE AQUI!  &gt;&gt;</a>
                </li>

                <li id='menu-item-2867'  class="menu-item menu-item-type-
post_type menu-item-object-page <?php check("apresentacao", $page);
check("publicacoes", $page); check("projeto", $page); ?> menu-item-has-
children" data-depth='0'>
                        <a  href="index.php" id='menu_i2'>O PROJETO</a>
                        <ul class="sub-menu">
                                <li id='menu-item-2868'  class="menu-item
menu-item-type-post_type menu-item-object-page" data-depth='1'><a
href="index.php"  id='menu_i2s1'>Importância dos exercícios</a></li>
                                <li id='menu-item-2870'  class="menu-item
menu-item-type-post_type menu-item-object-page" data-depth='1'><a
href="index.php?page=projeto" id='menu_i2s2'>Conheça o Projeto</a></li>
                                <li id='menu-item-2869'  class="menu-item
menu-item-type-post_type menu-item-object-page" data-depth='1'><a
href="index.php?page=publicacoes" id='menu_i2s3'>Publicações</a></li>
                        </ul>
                </li>
                <li id='menu-item-2833'  class="menu-item menu-item-type-
post_type menu-item-object-page <?php check("equipe", $page);
check("colaboradores", $page); ?> menu-item-has-children" data-depth='0'>
                        <a  href="index.php?page=equipe"
id='menu_i3'>NOSSA EQUIPE</a>
                        <ul class="sub-menu">
                                <li id='menu-item-2854'  class="menu-item
menu-item-type-post_type menu-item-object-page" data-depth='1'><a
href="index.php?page=equipe" id='menu_i3s1'>Quem somos</a></li>
                                <li id='menu-item-2855'  class="menu-item
menu-item-type-post_type menu-item-object-page" data-depth='1'><a
href="index.php?page=colaboradores" id='menu_i3s2'>Colaboradores</a></li>
                        </ul>
                </li>


                <li id='menu-item-2845'  class="menu-item menu-item-type-
post_type <?php check("noticias", $page); check("eventos", $page); ?>
menu-item-object-page menu-item-has-children" data-depth='0'>
                        <a  href="index.php?page=noticias"
id="menu_i4">NOTÍCIAS E EVENTOS</a>

                        <ul class="sub-menu">
                                <li id='menu-item-2813'  class="menu-item
menu-item-type-taxonomy menu-item-object-portfolio_categories" data-
depth='1'><a href="index.php?page=noticias" id="menu_i4s1">Notícias</a>
</li>
                                <li id='menu-item-2814'  class="menu-item
menu-item-type-taxonomy menu-item-object-portfolio_categories" data-
depth='1'><a href="index.php?page=eventos" id="menu_i4s2">Eventos</a>
</li>
                        </ul>

                </li>

                <li id='menu-item-2837'  class="menu-item menu-item-type-
post_type <?php check("perguntar", $page); check("diabetes", $page);
check("polineuropatia", $page); check("cuidados", $page); check("dicas",
$page); ?> menu-item-object-page menu-item-has-children" data-depth='0'>
```

```
                        <a  href="index.php?page=perguntar"
id="menu_i5">DICAS DE ESPECIALISTAS</a>
                        <ul class="sub-menu">
                                <li id='menu-item-2849'  class="menu-item
menu-item-type-post_type menu-item-object-page" data-depth='1'><a
href="index.php?page=perguntar" id="menu_i5s1">Escrever Perguntas</a>
</li>
                                <li id='menu-item-2819'  class="menu-item
menu-item-type-taxonomy menu-item-object-portfolio_categories" data-
depth='1'><a href="index.php?page=diabetes" id="menu_i5s2">Diabetes e
saúde</a> </li>
                                <li id='menu-item-2819'  class="menu-item
menu-item-type-taxonomy menu-item-object-portfolio_categories" data-
depth='1'><a href="index.php?page=polineuropatia"
id="menu_i5s3">Polineuropatia</a> </li>
                                <li id='menu-item-2819'  class="menu-item
menu-item-type-taxonomy menu-item-object-portfolio_categories" data-
depth='1'><a href="index.php?page=cuidados" id="menu_i5s4">Cuidados com
os pés</a> </li>
                        </ul>
                </li>
                <li id='menu-item-2835'  class="menu-item menu-item-type-
post_type <?php check("contato", $page); ?> menu-item-object-page" data-
depth='0'><a  href="index.php?page=contato" id="menu_i6">CONTATO</a>
</li>
        </ul>
</div>
```

```
<div class="widgets_holder side-element sidebar-widgets">
        <div class="widget widget_contact_info">
                <a href="app.apk"><h5 id="menu_app">Baixe nosso
aplicativo</h5></a>
                <div class="with_icons style-1">
                        <a href="http://www.usp.br/labimph/"
target="_blank"><img src="images/labimph.png" style="width: 220px;"
/></a><br/><br/>
                        <div>
                                <span class="icon icon-home"></span>
                                <div id="menu_address">USP, LabiMPH, Rua
Cipotânea 51, Cidade Universitária, São Paulo/SP<br/>CEP: 05360-160</div>
                        </div>
                        <div>
                                <span class="icon icon-phone"></span>
                                <div><b id="menu_contact">Contato:
</b>+55 (11) 3091-8426 </div>
                        </div>
                        <div>
                                <span class="icon icon-mail-1"></span>
                                <div>labimphfofito@gmail.com</div>
                        </div>
                </div>
        </div>
        <div class="widget widget_social_media_icons">
                <ul class="social_media">

                        <li class="facebook"><a class="icon-facebook"
target="_self" href="" title="Facebook"><span>Facebook</span></a></li>
                        <li class="youtube-play"><a class="icon-youtube-
play" target="_self" href=""
title="YouTube"><span>Youtube</span></a></li>
                </ul>
        </div>
```

```
</div>
<?php

class ScriptDB
{
        private $Link;
        private $Connection;
        private $ResultSet;
        private $RecordCount;
        private $ColumnCount;

        private $KeepSequence;
        private $PrimaryKey;
        private $ListBoxField;
        private $OrderBy;

        private $TableName;
        private $FieldsName;
        private $FieldsAlias;
        private $FieldsCount;
        private $ViewColumns;
        private $ViewColSpan;
        private $ViewRowSpan;
        private $Components;

        private $SelectValues  = array();
        private $SelectCaption = array();
        private $BeforeValue   = array();

        //////////////////////////////////////////////
        //             INTERFACE METHODS            //
        //////////////////////////////////////////////

        function __construct()
        {
                $this->KeepSequence = true;
                $this->OrderBy = "";
        }

        function Set($Property, $Value)
        {
                if(strpos($Value,"|") > 0 && !strpos($Value,"||"))

                        $this->$Property = explode("|",$Value);
                else
                        $this->$Property = $Value;
        }


        function Get($Property)
        {
                return $this->$Property;
        }


        //////////////////////////////////////////////
        //             DATABASE METHODS             //
        //////////////////////////////////////////////


        function Connect()
        {
                $server = "mysql.uttersensor.com";
                $user ="uttersensor";
                $pass ="ali1420";
```

```
            $database ="uttersensor";

            $this->Connection = mysql_connect($server,$user,$pass);
            mysql_select_db($database, $this->Connection);

            if(!$this->Connection)
                    return false;

            return true;
    }


    function ExecQuery($sql)
    {
            if(!$this->Connect())
                    return false;

            $this->ResultSet = mysql_query($sql, $this->Connection);

            if (!$this->ResultSet)
                    return false;

            if(is_bool($this->ResultSet))
               return true;

            $this->RecordCount = mysql_num_rows($this->ResultSet);
            $this->ColumnCount = mysql_num_fields($this->ResultSet);

            return true;
    }


    function IsValidDate($date)
    {
            if(!isset($date) || $date == "")
                    return false;

            list($dd,$mm,$yy) = explode("/",$date);

            if($dd != "" && $mm != "" && $yy != "")
            {
               if(intval($dd) == 0 || intval($mm) == 0 || intval($yy)
== 0)
                 return false;
               else
                    return checkdate($mm,$dd,$yy);
            }

            return false;
    }


    function BeforeInsert($FieldName, $Values)
    {
            $this->BeforeAdd[$FieldName] = $Values;
    }

    function BeforeUpdate($FieldName, $Values)
    {
            $this->BeforeAdd[$FieldName] = $Values;
    }

    function CreateInsert()
    {
          $this->FieldsCount = count($this->FieldsName);
```

```
		$sql = "INSERT INTO ".$this->TableName." (";

		$sql .= $this->FieldsName[0];
		for($i=1; $i<$this->FieldsCount; $i++)
			$sql .= ",".$this->FieldsName[$i];

		$sql .= ") VALUES(";

		for($i=0; $i<$this->FieldsCount; $i++)
		{
			$Value = $_POST[$this->FieldsName[$i]];

			if(strlen($Value) == 0 || $Value == "NULL")
				$Value = "NULL";
			else if($this->IsValidDate($Value))
				$Value = "STR_TO_DATE('$Value',
'%d/%m/%Y')";
			else if($this->Components[$i] == "hidden" &&
substr($Value,0,1) == "@")
				$Value = substr($Value,2,strlen($Value));
			else
				$Value = "'".str_replace("\\''","''",
str_replace("'", "''", $Value))."'";

			if(isset($this->BeforeAdd[$this-
>FieldsName[$i]]))
				$Value = $this->BeforeAdd[$this-
>FieldsName[$i]];

			$sql .= $Value.",";
		}

		$sql = substr($sql,0,-1).");";

		return $sql;
	}


	function CreateUpdate()
	{
	     $this->FieldsCount = count($this->FieldsName);

		$sql = "UPDATE ".$this->TableName." SET ";

		for($i=0; $i<$this->FieldsCount; $i++)
		{
			if($this->FieldsName[$i] == $this->PrimaryKey)
				continue;

			$Value = $_POST[$this->FieldsName[$i]];

			if(strlen($Value) == 0)
				$Value = "NULL";
			else if($this->IsValidDate($Value))
				$Value =
"to_date('$Value','DD/MM/YYYY')";
			else if($this->Components[$i] == "hidden" &&
substr($Value,0,1) == "@")
				$Value = substr($Value,2,strlen($Value));
			else
				$Value = "'".str_replace("'", "''",
$Value)."'";
```

```
                        if(isset($this->BeforeAdd[$this-
>FieldsName[$i]]))
                                $Value = $this->BeforeAdd[$this-
>FieldsName[$i]];

                        $sql .= $this->FieldsName[$i] ." = ". $Value.",";
                }

                $sql = substr($sql,0,-1)." WHERE ".$this->PrimaryKey." =
'".$_POST[$this->PrimaryKey]."'";

                return $sql;
        }

        function CreateDelete()
        {
                if($this->KeepSequence)
                        return "DELETE FROM ".$this->TableName." WHERE
".$this->PrimaryKey." = '".$_POST[$this->PrimaryKey]."'; UPDATE ".$this-
>TableName." SET ".$this->PrimaryKey." = '".$_POST[$this->PrimaryKey]."'
WHERE ".$this->PrimaryKey." = (SELECT COALESCE(MAX(".$this-
>PrimaryKey."),-1) FROM ".$this->TableName.") AND  ".$this->PrimaryKey."
> '".$_POST[$this->PrimaryKey]."';";
                else
                        return "DELETE FROM ".$this->TableName." WHERE
".$this->PrimaryKey." = '".$_POST[$this->PrimaryKey]."';";
        }

        function GetLastError()
        {
                return mysql_error();
        }

        function ShowColumns($table)
        {
           $this->ExecQuery("SELECT COLUMN_NAME FROM
INFORMATION_SCHEMA.COLUMNS WHERE TABLE_NAME = '$table'");
           return $this->GetPippedCol(0);
        }

        function GetQueryValue($row, $col)
        {
                return mysql_result($this->ResultSet,$row,$col);
        }


        function GetPippedCol($col)
        {
                if($this->RecordCount == 0)
                  return "";

                $PipedValues = $this->GetQueryValue(0, $col);

                for($i=1; $i<$this->RecordCount; $i++)
                        $PipedValues .= "|".$this->GetQueryValue($i,
$col);

                return $PipedValues;
        }


        function GetFieldName($col)
        {
                return mysql_field_name($this->ResultSet, $col);
        }
```

```
        function CloseDB()
        {
                mysql_close($this->Connection);
        }



        ///////////////////////////////////////////////
        //           HTML STUFFS                     //
        ///////////////////////////////////////////////

        function GetPassword( $length = 8, $chars =
'abcdefghijklmnopqrstuvwxyz0123456789')
        {
                return substr(str_shuffle( $chars ), 0, $length );
        }

        function GetHtmlFieldValues()
        {
              $this->FieldsCount = count($this->FieldsName);

                $sql = "";
                for($i=0; $i<$this->FieldsCount; $i++)
                {
                   if(isset($_POST[$this->FieldsName[$i]]))
                     $sql .= "<b>".$this->FieldsName[$i].":</b>
".$_POST[$this->FieldsName[$i]]."<br/>";
                }
                return $sql;
        }

        function GetHtmlFromQuery($html, $row)
        {
                $sql = "";

                for($i=0; $i<$this->ColumnCount; $i++)
                   $sql .= str_replace("[-v-]",$this->GetQueryValue($row,
$i), str_replace("[-f-]",$this->GetFieldName($i), $html));

                return $sql;
        }

        function GetHtmlFromQueryCols($html, $row, $start, $end)
        {
                $sql = "";

                for($i=$start; $i<$end; $i++)
                   $sql .= str_replace("[-v-]",$this->GetQueryValue($row,
$i), str_replace("[-f-]",ucfirst($this->GetFieldName($i)),
$html))."<br/>";

                return $sql;
        }

        function QueryToTable($html)
        {
                $sql = "<table $html>";

                $sql .= "\t<tr>";
                for($i=0; $i<$this->ColumnCount; $i++)
                        $sql .= "<th>". $this->GetFieldName($i)."</th>";
                $sql .= "\t</tr>";
```

```
                for($i=0; $i<$this->RecordCount; $i++)
                {
                        $sql .= "\t\t<tr>";
                        for($j=0; $j<$this->ColumnCount; $j++)
                                $sql .= "<td>".$this->GetQueryValue($i,
$j)."</td>";
                        $sql .= "\t\t</tr>";
                }

                $sql .= "</table>";

                return $sql;
        }

        function GenerateForm($FormTitle, $UpdateArea)
        {
                $this->FieldsCount = count($this->FieldsName);
                $IsShowing = false;
                $FieldValue = "";

                if(isset($_POST['secrethiddenfield']))
                {
                        if($_POST['secrethiddenfield'] == "insert")
                        {
                                $this->ExecQuery($this->CreateInsert());
                                $this->GetLastError();
                                echo "<h3>Registro cadastrado com
sucesso!</h3><br/><a href=\"".$_SERVER["PHP_SELF"]."\">Clique aqui</a>
para retornar à página de cadastro.";
                                return 1;
                        }
                        else if($_POST['secrethiddenfield'] == "show")
                        {
                                $IsShowing = true;
                                $this->ExecQuery("SELECT ".implode(",",
$this->FieldsName)." FROM ". $this->TableName . " WHERE ".$this-
>PrimaryKey." = '".$_POST[$this->PrimaryKey]."' LIMIT 1");
                                echo $this->GetLastError();
                        }
                        else if($_POST['secrethiddenfield'] == "update")
                        {
                                $this->ExecQuery($this->CreateUpdate());
                                echo "<h3>Registro atualizado com
sucesso!</h3><br/><a href=\"".$_SERVER["PHP_SELF"]."\">Clique aqui</a>
para retornar à página de cadastro.";
                                return 2;
                        }
                        else if($_POST['secrethiddenfield'] == "delete")
                        {
                                $this->ExecQuery($this->CreateDelete());
                                echo "<h3>Registro deletado com
sucesso!</h3><br/><a href=\"".$_SERVER["PHP_SELF"]."\">Clique aqui</a>
para retornar à página de cadastro.";
                                return 3;
                        }
                }

                $html .= "<h2>".$FormTitle."</h2><br/>\n<form
name=\"".$this->TableName."\" method=\"POST\"
action=\"".$_SERVER["PHP_SELF"]."\">\n";
                $html .= "\n<table style=\"width: 100%;\">\n\n";

                for($i=0; $i<$this->FieldsCount; $i++)
                {
                        if($i%$this->ViewColumns == 0)
```

```
                        {
                                if($i == 0)
                                        $html .= "\t<tr>\n";
                                else
                                        $html .= "\n\t</tr>\n\t<tr>";
                        }

                        if($IsShowing)
                        {
                                if($this->Components[$i] == "select" ||
$this->Components[$i] == "textarea" )
                                        $FieldValue = $this-
>GetQueryValue(0, $i);
                                else
                                        $FieldValue = "value=\"".$this-
>GetQueryValue(0, $i)."\"";
                        }

                        if($this->Components[$i] == "text")
                                $html .=
"\t\t\n<td>\n\t\t\t\t<label><b>".$this-
>FieldsAlias[$i]."</b></label>\n<input type=\"text\" name=\"".$this-
>FieldsName[$i]."\"  style=\"width: 98%;\" ".$this->GetFieldExtra($this-
>FieldsName[$i])." $FieldValue /><br/><br/>\t\t\n</td>";
                        else if($this->Components[$i] == "select")
                                $html .=
"\t\t\n<td>\n\t\t\t<label><b>".$this-
>FieldsAlias[$i]."</b></label>\n<select type=\"text\" name=\"".$this-
>FieldsName[$i]."\"  style=\"width: 98%;\" ".$this->GetFieldExtra($this-
>FieldsName[$i])." >".$this->GetSelectValues($this->FieldsName[$i],
$FieldValue)."\t\t\n</select><br/><br/>\t\t</td>";
                        else if($this->Components[$i] == "textarea")
                                $html .= "\t\t\n<td
colspan=\"2\">\n\t\t\t<label><b>".$this-
>FieldsAlias[$i]."</b></label>\n<textarea name=\"".$this-
>FieldsName[$i]."\" style=\"resize: none; width: 99%; height: 100px;\"
".$this->GetFieldExtra($this-
>FieldsName[$i]).">$FieldValue</textarea><br/><br/>\t\t</td>";
                        else if($this->Components[$i] == "hidden")
                                $html .= "\t\t\n<td>\n\t\t\t<input
type=\"hidden\" name=\"".$this->FieldsName[$i]."\" ".$this-
>GetFieldExtra($this->FieldsName[$i])." $FieldValue />\t\t</td>";
                }

                $html .= "\n\t</tr>\n</table>";
                $html .= "<input type=\"hidden\"
name=\"secrethiddenfield\" value=\"\" />";

                if(!$IsShowing)
                        $html .= "\n\t<div style=\"width: 100%; text-
align: center;\"><input type=\"button\" value=\"Cadastrar\"
onclick=\"javascript: document.".$this-
>TableName.".secrethiddenfield.value='insert'; document.".$this-
>TableName.".submit();\" /></div>\n";
                else
                        $html .= "\n\t<input type=\"button\"
value=\"Atualizar Registro\" onclick=\"javascript: document.".$this-
>TableName.".secrethiddenfield.value='update'; document.".$this-
>TableName.".submit();\" />  <input style=\"float: right;\"
type=\"button\" value=\"Deletar Registro\" onclick=\"javascript:
if(confirm('Tem certeza que deseja deletar esse registro? Não será
possível recuperá-lo.')) {document.".$this-
>TableName.".secrethiddenfield.value='delete'; document.".$this-
>TableName.".submit();}\" /><br/>\n";
```

```
		if($UpdateArea)
		{
			$this->ExecQuery("SELECT ".$this->PrimaryKey.",
".$this->ListBoxField." FROM ".$this->TableName." ORDER BY 2 ".$this-
>OrderBy);
			$this->AddSelectValues($this->TableName, $this-
>GetPippedCol(0), $this->GetPippedCol(1));

			$html .=
"<br/><br/><hr/><br/>\t\t\n\n<label><b>Registros
cadastrados</b></label><br/>";
			$html .= "<select multiple=\"multiple\"
size=\"6\" ondblclick=\"javascript: document.".$this-
>TableName.".secrethiddenfield.value= 'show';  document.".$this-
>TableName.".".$this->Get("PrimaryKey").".value =
this.options[this.selectedIndex].value;  document.".$this-
>TableName.".submit();\" style=\"width: 100%;\"  >";

			$html .= $this->GetSelectValues($this-
>TableName);

			$html .= "\n</select>\n<br/><br/>\n";
		}

		$html .= "</form>";

		echo $html;
	}


	function AddFieldExtra($FieldName, $HtmlCode)
	{
		$this->FieldsExtra[$FieldName] = $HtmlCode;
	}


	function GetFieldExtra($FieldName)
	{
		if(!isset($this->FieldsExtra[$FieldName]))
			return "";

		return $this->FieldsExtra[$FieldName];
	}


	function AddSelectValues($FieldName, $Values, $Titles = null)
	{
		$this->SelectValues[$FieldName] = $Values;

		if($Titles == null)
			$this->SelectCaption[$FieldName] = $Values;
		else
			$this->SelectCaption[$FieldName] = $Titles;
	}


	function GetSelectValues($FieldName, $SelectedValue = "")
	{
		if(!isset($this->SelectValues[$FieldName]))
			return "";

		$ItemsValue = explode("|", $this-
>SelectValues[$FieldName]);
		$ItemsTitle = explode("|", $this-
>SelectCaption[$FieldName]);
```

```
		$ItemsCount = count($ItemsValue);

		$html = "";

		for($i=0; $i<$ItemsCount; $i++)
		{
			if($ItemsValue[$i] == $SelectedValue)
				$html .= "\n\t\t\t<option selected 
value=\"".$ItemsValue[$i]."\">".$ItemsTitle[$i]."</option>";
			else
				$html .= "\n\t\t\t<option 
value=\"".$ItemsValue[$i]."\">".$ItemsTitle[$i]."</option>";
		}

		return $html;
	}

	function QueryAsHtmlOptions($TextIndex, $ValueIndex)
	{
		$html = "";

		for($i=0; $i<$this->RecordCount; $i++)
		  $html .= "<option value=\"".$this->GetQueryValue($i, 
$ValueIndex)."\">".$this->GetQueryValue($i, $TextIndex)."</option>";

		return $html;
	}


	function TitleCase($string, $exceptions = 
array('da','de','do','e','dos','das','por','à','em','na','no','com',
'para'))
	{
		$string = 
strtolower(strtr($string,"ÁÉÍÓÚÂÊÔÃÕÀÈÌÒÙÇ","áéíóúâêôãõàèìòùç"));
		$words = explode(" ", $string);
		$newwords = array();

		foreach ($words as $word)
		{
			if(!in_array($word, $exceptions))
				$word = strtoupper(strtr(substr($word,0, 
1),"áéíóúâêôãõàèìòùç","ÁÉÍÓÚÂÊÔÃÕÀÈÌÒÙÇ")).strtolower(strtr(substr($word,
1),"ÁÉÍÓÚÂÊÔÃÕÀÈÌÒÙÇ","áéíóúâêôãõàèìòùç"));

			array_push($newwords, $word);
		}

		return join(" ", $newwords);
	}
}

?>

<?php


	if(isset($_POST['action']))
	{
		$action = $_POST['action'];

		if($action == "step1")
		{
```

```
        require_once('../MysqlDB.php');
                                                $Obj = new ScriptDB();

                                                $Obj->Set("TableName",
"saedd_occur_01");
                                                $Obj->Set("FieldsName",
"fr_q1|fr_q2|fr_q3|fr_q4|fl_q1|fl_q2|fl_q3|fl_q4|user_id|registration_dat
e");
                                                $Obj-
>BeforeInsert("registration_date","NOW()");

                                                $sql = $Obj-
>CreateInsert();

                                                $Obj->Connect();
                                                if($Obj->ExecQuery($sql))
                                                {
                                                        $sql = "INSERT
INTO saedd_quests(user_id,quest_datetime,quest)
VALUES('".$_POST['user_id']."',NOW(),'occurrence_1');";
                                                        $Obj-
>ExecQuery($sql);

                                                        echo "1";

                                                }
                                                else
                                                {
                                                          echo $Obj-
>GetLastError();
                                                }

                                                $Obj->CloseDB();


                }
                else if($action == "initial")
                {

        require_once('../MysqlDB.php');
                                                $Obj = new ScriptDB();
                                                $Obj->Connect();

                                                $sql = "INSERT INTO
saedd_quests(user_id,quest_datetime,quest)
VALUES('".$_POST['user_id']."',NOW(),'program_start');";
                                                $Obj->ExecQuery($sql);
                                                echo "1";

                                                $Obj->CloseDB();
                }
                return;
        }
?>

<?php include("php/pages/back.php"); ?>

<link rel='stylesheet' href='css/myform.css' type='text/css' media='all'
/>

<style type="text/css">
.myform input[type="text"],
.myform input[type="date"],
.myform input[type="datetime"],
```

```
.myform input[type="email"],
.myform input[type="number"],
.myform input[type="search"],
.myform input[type="time"],
.myform input[type="url"],
.myform input[type="password"],
.myform textarea,
.myform select {

    border-top: 1px solid #f0f0f0;
    border-left: 1px solid #f0f0f0;
    border-bottom: 1px solid #FAFAFA;
    border-right: 1px solid #FAFAFA;
    background: #e0e0e0;
}

.default-style input[type="button"]:hover {background-color: #FC9D0D;}
.default-style input[type="button"] {background-color: #428bca; font-
weight: bold;}

.default-style input[type="button"]:hover.extra  {background-color:
#FC9D0D;}
.default-style input[type="button"].extra  {background-color: #00AA00;
font-weight: bold;}
</style>


<script type="text/javascript">


        function AcceptTip()
        {
           jQuery.ajax({
                type: "POST",
                url: "php/pages/program.php",
                data: {user_id : <?php echo $_SESSION['id']; ?>,  action
: "initial"}
          })
         .done(function(html) {
                if(html.trim() == "1")
                {
                          window.location = 'index.php?page=program';
                }
                else
                  jQuery("#msg").html("Erro: '"+html+"'");
          });

     }


</script>



<?php
                        require_once('php/MysqlDB.php');
                        $id = $_SESSION['id'];

                        $Obj = new ScriptDB();
                        $Obj->Connect();
                        $Obj->ExecQuery("SELECT (SELECT COUNT(*) FROM
saedd_quests WHERE user_id = '$id' AND quest = 'program_start'),0");

                        $p1 = $Obj->GetQueryValue(0, 0);
                        $p2 = $Obj->GetQueryValue(0, 1);
```

```
?>

<script type='text/javascript'>
                var canReload = false;
                function SetGrade(num) {
                        jQuery("#difficult").val(num);
                        canReload = true;
                        updateAwards(5);
                }

                jQuery(document).ready(function() {
                        updateAwards(5);
                });

                function updateAwards(acc)
                {
                        jQuery("#saida").html(ShowInfo("Aguarde..."));

                        jQuery.ajax({
                                type: "POST",
                                url: "php/pages/protocolo_final2.php",
                                data: {action : acc, difficult :
jQuery("#difficult").val(), day : jQuery("#vday").val(), position :
jQuery("#vposition").val(), block : jQuery("#vblock").val()}
                        })
                        .done(function(html) {

                            jQuery("#saida").html("");
                            var item = html.split("|");
                            jQuery("#coin").html(item[0]);
                            jQuery("#medal").html(item[1]);
                            jQuery("#trophy").html(item[2]);
                            jQuery("#try").html(item[3]);
                            jQuery("#day").html(item[4]);

                           jQuery("#difficult").val("");

                            if(parseInt(item[5]) == 1) {

        jQuery("#rulerarea").css('display','none');

        jQuery("#videoarea").html("<br/><br/><h4><span>Parabéns você
concluiu todos os exercícios!</span></h4><br/><h3><span>Retorne essa
semana para dar continuidade ao seu programa de exercícios</span></h3>");
                                return;
                            }

                            if(acc == 5) {
                               jQuery("#exercise").html("B"+item[6]+"-
G"+item[7]);
                               jQuery("#videoarea").html(item[8]);
                               jQuery("#vday").val(item[4]);
                               jQuery("#vblock").val(item[6]);
                               jQuery("#vposition").val(item[7]);

                                if(canReload)
                                  window.location =
'index.php?page=program';
                                else
                                  Preload(item[9]);
```

```
                            }

                          if(acc == 4)
                             window.location = 'index.php?page=program';


                        });
                }
</script>


<script type='text/javascript' src='js/pixi.min.js'></script>
<script type='text/javascript' src='js/sound.js'></script>
<script type='text/javascript' src='js/ruler_video.js'></script>

<style type="text/css">
        h4 span {color: #428BCA;}
</style>

<script type="text/javascript">

        var pos = 1;

        function showArea() {

                for(var i=1; i<=6; i++)
                  jQuery("#area"+i).css("display","none");

                if(pos <= 6) {
                  pos++;

                jQuery("#area"+pos).css("display","inline");

                if(pos > 1)
                 jQuery("#btstart").css("display","inline");
                else
                 jQuery("#btstart").css("display","none");

                if(pos >= 6) {
                   jQuery("#btend").css("display","none");
                   jQuery("#btstart").css("display","none");
                }


                return;
                }
        }

        function backArea() {

                for(var i=1; i<=6; i++)
                  jQuery("#area"+i).css("display","none");


                if(pos > 1) {
                  pos -= 1;

                jQuery("#area"+pos ).css("display","inline");

                if(pos > 1)
                 jQuery("#btstart").css("display","inline");
                else
                 jQuery("#btstart").css("display","none");

                if(pos >= 6) {
```

```
                    jQuery("#btend").css("display","none");
                    jQuery("#btstart").css("display","none");
                }

                return;
                }
        }
</script>


<div  class="content_row default-style no-composer overlap fullwidth">
        <div class="content_row_wrapper clearfix " style="min-height:
800px; ">
                <div class="col col-sm-12 col-xs-12 ">
                        <div class="single post-1872 staff type-staff
status-publish has-post-thumbnail hentry" id="person-1872">


                        <div id='etapa1' style='display: <?php if($p1 ==
0) echo "inline"; else echo "none"; ?>'>



                                        <div id='area1' >
                                                <h2>PARABÉNS!</h2>
                                                Estamos felizes que você
tenha chegado até aqui!<br/><strong>Isso mostra que você tem interesse em
melhorar a saúde e a qualidade de vida dos seus pés.</strong><br/>
                                                Antes de iniciar os
exercícios gostaríamos de explicar algumas regras que tornarão o processo
mais divertido. <br/><br/><br/>

                                IMPORTANTE: Caso você não consiga
realizar os exercícios, peça ajuda para um familiar ou um profissional da
saúde.<br/><br/>
                                <b style='color: #FF0000;'>Certifique-se
que a sua glicemia está normal e que fará uso da sua medicação e/ou
insulina para que não sofra hiperglicemia. E não se esqueça de se
alimentar adequadamente!</b><br/><br/>

                                        </div>


                                        <div id='area2' style="display:
none;">
                                           <h2>RESUMO DE UMA PARTIDA -OU
SESSÃO</h2>
                                            Cada partida consiste na
realização de <b>8 exercícios para pés e tornozelos</b>.
                                            <br/>As partidas não
ultrapassam 20 minutos e podem ser executadas em qualquer ambiente de sua
escolha. <b>Recomendamos de 2 à 3 vezes por semana, sendo que não é
possível realizar 2 sessões no mesmo dia!</b><br/><br/>

                                            <h4>1 partida = 8 exercícios
(No máximo 20 minutos)</h4>

                                                <img
src="images/exercicio/man.png" style="width: 70px;">
                                                <img
src="images/exercicio/man.png" style="width: 70px;">
                                                <img
src="images/exercicio/man.png" style="width: 70px;">
                                                <img
src="images/exercicio/man.png" style="width: 70px;">
```

```
                                                <img
src="images/exercicio/man.png" style="width: 70px;">
                                                <img
src="images/exercicio/man.png" style="width: 70px;">
                                                <img
src="images/exercicio/man.png" style="width: 70px;">
                                                <img
src="images/exercicio/man.png" style="width: 70px;">
                                                <img
src="images/exercicio/right.png" >
                                                <img
src="images/date.png" >
                                                <br/><br/><br/>
                                        </div>


                                <div id='area3' style="display: none;">
                                        <h2>PREPARAÇÃO</h2>
                                        Para a realização dos exercícios
propostos tenha sempre por perto uma <b>toalha de mão, uma bolinha
pequena (que poderá ser de tênis, lisa ou cravo), uma bexiga (que deverá
previamente ser preenchida com maisena ou farinha de trigo), lápis,
algodão, uma faixa elástica (comumente utilizadas na fisioterapia ou um
elástico grosso)</b> e <span style='#color: #428BCA;'>muita
disposição</span>!<br/><br/>
                                                <h4>Reserve antes de uma
partida</h4>
                                                <img
src="images/exercicio/toalha.png" style="height: 80px;">
                                                <img
src="images/exercicio/tenis.png" style="height: 80px;">
                                                <img
src="images/exercicio/bexiga.png" style="height: 80px;">
                                                <img
src="images/exercicio/lapis.png" style="height: 80px;">
                                                <img
src="images/exercicio/thera.png" style="height: 80px;">
                                                <img
src="images/exercicio/algodao.png" style="height: 80px;">
                                                <img
src="images/exercicio/right.png" >
                                                <img
src="images/exercicio/sorriso.png" style="height: 80px;">
                                        <br/><br/><br/>
                                </div>

                                <div id='area4' style="display: none;">
                                        <h2>OBJETIVO</h2>
                                        Este programa de exercícios tem
como objetivo promover a realização de exercícios com foco nos
<strong>pés e tornozelos</strong>, com intuito de garantir uma boa
funcionalidade dos músculos e articulações que são comprometidos nas
pessoas com diabetes, especialmente as pessoas com polineuropatia.
                                        <br/><br/><strong>Funciona como
um jogo</strong> onde a chave para dominar o sistema depende de você!
Vamos entender como este jogo funciona?<br/><br/><br/>
                                </div>

                                <div id='area5' style="display: none;">
                                        <h2>Como funciona?</h2>
                                        Ao final de cada exercício você
mesmo avaliará a dificuldade de realizar o exercício, através de uma
escala de 0 à 10, sendo que 0 siginifia <b>NADA CANSATIVO</b> e 10
<b>MUITO CANSATIVO</b>.
                                        <br/><br/>
```

```
                                        <img src="images/ruler.png"
/><br/><br/><br/>
                                </div>

                                <div id='area6' style="display: none;">
                                        <h2>Recompensas</h2>
                                        A <strong>cada exercício</strong>
realização de um você será <strong>premiado com uma ficha</strong>,
independente se você achou fácil ou difícil.<br/>
                                        A cada exercício executado
<strong>sem dificuldade</strong> (Valores de 0 a 2 na régua), você
ganhará <strong>1 moeda</strong>.<br/>
                                        Com o acúmulo de <strong>8
moedas</strong>, você ganhará <strong>1 medalha</strong> e quando estiver
com <strong>2 medalhas</strong>, você ganhará <strong>um troféu</strong>
que lhe mudará de aumentará seu nível de experiência.<br/><br/>
                                        <strong>É de extrema importância
ser honesto consigo mesmo nas avaliações.</strong>
                                        <br/><br/>
                                        Dê o seu melhor e seja um expert
e compartilhe o seu desempenho com os seus amigos. Pronto para
começar?<br/><br/><br/>
                                        <br/><br/>
                                        <h4>Cada exercício = 1 ficha</h4>
                                        <img
src="images/exercicio/man.png" style="height: 80px;">
                                        <img
src="images/exercicio/right.png" >
                                        <img src="images/try.png"
style="height: 80px;"><br/><br/>

                                        <h4>Cada exercício sem
dificuldade = 1 moeda</h4>
                                        <img
src="images/exercicio/sorriso.png" style="height: 80px;">
                                        <img
src="images/exercicio/right.png" >
                                        <img src="images/coin.png"
style="height: 60px;"><br/><br/>

                                        <h4>Cada 8 moedas = 1
medalha</h4>
                                        <img src="images/coin.png"
style="height: 60px;">
                                        <img src="images/coin.png"
style="height: 60px;">
                                        <img src="images/coin.png"
style="height: 60px;">
                                        <img src="images/coin.png"
style="height: 60px;">
                                        <img src="images/coin.png"
style="height: 60px;">
                                        <img src="images/coin.png"
style="height: 60px;">
                                        <img src="images/coin.png"
style="height: 60px;">
                                        <img src="images/coin.png"
style="height: 60px;">
                                        <img
src="images/exercicio/right.png" >
                                        <img src="images/medal.png"
style="height: 60px;">
                                        <br/><br/>
```

```
                                        <h4>Cada 2 medalhas = 1 trofeu
(sobe de nível)</h4>
                                        <img src="images/medal.png"
style="height: 60px;">
                                        <img src="images/medal.png"
style="height: 60px;">
                                        <img
src="images/exercicio/right.png" >
                                        <img src="images/trophy.png"
style="height: 60px;">
                                        <img
src="images/exercicio/right.png" >
                                        <img
src="images/exercicio/levelup.png" style="height: 80px;">

                                        <br/><br/><br/>

                                        <br/><div id='msg' style='color:
#CC2233;'></div><br/>
                                        <input class="button submit"
onclick="javascript:AcceptTip();" value="Entendi, quero COMEÇAR!"
type="button">
                                </div>



                                <input type="button"
onclick="javascript:backArea();" id='btstart' style='display: none;'
value="Voltar para explicação anterior..." />
                                <input type="button"
onclick="javascript:showArea();" id='btend' value="Ok! Entendi e quero
continuar..." />




                        </div>



                        <div id='etapa1' style='display: <?php if($p1 !=
0) echo "inline"; else echo "none"; ?>'>


<?php
                                        $user_id = $_SESSION['id'];

                                        require_once('php/MysqlDB.php');
                                        $Obj = new ScriptDB();


                                        if(isset($_GET['start'])) {

                                                $Obj->ExecQuery("DELETE
FROM saedd_awards WHERE user_id = '$user_id'");
                                                $Obj->ExecQuery("DELETE
FROM saedd_protocol WHERE user_id = '$user_id'");
                                        }

                                        $Obj->ExecQuery("SELECT COUNT(*)
FROM saedd_awards WHERE user_id = '$user_id'");


                                        if(intval($Obj->GetQueryValue(0,
0)) == 0) {
```

```
                                                $sql = "INSERT INTO
saedd_awards(coins, medals, trophies, last_day, tries, user_id) VALUES(0,
0, 0, CURDATE(), 0, '$user_id')";

                                                if(!$Obj-
>ExecQuery($sql))
                                                  echo "<h2>Erro ao
inserir dados de recompensa.</h2>";
                                        }


                                        $Obj->ExecQuery("SELECT COUNT(*)
FROM saedd_protocol WHERE user_id = '$user_id'");


                                        if(intval($Obj->GetQueryValue(0,
0)) == 0) {

                                                $sql = "INSERT INTO
saedd_protocol(protocol, block, position, number, difficult, user_id,
registration_date) SELECT protocol, block, position, exercise, NULL,
'$user_id', NULL FROM saedd_blocks WHERE protocol = COALESCE((SELECT
protocol FROM saedd_users WHERE id = '$user_id'), NULL, 1)";

                                                if(!$Obj-
>ExecQuery($sql))
                                                  echo "<h2>Erro ao
inserir dados de execícios.</h2>";

                                                $sql = "UPDATE
saedd_protocol SET keep_day = '1' WHERE block = '1' AND user_id =
'$user_id' ";

                                                if(!$Obj-
>ExecQuery($sql))
                                                  echo "<h2>Erro ao
inserir dados de execícios.</h2>";
                                        }

                                        $Obj->CloseDB();
                                ?>


                                <table>

                                        <tr style="text-align: center;
font-size: 18px; font-weight: bold;"><td id="program_p1">Fichas</td><td
id="program_p2">Moedas</td><td id="program_p3">Medalhas</td><td
id="program_p4">Troféus</td><td id="program_p5">Dia</td><td
id="program_p6">Exercício</td></tr>
                                        <tr style="text-align:
center;"><td><img src='images/try.png' /></td><td><img
src='images/coin.png' style='width: 64px;' /></td><td><img
src='images/medal.png' /></td><td><img src='images/trophy.png'
/></td><td><img src='images/date.png' /></td><td><img
src='images/check.png' /></td></tr>
                                        <tr style="text-align: center;
font-size: 18px; font-weight: bold;"><td>x <span
id='try'>0</span></td><td>x <span id='coin'>0</span></td><td>x <span
id='medal'>0</span></td><td>x <span id='trophy'>0</span></td><td>x <span
id='day'>0</span></td><td><span id='exercise'>---</span></td></tr>
                                </table>

                                <div id='saida'></div><br/><br/>
```

```
                                <!--
                                <input type="button"
onclick="javascript:updateAwards(1);" value="+1 moeda" />
                                <input type="button" style="margin-left:
40px;" onclick="javascript:updateAwards(3);" value="+1 medalha" />
                                <input type="button" style="margin-left:
40px;" onclick="javascript:updateAwards(0);" value="+1 trofeu" />
                                <input type="button" style="margin-left:
40px;" onclick="javascript:updateAwards(2);" value="+1 tentativa
(Exercício difícil)" />
                                <input type="button" style="margin-left:
40px;"  onclick="javascript:updateAwards(4);" value="Mudar dia" />

                                <br/><br/>-->




                                <span style='display: none;'
id='program_status1'>Nada cansativo</span>
                                <span style='display: none;'
id='program_status2'>Pouco cansativo</span>
                                <span style='display: none;'
id='program_status3'>Cansativo</span>
                                <span style='display: none;'
id='program_status4'>Muito cansativo</span>
                                <span style='display: none;'
id='program_time'>Começando em: </span>


                                <div id='videoarea'></div><div
class="clear clearfix"></div><br/><br/>

                                <div id="rulerarea">
                                        <canvas id='game' width="720"
height="800"></canvas>
                                </div>
                                <b id="program_tipmarker">Clique em cima
da nota sugerida ou arraste o marcador no grau que corresponde ao seu
esforço para realizar o exercício. <b>É importante você avaliar
corretamente o seu grau de dificuldade, pois é através da sua avaliação
que o software vai tornar a sua progressão individualizada.</b><br/><br/>


                                <input type="hidden" id="vday" value=""
/>
                                <input type="hidden" id="vblock" value=""
/>
                                <input type="hidden" id="vposition"
value="" />
                                <input type="hidden" id="difficult"
value="" />
                                <input type="hidden" id="banned" value=""
/>


                        </div>




                        </div>
```

```
                </div>


        </div>
</div>
```

```php
<?php
        session_start();

        function getLanguage($str, $defaut = "") {

                $language = "pt_br";

                if(isset($_SESSION["language_code"]))
                        $language = $_SESSION["language_code"];
                else
                        $_SESSION["language_code"] = $language;

                if($language == "pt_br")
                        return $defaut;

                include("../languages/".$language.".php");

                return $translations[trim($str)];
        }


        function ChangeUserAwards($type, $position, $block, $user_id) {

             require_once('../MysqlDB.php');
             $Obj = new ScriptDB();
             $sql = "";

             if($position == "")
               $position = "last_position";
             else
               $position = "(CASE WHEN $position = (SELECT MAX(position)
FROM saedd_protocol WHERE user_id = '$user_id' AND block = $block) THEN 1
ELSE last_position+1 END)";

             if($type == 1)
               $sql = "UPDATE saedd_awards SET coins = coins+1, medals =
floor(coins/8), trophies = floor(coins/16), tries = tries+1,
last_position = $position WHERE user_id = '$user_id'";
             else if($type == 0)
               $sql = "UPDATE saedd_awards SET coins = coins+16, medals =
floor(coins/8), trophies = floor(coins/16), tries = tries+16 WHERE
user_id = '$user_id'";
             else if($type == 2)
               $sql = "UPDATE saedd_awards SET tries = tries+1,
last_position = $position WHERE user_id = '$user_id'";
             else if($type == 3)
               $sql = "UPDATE saedd_awards SET coins = coins+8, medals =
floor(coins/8), trophies = floor(coins/16), tries = tries+8 WHERE user_id
= '$user_id'";
             else if($type == 4)
               $sql = "UPDATE saedd_awards SET last_day = CURDATE() -
INTERVAL 1 DAY WHERE user_id = '$user_id'";
```

```
            if(!$Obj->ExecQuery($sql))
               echo "erro";

            if($type == 4)
              if(!$Obj->ExecQuery("UPDATE saedd_protocol SET
registration_date =  (CURDATE() - INTERVAL 1 DAY) WHERE
DATE(registration_date) = CURDATE() AND  user_id = '$user_id'"))
                echo "erro";

            return 1;

         }


         if(isset($_POST['action'])) {

            $user_id = $_SESSION['id'];

            $action = intval($_POST['action']);
            require_once('../MysqlDB.php');
            $Obj = new ScriptDB();
            $html = "";

            if($action == 1)
                 ChangeUserAwards(1,"",1, $user_id);
            else if($action == 0)
                 ChangeUserAwards(0,"",1, $user_id);
            else if($action == 2)
                 ChangeUserAwards(2,"",1, $user_id);
            else if($action == 3)
                 ChangeUserAwards(3,"",1, $user_id);
            else if($action == 4)
                 ChangeUserAwards(4,"",1, $user_id);

            else if($action == 5) {

                 if(isset($_POST["difficult"]) && $_POST["difficult"] !=
"") {

                     $hard =
floatval(str_replace(",",".",$_POST["difficult"]));
                     $day = intval($_POST["day"]);
                     $block = intval($_POST["block"]);
                     $position = intval($_POST["position"]);

                     $sql = "UPDATE saedd_protocol SET difficult =
'$hard', registration_date = NOW(), keep_day = keep_day-1, exec_count =
exec_count+1 WHERE user_id = '$user_id' AND position = '$position' AND
block = '$block';";

                     if($Obj->ExecQuery($sql)) {
                        if($hard <= 2.0)
                           ChangeUserAwards(1,$position,1, $user_id);
                        else
                           ChangeUserAwards(2,$position,1, $user_id);
                     }
                     else
                       echo "ERROR: ".$sql." ".$Obj->GetLastError();

                     if($hard >= 2.1 && $hard <= 7.0) {
                         $sql = "UPDATE saedd_protocol SET keep_day = 2
WHERE user_id = '$user_id' AND position = '$position' AND block =
'$block';";
                     }
```

```
                    else if($hard <= 2.0) {
                        $sql = "UPDATE saedd_protocol SET keep_day = 1
WHERE user_id = '$user_id' AND position = '$position' AND block =
$block+1;";
                    }
                    else if($hard >= 7.1 && $block != 1) {
                        $Obj->ExecQuery("UPDATE saedd_protocol SET
keep_day = 2 WHERE user_id = '$user_id' AND position = '$position' AND
block = $block-1;");
                        $sql = "UPDATE saedd_protocol SET keep_day = NULL
WHERE user_id = '$user_id' AND position = '$position' AND block =
'$block';";
                    }
                    else if($hard >= 7.1 && $block == 1) {
                        $sql = "UPDATE saedd_protocol SET keep_day = 2
WHERE user_id = '$user_id' AND position = '$position' AND block =
$block;";
                    }

                    if(!$Obj->ExecQuery($sql))
                         echo "ERROR 2: ".$sql." ".$Obj->GetLastError();

                }

                $langcode = $_SESSION["language_code"];

                $sql = "SELECT a.id, title, a.block, a.position,
a.number, difficult, evolution, description, video, exec_count, objetive
FROM saedd_protocol a, saedd_exercises WHERE language = '$langcode' AND
code = number AND user_id = '$user_id' AND position = (SELECT
last_position FROM saedd_awards WHERE user_id = a.user_id) AND keep_day
IS NOT NULL AND keep_day > 0 ORDER BY block, position LIMIT 1";
                $Obj->ExecQuery($sql);

                $count = $Obj->Get("RecordCount");

                if($count != 0) {
                   $id = $Obj->GetQueryValue(0, 0);
                   $block = $Obj->GetQueryValue(0, 2);
                   $position = $Obj->GetQueryValue(0, 3);
                   $title = $Obj->GetQueryValue(0, 1); /*$Obj-
>GetQueryValue(0, 4) . " - " .*/ ;
                   $hard = $Obj->GetQueryValue(0, 5);
                   $evo = $Obj->GetQueryValue(0, 6);
                   $exe = $Obj->GetQueryValue(0, 9);

                   $html .= "|".$block."|".$position."|";
                   $html .= "<h4><span>$title</span></h4>";
                   $html .= "<h4><span style='color:
#22CC33;'>$evo</span> - ".getLanguage("PROTOCOL_EXECUTION","Execução")."
$exe</h4>";

                   if($hard != "")
                     $html .=
"<b>".getLanguage("PROTOCOL_LASTTRY","Última tentativa
(Dificuldade):")."</b> $hard<br/>";

                    $html .= "<br/><b>".getLanguage("PROTOCOL_HOW","Como
executar: ")."</b> ".$Obj->GetQueryValue(0,
7)."<br/><b>".getLanguage("PROTOCOL_OBJECTIVE","Objetivo: ").":</b>
".$Obj->GetQueryValue(0, 10);
                    $html .= "<br/>";

                    $html .= "|".$Obj->GetQueryValue(0, 8);
                }
```

```
                else {
                    $html .= "|0|0|<br/><br/><br/><h4><span>Parabéns você
concluiu todos os exercícios!</span></h4><br/><h3><span>Retorne essa
semana para dar continuidade ao seu programa de exercícios</span></h3>|--
|--";
                }
           }



           $sql = "UPDATE saedd_awards SET days = (CASE WHEN last_day =
CURDATE() THEN days ELSE days+1 END), last_day = CURDATE() WHERE user_id
= '$user_id'";
            if(!$Obj->ExecQuery($sql))
              echo "Erro!";

           $Obj->ExecQuery("SELECT coins, medals,
CONCAT(trophies,'<br/>', (SELECT title FROM saedd_trophies WHERE trophies
>= min_trophies AND trophies <= max_trophies limit 1)), tries, days, CASE
WHEN (SELECT COUNT(*) FROM saedd_protocol WHERE DATE(registration_date) =
last_day AND user_id = saedd_awards.user_id) >= (SELECT MAX(position)
FROM saedd_protocol WHERE user_id = saedd_awards.user_id) THEN 1 ELSE 0
END FROM saedd_awards WHERE user_id = '$user_id'");
           $count = $Obj->Get("RecordCount");

           if($count == 1)
             echo utf8_encode($Obj->GetQueryValue(0, 0)."|".$Obj-
>GetQueryValue(0, 1)."|".$Obj->GetQueryValue(0, 2)."|".$Obj-
>GetQueryValue(0, 3)."|".$Obj->GetQueryValue(0, 4)."|".$Obj-
>GetQueryValue(0, 5).$html);
          else
             echo "Erro!";
          return;
        }
?>


<div  class="content_row row vc_row wpb_row vc_row-fluid default-style
fullwidth" style="position:relative;overflow:hidden;">
        <div class="rt-parallax-background" data-rt-parallax-
direction="1" data-rt-parallax-effect="horizontal" style="background-
image: url(images/portfolio_header.jpg);background-repeat:
repeat;background-size: cover;background-position: right
top;width:100%;height:100%;top:0;"></div>
        <div class="content_row_wrapper  default" style="padding-
top:25px;padding-bottom:15px;">
                <div class="vc_col-sm-12 wpb_column vc_column_container"
>
                        <div class="wpb_wrapper">
                                <div class="rt_heading_wrapper style-4">
                                        <h1 class="rt_heading  style-4"
>Simulação de Protocolo</h1>
                                </div>
                        </div>
                </div>
        </div>
</div>
```

```
<div  class="content_row row vc_row wpb_row vc_row-fluid alt-style-1
fullwidth border_grid fixed_heights" >
        <div class="content_row_wrapper  default" style="padding-
top:50px;padding-bottom:50px; min-height: 500px;">
                <div class="vc_col-sm-12 wpb_column vc_column_container"
>
                        <div class="wpb_wrapper">


                                <?php
                                        $user_id = 1;

                                        require_once('php/MysqlDB.php');
                                        $Obj = new ScriptDB();


                                        if(isset($_GET['start'])) {

                                                $Obj->ExecQuery("DELETE
FROM saedd_awards WHERE user_id = '$user_id'");
                                                $Obj->ExecQuery("DELETE
FROM saedd_protocol WHERE user_id = '$user_id'");
                                        }

                                        $Obj->ExecQuery("SELECT COUNT(*)
FROM saedd_awards WHERE user_id = '$user_id'");


                                        if(intval($Obj->GetQueryValue(0,
0)) == 0) {

                                                $sql = "INSERT INTO
saedd_awards(coins, medals, trophies, last_day, tries, user_id) VALUES(0,
0, 0, CURDATE(), 0, '$user_id')";

                                                if(!$Obj-
>ExecQuery($sql))
                                                  echo "<h2>Erro ao
inserir dados de recompensa.</h2>";
                                        }


                                        $Obj->ExecQuery("SELECT COUNT(*)
FROM saedd_protocol WHERE user_id = '$user_id'");


                                        if(intval($Obj->GetQueryValue(0,
0)) == 0) {

                                                $sql = "INSERT INTO
saedd_protocol(protocol, block, position, number, difficult, user_id,
registration_date) SELECT protocol, block, position, exercise, NULL,
'$user_id', NULL FROM saedd_blocks WHERE protocol = COALESCE((SELECT
protocol FROM saedd_users WHERE id = '$user_id'), NULL, 1)";

                                                if(!$Obj-
>ExecQuery($sql))
                                                  echo "<h2>Erro ao
inserir dados de execícios.</h2>";

                                                $sql = "UPDATE
saedd_protocol SET keep_day = '1' WHERE block = '1' AND user_id =
'$user_id' ";
```

```
                                                if(!$Obj-
>ExecQuery($sql))
                                                  echo "<h2>Erro ao
inserir dados de execícios.</h2>";
                                        }

                                        $Obj->CloseDB();
                                ?>


                                <table>
                                        <tr style="text-align: center;
font-size: 18px; font-weight:
bold;"><td>Fichas</td><td>Moedas</td><td>Medalhas</td><td>Troféus</td><td
>Dia</td><td>Exercício</td></tr>
                                        <tr style="text-align:
center;"><td><img src='images/try.png' /></td><td><img
src='images/coin.png' /></td><td><img src='images/medal.png'
/></td><td><img src='images/trophy.png' /></td><td><img
src='images/date.png' /></td><td><img src='images/check.png' /></td></tr>
                                        <tr style="text-align: center;
font-size: 18px; font-weight: bold;"><td>x <span
id='try'>0</span></td><td>x <span id='coin'>0</span></td><td>x <span
id='medal'>0</span></td><td>x <span id='trophy'>0</span></td><td>x <span
id='day'>0</span></td><td><span id='exercise'>---</span></td></tr>
                                </table>

                                <div id='saida'></div><br/><br/>

                                <input type="button"
onclick="javascript:updateAwards(1);" value="+1 moeda" />
                                <input type="button" style="margin-left:
40px;" onclick="javascript:updateAwards(3);" value="+1 medalha" />
                                <input type="button" style="margin-left:
40px;" onclick="javascript:updateAwards(0);" value="+1 trofeu" />
                                <input type="button" style="margin-left:
40px;" onclick="javascript:updateAwards(2);" value="+1 tentativa
(Exercício difícil)" />
                                <input type="button" style="margin-left:
40px;"  onclick="javascript:updateAwards(4);" value="Mudar dia" />

                                <br/><br/>

                                <div id='videoarea'></div><br/>
                                <div id="rulerarea"><canvas id='game'
width="720" height="800"></canvas></div>


                                <input type="hidden" id="vday" value=""
/>
                                <input type="hidden" id="vblock" value=""
/>
                                <input type="hidden" id="vposition"
value="" />
                                <input type="hidden" id="difficult"
value="" />
                                <input type="hidden" id="banned" value=""
/>
                        </div>
                </div>
        </div>
</div>
<?php
                        function base64_to_file($base64, $id)
                        {
```

```
                            $ifp =
fopen("../../files/3dfoot/".$id.".png", "wb");
                            $data = explode(',', $base64);
                            fwrite($ifp, base64_decode($data[1]));
                            fclose($ifp);
                        }

        if(isset($_POST['action']))
        {
                $action = $_POST['action'];

                if($action == "step1")
                {

        require_once('../MysqlDB.php');
                                                $Obj = new ScriptDB();

                                                $Obj->Set("TableName",
"saedd_occur_01");
                                                $Obj->Set("FieldsName",
"fr_q1|fr_q2|fr_q3|fr_q4|fl_q1|fl_q2|fl_q3|fl_q4|user_id|registration_dat
e");
                                                $Obj-
>BeforeInsert("registration_date","NOW()");

                                                $sql = $Obj-
>CreateInsert();

                                                $Obj->Connect();
                                                if($Obj->ExecQuery($sql))
                                                {
                                                        $sql = "INSERT
INTO saedd_quests(user_id,quest_datetime,quest)
VALUES('".$_POST['user_id']."',NOW(),'occurrence_1');";
                                                        $Obj-
>ExecQuery($sql);

                                                        echo "1";

                                                }
                                                else
                                                {
                                                          echo $Obj-
>GetLastError();
                                                }

                                                $Obj->CloseDB();


                }
                else if($action == "step2")
                {

        require_once('../MysqlDB.php');
                                                $Obj = new ScriptDB();

                                                $Obj->Set("TableName",
"saedd_occur_02");
                                                $Obj->Set("FieldsName",
"fr_q1|fr_q2|fr_q3|fr_q4|fr_q5|fr_q6|fl_q1|fl_q2|fl_q3|fl_q4|fl_q5|fl_q6|
user_id|registration_date");
                                                $Obj-
>BeforeInsert("registration_date","NOW()");
```

```
                                                $sql = $Obj-
>CreateInsert();

                                                $Obj->Connect();
                                                if($Obj->ExecQuery($sql))
                                                {
                                                        $sql = "INSERT
INTO saedd_quests(user_id,quest_datetime,quest)
VALUES('".$_POST['user_id']."',NOW(),'occurrence_2');";
                                                        $Obj-
>ExecQuery($sql);

                                                        echo "1";

                                                }
                                                else
                                                {
                                                          echo $Obj-
>GetLastError();
                                                }

                                                $Obj->CloseDB();


                }
                else if($action == "step3")
                {

        require_once('../MysqlDB.php');
                                                $Obj = new ScriptDB();

                                                $Obj->Set("TableName",
"saedd_occur_03");
                                                $Obj->Set("FieldsName",
"fr_q1|fr_q2|fr_q3|fr_q4|fr_q5|fr_q6|fr_q7|fl_q1|fl_q2|fl_q3|fl_q4|fl_q5|
fl_q6|fl_q7|user_id|registration_date");
                                                $Obj-
>BeforeInsert("registration_date","NOW()");

                                                $sql = $Obj-
>CreateInsert();

                                                $Obj->Connect();
                                                if($Obj->ExecQuery($sql))
                                                {
                                                        $sql = "INSERT
INTO saedd_quests(user_id,quest_datetime,quest)
VALUES('".$_POST['user_id']."',NOW(),'occurrence_3');";
                                                        $Obj-
>ExecQuery($sql);
                                                        $sql = "UPDATE
saedd_users SET stars = (stars+1) WHERE id =
'".trim($_POST['user_id'])."'";
                                                        $Obj-
>ExecQuery($sql);
                                                        echo "1";

                                                }
                                                else
                                                {
                                                          echo $Obj-
>GetLastError();
                                                }

                                                $Obj->CloseDB();
```

```
                }
                else if($action == "step4")
                {
                        base64_to_file($_POST['data'],
$_POST['user_id']."_scene_".$_POST['pos']);
                        echo "1";
                }
                else if($action == "conclude")
                {

        require_once('../MysqlDB.php');
                                                        $Obj = new ScriptDB();
                                                        $Obj->Connect();

                                                        $sql = "INSERT INTO
saedd_quests(user_id,quest_datetime,quest)
VALUES('".$_POST['user_id']."',NOW(),'occurrence_4');";
                                                        $Obj->ExecQuery($sql);

                                                        $sql = "UPDATE
saedd_users SET stars = (stars+1) WHERE id =
'".trim($_POST['user_id'])."'";
                                                        $Obj->ExecQuery($sql);

                                                        echo "1";

                                                        $Obj->CloseDB();
                }
                return;
        }
?>

<?php include("php/pages/back.php"); ?>

<link rel='stylesheet' href='css/myform.css' type='text/css' media='all'
/>

<style type="text/css">
#myform input[type="text"],
#myform input[type="date"],
#myform input[type="datetime"],
#myform input[type="email"],
#myform input[type="number"],
#myform input[type="search"],
#myform input[type="time"],
#myform input[type="url"],
#myform input[type="password"],
#myform textarea,
#myform select {

    border-top: 1px solid #f0f0f0;
    border-left: 1px solid #f0f0f0;
    border-bottom: 1px solid #FAFAFA;
    border-right: 1px solid #FAFAFA;
    background: #e0e0e0;
}

.default-style input[type="button"]:hover {background-color: #FC9D0D;}
.default-style input[type="button"] {background-color: #428bca; font-
weight: bold;}

.default-style input[type="button"]:hover.extra  {background-color:
#BBB;}
```

```
.default-style input[type="button"].extra  {background-color: #888; font-
weight: bold;}

h4 span {color: #428BCA;}

</style>


<script type="text/javascript">


        function ValidateStep1()
        {
           var leftFoot = ["0","0","0","0"];
           var rightFoot = ["0","0","0","0"];

           jQuery("#msg").html("");

           for(i=1; i<=3; i++)
           {
              pos = i-1;

              if(jQuery("#oc_"+i+"_fl").is(':checked') == true)
              {
                 leftFoot[pos] = "1";

                 if(jQuery("#fl_"+i+"_d1").is(':checked') == true)
                   leftFoot[pos] += "1";
                 else
                   leftFoot[pos] += "0";

                 if(jQuery("#fl_"+i+"_d2").is(':checked') == true)
                   leftFoot[pos]+= "1";
                 else
                   leftFoot[pos] += "0";

                 if(jQuery("#fl_"+i+"_d3").is(':checked') == true)
                   leftFoot[pos]+= "1";
                 else
                   leftFoot[pos] += "0";

                 if(jQuery("#fl_"+i+"_d4").is(':checked') == true)
                   leftFoot[pos] += "1";
                 else
                   leftFoot[pos] += "0";

                 if(jQuery("#fl_"+i+"_d5").is(':checked') == true)
                   leftFoot[pos] += "1";
                 else
                   leftFoot[pos] += "0";

                 if(leftFoot[pos] == "100000")
                 {
                    jQuery("#msg").html("Informe os dedos do pé esquerdo
referente a pergunta "+i);
                    return;
                 }

              }

              if(jQuery("#oc_"+i+"_fr").is(':checked') == true)
              {
                 rightFoot[pos] = "1";

                 if(jQuery("#fr_"+i+"_d1").is(':checked') == true)
```

```
                      rightFoot[pos] += "1";
                    else
                      rightFoot[pos] += "0";

                    if(jQuery("#fr_"+i+"_d2").is(':checked') == true)
                      rightFoot[pos]+= "1";
                    else
                      rightFoot[pos] += "0";

                    if(jQuery("#fr_"+i+"_d3").is(':checked') == true)
                      rightFoot[pos]+= "1";
                    else
                      rightFoot[pos] += "0";

                    if(jQuery("#fr_"+i+"_d4").is(':checked') == true)
                      rightFoot[pos] += "1";
                    else
                      rightFoot[pos] += "0";

                    if(jQuery("#fr_"+i+"_d5").is(':checked') == true)
                      rightFoot[pos] += "1";
                    else
                      rightFoot[pos] += "0";

                    if(rightFoot[pos] == "100000")
                    {
                       jQuery("#msg").html("Informe os dedos do pé direto
referente a pergunta "+i);
                       return;
                    }

                 }

              }

              if(jQuery("#oc_4_fl").is(':checked') == true)
                 leftFoot[3] = "1";

              if(jQuery("#oc_4_fr").is(':checked') == true)
                 rightFoot[3] = "1";



              jQuery.ajax({
                   type: "POST",
                   url: "php/pages/occurrence.php",
                   data: {fr_q1 : rightFoot[0], fr_q2 : rightFoot[1], fr_q3
: rightFoot[2], fr_q4 : rightFoot[3], fl_q1 : leftFoot[0], fl_q2 :
leftFoot[1], fl_q3 : leftFoot[2], fl_q4 : leftFoot[3], user_id : <?php
echo $_SESSION['id']; ?>,  action : "step1"}
             })
            .done(function(html) {
                   if(html == "1")
                   {
                           var result = "<h2>Questionário de Ocorrências 1/4
respondido com sucesso!</h2><br/>";
                           result += "<p>Agradecemos a paciência! Continue o
cadastro para que possamos conhecer mais do seu pé.</p>";
                           result += "<br/><br/><div class='submit-
button'><input class='button submit' onclick=\"javascript:window.location
= 'index.php?page=occurrence';\" value='Clique aqui para continuar...'
type='button'><span class='loading'></span></div>";


                           jQuery("#etapa1").html(result);
```

```
                        jQuery("html, body").animate({ scrollTop: 0 },
"slow");
                }
                else
                  jQuery("#msg").html("Erro: "+html);
            });
        }


        function ValidateStep2()
        {
           var leftFoot = ["0","0","0","0","0","0"];
           var rightFoot = ["0","0","0","0","0","0"];

           jQuery("#msg2").html("");

           for(i=5; i<=10; i++)
           {
              pos = i-1;

              if(jQuery("#oc_"+i+"_fl").is(':checked') == true)
              {
                 leftFoot[pos] = "1";

                 if(jQuery("#fl_"+i+"_d1").is(':checked') == true)
                   leftFoot[pos] += "1";
                 else
                   leftFoot[pos] += "0";

                 if(jQuery("#fl_"+i+"_d2").is(':checked') == true)
                   leftFoot[pos]+= "1";
                 else
                   leftFoot[pos] += "0";

                 if(jQuery("#fl_"+i+"_d3").is(':checked') == true)
                   leftFoot[pos]+= "1";
                 else
                   leftFoot[pos] += "0";

                 if(jQuery("#fl_"+i+"_d4").is(':checked') == true)
                   leftFoot[pos] += "1";
                 else
                   leftFoot[pos] += "0";

                 if(jQuery("#fl_"+i+"_d5").is(':checked') == true)
                   leftFoot[pos] += "1";
                 else
                   leftFoot[pos] += "0";

                 if(jQuery("#fl_"+i+"_d6").is(':checked') == true)
                   leftFoot[pos] += "1";
                 else
                   leftFoot[pos] += "0";

                 if(jQuery("#fl_"+i+"_d7").is(':checked') == true)
                   leftFoot[pos] += "1";
                 else
                   leftFoot[pos] += "0";

                 if(jQuery("#fl_"+i+"_d8").is(':checked') == true)
                   leftFoot[pos] += "1";
                 else
                   leftFoot[pos] += "0";
```

```
                    if(leftFoot[pos] == "100000000")
                    {
                       jQuery("#msg2").html("Informe os dedos ou região do
pé esquerdo referente a pergunta "+i);
                       return;
                    }

                 }

                 if(jQuery("#oc_"+i+"_fr").is(':checked') == true)
                 {
                    rightFoot[pos] = "1";

                    if(jQuery("#fr_"+i+"_d1").is(':checked') == true)
                      rightFoot[pos] += "1";
                    else
                      rightFoot[pos] += "0";

                    if(jQuery("#fr_"+i+"_d2").is(':checked') == true)
                      rightFoot[pos]+= "1";
                    else
                      rightFoot[pos] += "0";

                    if(jQuery("#fr_"+i+"_d3").is(':checked') == true)
                      rightFoot[pos]+= "1";
                    else
                      rightFoot[pos] += "0";

                    if(jQuery("#fr_"+i+"_d4").is(':checked') == true)
                      rightFoot[pos] += "1";
                    else
                      rightFoot[pos] += "0";

                    if(jQuery("#fr_"+i+"_d5").is(':checked') == true)
                      rightFoot[pos] += "1";
                    else
                      rightFoot[pos] += "0";

                    if(jQuery("#fr_"+i+"_d6").is(':checked') == true)
                      rightFoot[pos] += "1";
                    else
                      rightFoot[pos] += "0";

                    if(jQuery("#fr_"+i+"_d7").is(':checked') == true)
                      rightFoot[pos] += "1";
                    else
                      rightFoot[pos] += "0";

                    if(jQuery("#fr_"+i+"_d8").is(':checked') == true)
                      rightFoot[pos] += "1";
                    else
                      rightFoot[pos] += "0";

                    if(rightFoot[pos] == "100000000")
                    {
                       jQuery("#msg2").html("Informe os dedos ou região do
pé direto referente a pergunta "+i);
                       return;
                    }

                 }

              }

              jQuery.ajax({
```

```
                type: "POST",
                url: "php/pages/occurrence.php",
                data: {fr_q1 : rightFoot[0], fr_q2 : rightFoot[1], fr_q3
: rightFoot[2], fr_q4 : rightFoot[3], fr_q5 : rightFoot[4], fr_q6 :
rightFoot[5], fl_q1 : leftFoot[0], fl_q2 : leftFoot[1], fl_q3 :
leftFoot[2], fl_q4 : leftFoot[3], fl_q5 : leftFoot[4], fl_q6 :
leftFoot[5], user_id : <?php echo $_SESSION['id']; ?>,  action : "step2"}
          })
         .done(function(html) {
                if(html == "1")
                {
                        var result = "<h2>Questionário de Ocorrências 2/4
respondido com sucesso!</h2><br/>";
                        result += "<p>Agradecemos a paciência! Continue o
cadastro para que possamos conhecer mais do seu pé.</p>";
                        result += "<br/><br/><div class='submit-
button'><input class='button submit' onclick=\"javascript:window.location
= 'index.php?page=occurrence';\" value='Clique aqui para continuar...'
type='button'><span class='loading'></span></div>";


                        jQuery("#etapa2").html(result);
                        jQuery("html, body").animate({ scrollTop: 0 },
"slow");
                }
                else
                  jQuery("#msg2").html("Erro: "+html);
          });
     }




        function ValidateStep3()
        {
           var leftFoot = ["0","0","0","0","0","0","0"];
           var rightFoot = ["0","0","0","0","0","0","0"];

           var occNum = 0;

           jQuery("#msg2").html("");

           for(i=11; i<=16; i++)
           {
              pos = i-1;

              if(jQuery("#oc_"+i+"_fl").is(':checked') == true)
              {
                 occNum++;
                 leftFoot[pos] = "1";

                 if(jQuery("#fl_"+i+"_d1").is(':checked') == true)
                   leftFoot[pos] += "1";
                 else
                   leftFoot[pos] += "0";

                 if(jQuery("#fl_"+i+"_d2").is(':checked') == true)
                   leftFoot[pos]+= "1";
                 else
                   leftFoot[pos] += "0";

                 if(jQuery("#fl_"+i+"_d3").is(':checked') == true)
                   leftFoot[pos]+= "1";
                 else
                   leftFoot[pos] += "0";
```

```
                    if(jQuery("#fl_"+i+"_d4").is(':checked') == true)
                      leftFoot[pos] += "1";
                    else
                      leftFoot[pos] += "0";

                    if(jQuery("#fl_"+i+"_d5").is(':checked') == true)
                      leftFoot[pos] += "1";
                    else
                      leftFoot[pos] += "0";

                    if(jQuery("#fl_"+i+"_d6").is(':checked') == true)
                      leftFoot[pos] += "1";
                    else
                      leftFoot[pos] += "0";

                    if(jQuery("#fl_"+i+"_d7").is(':checked') == true)
                      leftFoot[pos] += "1";
                    else
                      leftFoot[pos] += "0";

                    if(jQuery("#fl_"+i+"_d8").is(':checked') == true)
                      leftFoot[pos] += "1";
                    else
                      leftFoot[pos] += "0";

                    if(leftFoot[pos] == "100000000")
                    {
                       jQuery("#msg3").html("Informe os dedos ou região do
pé esquerdo referente a pergunta "+i);
                       return;
                    }

                 }

                 if(jQuery("#oc_"+i+"_fr").is(':checked') == true)
                 {
                    occNum++;
                    rightFoot[pos] = "1";

                    if(jQuery("#fr_"+i+"_d1").is(':checked') == true)
                      rightFoot[pos] += "1";
                    else
                      rightFoot[pos] += "0";

                    if(jQuery("#fr_"+i+"_d2").is(':checked') == true)
                      rightFoot[pos]+= "1";
                    else
                      rightFoot[pos] += "0";

                    if(jQuery("#fr_"+i+"_d3").is(':checked') == true)
                      rightFoot[pos]+= "1";
                    else
                      rightFoot[pos] += "0";

                    if(jQuery("#fr_"+i+"_d4").is(':checked') == true)
                      rightFoot[pos] += "1";
                    else
                      rightFoot[pos] += "0";

                    if(jQuery("#fr_"+i+"_d5").is(':checked') == true)
                      rightFoot[pos] += "1";
                    else
                      rightFoot[pos] += "0";
```

```
                if(jQuery("#fr_"+i+"_d6").is(':checked') == true)
                  rightFoot[pos] += "1";
                else
                  rightFoot[pos] += "0";

                if(jQuery("#fr_"+i+"_d7").is(':checked') == true)
                  rightFoot[pos] += "1";
                else
                  rightFoot[pos] += "0";

                if(jQuery("#fr_"+i+"_d8").is(':checked') == true)
                  rightFoot[pos] += "1";
                else
                  rightFoot[pos] += "0";

                if(rightFoot[pos] == "100000000")
                {
                   jQuery("#msg3").html("Informe os dedos ou região do
pé direto referente a pergunta "+i);
                   return;
                }

             }

          }

          jQuery.ajax({
               type: "POST",
               url: "php/pages/occurrence.php",
               data: {fr_q1 : rightFoot[0], fr_q2 : rightFoot[1], fr_q3
: rightFoot[2], fr_q4 : rightFoot[3], fr_q5 : rightFoot[4], fr_q6 :
rightFoot[5], fr_q7 : rightFoot[6], fl_q1 : leftFoot[0], fl_q2 :
leftFoot[1], fl_q3 : leftFoot[2], fl_q4 : leftFoot[3], fl_q5 :
leftFoot[4], fl_q6 : leftFoot[5], fl_q7 : leftFoot[6], user_id : <?php
echo $_SESSION['id']; ?>,  action : "step3"}
          })
        .done(function(html) {
               if(html == "1")
               {
                      var result = "<h2>Questionário de Ocorrências 3/4
respondido com sucesso!</h2><br/>";

                      if(occNum > 0)
                        result += "<p>Agradecemos a paciência! <b
style='#color: #cc2233;'>Infelizmente você não está apto a realizar os
exercícios de forma autônoma, pois as ocorrências relatadas requerem
cuidados profissionais.</b> Mostre essa ferramenta ao seu médido e
questione-o sobre como deverá proceder o uso da mesma. Agradecemos a sua
parcipação e esperamos em breve tê-lo de volta ao SoPeD.<br/><br/>Para
finalizar, Gostaríamos de pedir que fizesse um modelo 3D das ocorrências
em seu pé na próxima etapa. É fácil e divertido!</p>";
                      else
                        result += "<p>Agradecemos a paciência!
Aparentemente, você está apto a utilizar a ferramenta, entretanto, a
opnião profissional é muito importante. Consulte seu médico para mais
detalhes.<br/><br/>Para finalizar, Gostaríamos de pedir que fizesse um
modelo 3D das ocorrências em seu pé na próxima etapa. É fácil e
divertido!</p>";

                      result += "<br/><br/><div class='submit-
button'><input class='button submit' onclick=\"javascript:window.location
= 'index.php?page=occurrence';\" value='Clique aqui para continuar...'
type='button'><span class='loading'></span></div>";
```

```
                        jQuery("#etapa3").html(result);
                        jQuery("html, body").animate({ scrollTop: 0 },
"slow");
                }
                else
                  jQuery("#msg3").html("Erro: "+html);
          });
     }



        function ValidateStep4()
        {
           for(i=0; i<13; i++)
           {
                if(sceneFrame[i] != "")
                {
                         jQuery.ajax({
                                type: "POST",
                                url: "php/pages/occurrence.php",
                                data: {data : sceneFrame[i], pos : i,
user_id : <?php echo $_SESSION['id']; ?>,  action : "step4"}
                          })
                         .done(function(html) {

                          });
                }
           }

           jQuery.ajax({
                type: "POST",
                url: "php/pages/occurrence.php",
                data: {user_id : <?php echo $_SESSION['id']; ?>,  action
: "conclude"}
          })
         .done(function(html) {
                if(html == "1")
                {
                          var result = "<h2>Questionário de Ocorrências
respondido com sucesso!</h2><br/>";
                          result += "<p>Parabéns! Seu questionário de
ocorrências está completo! Agora você poderá partir para os exercícios,
caso não tenhado obtido indicação contrária na ferramenta.</p>";
                          result += "<br/><br/><div class='submit-
button'><input class='button submit' onclick=\"javascript:window.location
= 'index.php?page=user_start';\" value='Concluir questionário'
type='button'><span class='loading'></span></div>";
                          result += "<br/><br/><h2>Obrigado por responder
todas as etapas!</h2><p>Você está pronto para avançar no SoPeD. Por
concluir essa etapa você ganhou uma estrela! <h2><img
src='images/star.png' /> x 1</h2><br/><b style='color: #428BCA;'>As
estrelas liberam ferramentas no software que auxiliam no cuidado dos seus
pés. Quanto mais estrelas, melhor a saúde do seu pé!</b></p>";

                          jQuery("#etapa4").html(result);
                          jQuery("html, body").animate({ scrollTop: 0 },
"slow");
                }
                else
                  jQuery("#msg4").html("Erro: "+html);
          });

     }
```

```
</script>

<?php
                        require_once('php/MysqlDB.php');
                        $id = $_SESSION['id'];

                        $Obj = new ScriptDB();
                        $Obj->Connect();
                        $Obj->ExecQuery("SELECT (SELECT COUNT(*) FROM
saedd_occur_01 WHERE user_id = '$id'), (SELECT COUNT(*) FROM
saedd_occur_02 WHERE user_id = '$id'), (SELECT COUNT(*) FROM
saedd_occur_03 WHERE user_id = '$id'), (SELECT COUNT(*) FROM saedd_quests
WHERE user_id = '$id' AND quest = 'occurrence_4')");

                        $oc1 = $Obj->GetQueryValue(0, 0);
                        $oc2 = $Obj->GetQueryValue(0, 1);
                        $oc3 = $Obj->GetQueryValue(0, 2);
                        $oc4 = $Obj->GetQueryValue(0, 3);

?>



        <script type='text/javascript' src='js/pixi.min.js'></script>
        <script type='text/javascript' src='js/main.js'></script>


                                <?php

                                        $occorrency = array(
                                                                "Unha solta",
"A unha de um dedo do pé pode acidentalmente se soltar se for atingida
por alguma coisa dura, como derrubar um objeto pesado sobre ela. Algumas
vezes, ela não se solta completamente de uma vez, mas vagarosamente se
solta do dedo. A unha crescerá novamente, mas até lá você precisa cuidar
do dedo sem unha para que ele não se infecte ou machuque ainda mais.",
"unha.png", "Você tem alguma unha solta nos pés? Em qual? Em qual dedo?",
                                                                "Dedos em
martelo", "É uma deformidade do pé em que os dedos estão dobrados nas
articulações mais distantes das unhas e esticados nas articulações mais
próximas das unhas (então ela fica voltada para cima).",
"dedo_martelo.png", "Você tem dedos em martelo? Em qual pé?",
                                                                "Dedos em
garra", "É uma deformidade do pé em que os dedos estão dobrados por
inteiro, ficando as unhas voltadas para frente.", "garras.png", "Você tem
dedos em garras? Em qual pé?",
                                                                "Joanete -
Hálux Valgo", "É uma deformidade do pé, mais conhecida como joanete, em
que o dedão (hálux) encontra-se desviado e vai ao encontro aos outros
dedos menores.", "halux.png", "Você tem Joanente (hálux Valgo)? Em qual
pé?",


                                                                "Dor",
"Incômodo, sem motivo físico aparente, que provoca a sensação de mal
estar, mesmo sem contato física", "dor.png", "Você sente dor nos pés? Em
qual? Onde é a dor?",
                                                                "Calos", "São
sinais que nos dizem que o pé está tendo pontos de pressão de uma forma
desigual. O corpo reage a esses pontos de maior pressão, como uma forma
de defesa, produzindo mais queratina, uma substância bastante presente na
planta (sola) do pé, mas que, quando aumentada, deixa a pele mais grossa
nesses locais.", "calo.png", "Você tem calos em algum dos pés? Em qual?
Onde estão os calos?",
```

"Bolha","É uma bolsa de água localizada sobre a pele. Ocorre um aumento do espaço entre as camadas da pele, gerado pelo deslocamento entre elas e geralmente preenchido com líquido incolor. Esse deslocamento ocorre muitas vezes por atrito excessivo, como um sapato muito apertado ou muito largo.", "bolha.png", "Você tem bolhas em algum dos pés? Em qual? Onde estão as bolhas?",

"Rachaduras", "São fissuras (pequenos cortes) nos pés, geralmente com a sensação de estarem secos. Ocorrem pelo aumento de espessura da pele e a consequente perda de elasticidade, podendo inclusive, ser bastante dolorosas e causar sangramentos. Elas surgem na maioria das vezes nos calcanhares, mas podem surgir em outras áreas.", "rachadura.png", "Você tem rachaduras em algum dos pés? Em qual? Onde estão as rachaduras?",

"Micoses", "São infecções causadas por fungos que, nos pés, pode afetar a pele (principalmente entre os dedos) e as unhas. Na pele são percebidas manchas brancas ou vermelhas, além de pequenas vesículas (bolhas cheias de líquido) e prurido (coceira). Quando descamam, podem gerar fissuras dolorosas. Nas unhas, as manifestações podem ser manchas brancas na superfície ou embaixo da unha e alterações da coloração, que vão desde branco-amarelado até o acastanhado ou preto.", "micose.png", "Você tem micose em algum dos pés? Em qual? Onde estão as micoses?",

"Olho de Peixe", "O Olho de Peixe é o nome popular de uma enfermidade que tem a aparência de um calo e origina-se de uma verruga. É um 'ferimento' profundo que aponta apenas uma pontinha preta para fora da pele, mas na verdade está bem profundo.", "olho_peixe.png", "Você tem Olho de Peixe nos pés? Em qual? Onde?",

"Pús", "É uma secreção (líquido seroso) de coloração amarelada ou amarelo-esverdeada, geralmente malcheirosa. Ela contém restos de células, proteínas e bactérias envolvidas num processo infeccioso. Procure atendimento médico. Pode representar uma condição grave e que inspira cuidados.", "pus.png", "Há pús em seus pés? Em qual? Onde está o pús?",

"Ferida aberta", "É qualquer interrupção da integridade e continuidade da pele. A pele é uma importante barreira protetora contra microrganismos causadores de doenças. Por isso quando há uma ferida abeta, o risco de infecções aumenta.", "ferida.png", "Você tem feridas em algum dos pés? Em qual? Onde estão as feridas?",

"Saída Líquido", "Se houver saída de líquido de qualquer cor (vermelha, amarela ou transparente), pode significar uma infecção. Procure atendimento médico. Pode representar uma condição grave e que inspira cuidados.", "liquido.png", "Há saída de líquido nos seus pés? Em qual? Onde estão as saídas de líquido?",

"Sangramento", "Se houver saída de sangue em algum local do pé, procure atendimento médico. Pode representar uma condição grave e que inspira cuidados.", "sangramento.png", "Há sangramento nos seus pés? Em qual? Onde está o sangramento?",

"Pé com coloração preta", "O pé de coloração preta indica que já houve morte das células do tecido por falta de oxigenação. Procure atendimento médico. Pode representar uma condição grave e que inspira cuidados.", "preto.png", "Há áreas pretas no seu pé? Em qual? Onde está a coloração preta?",

"Pé com coloração vermelha", "A vermelhidão (ou rubor) é um dos primeiros sinais de inflamação. Portanto, se presente, o pé está provavelmente na fase aguda de um processo inflamatório, decorrente de alguma agressão ao tecido (seja por infecção ou não). Procure atendimento médico. Pode

```
representar uma condição grave e que inspira cuidados.", "vermelho.png",
"Há áreas vermelhas no seu pé? Em qual? Onde está a coloração vermelha?",
                                                                "Pé com
coloração branca", "Juntamente com sensação de queda da temperatura, o pé
de coloração mais branca que o normal indica diminuição do fluxo
sanguíneo. Procure atendimento médico. É uma situação de emergência, pois
pode haver obstrução de alguma importante artéria.", "branco.png", "Há
coloração branca no seu pé? Em qual? Onde está a coloração branca?"


                                        );

                                ?>


<div  class="content_row default-style no-composer overlap fullwidth">
        <div class="content_row_wrapper clearfix ">
                <div class="col col-sm-12 col-xs-12 " style='min-height:
800px;'>
                        <div class="single post-1872 staff type-staff
status-publish has-post-thumbnail hentry" id="person-1872">





                        <div id='etapa4' style='display: <?php if($oc4 ==
0) echo "inline"; else echo "none"; ?>; height: 800px;'>

                                <h2 id="occurrence_title">Questionário
sobre ocorrências</h2><br/><br/>
                                <b id="occurrence_tip1">Por favor, ajude-
nos a ver o seu pé de forma tridimencional ou 3D.</b>
                                <ul>
                                        <li id="occurrence_i1">Selecione
as ocorrências na lista de botões abaixo e clique no local onde ela
ocorre dentro do pé 3D</li>
                                        <li id="occurrence_i2">Utilize os
botões em seta para girar o modelo 3D e encontrar a melhor vista para a
ocorrência</li>
                                        <li id="occurrence_i3">Para
excluir uma ocorrência, clique exatamente sobre ela.</li>
                                </ul>
                                <hr/>
                                <b  style='color: #428BCA;'
id="occurrence_tip2">Selecione uma ocorrência abaixo e clique na posição
onde ela ocorre.</b><br/><br/>

        <label for='op1' style='margin-right: 15px;' data-
toggle="tooltip" title="Dor no pé"><input type='radio' checked
value='Dor' name='opadd' id='op1' /><span
id='occurrence_s1'>Dor</span></label>
        <label for='op2' style='margin-right: 15px;' data-
toggle="tooltip" title="São sinais que nos dizem que o pé está tendo
pontos de pressão de uma forma desigual. O corpo reage a esses pontos de
maior pressão, como uma forma de defesa, produzindo mais queratina, uma
substância bastante presente na planta (sola) do pé, mas que, quando
aumentada, deixa a pele mais grossa nesses locais."><input type='radio'
value='Calo' name='opadd' id='op2' /><span
id='occurrence_s2'>Calo</span></span></label>
        <label for='op3' style='margin-right: 15px;' data-
toggle="tooltip" title="É UMA BOLSA DE ÁGUA LOCALIZADA SOBRE A PELE.
Ocorre um aumento do espaço entre as camadas da pele, gerado pelo
deslocamento entre elas e geralmente preenchido com líquido incolor. Esse
deslocamento ocorre muitas vezes por atrito excessivo (por sapato muito
```

```
apertado ou muito largo, por exemplo)."><input type='radio' value='Bolha'
name='opadd' id='op3' /><span id='occurrence_s3'>Bolha</span></label>
        <label for='op4' style='margin-right: 15px;' data-
toggle="tooltip" title="São fissuras (pequenos cortes) nos pés,
geralmente com a sensação de estarem secos. Ocorrem pelo aumento de
espessura da pele e a consequente perda de elasticidade, podendo
inclusive, ser bastante dolorosas e causar sangramentos. Elas surgem na
maioria das vezes nos calcanhares, mas podem surgir em outras
áreas."><input type='radio' value='Rachadura' name='opadd' id='op4'
/><span id='occurrence_s4'>Rachadura</span></label>
        <label for='op5' style='margin-right: 15px;' data-
toggle="tooltip" title="São infecções causadas por fungos que, nos pés,
pode afetar a pele (principalmente entre os dedos) e as unhas. Na pele
são percebidas manchas brancas ou vermelhas, além de pequenas vesículas
(bolhas cheias de líquido) e prurido (coceira). Quando descamam, podem
gerar fissuras dolorosas. Nas unhas, as manifestações podem ser manchas
brancas na superfície ou embaixo da unha e alterações da coloração (desde
branco-amarelado, ao acastanhado ou preto."><input type='radio'
value='Micose' name='opadd' id='op5' /><span
id='occurrence_s5'>Micose</span></label>
        <label for='op6' style='margin-right: 15px;' data-
toggle="tooltip" title="O Olho de Peixe é o nome popular de uma
enfermidade que tem a aparência de um calo e origina-se de uma verruga. É
um ferimento profundo que aponta apenas uma pontinha preta para fora da
pele, mas na verdade está bem profundo."><input type='radio' value='Olho
de peixe' name='opadd' id='op6' /><span id='occurrence_s6'>Olho de
peixe</span></label><br/><br/>
        <label for='op7' style='margin-right: 15px;' data-
toggle="tooltip" title="A unha de um dedo do pé pode acidentalmente se
soltar se for atingida por alguma coisa dura, como derrubar um objeto
pesado sobre ela. Algumas vezes, ela não se solta completamente de uma
vez, mas vagarosamente se solta do dedo. A unha crescerá novamente, mas
até lá você precisa cuidar do dedo sem unha para que ele não se infecte
ou machuque ainda mais."><input type='radio' value='Unha solta'
name='opadd' id='op7' /><span id='occurrence_s7'>Unha
solta</span></label>
        <label for='op8' style='margin-right: 15px;' data-
toggle="tooltip" title="É uma deformidade do pé em que os dedos estão
dobrados nas articulações mais distantes das unhas e esticados nas
articulações mais próximas das unhas (então ela fica voltada para
cima)."><input type='radio' value='Dedo em martelo' name='opadd' id='op8'
/><span id='occurrence_s8'>Dedos em martelo</span></label>
        <label for='op9' style='margin-right: 15px;' data-
toggle="tooltip" title="É uma deformidade do pé em que os dedos estão
dobrados por inteiro, ficando as unhas voltadas para frente."><input
type='radio' value='Dedos em garra' name='opadd' id='op9' /><span
id='occurrence_s9'>Dedos em garra</span></label>
        <label for='op10' style='margin-right: 15px;' data-
toggle="tooltip" title="É uma deformidade do pé, mais conhecida como
joanete, em que o dedão (hálux) encontra-se desviado e vai ao encontro
aos outros dedos menores."><input type='radio' value='Joanete'
name='opadd' id='op10' /><span
id='occurrence_s10'>Joanete</span></label><br/><br/>
        <label for='op11' style='margin-right: 15px;' data-
toggle="tooltip" title="É uma secreção (líquido seroso) de coloração
amarelada ou amarelo-esverdeada, geralmente malcheirosa. Ela contém
restos de células, proteínas e bactérias envolvidas num processo
infeccioso. Procure atendimento médico. Pode representar uma condição
grave e que inspira cuidados"><input type='radio' value='Pus'
name='opadd' id='op11' /><span id='occurrence_s11'>Pús</span></label>
        <label for='op12' style='margin-right: 15px;' data-
toggle="tooltip" title="É qualquer interrupção da integridade e
continuidade da pele. A pele é uma importante barreira protetora contra
microrganismos causadores de doenças. Por isso quando há uma ferida
```

```
abeta, o risco de infecções aumenta."><input type='radio' value='Ferida' 
name='opadd' id='op12' /><span id='occurrence_s12'>Ferida</span></label>
        <label for='op13' style='margin-right: 15px;' data-
toggle="tooltip" title="Se houver saída de líquido de qualquer cor 
(vermelha, amarela ou transparente), pode significar uma infecção. 
Procure atendimento médico. Pode representar uma condição grave e que 
inspira cuidados."><input type='radio' value='Líquido' name='opadd' 
id='op13' /><span id='occurrence_s13'>Líquido</span></label>
        <label for='op14' style='margin-right: 15px;' data-
toggle="tooltip" title="Se houver saída de sangue em algum local do pé, 
procure atendimento médico. Pode representar uma condição grave e que 
inspira cuidados."><input type='radio' value='Sangramento' name='opadd' 
id='op14' /><span id='occurrence_s14'>Sangramento</span></label>
        <label for='op15' style='margin-right: 15px;' data-
toggle="tooltip" title="O pé de coloração preta indica que já houve morte 
das células do tecido por falta de oxigenação (necrose). Procure 
atendimento médico. Pode representar uma condição grave e que inspira 
cuidados. "><input type='radio' value='Cor Preta' name='opadd' id='op15' 
/><span id='occurrence_s14'>Pele Preta</span></label>
        <label for='op16' style='margin-right: 15px;' data-
toggle="tooltip" title="Um pé isquêmico possui uma coloração roxa e está 
relacionado a uma circulação precária. A isquemia torna o pé muito 
sensível a qualquer lesão, fissura ou até mesmo a inflamação. É 
importante ficar atento a estes sinais , pois essa falta de sangue pode 
estar relacionado a obstrução das grandes artérias que irrigam a perna ou 
pela oclusão das microartérias que irrigam os tecidos dos pés."><input 
type='radio' value='Cor Roxa' name='opadd' id='op16' /><span 
id='occurrence_s15'>Pele Roxa</span></label>
        <label for='op17' style='margin-right: 15px;' data-
toggle="tooltip" title="Juntamente com sensação de queda da temperatura, 
o pé de coloração mais branca que o normal indica diminuição do fluxo 
sanguíneo. Isso geralmente é observado ao elevar os pés - palidez à 
elevação e ao colocar os pés no chão, surge o rubor postural, denotando 
má circulação. Procure atendimento médico. É uma situação de emergência, 
pois pode haver obstrução de alguma importante artéria."><input 
type='radio' value='Cor Pálida' name='opadd' id='op17' /><span 
id='occurrence_s16'>Pele Pálida</span></label>


                                <div class="clear"></div><br/><br/>
                                <div>
                                        <b id='occurrence_tip3'>Utilize 
os botões abaixo para girar o pé ou mudar a orientação.</b><br/>
                                        <canvas id="game" width="640" 
height="480"></canvas>
                                </div>


                                <div class="clear"></div><br/>
                                <div id='msg4' style='color: 
#CC2233;'></div>
                                <br/>
                                <input class="button submit" 
onclick="javascript:ValidateStep4();" value="<?php echo 
getLanguage("OCCURRENCE_BUTTON", "Concluir questionário");?>" 
type="button">

                        </div>




                        <div id='etapa5'  style='display: <?php if($oc4 
>= 1) echo "inline"; else echo "none"; ?>; height: 800px;'>
```

```
                                        <h2>Parabéns! Você respondeu
todos o questionários de ocorrência!</h2><br/><br/>

                                        <b style="color:
#428BCA;">Próximos passos</b><br/>
                                        &bull; Exercícios;<br/>
                                        &bull; Tirar suas dúvidas com os
especialistas;<br/>

                                        <!--<br/><br/><h2>Sua próxima
avaliação: <b style="color: #428BCA;">26/07/2017</b></h2> -->
                        </div>





                        </div>
                </div>
        </div>
</div>


<!--
<div  class="content_row alt-style-1 no-composer overlap fullwidth">
        <div class="content_row_wrapper clearfix ">
                <div class="col col-sm-12 col-xs-12 ">
                        <div class="single post-1872 staff type-staff
status-publish has-post-thumbnail hentry" id="person-1872">

                        <div align="center">
                                <canvas id="game" width="640"
height="480"></canvas>
                        </div>

                        <br/><br/>

                        <h2>Imagens do meu pé em 09/05/2016</h2>
                        <img src="images/pe3d/bolhape.jpg" style='width:
200px;' />


                        </div>
                </div>


        </div>
</div>
-->






<script type="text/javascript">
        jQuery(document).ready(function() { Preload(); });
</script>





<?php
        if(isset($_POST['action']))
        {
                $action = $_POST['action'];
```

```
                if($action == "tutorial")
                {

        require_once('../MysqlDB.php');
                                                                $Obj = new ScriptDB();

                                                                $sql = "UPDATE
saedd_users SET tutorial = 1 WHERE id =
'".intval($_POST["user_id"])."';";

                                                                if($Obj->ExecQuery($sql))
                                                                {
                                                                        echo "1";
                                                                }
                                                                else
                                                                {
                                                                          echo $Obj-
>GetLastError();
                                                                }

                                                                $Obj->CloseDB();


                }

                return;
        }
?>

<script>

                <?php
                        require_once('php/MysqlDB.php');
                        $id = $_SESSION['id'];

                        $Obj = new ScriptDB();
                        $Obj->Connect();
                        $Obj->ExecQuery("SELECT tutorial FROM saedd_users
WHERE id = '$id'");

                        $tutorial = intval($Obj->GetQueryValue(0, 0));

                        if(isset($_POST["tutorial"]) ||
isset($_GET["tutorial"]) || $tutorial == 0) {
                                echo "
                                        jQuery(document).ready(function()
{

        introJs().oncomplete(function() {
                                                                        updateTutorial();
                                                                }).onexit(function() {


        }).setOption({'nextLabel': 'Próximo', 'prevLabel': 'Anterior',
'skipLabel': 'Pular tutorial', 'doneLabel': 'Finalizar'}).start();
                                        });
                                ";
                        }
                ?>


        function updateTutorial()
        {
           jQuery.ajax({
                type: "POST",
```

```
                url: "php/pages/user_start.php",
                data: {user_id : <?php echo $_SESSION['id']; ?>,  action
: "tutorial"}
          })
         .done(function(html) {

          });

     }

</script>

        <link rel="stylesheet" href="css/introjs.css" type="text/css"
media="screen" />

        <script src="js/intro.js"></script>

        <script type="text/javascript"
src="js/plupload/plupload.full.min.js"></script>



<link rel='stylesheet' href='css/myform.css' type='text/css' media='all'
/>

<style type="text/css">
#myform input[type="text"],
#myform input[type="date"],
#myform input[type="datetime"],
#myform input[type="email"],
#myform input[type="number"],
#myform input[type="search"],
#myform input[type="time"],
#myform input[type="url"],
#myform input[type="password"],
#myform textarea,
#myform select {

    border-top: 1px solid #f0f0f0;
    border-left: 1px solid #f0f0f0;
    border-bottom: 1px solid #FAFAFA;
    border-right: 1px solid #FAFAFA;
    background: #e0e0e0;
}

.alt-style-1 input[type="button"]:hover {background-color: #FC9D0D;}
.alt-style-1 input[type="button"] {background-color: #428bca; font-
weight: bold;}

.alt-style-1 input[type="button"]:hover.extra  {background-color: #BBB;}
.alt-style-1 input[type="button"].extra  {background-color: #888; font-
weight: bold;}
b span{color: #428bca;}



.default-style input[type="button"]:hover {background-color: #FC9D0D;}
.default-style input[type="button"] {background-color: #428bca; font-
weight: bold;}

.default-style input[type="button"]:hover.extra  {background-color:
#BBB;}
.default-style input[type="button"].extra  {background-color: #888; font-
weight: bold;}
```

```
</style>

        <?php include("php/pages/internal.php"); ?>

        <?php
                function GetTranslation($str)
                {
                        if($str == "registration")
                           return getLanguage("AUTO_TIME1", "Fiz meu
cadastro no SOPED!");
                        if($str == "fall")
                           return getLanguage("AUTO_TIME2", "Respondi o
questionário de quedas.");
                        if($str == "mnsi")
                           return getLanguage("AUTO_TIME3", "Respondi o
questionário de triagem neoropática.");
                        if($str == "foot_health")
                           return getLanguage("AUTO_TIME4", "Respondi o
questionário de saúde dos pés.");
                        if($str == "occurrence_1")
                           return getLanguage("AUTO_TIME5", "Respondi o
questionário de ocorrências 1.");
                        if($str == "occurrence_2")
                           return getLanguage("AUTO_TIME6", "Respondi o
questionário de ocorrências 2.");
                        if($str == "occurrence_3")
                           return getLanguage("AUTO_TIME7", "Respondi o
questionário de ocorrências 3.");
                        if($str == "occurrence_4")
                           return getLanguage("AUTO_TIME8", "Criei o
modelo 3D das ocorrências do meu pé.");
                        if($str == "program_start")
                           return getLanguage("AUTO_TIME9", "Iniciei meu
programa de exercícios.");
                }
         ?>

<div  class="content_row default-style no-composer overlap fullwidth">
        <div class="content_row_wrapper clearfix " data-step="1" data-
intro="<b>Seja bem vindo ao Soped!</b><br/>Gostaríamos de apresentar um
tutorial do software. Ele irá se repetir até você completá-lo.<br/>Esse é
o Painel do usuário, com as instruções do que fazer." data-
position='top'>


                <div class="vc_col-sm-3 wpb_column vc_column_container
vc_custom_1421091415717" >
                        <div class="wpb_wrapper" data-step="14" data-
intro="Parabéns! Você está pronto para iniciar o uso do SoPed! Que tal
começar modificando sua foto?" data-position='right'>
                                <article  class="icon-content-box
content-box icon-top icon-style-3">
```

```
<?php

                        require_once('php/MysqlDB.php');
                        $id = $_SESSION['id'];
                        echo "<strong id='auto_sopedid'>Seu código 
SOPED:</strong> $id";

                        $Obj = new ScriptDB();
                        $Obj->Connect();
                        $Obj->ExecQuery("SELECT 
DATE_FORMAT(access_datetime,'%d/%m/%Y %H:%i:%s') FROM saedd_access WHERE 
user_id = '$id' ORDER BY access_datetime DESC LIMIT 2");

                        $count = $Obj->Get('RecordCount');
                        $last = "";
                        if($count == 2)
                           $last = $Obj->GetQueryValue(1, 0);
                        else if($count == 1)
                          $last = "Primeiro acesso";


                        $Obj->ExecQuery("SELECT gender, stars, (SELECT 
COUNT(*) FROM saedd_foothealth WHERE user_id = saedd_users.id), (SELECT 
COUNT(*) FROM saedd_occur_03 WHERE user_id = saedd_users.id), (SELECT 
COUNT(*) FROM saedd_quests WHERE user_id = saedd_users.id AND quest = 
'occurrence_4'), (SELECT COUNT(*) FROM saedd_quests WHERE user_id = 
saedd_users.id AND quest = 'program_start') FROM saedd_users WHERE id = 
'$id'");
                        $gender = $Obj->GetQueryValue(0, 0);
                        $stars = $Obj->GetQueryValue(0, 1);
                        $foot = intval($Obj->GetQueryValue(0, 2));
                        $oc = intval($Obj->GetQueryValue(0, 3));
                        $foot3d = intval($Obj->GetQueryValue(0, 4));
                        $start = intval($Obj->GetQueryValue(0, 5));

                        $rec = "<a href='index.php?page=user_auto' 
id='auto_guide1'>Responda a auto-avaliação</a>";

                        if($foot == 0)
                          $rec = "<a href='index.php?page=user_auto' 
id='auto_guide1'>Responda a auto-avaliação</a>";
                        else if($foot3d == 0)
                          $rec = "<a href='index.php?page=occurrence' 
id='auto_guide2'>Responda ao questionário de ocorrências</a>";
                        else if($start != 0)
                          $rec = "<a href='index.php?page=program' 
id='auto_guide3'>Continue os exercícios</a>";
                        else if($oc == 1)
                          $rec = "<a href='index.php?page=program' 
id='auto_guide4'>Inicie os exercícios</a>";


                                                $path = "";

                                                if($gender == "M")
                                                  $path = 
"images/icons/male_avatar.png";
                                                else
                                                  $path = 
"images/icons/female_avatar.png";


        if(file_exists("files/users/$id.jpg"))
```

```
                                                  $path =
"files/users/$id.jpg";

                                                echo "<img id='upbt'
src='$path' style='width: 300px; border: 1px solid #BBB; padding: 2px;
background: #FFF; cursor: pointer;' /><br/><br/><span
id='auto_phototip'>Clique no avatar e mude a foto.</span><br/><br/><div
id='saida'></div>";
                                                echo
"<script>jQuery('#stars').val('$stars');</script>";
/**/
                                        ?>

                                </article>
                        </div>
                </div>

                <div class="vc_col-sm-9 wpb_column vc_column_container
vc_custom_1421091415717" >
                        <div class="wpb_wrapper">
                                <article  class="icon-content-box
content-box icon-top icon-style-3">
                                        <h4><strong><?php echo
$_SESSION['name']; ?>,<br/><span id="auto_welcome">seja bem vindo ao
SoPeD!</span></strong></h4>
                                        <br/><p><strong
id='auto_lastaccess'>Seu último acesso:</strong> <?php echo $last; ?></p>
                                        <p data-step="6" data-
intro="Quando você concluir uma etapa do software receberá uma
recompensa. Para desbloquear as funções de questionário de ocorrências e
exercício você precisará de estrelas." data-position='top'><strong
id='auto_mystars'>Minhas estrelas (Nível de aptidão SoPeD):</strong><br/>
<img src='images/star.png' > x <?php echo $stars; ?> </p>
                                        <p data-step="9" data-intro="Você
não precisa lembrar de todas essas instruções, basta seguir as
recomendações do software (bem aqui)." data-position='top'><strong
id='auto_guidance'>Recomendação:</strong> <b><?php echo $rec; ?></p>

                                </article>
                        </div>
                </div>

        </div>
</div>


<div  class="content_row row vc_row wpb_row vc_row-fluid alt-style-1
fullwidth border-top border-bottom" >
        <div class="content_row_wrapper  default" style="padding-
top:60px;padding-bottom:60px;">
                <div class="vc_col-sm-12 wpb_column vc_column_container"
>
                        <div class="wpb_wrapper" data-step="5" data-
intro="O resultado das avaliações e questionário são apresentados e
detalhados nesse bloco (que é o seu histórico). É importante ler e seguir
as observações indicadas aqui." data-position='top'>

                                <div class="rt_heading_wrapper style-4">
                                        <span class="punchline"
id='auto_history'>Histórico</span>
                                        <h4 class="rt_heading
with_punchline style-4" id="auto_myresults">Meus dados e resultados</h4>
                                </div>
```

```php
<?php
			require_once('php/MysqlDB.php');
			$id = $_SESSION['id'];

			$Obj = new ScriptDB();
			$Obj->Connect();
			$Obj->ExecQuery("SELECT name as 'Nome Completo', birthday as 'Data de nascimento', gender as 'Sexo',cpf as 'CPF',email as 'Email',diabetic as 'Portador de diabetes',year as 'Ano de descoberta',insulin as 'Usa insulina',medicines as 'Quantidade de medicamentos que faz uso',cancer as 'Operado de cancer',cellphone as 'Telefone Celular',homephone 'Telefone Fixo',state AS 'Estado',city as 'Cidade',address as 'Endereço',origin as 'Como chegou ao SoPeD', project as 'Projeto', diseases as doenças FROM saedd_users WHERE id = '$id'");

			$count = $Obj->Get('RecordCount');
			$cols = $Obj->Get('ColumnCount')-1;

			echo "<div id='Unique-ID' class='rt-toggle CSS-Class-Name'>
				<ol>";


			for($i=0; $i<$count; $i++)
			{
				$num = 1;
				$title = "Meus dados pessoais";

				$content = "";

				for($j=0; $j<$cols; $j++)
				  $content .= "<b>".$Obj->GetFieldName($j).": </b> ".($Obj->GetQueryValue($i, $j))."<br/>";
				$content .= "<h4>Outras doenças</h4>".str_replace("|",", ",$Obj->GetQueryValue($i, $cols))."<br/><br/><br/>";
				echo "<li>
					<div class='toggle-head'><div class='toggle-number'>$num</div>
					<div class='toggle-title'>$title</div></div>
					<div class='toggle-content'><div class='wpb_text_column wpb_content_element '>
					<div class='wpb_wrapper'>
						<p style='font-weight: normal;'>$content</p></div></div>
					</div>
				</li>";

			}

			$Obj->ExecQuery("SELECT (SELECT q1+q2+q3+q4+q5+q6+q7+q8+q9+q10+q11+q12+q13+q14+q15 FROM saedd_mnsi WHERE user_id = '$id') as 'Questionário MNSI', (SELECT q1+q2+q3+q4+q5 FROM saedd_falls WHERE user_id = '$id') as 'Questionário de quedas', (SELECT round((q1+q2+q3+q4+q5+q6+q7+q8+q9+q10+q11+q12)/12) FROM saedd_foothealth WHERE user_id = '$id') AS 'Questionário de saúde dos pés'");

			$count = $Obj->Get('RecordCount');
			$cols = $Obj->Get('ColumnCount');




			for($i=0; $i<$count; $i++)
```

```
                        {
                                $num = 2;
                                $title =
getLanguage("AUTO_SELFTEST1","Resultado dos questionários de auto-
avaliação");

                                $content = "";

                                $points = $Obj->GetQueryValue(0, 0);
                                if($points == "")
                                  $points = -1;

                                $points2 = $Obj->GetQueryValue(0, 1);
                                if($points2 == "")
                                  $points2 = -1;

                                $points3 = $Obj->GetQueryValue(0, 2);
                                if($points3 == "")
                                  $points3 = -1;

                                $points  = intval($points);
                                $points2 = intval($points2);
                                $points3 = intval($points3);



                                if($points != -1)
                                  $content .= "<h4>Triagem Neuropática
(MNSI)</h4>";

                                if($points == 0)
                                   $content .= "<b><span>AUSENCIA DE
SINTOMAS DA NEUROPATIA</span></b><p>Aparentemente você não tem os
principais sintomas da neuropatia diabética. Sendo assim, continue
cuidando da sua saúde e faça os exercícios recomendados para os seus pés.
A prevenção é fundamental para que seus pés e sua saúde continuem
saudáveis. Continue preenchendo as avaliações para saber se você poderá
fazer os exercícios disponíveis no software.</p>";
                                else if($points <= 3 && $points != -1)
                                   $content .= "<b><span>POSSIBILIDADE DA
NEUROPATIA LEVE</span></b><p>Verificamos que você tem chances de ter uma
NEUROPATIA LEVE. Se você não conversou com sua equipe de saúde, está na
hora de informá-los sobre o que está sentindo. Os exercícios podem lhe
ajudar. Continue preenchendo as avaliações para saber se você poderá
fazer os exercícios recomendados pelo software.</p>";
                                else if($points <= 7 && $points != -1)
                                   $content .= "<b><span>POSSIBILIDADE DE
NEUROPATIA MODERADA</span></b><p>Você tem chances de ter uma NEUROPATIA
MODERADA. Já conversou com a equipe de saúde sobre o assunto? Você não
está sozinho, mais de 50% da população diabética pode ser afetado pela
neuropatia. Os exercícios disponibilizados aqui podem lhe ajudar, mas não
dispensa uma visita no especialista. Continue preenchendo as etapas
seguintes, para saber se você poderá fazer os exercícios disponíveis no
software e vamos exercitar o seu pé! Preparado(a)?</p>";
                                else if($points >= 8)
                                   $content .= "<b><span>POSSIBILIDADE DE
NEUROPATIA GRAVE</span></b><p>Atenção! Você tem chance de ter uma
NEUROPATIA GRAVE. É fundamental que você converse sua equipe de saúde. E
lembre-se, você não está sozinho, mais de 50% da população diabética pode
ser afetado pela neuropatia. Continue preenchendo os questionários para
saber se você poderá fazer os exercícios disponíveis no software. Os
exercícios podem ser úteis, mas não dispensa uma visita no
especialista.</p>";

                                if($points2 != -1)
```

```
                                $content .= "<h4>Questionários de
quedas</h4>";

                              if($points2 == 0)
                                $content .= "<b><span>VOCÊ ESTÁ COM BOM
EQUILÍBRIO!</span></b><p>Aparentemente você não tem problemas com
equilíbrio. Continue prestando atenção ao se exercitar e ao fazer
atividades do dia a dia. Continue preenchendo as avaliações para saber se
você poderá fazer os exercícios disponíveis no software.</p>";
                              else if($points2 < 3 && $points2 != -1)
                                $content .= "<b><span>VOCÊ ESTÁ COM
EQUILÍBRIO RAZOÁVEL!</span></b><p>Atenção! Você pode estar propício à
quedas. Redobre a atenção ao se exercitar e ao fazer atividades do dia a
dia. Continue preenchendo as avaliações para saber se você poderá fazer
os exercícios disponíveis no software. Os exercícios podem ser úteis, mas
não dispensa uma visita no especialista.</p>";
                              else if($points2 >= 3)
                                $content .= "<b><span>VOCÊ ESTÁ COM
SÉRIAS DIFICULDADES DE EQUILÍBRIO!</span></b><p>Atenção! Você pode estar
propício à quedas. Redobre a atenção ao se exercitar e ao fazer
atividades do dia a dia. É fundamental que você converse sua equipe de
saúde. Continue preenchendo os questionários para saber se você poderá
fazer os exercícios disponíveis no software. Os exercícios podem ser
úteis, mas não dispensa uma visita no especialista.</p>";

                              if($points3 != -1)
                                $content .= "<h4>Saúde dos pés</h4>";

                              if($points3 <= 1 && $points3 != -1)
                                 $content .= "<b><span>PÉS
SAUDÁVEIS</span></b><p>Parabéns! Aparentemente os seus pés estão sendo
bem cuidados. Continue cuidando da sua saúde e fique atento. Ao final das
avaliações, saberemos se você está apto para a realização dos exercícios.
A prevenção é fundamental para que seus pés e sua saúde continuem
saudáveis. Aproveite o material disponível neste software e exercite o
seus pés!</p>";
                              else if($points3 == 2)
                                 $content .= "<b><span>LEVE LIMITAÇÃO
NA SAÚDE DOS PÉS</span></b><p>Verificamos que há um pouco de limitação na
saúde dos seus pés. Eles doem durante a realização de pequenas atividades
e já começam a lhe incomodar. É o momento de ficar atento aos cuidados
com os seus pés, pois a prevenção é fundamental para que se mantenham
saudáveis. Continue preenchendo as avaliações para saber se você poderá
fazer os exercícios disponíveis aqui. Os exercícios podem ser úteis, mas
não dispensa uma boa conversa com a equipe de saúde que lhe
acompanha.</p>";
                              else if($points3 == 3)
                                 $content .= "<b><span>PÉ POUCO
SAUDÁVEL</span></b><p>Aparentemente os seus pés são pouco saudáveis.
Alguns estudos mostram que os pacientes não conversam sobre os pés com os
profissionais da saúde. Se você é parte desta população, mude sua
atitude. Os cuidados com os pés e a inspeção frequente são recomendados
para todas as pessoas com diabetes, pois se trata de uma doença
silenciosa. Continue preenchendo as avaliações para saber se você poderá
fazer os exercícios disponíveis aqui. Os exercícios podem ser úteis, mas
não dispensa uma visita no especialista.</p>";
                              else if($points3 == 4)
                                 $content .= "<b><span>PÉ NÃO
SAUDÁVEL</span></b><p>Atenção! A nossa avaliação sugere que aparentemente
os seus pés não estão saudáveis. É importante lembrar que os cuidados com
os pés e a inspeção frequente são recomendados para todas as pessoas com
diabetes, pois trata-se de uma doença silenciosa. É de fundamental
importância que você converse com os profissionais da saúde sobre quais
cuidados deve ter e que também crie o hábito de dar mais atenção para
seus pés. Continue preenchendo as avaliações para saber se você poderá
```

```
fazer os exercícios disponíveis aqui. Os exercícios podem ser muito
úteis, mas não dispensam uma visita no especialista.</p>";

                                if($points != -1)
                                {
                                        echo "<li>
                                                <div class='toggle-
head'><div class='toggle-number'>$num</div>
                                                <div class='toggle-
title'>$title</div></div>
                                                <div class='toggle-
content'><div class='wpb_text_column wpb_content_element'>
                                                <div class='wpb_wrapper'
style='font-weight: normal;'>

        <br/><br/>$content<br/><br/><br/>
                                                </div>
                                                </div>
                                                </div>
                                             </li>";
                                }

                        }




                        $Obj->ExecQuery("SELECT (SELECT
fr_q1+fr_q2+fr_q3+fr_q4+fl_q1+fl_q2+fl_q3+fl_q4 FROM saedd_occur_01 WHERE
user_id = '$id') as 'oc1', (SELECT
fr_q1+fr_q2+fr_q3+fr_q4+fr_q5+fr_q6+fl_q1+fl_q2+fl_q3+fl_q4+fl_q5+fl_q6
FROM saedd_occur_02 WHERE user_id = '$id') as 'oc2', (SELECT
fr_q1+fr_q2+fr_q3+fr_q4+fr_q5+fr_q6+fr_q7+fl_q1+fl_q2+fl_q3+fl_q4+fl_q5+f
l_q6+fl_q7 FROM saedd_occur_03 WHERE user_id = '$id') AS 'oc3'");

                        $count = $Obj->Get('RecordCount');
                        $cols = $Obj->Get('ColumnCount');




                        for($i=0; $i<$count; $i++)
                        {
                                $num = 2;
                                $title =
getLanguage("AUTO_SELFTEST2","Resultado dos questionários de
ocorrências");

                                $content = "";

                                $points = $Obj->GetQueryValue(0, 0);
                                if($points == "")
                                  $points = -1;

                                $points2 = $Obj->GetQueryValue(0, 1);
                                if($points2 == "")
                                  $points2 = -1;

                                $points3 = $Obj->GetQueryValue(0, 2);
                                if($points3 == "")
                                  $points3 = -1;

                                $points  = intval($points);
                                $points2 = intval($points2);
```

```
                                $points3 = intval($points3);

                                if($points > 0)
                                  $content .= "<h4>Seu pé tem ocorrências
genética ou unhas soltas (Joanete, dedos em garra ou martelo)</h4>Procure
um profissional da saúde para tratar os seus pés e ter uma melhor
qualidade de vida.<br/><br/><br/>";

                                if($points2 > 0)
                                  $content .= "<h4>Seus pés têm
ocorrências comuns (Dor, calos, rachadura, micoses, etc.)</h4>Procure um
profissional da saúde para tratar os seus pés e ter uma melhor qualidade
de vida.<br/><br/><br/>";

                                if($points3 > 0)
                                  $content .= "<h4>Seus pés têm
ocorrências graves (Pús, sangramento, feridas, coloração)</h4><b
style='color: #cc2233;'>Você não está apto a realizar exercícios sem
consultar um especialista!</b><br/><br/><br/>";

                                if($points != -1)
                                {
                                        echo "<li>
                                                <div class='toggle-
head'><div class='toggle-number'>$num</div>
                                                <div class='toggle-
title'>$title</div></div>
                                                <div class='toggle-
content'><div class='wpb_text_column wpb_content_element'>
                                                <div class='wpb_wrapper'
style='font-weight: normal;'>

        <br/><br/>$content<br/><br/><br/>
                                                </div>
                                                </div>
                                                </div>
                                             </li>";
                                }

                        }



                        echo "</ol>
                                </div>";
?>

                        </div>
                </div>
        </div>
</div>




<div  class="content_row row vc_row wpb_row vc_row-fluid default-style
fullwidth border-top border-bottom" >
        <div class="content_row_wrapper  default" style="padding-
top:60px;padding-bottom:60px;">
                <div class="vc_col-sm-12 wpb_column vc_column_container"
>
                        <div class="wpb_wrapper">
                                <div class="rt_heading_wrapper style-4">
                                        <span class="punchline"
id="auto_timeline">Linha do Tempo</span>
```

```
                                        <h4 class="rt_heading
with_punchline style-4" id="auto_timeline_history" >Minha história no
SoPeD</h4>
                                </div>
                                <section  class="timeline">

<?php



                        $Obj->Connect();
                        $Obj->ExecQuery("SELECT
DATE_FORMAT(quest_datetime,'%d/%m/%Y %H:%i:%s'), quest FROM saedd_quests
WHERE user_id = '$id' ORDER BY quest_datetime DESC");
                        $count = $Obj->Get('RecordCount');

                        for($i=0; $i<$count; $i++)
                        {
                                echo "
                                        <div>
                                                <span class='event-date'>
                                                <span class='day'>".$Obj-
>GetQueryValue($i, 0)."</span>
                                                </span>
                                                <div class='event-
details'>

        <p>".GetTranslation($Obj->GetQueryValue($i, 1))."</p>
                                                </div>
                                        </div>

                                        ";
                        }

?>


                                </section>
                        </div>
                </div>
        </div>
</div>



<script type="text/javascript">



var uploader = new plupload.Uploader({
  browse_button: 'upbt',
  url : 'js/plupload/upload.php?path=',
filters: {
  mime_types : [
    { title : "Imagem", extensions : "jpg,JPG,jpge,JPGE" }
  ],
  max_file_size: "2mb"
}
});



uploader.init();

uploader.bind('FilesAdded', function(up, files) {
```

```
  var html = '';
  plupload.each(files, function(file) {

  var fileName = file.name;
  uploader.settings.url =
"js/plupload/upload.php?path=files/users/&id="+<?php echo $id; ?>;

   document.getElementById('saida').innerHTML += "Enviando <b>"
+file.name+"</b> (<b id='"+file.id+"'></b> de
"+plupload.formatSize(file.size)+")";

  });

  document.getElementById('saida').innerHTML += html;
  uploader.start();
});

uploader.bind('UploadProgress', function(up, file) {
        document.getElementById(file.id).innerHTML = file.percent + "%";
});

uploader.bind('Error', function(up, err) {
    if(err.code == '-601')
        document.getElementById('saida').innerHTML = "Arquivo enviado não
é um formato válido: <b>"+err.file.name+"</b>.<br/>Envie em '.JPG'";
    else if(err.code == '-600')
        document.getElementById('saida').innerHTML = "Arquivo enviado é
grande demais: <b>"+err.file.name+"</b> O limite é '2 MB'.";
    else
        document.getElementById('saida').innerHTML = err.code + ": " +
err.message;
});

uploader.bind('FileUploaded', function(up, file, info){
        jQuery('#saida').html(file.name+" enviado com sucesso!");
        jQuery('#upbt').prop('src','files/users/<?php echo $id; ?>.jpg');
});
</script>

<script type='text/javascript'>

        function GetStars(count, page)
        {
                if(count > parseInt(jQuery("#stars").val()) &&
jQuery("#stars").val() != "")
                {
                   alert("Você precisa de pelo menos "+count+" estrela
para acessar essa opção.\nPara conseguir essas estrelas siga as
instruções do painel principal.");
                   return;
                }

                window.location = 'index.php?page='+page;
        }

</script>

<input type='hidden' id='stars' value='0' />

<div  class="content_row row vc_row wpb_row vc_row-fluid  alt-style-1
fullwidth border_grid fixed_heights" >
        <div class="content_row_wrapper  default" style="padding-
top:40px;padding-bottom:40px; margin-bottom: 40px;">
```

```
                <table style="width: 100%; text-align: center;" data-
step="2" data-intro="Para utilizar qualquer funcionalidade do software,
utilize o menu principal." data-position='bottom'>
                        <tr>
                                <td data-step="3" data-intro="Guarde bem
esse menu. Sempre que quiser voltar a essa tela, clique aqui." data-
position='bottom'>
                                                        <a
href="index.php?page=user_start" title="" target="_self">
                                                                <img
src="images/icons/op5.png" style='width: 64px;' /><br/>
                                                                <span
id='internal_m1'>Início</span>
                                                        </a>
                                </td>

                                <td data-step="4" data-intro="Aqui está a
primeira opção que você deve acessar no software: a auto-avaliação. A
cada 30 dias você precisa responder a auto-avaliação do software. Essa
ferramenta te permitira saber o seu grau de neuropatia." data-
position='bottom'>


                                                        <a
href="index.php?page=user_auto" title="Box Heading" target="_self">
                                                                <img
src="images/icons/op6.png" style='width: 64px;' /><br/>
                                                                <span
id='internal_m2'>Avaliação</span>
                                                        </a>
                                <td >



                                <td data-step="7" data-intro="Após
responder a Auto-avaliação você receberá uma estrela e desbloqueará essa
função, que criará um retrato 3D do seu pé. A cada 30 dias você precisa
responder a auto-avaliação do software e isso nos indicará se você está
apto ou não a realizar exercícios." data-position='bottom'>
                                                        <a
href="javascript:GetStars(1,'occurrence');" title="Box Heading"
target="_self">
                                                                <img
src="images/icons/foot.png" style='width: 64px;' /><br/>
                                                                <span
id='internal_m3'>Ocorrências</span>
                                                        </a>
                                </td>
                                <!--
                                <td data-step="8" data-
intro="Opcionalmente você poderá responder ao checklist de ocorrências,
que nos fornecerá um questionário preciso da saúde do seu pé e nos
indicará se você está apto ou não a realizar exercícios." data-
position='bottom'>
                                                        <a
href="javascript:GetStars(1,'checklist');" title="Box Heading"
target="_self">
                                                                <img
src="images/icons/check.png" style='width: 64px;' /><br/>
                                                                <span
id='internal_m4'>Checklist</span>
                                                        </a>
                                </td>
                                -->
```

```
                                <td data-step="10" data-intro="Se o
software informar que você está apto a realizar exercícios clique aqui.
ESSE É O CORAÇÃO DO SOFTWARE. Você precisa de pelo menos 3 estrelas para
iniciar os exercícios. Assim que iniciar será apresentada as regras de
uso." data-position='bottom'>
                                                        <a
href="javascript:GetStars(2,'program');" title="Box Heading"
target="_self">
                                                                <img
src="images/icons/op2.png" style='width: 64px;' /><br/>
                                                                <span
id='internal_m5'>Exercícios</span>
                                                        </a>
                                </td>

                                <td data-step="11" data-intro="Você
poderá interagir com outros usuário do software, conversar e ver as
conquistas deles, além de trocar seus pontos por prêmios." data-
position='bottom'>
                                                <b class="heading">
                                                        <a
href="index.php?page=social" title="Box Heading" target="_self">
                                                                <img
src="images/social.png" style='width: 64px;' /><br/>
                                                                <span
id='internal_m6'>Social</span>
                                                        </a>
                                                </b>
                                </td>

                                <td data-step="12" data-intro="Você
poderá enviar dúvidas para os especialistas, rever as explicações e
solicitar explicações sobre o software nesse menu." data-
position='bottom'>
                                                        <a
href="index.php?page=questions" title="Box Heading" target="_self">
                                                                <img
src="images/icons/Help.png" style='width: 64px;' /><br/>
                                                                <span
id='internal_m7'>Dúvidas</span>
                                                        </a>
                                </td>

                                <td data-step="13" data-intro="Clique
nesse botão para finalizar o uso do software. Não deixe o software aberto
depois do uso." data-position='bottom'>
                                                        <a
href="index.php?page=start&action=logout" title="" target="_self">
                                                        <img
src="images/icons/sair.png" style='width: 64px;' /><br/>
                                                                <span
id='internal_m8'>Sair</span>
                                                        </a>
                                </td>
                        </tr>
                </table>

        </div>
</div>


<div  class="content_row row vc_row wpb_row vc_row-fluid default-style
fullwidth" style="position:relative;overflow:hidden;">
        <div class="rt-parallax-background" data-rt-parallax-
direction="1" data-rt-parallax-effect="horizontal" style="background-
```

```
image: url(images/portfolio_header.jpg);background-repeat:
repeat;background-size: cover;background-position: right
top;width:100%;height:100%;top:0;"></div>
        <div class="content_row_wrapper  default" style="padding-
top:25px;padding-bottom:15px;">
                <div class="vc_col-sm-12 wpb_column vc_column_container"
>
                        <div class="wpb_wrapper">
                                <div class="rt_heading_wrapper style-4">
                                        <h1 class="rt_heading  style-4"
id="home_title">Importância dos exercícios</h1>
                                </div>
                        </div>
                </div>
        </div>
</div>


<div  class="content_row row vc_row wpb_row vc_row-fluid default-style
fullwidth" >
        <div class="content_row_wrapper  default" style="padding-
top:40px;padding-bottom:0px;">
                <div class="vc_col-sm-8 wpb_column vc_column_container" >
                        <div class="wpb_wrapper">
                                <div class="rt_quote" >
                                        <p style="text-align: justify;
font-size: 15px;">
                                                <span class="icon-quote-
left"></span>
<span id="home_content">
O Sistema de Orientação ao Pé Diabético (SoPeD) tem por objetivo
personalizar um programa de exercícios para os pés e tornozelos de
pessoas com Diabetes Mellitus considerando as condições de cada
indivíduo. Além de facilitar a realização destes exercícios, o software
dá o suporte para facilitar o autocuidado e a autoavaliação a fim de
promover a melhora da saúde dos pés. Este programa possibilita a
prevenção, tratamento e manutenção da saúde de pessoas com pé diabético
ou que não atingiram essa condição, mas possuem fatores de risco.
<br/><br/>
Os pés de um indivíduo com diabetes são o principal alvo de complicações
biomecânicas que irão resultar na perda de mobilidade articular, fraqueza
da musculatura do pé e perdas de sensibilidade. No entanto, ações
preventivas como: EXERCÍCIOS ESPECÍFICOS para o pé são altamente
recomendadas para atrasar ou mesmo evitar tais complicações. Ações estas
que podem reduzir o impacto da doença sobre a qualidade de vida da pessoa
com diabetes.
<br/><br/>
<b>A PREVENÇÃO É A MELHOR MODALIDADE TERAPÊUTICA PARA A POLINEUROPATIA
DIABÉTICA.</b>
<br/><br/>
A principal característica dessa ferramenta é permitir que, de acordo com
a dificuldade enfrentada em cada exercício, o software consiga ajustar a
dificuldade para a próxima realização de exercício, seguindo as
possibilidades de cada pessoa.
</span>
                                                <span class="icon-quote-
right"></span>
                                        </p>
                                </div>

<div id="Unique-ID" class="rt-toggle CSS-Class-Name"><ol><li
class=""><div class="toggle-head"><div class="toggle-number"><span
class="icon-search"></span></div><div class="toggle-title"
id="home_learnmore">Entendendo um pouco mais</div></div><div
class="toggle-content"><div class="wpb_text_column wpb_content_element ">
```

```
<div class="wpb_wrapper">
<p id='home_learnmore_content'>

A medida que a polineuropatia progride, os seus músculos, articulações e
estruturas neurais são prejudicadas, <b>principalmente as articulações
dos pés e tornozelos</b>.<br/><br/>
Todos estes fatores irão levar a uma piora na qualidade de vida da pessoa
com diabetes, este que poderá ficar dependente de outras pessoas para
realizar atividades simples do dia a dia e, numa progressão mais
avançada, caso não cuide atentamente da saúde dos pés, poderá ter úlceras
que é um fator primordial para aumentar o risco de amputação.
Acreditamos, com base em estudos, que a prática de exercícios específicos
para os pés e tornozelos, focando nestas perdas articulares e musculares
podem prevenir o aparecimento destas desordens citadas, visto que
exercícios propostos no software visam prevenir estas articulações e
musculatura que são afetadas pelo aparecimento da neuropatia diabética,
que nada mais é do que a junção de todos estes sintomas e sinais. Somente
os exercícios específicos para esta musculatura podem atuar positivamente
para prevenir estes danos que a polineuropatia causa.
<br/><br/>

<a href="index.php?page=publicacoes">Confira alguns estudos aqui</a>

</p></div></div>
</div></li>
</ol>
</div>


                                <div class="vc_empty_space"
style="height: 32px" ><span class="vc_empty_space_inner"></span></div>
                        </div>
                </div>
                <div class="vc_col-sm-4 wpb_column vc_column_container" >
                        <div class="wpb_wrapper">
                                <div class="wpb_single_image
wpb_content_element vc_align_center">
                                        <figure class="wpb_wrapper
vc_figure">
                                                <div
class="vc_single_image-wrapper vc_box_border_grey"><img
src="images/face.png" class="vc_single_image-img attachment-full"
style="height: 300px;" /></div>
                                        </figure>
                                </div>
                        </div>
                </div>
        </div>
</div>



<div  class="content_row row vc_row wpb_row vc_row-fluid alt-style-1
fullwidth border_grid fixed_heights" >

        <div class="content_row_wrapper  default" style="padding-
top:50px;padding-bottom:50px;"><div class="vc_col-sm-6 wpb_column
vc_column_container" >
                <div class="wpb_wrapper">
                        <div class="rt_heading_wrapper style-6">

                                                <h4 class="rt_heading
style-6" ><span class="icon-glyph-2 heading_icon"></span> <span
id="home_lastnews">Últimas notícias</span></h4>
```

```
                                        </div><section class="latest_news
clearfix style-1"><article>

                                        <div class="date">
                                                <span
class="day">20</span>
                                                <span class="year">Mar

2016</span>
                                        </div>


                                        <div class="text">
                                                <h5
class="clean_heading"><a class="title" href="" title=""
rel="bookmark">Lançamento da ferramenta</a></h5>
                                                <p>É com grande orgulho
que apresentamos a todos a nova versão do SoPeD.</p>
                                        </div>

</article>
<article>

                                        <div class="date">
                                                <span
class="day">19</span>
                                                <span class="year">Mar

2016</span>
                                        </div>


                                        <div class="text">
                                                <h5
class="clean_heading"><a class="title" href="" title="Audio Post"
rel="bookmark">Cadastro de dados</a></h5>
                                                <p>Já está disponível o
cadastro de usuários do SoPeD. O cadastro é rapido e fácil.</p>
                                        </div>

</article>
<article>

                                        <div class="date">
                                                <span
class="day">18</span>
                                                <span class="year">Mar

2016</span>
                                        </div>


                                        <div class="text">
                                                <h5
class="clean_heading"><a class="title" href="" title=""
rel="bookmark">Publicação de artigo do SoPeD</a></h5>
                                                <p>A equipe do SoPeD
publicou um artigo relatando os benefícios de uso da ferramenta em uma
importante revista de circulação internacional. <a href="#">Clique aqui
para baixá-lo!</a></p>
                                        </div>

</article>
</section>
                </div>
        </div>

        <div class="vc_col-sm-6 wpb_column vc_column_container" >
```

```
                <div class="wpb_wrapper">
                        <div class="rt_heading_wrapper style-6">

                                                        <h4 class="rt_heading
style-6" ><span class="icon-glyph-15 heading_icon"></span> <span
id="home_letknow">Vamos conhecer o SoPeD</span></h4>
                                                </div>
        <div class="wpb_text_column wpb_content_element ">
                <div class="wpb_wrapper">
                        <p id="home_letknow_tip">É muito fácil começar a
utilizar a ferramenta, basta seguir os passos indicados abaixo. Clique
nas abas para detalhar as informações.</p>

                </div>
        </div>
<div id="Unique-ID" class="rt-toggle CSS-Class-Name"><ol>


<li  class=""><div class="toggle-head"><div class="toggle-
number">1</div><div class="toggle-title" id="home_letknow_t1">Clique Aqui
para iniciar</div></div><div class="toggle-content"><div
class="wpb_text_column wpb_content_element ">
<div class="wpb_wrapper">
<p id="home_letknow_c1">O menu principal, na lateral do site, ficará fixo
durante todo o uso da ferramenta. Usuário de Tablet e Smartphones verão o
menu na parte superior do dispositivo! Mesmo que você já tenha feito o
primeiro acesso, clique sempre nesse menu.</p></div></div>
</div></li>

<li  class=""><div class="toggle-head"><div class="toggle-
number">2</div><div class="toggle-title" id="home_letknow_t2">Preencha o
formulário de dados pessoais</div></div><div class="toggle-content"><div
class="wpb_text_column wpb_content_element ">
<div class="wpb_wrapper">
<p id="home_letknow_c2">Clique em "Não tenho cadastro" e preencha o
formulário de dados para o acesso. Os campos mais importante são o e-mail
e a senha de acesso, que deverá ser informado para que suas atividades
sejam salvas.</p></div></div>
</div></li>

<li  class=""><div class="toggle-head"><div class="toggle-
number">3</div><div class="toggle-title" id="home_letknow_t3">Responda ao
questionário de auto-avaliação</div></div><div class="toggle-
content"><div class="wpb_text_column wpb_content_element ">
<div class="wpb_wrapper">
<p id="home_letknow_c3">Os questionários de autoavaliação são
fundamentais para acompanhar o estado de saúde de seus pés. Além do mais,
os exercícios só são liberados após a conclusão da autoavaliação. Pois é
através dela que verificaremos se você tem condições para realizar os
exercícios aqui propostos.</p></div></div>
</div></li>

<li  class=""><div class="toggle-head"><div class="toggle-
number">4</div><div class="toggle-title" id="home_letknow_t4">Inicie os
exercícios</div></div><div class="toggle-content"><div
class="wpb_text_column wpb_content_element ">
<div class="wpb_wrapper">
<p id="home_letknow_c4">Após a conclusão da autoavaliação, caso você
esteja apto a realizar os exercícios disponibilizados neste software,
usufrua do jogo aqui proposto. Este jogo inclui exercícios específicos
para os pés. Dedique-se e se mantenha no topo. Mas atenção, não é a
quantidade de exercícios e repetições que fazem de você o melhor, e sim a
qualidade e dedicação que você disponibiliza em cada exercício.
Preparado?</p></div></div>
</div></li>
```

```
<li  class=""><div class="toggle-head"><div class="toggle-
number">5</div><div class="toggle-title" id="home_letknow_t5">Acompanhe a
sua evolução</div></div><div class="toggle-content"><div
class="wpb_text_column wpb_content_element ">
<div class="wpb_wrapper">
<p id="home_letknow_c5">Todas as suas avaliações ficam disponíveis no seu
painel, facilitando o acompanhamento de sua evolução. Além do mais é
possível acompanhar todas as atividades realizadas na sua linha do
tempo.</p></div></div>
</div></li></ol>


</div>
</div>
</div>
</div>
</div>





<?php
        $mystyle1 = "default-style";
        include("php/pages/reasons.php");
?>




<!--
<div  class="content_row row vc_row wpb_row vc_row-fluid alt-style-1
fullwidth" >
        <div class="content_row_wrapper  default" style="padding-
top:50px;padding-bottom:50px;">
                <div class="vc_col-sm-12 wpb_column vc_column_container"
>
                        <div class="wpb_wrapper">
                                <section id="testimonial-650650"
class="border_grid testimonials clearfix  left " data-column-width="2">
                                        <div class="row">
                                                <div id="2353"
class="testimonial col col-sm-6  first loop post-2353 testimonial type-
testimonial status-publish has-post-thumbnail hentry">
                                                        <div
class="client_image gradient">
                                                                <img
src="images/face1.jpg" alt="" class="" />
                                                        </div>
                                                        <div class="text
with_image">
                                                                <p>

        <span class="icon-quote-left"></span>

        Foi notória a melhora das dores e da minha percebção de bem estar
após o uso da ferramenta. A possibilidade de utilizá-la nos meu horários
livre tornou tudo ainda mais fácil.<span class="icon-quote-right"></span>
                                                                </p>
                                                                <div
class="client_info">

        Paula Torres
```

```
                                                                                              -
<span>Administradora</span>
                                                                                      </div>
                                                                              </div>
                                                                      </div>
                                                                      <div id="1737"
class="testimonial col col-sm-6  last loop post-1737 testimonial type-
testimonial status-publish has-post-thumbnail hentry">
                                                                              <div
class="client_image gradient">
                                                                                      <img
src="images/face-8.jpg" alt="" class="" />
                                                                              </div>
                                                                              <div class="text
with_image">
                                                                                      <p>

        <span class="icon-quote-left"></span>

        Além de possibilitar uma auto-avaliação dos pacientes, a
ferramenta facilita o trabalho de profissionais de fisioterapia.<span
class="icon-quote-right"></span>
                                                                                      </p>
                                                                                      <div
class="client_info">

        Dr. Evandro Nunes
                                                                                              -
<span> Fisioterapeuta </span>   <br/>

        <a href="" target="_blank" title="" class="client_link">Centro de
Pesquisa</a>
                                                                                      </div>
                                                                              </div>
                                                                      </div>
                                                              </div>

                                              </section>
                                      </div>
                              </div>
        </div>
</div>
-->
<div  class="content_row row vc_row wpb_row vc_row-fluid default-style
fullwidth" style="position:relative;overflow:hidden;">
        <div class="rt-parallax-background" data-rt-parallax-
direction="1" data-rt-parallax-effect="horizontal" style="background-
image: url(images/portfolio_header.jpg);background-repeat:
repeat;background-size: cover;background-position: right
top;width:100%;height:100%;top:0;"></div>
        <div class="content_row_wrapper  default" style="padding-
top:25px;padding-bottom:15px;">
                <div class="vc_col-sm-12 wpb_column vc_column_container"
>
                        <div class="wpb_wrapper">
                                <div class="rt_heading_wrapper style-4">
                                        <h1 class="rt_heading  style-4"
id="diabetes_title">Diabetes</h1>
                                </div>
                        </div>
                </div>
        </div>
</div>
```

```
<div  class="content_row row vc_row wpb_row vc_row-fluid default-style
fullwidth border_grid fixed_heights" >
        <div class="content_row_wrapper  default" style="padding-
top:50px;padding-bottom:50px; min-height: 500px;">
                <div class="vc_col-sm-12 wpb_column vc_column_container"
>
                        <div class="wpb_wrapper">
                                <div class="rt_heading_wrapper style-6">
                                        <h4 class="rt_heading  style-6"
><span class="icon-glyph-15 heading_icon"></span> <span
id='diabetes_topic1'>Conheça mais sobre o Diabetes</span></h4>
                                </div>
                                <div class="wpb_text_column
wpb_content_element ">
                                        <div class="wpb_wrapper">
                                                <p
id="diabetes_topic1_tip">Gostaria de saber um pouco mais sobre diabetes?
Clique nas abas para detalhar as informações.</p>
                                        </div>
                                </div>





        <script type='text/javascript' src='js/pixi.min.js'></script>
        <script type='text/javascript' src='js/complicacoes.js'></script>


        <script type="text/javascript"
src="js/fancybox/jquery.fancybox.pack.js?v=2.1.5"></script>
        <link rel="stylesheet" type="text/css"
href="js/fancybox/jquery.fancybox.css?v=2.1.5" media="screen" />
        <link rel="stylesheet" type="text/css"
href="js/fancybox/helpers/jquery.fancybox-buttons.css?v=1.0.5" />
        <script type="text/javascript"
src="js/fancybox/helpers/jquery.fancybox-buttons.js?v=1.0.5"></script>


<script type="text/javascript">

        function updateDescription(title, description, path) {
                jQuery("#mytitle").html(title);
                jQuery("#mypicture").attr('src', path);
                jQuery("#mycontent").html(description);
                jQuery("#btdesc").trigger( "click" );
        }

        function addoccurencies() {

<?php
                require_once('php/MysqlDB.php');
                $Obj = new ScriptDB();

                $Obj->Connect();
                $Obj->ExecQuery("SELECT px, py, title, content, path FROM
saedd_diabetes WHERE language = '$langcode' ORDER BY title");

                $count = $Obj->Get('RecordCount');

                $html = "";

                for($i=0; $i<$count; $i++) {
                        $px = $Obj->GetQueryValue($i, 0);
```

```
                        $py = $Obj->GetQueryValue($i, 1);
                        $title =
str_replace("\r","<br/>",str_replace("\n","<br/>",str_replace("\"","&quot
;",$Obj->GetQueryValue($i, 2))));
                        $content =
str_replace("\r","<br/>",str_replace("\n","<br/>",str_replace("\"","&quot
;",$Obj->GetQueryValue($i, 3))));
                        $path = $Obj->GetQueryValue($i, 4);
                        $html .=
"insertOccurency($px,$py,\"$title\",\"$content\",'$path');\n";
                }

                $Obj->CloseDB();
                echo $html;
?>
        }

        jQuery(document).ready(function() { onRunning = addoccurencies;
Preload(); jQuery('.fancybox').fancybox();});




</script>

        <div id="inline1" style="width: 100%; height: auto; display:
none;">
                <h3 id='mytitle'></h3>
                <img src='' id='mypicture' style='width: 160px; margin: 0
auto' /><br/><br/>
                <p id='mycontent' style='max-width: 480px; text-align:
justify;'>
                </p>
        </div>

                <a class="fancybox" id='btdesc' href="#inline1"></a>



        <div id="Unique-ID" class="rt-toggle CSS-Class-Name">
                                        <ol>
                                                <li  class="">
                                                        <div
class="toggle-head">
                                                                <div
class="toggle-number">1</div>
                                                                <div
class="toggle-title" id="diabetes_topic1_toggle1">O que é diabetes?</div>
                                                        </div>
                                                        <div
class="toggle-content">
                                                                <div
class="wpb_text_column wpb_content_element "
id="diabetes_topic1_content1">

        <div class="wpb_wrapper">
                                                                                <p style

principal da insulina é promover a entrada de glicose para as células do
organismo de forma que ela possa ser aproveitada para as diversas
atividades celulares. Quando a pessoa tem diabetes o organismo não
fabrica insulina e não consegue utilizar a glicose adequadamente. O nível
de glicose no sangue fica alto - <b>a famosa hiperglicemia</b>. Se esse
quadro permanecer por longos períodos, poderá haver danos em órgãos,
vasos sanguíneos e nervos.
```

```
</p>

</div>
</div>
</div>
</li>
<li  class="">
<div
class="toggle-head">
<div
class="toggle-number">2</div>
<div
class="toggle-title" id="diabetes_topic1_toggle2">Tipos de Diabetes</div>
</div>
<div
class="toggle-content">
<div
class="wpb_text_column wpb_content_element ">

<div class="wpb_wrapper">
<p style
complicações.



Esse tipo de diabetes causa sintomas como: vontade frequente de urinar,
boca muito seca, perda de peso repentina, fome, cansaço e fraqueza. O
tratamento é feito com o uso diário de insulina acompanhados de uma dieta
alimentar.



representa a grande maioria dos casos (aproximadamente 90% dos casos). Os
sintomas são sede, vontade de urinar frequente, muita fome, dificuldade
na cicatrização de feridas e visão turva, geralmente há associação com
aumento de peso. O tratamento neste caso depende do estado de saúde do
indivíduo e normalmente é feito sem o uso da insulina. Na maior parte dos
casos feito com o uso de remédios orais, alimentação adequada e exercício
físico regular.
</p>

</div>
</div>
</div>
</li>

<li  class="">
<div
class="toggle-head">
<div
class="toggle-number">3</div>
<div
class="toggle-title" id="diabetes_topic1_toggle3">Complicações da
Diabetes</div>
</div>
<div
class="toggle-content">
<div
class="wpb_text_column wpb_content_element ">

<div class="wpb_wrapper">
<p id="d
<canvas id="game"></canvas><br/><br/>
</p>
```

```
        </div>
                                                                </div>
                                                        </div>
                                                </li>

                                                <li  class="">
                                                        <div
class="toggle-head">
                                                                <div
class="toggle-number">4</div>
                                                                <div
class="toggle-title" id="diabetes_topic1_toggle4">Como o SoPeD pode lhe
ajudar?</div>
                                                        </div>
                                                        <div
class="toggle-content">
                                                                <div
class="wpb_text_column wpb_content_element ">

        <div class="wpb_wrapper" >
                                                                                <p style
proporcionando informações, autoavaliações e cuidados, incluindo uma
série de exercícios personalizados de fácil realização em um ambiente de
escolha do indivíduo, sem a necessidade constante de um profissional por
perto. Esses exercícios geram uma intervenção precoce nas principais
causas de formação de úlcera e proporciona então uma melhor qualidade de
vida da pessoa com diabetes.</b></p>

        </div>
                                                                </div>
                                                        </div>
                                                </li>
                                        </ol>
                                </div>
                        </div>
                </div>
        </div>
</div>

<?php
        $mystyle1 = "alt-style-1";
        include("php/pages/reasons.php");
?>


<div  class="content_row row vc_row wpb_row vc_row-fluid <?php echo
$mystyle1; ?> fullwidth border_grid fixed_heights" >
        <div class="content_row_wrapper  default" style="padding-
top:40px;padding-bottom:40px;"><div class="vc_col-sm-4 wpb_column
vc_column_container vc_custom_1421091415717" >
                <div class="wpb_wrapper">
                        <article  class="icon-content-box content-box
icon-top icon-style-3"><div class="icon-holder"><a
href="index.php?page=start" title="" target="_self"><span class="icon-
new-link-1"></span></a></div><div class="text-holder">
        <h4 class="heading">
                <a href="index.php?page=start" title="Box Heading"
target="_self" id='reasons_use_title'>
                        Por que utilizar?
                </a>
        </h4> <p class="aligncenter" id='reasons_use_content'>Além de ser
fácil e gratuito a ferramenta se ajusta aos horários do paciente e
promove uma melhora do bem estar dos pés.</p>
</div></article>
```

```
                </div>
        </div>

        <div class="vc_col-sm-4 wpb_column vc_column_container" >
                <div class="wpb_wrapper">
                        <article  class="icon-content-box content-box
icon-top icon-style-3"><div class="icon-holder"><a
href="index.php?page=perguntar" title="" target="_self"><span
class="icon-new-user-1"></span></a></div><div class="text-holder">
        <h4 class="heading">
                <a href="index.php?page=perguntar" title="Box Heading"
target="_self" id='reasons_talk_title'>
                        Fale com um Especialista!
                </a>
        </h4> <p class="aligncenter" id='reasons_talk_content'>Em caso de
dúvidas consulte nossos especialistas online. Estamos sempre prontos para
atendê-lo!</p>
</div></article>
                </div>
        </div>

        <div class="vc_col-sm-4 wpb_column vc_column_container" >
                <div class="wpb_wrapper">
                        <article  class="icon-content-box content-box
icon-top icon-style-3"><div class="icon-holder"><a
href="index.php?page=compartilhar2" title="" target="_self"><span
class="icon-new-heart-1"></span></a></div><div class="text-holder">
        <h4 class="heading">
                <a href="index.php?page=compartilhar2" title="Box
Heading" target="_self" id='reasons_share_title'>
                        Gostou? Compartilhe!
                </a>
        </h4> <p class="aligncenter" id='reasons_share_content'>Indique o
SoPeD para amigos e pessoas da sua rede de contato. Você estará ajudando
a melhorar a ferramenta.</p>
</div></article>
                </div>
        </div>
</div>
</div>
<?php
        if(isset($_POST['action']))
        {
                $action = $_POST['action'];

                if($action == "mnsi")
                {

        require_once('../MysqlDB.php');
                                                          $Obj = new ScriptDB();

                                                          $Obj->Set("TableName",
"saedd_mnsi");
                                                          $Obj->Set("FieldsName",
"q1|q2|q3|q4|q5|q6|q7|q8|q9|q10|q11|q12|q13|q14|q15|user_id|registration_
date");
                                                          $Obj-
>BeforeInsert("registration_date","NOW()");

                                                          $sql = $Obj-
>CreateInsert();

                                                          $Obj->Connect();
                                                          $Obj->ExecQuery("DELETE
FROM saedd_mnsi WHERE user_id = '".intval($_POST['user_id'])."'");
```

```
                                                    $Obj->ExecQuery("DELETE
FROM saedd_falls WHERE user_id = '".intval($_POST['user_id'])."'");
                                                    $Obj->ExecQuery("DELETE
FROM saedd_foothealth WHERE user_id = '".intval($_POST['user_id'])."'");

                                                    if($Obj->ExecQuery($sql))
                                                    {
                                                            $sql = "INSERT
INTO saedd_quests(user_id,quest_datetime,quest)
VALUES('".$_POST['user_id']."',NOW(),'mnsi');";
                                                            $Obj-
>ExecQuery($sql);

                                                            echo "1";

                                                    }
                                                    else
                                                    {
                                                              echo $Obj-
>GetLastError();
                                                    }

                                                    $Obj->CloseDB();


                }
                else if($action == "fall")
                {

        require_once('../MysqlDB.php');
                                                    $Obj = new ScriptDB();

                                                    $Obj->Set('TableName',
'saedd_falls');
                                                    $Obj->Set('FieldsName',
'q1|q2|q3|q4|q5|q6|description|user_id|registration_date');
                                                    $Obj-
>BeforeInsert("registration_date","NOW()");

                                                    $sql = $Obj-
>CreateInsert();

                                                    $Obj->Connect();

                                                    if($Obj-
>ExecQuery(($sql)))
                                                    {
                                                            $sql = "INSERT
INTO saedd_quests(user_id,quest_datetime,quest)
VALUES('".$_POST['user_id']."',NOW(),'fall');";
                                                            $Obj-
>ExecQuery($sql);

                                                            echo "1";
                                                    }
                                                    else
                                                    {
                                                               echo $Obj-
>GetLastError();
                                                    }

                                                    $Obj->CloseDB();

                }
                else if($action == "footheath")
```

```
                {
                                                        session_start();

        require_once('../MysqlDB.php');
                                                        $Obj = new ScriptDB();

                                                        $Obj->Set('TableName',
'saedd_foothealth');
                                                        $Obj->Set('FieldsName',
'q1|q2|q3|q4|q5|q6|q7|q8|q9|q10|q11|q12|user_id|registration_date');
                                                        $Obj-
>BeforeInsert("registration_date","NOW()");

                                                        $sql = $Obj-
>CreateInsert();

                                                        $Obj->Connect();

                                                        if($Obj-
>ExecQuery(($sql)))
                                                        {
                                                                $sql = "INSERT
INTO saedd_quests(user_id,quest_datetime,quest)
VALUES('".$_POST['user_id']."',NOW(),'foot_health');";
                                                                $Obj-
>ExecQuery($sql);
                                                                $sql = "UPDATE
saedd_users SET stars = (stars+1) WHERE id =
'".trim($_POST['user_id'])."'";
                                                                $Obj-
>ExecQuery($sql);
                                                                echo "1";
                                                        }
                                                        else
                                                        {
                                                                  echo $Obj-
>GetLastError();
                                                        }

                                                        $Obj->CloseDB();

                }
                return;
        }
?>

<?php include("php/pages/back.php"); ?>

<link rel='stylesheet' href='css/myform.css' type='text/css' media='all'
/>

<style type="text/css">
#myform input[type="text"],
#myform input[type="date"],
#myform input[type="datetime"],
#myform input[type="email"],
#myform input[type="number"],
#myform input[type="search"],
#myform input[type="time"],
#myform input[type="url"],
#myform input[type="password"],
#myform textarea,
#myform select {

    border-top: 1px solid #f0f0f0;
```

```
    border-left: 1px solid #f0f0f0;
    border-bottom: 1px solid #FAFAFA;
    border-right: 1px solid #FAFAFA;
    background: #e0e0e0;
}

.default-style input[type="button"]:hover {background-color: #FC9D0D;}
.default-style input[type="button"] {background-color: #428bca; font-
weight: bold;}

.default-style input[type="button"]:hover.extra  {background-color:
#BBB;}
.default-style input[type="button"].extra  {background-color: #888; font-
weight: bold;}


h4 span {color: #428BCA;}
</style>




<script type="text/javascript">

        function ValidateMNSI()
        {
           var points = 0;
           jQuery("#msg").html("");

           for(i=1; i<=15; i++)
           {
              if(jQuery("#mnsi_"+i+"_r1").is(':checked') == false &&
jQuery("#mnsi_"+i+"_r2").is(':checked') == false)
              {
                 jQuery("#msg").html("A pergunta "+(i)+" está sem
resposta.");
                 return;
              }
               if(jQuery("#mnsi_"+i+"_r1").is(':checked'))
                 points++;
           }

            p1 = jQuery("input[name='mnsi_1']:checked").val();
            p2 = jQuery("input[name='mnsi_2']:checked").val();
            p3 = jQuery("input[name='mnsi_3']:checked").val();
            p4 = jQuery("input[name='mnsi_4']:checked").val();
            p5 = jQuery("input[name='mnsi_5']:checked").val();
            p6 = jQuery("input[name='mnsi_6']:checked").val();
            p7 = jQuery("input[name='mnsi_7']:checked").val();
            p8 = jQuery("input[name='mnsi_8']:checked").val();
            p9 = jQuery("input[name='mnsi_9']:checked").val();
            p10 = jQuery("input[name='mnsi_10']:checked").val();
            p11 = jQuery("input[name='mnsi_11']:checked").val();
            p12 = jQuery("input[name='mnsi_12']:checked").val();
            p13 = jQuery("input[name='mnsi_13']:checked").val();
            p14 = jQuery("input[name='mnsi_14']:checked").val();
            p15 = jQuery("input[name='mnsi_15']:checked").val();


           jQuery.ajax({
                type: "POST",
                url: "php/pages/user_auto.php",
                data: {q1 : p1, q2 : p2, q3 : p3, q4 : p4, q5 : p5, q6 :
p6 , q7 : p7, q8 : p8, q9 : p9, q10 : p10, q11 : p11, q12 : p12, q13 :
```

```
p13, q14 : p14, q15 : p15, user_id : <?php echo $_SESSION['id']; ?>,
action : "mnsi"}
           })
           .done(function(html) {
                if(html == "1")
                {
                        var result = "<h1>Resultado da auto-avaliação
MNSI:</h1><br/>";

                        if(points == 0)
                           result += "<h4><span>AUSENCIA DE SINTOMAS DA
NEUROPATIA</span></h4><p>Aparentemente você não tem os principais
sintomas da neuropatia diabética. Sendo assim, continue cuidando da sua
saúde e faça os exercícios recomendados para os seus pés. A prevenção é
fundamental para que seus pés e sua saúde continuem saudáveis. Continue
preenchendo as avaliações para saber se você poderá fazer os exercícios
disponíveis no software.</p>";
                        else if(points <= 3)
                           result += "<h4><span>POSSIBILIDADE DA
NEUROPATIA LEVE</span></h4><p>Verificamos que você tem chances de ter uma
NEUROPATIA LEVE. Se você não conversou com sua equipe de saúde, está na
hora de informá-los sobre o que está sentindo. Os exercícios podem lhe
ajudar. Continue preenchendo as avaliações para saber se você poderá
fazer os exercícios recomendados pelo software.</p>";
                        else if(points <= 7)
                           result += "<h4><span>POSSIBILIDADE DE
NEUROPATIA MODERADA</span></h4><p>Você tem chances de ter uma NEUROPATIA
MODERADA. Já conversou com a equipe de saúde sobre o assunto? Você não
está sozinho, mais de 50% da população diabética pode ser afetada pela
neuropatia. Os exercícios disponibilizados aqui podem lhe ajudar, mas não
dispensa uma visita no especialista. Continue preenchendo as etapas
seguintes, para saber se você poderá fazer os exercícios disponíveis no
software e vamos exercitar o seu pé! Preparado(a)?</p>";
                        else if(points >= 8)
                           result += "<h4><span>POSSIBILIDADE DE
NEUROPATIA GRAVE</span></h4><p>Atenção! Você tem chance de ter uma
NEUROPATIA GRAVE. É fundamental que você converse com sua equipe de
saúde. E lembre-se, você não está sozinho, mais de 50% da população
diabética pode ser afetada pela neuropatia. Continue preenchendo os
questionários para saber se você poderá fazer os exercícios disponíveis
no software. Os exercícios podem ser úteis, mas não dispensa uma visita
no especialista.</p>";

                        result += "<br/><br/><div class='submit-
button'><input class='button submit' onclick=\"javascript:window.location
= 'index.php?page=user_auto';\" value='Clique aqui para continuar...'
type='button'><span class='loading'></span></div>";
                        result += "<br/><br/><h2>O que é
neuropatia?</h2><p>A neuropatia diabética é uma complicação do diabetes
que acomete os nervos periféricos, ocasionada por altos níveis de
glicemia no sangue. As lesões podem afetar mais de 50% das pessoas com
diabetes . Dentre os principais sinais e sintomas da neuropatia estão o
formigamento, dor ou queimação e perda de sensibilidade nos pés e/ou
pernas. E como consequência a estes e outros sintomas, é possível não
notar pequenos cortes, bolhas ou feridas que podem se infectar e/ou
evoluir para úlceras. Para prevenir é preciso controlar a glicemia, de
modo a evitar danos neurológicos futuros. E é importante examinar os seus
pés e pernas todos os dias. A inspeção frequente é recomendada para todas
as pessoas que tem diabetes, pois trata-se de uma doença silenciosa.
<br/><br/>AVALIAÇÃO E OS EXERCÍCIOS FORNECIDOS PELO SOFTWARE NÃO
DISPENSAM O TRATAMENTO MÉDICO E CONSULTAS REGULARES COM A EQUIPE DE SAÚDE
(ENFERMEIROS, FISIOTERAPEUTAS, MÉDICOS, ETC.).</p>";

                        jQuery("#etapa1").html(result);
```

```
                        jQuery("html, body").animate({ scrollTop: 0 },
"slow");
                }
                else
                  jQuery("#msg").html("Erro: "+html);
           });

        }



        function ValidateFall()
        {
           var p1 = 0, p2 = 0, p3 = 0, p4 = 0, p5 = 0, p6 = 0;

           jQuery("#msg2").html("");

           for(i=1; i<=4; i++)
           {
              if(jQuery("#fall"+i+"_r1").is(':checked') == false &&
jQuery("#fall"+i+"_r2").is(':checked') == false)
              {
                 jQuery("#msg2").html("A pergunta "+(i)+" está sem
resposta.");
                 return;
              }
           }

           if(jQuery("#fall1_r1").is(':checked') == true)
             p1 = 1;

           if(jQuery("#fall2_r1").is(':checked') == true)
             p2 = 1;

           if(jQuery("#fall3_r1").is(':checked') == true)
             p3 = 1;

           if(jQuery("#fall4_r1").is(':checked') == true)
           {
              if(jQuery("#fall4_extra").val() == "")
              {
                 jQuery("#msg2").html("Quantas quedas você levou?");
                 return;
              }

              p4 = jQuery("#fall4_extra").val();

              if(jQuery("#fall5_r1").is(':checked') == false &&
jQuery("#fall5_r2").is(':checked') == false)
              {
                 jQuery("#msg2").html("A pergunta 5 está sem resposta.");
                 return;
              }

              if(jQuery("#fall5_r1").is(':checked') == true)
                p5 = 1;

              if(jQuery("#fall6").val() == "7" &&
jQuery("#description").val() == "")
              {
                 jQuery("#msg2").html("Descreva a causa da última
queda.");
                 return;
              }
```

```
            p6 = jQuery("#fall6").val();
        }



        jQuery.ajax({
             type: "POST",
             url: "php/pages/user_auto.php",
             data: {q1 : p1, q2 : p2, q3 : p3, q4 : p4, q5 : p5, q6 :
p6, description :  jQuery("#description").val(), user_id : <?php echo
$_SESSION['id']; ?>,  action : "fall"}
          })
         .done(function(html) {
             if(html == "1")
             {
                     var result = "<h1>Resultado da auto-avaliação de
quedas:</h1><br/>";
                     points = p1+p2+p3+p4+p5;

                     if(points == 0)
                        result += "<h4><span>Você está com bom
equilíbrio!</span></h4><p>Aparentemente você não tem problemas com
equilíbrio. Continue prestando atenção ao se exercitar e ao fazer
atividades do dia a dia. Continue preenchendo as avaliações para saber se
você poderá fazer os exercícios disponíveis no software.</p>";
                     else if(points < 3)
                        result += "<h4><span>Você está com equilíbrio
razoável!</span></h4><p>Atenção! Você pode estar propício à quedas.
Redobre a atenção ao se exercitar e ao fazer atividades do dia a dia.
Continue preenchendo as avaliações para saber se você poderá fazer os
exercícios disponíveis no software. Os exercícios podem ser úteis, mas
não dispensa uma visita no especialista.</p>";
                     else if(points >= 3)
                        result += "<h4><span>Você está com sérias
dificuldades de equilíbrio!</span></h4><p>Atenção! Você pode estar
propício à quedas. Redobre a atenção ao se exercitar e ao fazer
atividades do dia a dia. É fundamental que você converse com sua equipe
de saúde. Continue preenchendo os questionários para saber se você poderá
fazer os exercícios disponíveis no software. Os exercícios podem ser
úteis, mas não dispensa uma visita no especialista.</p>";

                     result += "<br/><br/><div class='submit-
button'><input class='button submit' onclick=\"javascript:window.location
= 'index.php?page=user_auto';\" value='Clique aqui para continuar...'
type='button'><span class='loading'></span></div>";


                     jQuery("#etapa2").html(result);
                     jQuery("html, body").animate({ scrollTop: 0 },
"slow");
             }
             else
               jQuery("#msg2").html("Erro: "+html);
          });

      }

         function ValidateHeath()
         {
             for(i=1; i<=12; i++)
             {
                 if(typeof jQuery("input[name='q"+i+"']:checked").val()
=== "undefined")
                 {
```

```
                    jQuery("#msg3").html("A pergunta "+i+" está sem
resposta.");
                    return;
                }
            }


            p1 = jQuery("input[name='q1']:checked").val();
            p2 = jQuery("input[name='q2']:checked").val();
            p3 = jQuery("input[name='q3']:checked").val();
            p4 = jQuery("input[name='q4']:checked").val();
            p5 = jQuery("input[name='q5']:checked").val();
            p6 = jQuery("input[name='q6']:checked").val();
            p7 = jQuery("input[name='q7']:checked").val();
            p8 = jQuery("input[name='q8']:checked").val();
            p9 = jQuery("input[name='q9']:checked").val();
            p10 = jQuery("input[name='q10']:checked").val();
            p11 = jQuery("input[name='q11']:checked").val();
            p12 = jQuery("input[name='q12']:checked").val();

           points =
(parseInt(p1)+parseInt(p2)+parseInt(p3)+parseInt(p4)+parseInt(p5)+parseIn
t(p6)+parseInt(p7)+parseInt(p8)+parseInt(p9)+parseInt(p10)+parseInt(p11)+
parseInt(p12))/12.0;
           points = Math.round(points);

           jQuery.ajax({
                type: "POST",
                url: "php/pages/user_auto.php",
                data: {q1 : p1, q2 : p2, q3 : p3, q4 : p4, q5 : p5, q6 :
p6 , q7 : p7, q8 : p8, q9 : p9, q10 : p10, q11 : p11, q12 : p12, user_id
: <?php echo $_SESSION['id']; ?>,  action : "footheath"}
          })
         .done(function(html) {
                if(html == "1")
                {
                        var result = "<h1>Resultado da auto-avaliação de
saúde dos pés:</h1><br/>";

                        if(points <= 1)
                           result += "<h4><span>PÉS
SAUDÁVEIS</span></h4><p>Parabéns! Aparentemente os seus pés estão sendo
bem cuidados. Continue cuidando da sua saúde e fique atento. Ao final das
avaliações, saberemos se você está apto para a realização dos exercícios.
A prevenção é fundamental para que seus pés e sua saúde continuem
saudáveis. Aproveite o material disponível neste software e exercite o
seus pés!</p>";
                        else if(points == 2)
                           result += "<h4><span>LEVE LIMITAÇÃO NA SAÚDE
DOS PÉS</span></h4><p>Verificamos que há um pouco de limitação na saúde
dos seus pés. Eles doem durante a realização de pequenas atividades e já
começam a lhe incomodar. É o momento de ficar atento aos cuidados com os
seus pés, pois a prevenção é fundamental para que se mantenham saudáveis.
Continue preenchendo as avaliações para saber se você poderá fazer os
exercícios disponíveis aqui. Os exercícios podem ser úteis, mas não
dispensa uma boa conversa com a equipe de saúde que lhe acompanha.</p>";
                        else if(points == 3)
                           result += "<h4><span>PÉ POUCO
SAUDÁVEL</span></h4><p>Aparentemente os seus pés são pouco saudáveis.
Alguns estudos mostram que os pacientes não conversam sobre os pés com os
profissionais da saúde. Se você é parte desta população, mude sua
atitude. Os cuidados com os pés e a inspeção frequente são recomendados
para todas as pessoas com diabetes, pois se trata de uma doença
silenciosa. Continue preenchendo as avaliações para saber se você poderá
```

```
fazer os exercícios disponíveis aqui. Os exercícios podem ser úteis, mas
não dispensa uma visita no especialista.</p>";
                        else if(points == 4)
                           result += "<h4><span>PÉ NÃO
SAUDÁVEL</span></h4><p>Atenção! A nossa avaliação sugere que
aparentemente os seus pés não estão saudáveis. É importante lembrar que
os cuidados com os pés e a inspeção frequente são recomendados para todas
as pessoas com diabetes, pois trata-se de uma doença silenciosa. É de
fundamental importância que você converse com os profissionais da saúde
sobre quais cuidados deve ter e que também crie o hábito de dar mais
atenção para seus pés. Continue preenchendo as avaliações para saber se
você poderá fazer os exercícios disponíveis aqui. Os exercícios podem ser
muito úteis, mas não dispensam uma visita no especialista.</p>";

                        result += "<br/><br/><div class='submit-
button'><input class='button submit' onclick=\"javascript:window.location
= 'index.php?page=user_start';\" value='Concluir questionário'
type='button'><span class='loading'></span></div>";

                        result += "<br/><br/><h2>Obrigado por responder
todas as etapas!</h2><p>Você está pronto para avançar no SoPed. Por
responder os questionários você ganhou uma estrela! <h2><img
src='images/star.png' /> x 1</h2><br/><b style='color: #428BCA;'>As
estrelas liberam ferramentas no software que auxiliam no cuidado dos seus
pés.</b></p>";

                        jQuery("#etapa3").html(result);
                        jQuery("html, body").animate({ scrollTop: 0 },
"slow");
                }
                else
                  alert(html);
          });

        }


        function ShowItem1(res)
        {
                if(res == 1)
                {
                   jQuery("#plab").css("display", "inline");
                   jQuery("#extra").css("display", "inline");

                }
                else
                {
                   jQuery("#plab").css("display", "none");
                   jQuery("#extra").css("display", "none");

                }
        }

        function ShowItem2(res)
        {
                if(res == 6)
                   jQuery("#plab2").css("visibility", "visible");
                else
                   jQuery("#plab2").css("visibility", "hidden");
        }

</script>

<?php
                        require_once('php/MysqlDB.php');
```

```
                        $id = $_SESSION['id'];

                        $Obj = new ScriptDB();
                        $Obj->Connect();
                        $Obj->ExecQuery("SELECT (SELECT COUNT(*) FROM
saedd_mnsi WHERE user_id = '$id'), (SELECT COUNT(*) FROM saedd_falls
WHERE user_id = '$id'), (SELECT COUNT(*) FROM saedd_foothealth WHERE
user_id = '$id'), (SELECT DATE_FORMAT(registration_date, '%d/%m/%Y') FROM
saedd_foothealth WHERE user_id = '$id'), COALESCE((SELECT DATEDIFF(NOW(),
registration_date) FROM saedd_foothealth WHERE user_id = '$id'),0),
(SELECT DATE_FORMAT(DATE_ADD(DATE(registration_date), interval 30 day),
'%d/%m/%Y') FROM saedd_foothealth WHERE user_id = '$id')");

                        $mnsi = $Obj->GetQueryValue(0, 0);
                        $fall = $Obj->GetQueryValue(0, 1);
                        $heath = $Obj->GetQueryValue(0, 2);

                        $date = $Obj->GetQueryValue(0, 3);
                        $days = intval($Obj->GetQueryValue(0, 4));
                        $next = ($Obj->GetQueryValue(0, 5));

                        if($days >= 30) {
                                $mnsi = 0;
                                $fall = 0;
                                $heath = 0;
                        }
?>


<div  class="content_row default-style no-composer overlap fullwidth">
        <div class="content_row_wrapper clearfix " style='min-height:
800px;'>
                <div class="col col-sm-12 col-xs-12 ">
                        <div class="single post-1872 staff type-staff
status-publish has-post-thumbnail hentry" id="person-1872">


                        <div id='etapa1' style='display: <?php if($mnsi
== 0) echo "inline"; else echo "none"; ?>'>

                                <h2>Perguntas sobre a Neuropatia (MSNI) -
<b style='color: #428BCA;'>Etapa 1 de 3</b></h2><br/><br/>


                                <p>Este questionário é utilizado como
avaliação de apoio para descrever o grau de neuropatia do indivíduo. É
importante relembrar que ele não dispensa uma visita com profissionais de
saúde da área (médicos, fisioterapeutas, enfermeiros, terapeutas
ocupacionais, etc). O questionário abaixo foi adaptado e validado do <a
data-toggle="tooltip" title="Baseado em Michigan Neuropathy Screening
Instrument - Citado em 01/10/2013"
href="http://www.med.umich.edu/mdrtc/profs/documents/svi/MNSI_patient.pdf
" target="_blank">Instrumento de triagem Neuropática de do instituto de
Michigan</a>.
                                <br/><br/>Por favor, dedique alguns
minutos para responder às seguintes perguntas sobre a sensação em suas
pernas e pés.
                                <br/><br/><b  style='color:
#428BCA;'>Marque sim ou não com base em como você geralmente se
sente.</b><br/>
                                </p>

                                <form id='questions' name='questions'
method="post" action='index.php'>
                                <?php
```

```
                                        $perguntas = array("", "Suas
pernas e/ou pés ficam dormentes?", "Você costuma ter dor em queimação nas
suas pernas e/ou pés?",
                                        "Seus pés são hipersensíveis ao
toque a ponto de incomodar?", "Você tem cãimbras musculares em suas
pernas e/ou pés?",
                                        "Você costuma ter sensação de
agulhadas ou formigamento nas suas pernas e/ou pés?", "Dói ou incomoda
quando a roupa de cama toca sua pele das suas pernas ou pés?",
                                        "Quando você entra no banho
(chuveiro ou banheira), é dificil dizer se a água está quente ou fria nos
pés ou pernas?","Você já teve ou tem uma ferida aberta (úlcera) no seu
pé?",
                                        "Um médico alguma vez disse que
você tem neuropatia diabética?", "Você sente fraqueza geral na maior
parte do tempo?",
                                        "Seus sintomas são piores à
noite?", "Suas pernas doem quando você anda?",
                                        "Você consegue sentir seus pés
quando anda?", "A pele dos seus pés é tão seca a ponto de abrir
rachaduras?",
                                        "Você já sofreu alguma
amputação?"
                                        );


                                        for($i=1; $i<count($perguntas);
$i++)
                                        {
                                           echo "       <h6>".$i." -
".$perguntas[$i]."</h6>
                                                        <label
for='mnsi_".$i."_r1'><b> <input type='radio' name='mnsi_$i' value='1'
id='mnsi_".$i."_r1' /> Sim</b></label>
                                                        <label
for='mnsi_".$i."_r2' style='margin-left: 100px;'><b> <input type='radio'
value='0' name='mnsi_$i' id='mnsi_".$i."_r2' /> Não</b></label><br/><br/>
                                                        ";
                                        }
                                ?>

                                <div class="clear"></div>
                                <div id='msg' style='color:
#CC2233;'></div><br/>

                                <input type='hidden' value='0'
id='points' name='points' />

                                </form>

                                <input class="button submit"
onclick="javascript:ValidateMNSI();" value="Próxima etapa" type="button"
/>
                        </div>




                        <div id='etapa2'  style='display: <?php if($mnsi
== 1 && $fall == 0) echo "inline"; else echo "none"; ?>;'>
                                <h2>Investigação de Quedas - <b
style='color: #428BCA;'>Etapa 2 de 3</b></h2><br/><br/>

                                <form id='questions2' name='question2s'
method="post" action='index.php'>
```

```
                                <h6>1 - Tem medo de cair?</h6>
                                <label for="fall1_r1"><b> <input
type="radio" id="fall1_r1" name="fall1" value='1' /> Sim</b></label>
                                <label for="fall1_r2" style='margin-left:
100px;'><b> <input type="radio" value='0' name="fall1" id="fall1_r2" />
Não</b></label>

                                <br/><br/>
                                <h6>2 - Como você avalia o seu
equilíbrio?</h6>
                                <label for="fall2_r1"><b> <input
type="radio" id="fall2_r1" name="fall2"  value='0' /> Ruim</b></label>
                                <label for="fall2_r2" style='margin-left:
100px;'><b> <input type="radio" value='1' id="fall2_r2" name="fall2" />
Bom</b></label>

                                <br/><br/>
                                <h6>3 - Sente fraqueza nas pernas?</h6>
                                <label for="fall3_r1"><b> <input
type="radio" id="fall3_r1" name="fall3"  /> Sim</b></label>
                                <label for="fall3_r2" style='margin-left:
100px;'><b> <input type="radio" id="fall3_r2" name="fall3" />
Não</b></label>


                                <br/><br/>
                                <h6>4 - Sofreu queda nos últimos 6
meses?</h6>
                                <label for="fall4_r1"><b> <input
type="radio" name="fall4" id="fall4_r1"
onclick="javascript:ShowItem1(1);" /> Sim</b></label>
                                <label for="fall4_extra" id="plab"
style='margin-left: 20px; display: none;' ><b>Quantas? <input type="text"
style="border: 1px solid #666; background: #FFF; height: 24px; color:
#222; padding: 0; margin: 0; padding-left: 5px; width: 50px;"
id="fall4_extra" /> </b></label>
                                <label for="fall4_r2" style='margin-left:
100px;'><b> <input type="radio" onclick="javascript:ShowItem1(0);"
id="fall4_r2" name="fall4" /> Não</b></label>

                                <div style='display: none;' id="extra">

                                <br/><br/>
                                <h6>5 - Teve fratura, bateu a cabeça ou
precisou procurar hospital por conta da última queda?</h6>
                                <label for="fall5_r1"><b> <input
type="radio" id="fall5_r1" name="fall5" /> Sim</b></label>
                                <label for="fall5_r2" style='margin-left:
100px;'><b> <input type="radio" id="fall5_r2" name="fall5" />
Não</b></label>


                                <br/><br/>
                                <h6>6 - Qual foi a causa da última
queda?</h6>
                                <select size="1" name="fall6"  id="fall6"
style="border: 1px solid #666; background: #FFF; height: 24px; color:
#222; padding: 0; margin: 0; padding-left: 5px; width: 200px;"
onchange="javascript:ShowItem2(this.selectedIndex);">
                                        <option value='1'>Levantou muito
rápido</option>
                                        <option
value='2'>Tropeçou</option>
                                        <option value='3'>Perdeu o
equilíbrio</option>
```

```
                                        <option value='4'>Foi
empurrado</option>
                                        <option
value='5'>Desmaiou</option>
                                        <option value='6'>Perdeu forças
nas pernas</option>
                                        <option
value='7'>Outra...</option>
                                </select>

                                <label for="fall6_extra" id="plab2"
style='margin-left: 20px; visibility: hidden;' ><b>Descreva: <input
type="text" style="border: 1px solid #666; background: #FFF; height:
24px; color: #222; padding: 0; margin: 0; padding-left: 5px; width:
300px;" name='description' id="description" /> </b></label>
                                </div>

                                </form>
                                <div class="clear"></div><br/>
                                <div id='msg2' style='color:
#CC2233;'></div><br/>

                                <input class="button submit"
onclick="javascript:ValidateFall();" value="Próxima etapa" type="button">
                        </div>


                        <div id='etapa3'  style='display: <?php if($fall
== 1 && $heath == 0) echo "inline"; else echo "none"; ?>;'>
                                <h2>Saúde dos pés - <b style='color:
#428BCA;'>Etapa 3 de 3</b></h2><br/><br/>

                                Este questionário pergunta a sua opinião
sobre a saúde dos seus pés. Se não tiver certeza sobre como responder uma
pergunta, por favor, forneça a melhor resposta que puder.

                                <br/><br/><b style='font-size: 14px;'>As
perguntas a seguir são sobre a dor nos pés que você sentiu <span
style="color: #428BCA;">durante a semana
passada</span>.</b><br/><br/><br/>


                                <h6>1 - Qual o nível de dor nos pés que
você sentiu durante a semana passada?</h6>
                                <label for="q1a1" style='margin-left:
0px;'><b> <input type="radio" name="q1" id="q1a1" value='0' />
Nenhuma</b></label>
                                <label for="q1a2" style='margin-left:
30px;'><b> <input type="radio" name="q1" id="q1a2" value='1' /> Muito
leve</b></label>
                                <label for="q1a3" style='margin-left:
30px;'><b> <input type="radio" name="q1" id="q1a3" value='2' />
Leve</b></label>
                                <label for="q1a4" style='margin-left:
30px;'><b> <input type="radio" name="q1" id="q1a4" value='3' />
Moderada</b></label>
                                <label for="q1a5" style='margin-left:
30px;'><b> <input type="radio" name="q1" id="q1a5" value='4' />
Forte</b></label>

                                <br/><br/>
                                <h6>2 - Seus pés lhe causaram
dificuldades em seu trabalho ou sem suas atividades?</h6>
```

```
                                <label for="q2a1" style='margin-left:
0px;'><b> <input type="radio" name="q2" id="q2a1" value='0'  /> Nem um
pouco</b></label>
                                <label for="q2a2" style='margin-left:
30px;'><b> <input type="radio" name="q2" id="q2a2" value='1' /> Um
pouco</b></label>
                                <label for="q2a3" style='margin-left:
30px;'><b> <input type="radio" name="q2" id="q2a3" value='2' />
Moderadamente</b></label>
                                <label for="q2a4" style='margin-left:
30px;'><b> <input type="radio" name="q2" id="q2a4" value='3' />
Bastante</b></label>
                                <label for="q2a5" style='margin-left:
30px;'><b> <input type="radio" name="q2" id="q2a5" value='4' />
Extremamente</b></label>

                                <br/><br/>
                                <h6>3 - Você foi limitado em algum tipo
de trabalho que você poderia fazer por causa de seus pés?</h6>
                                <label for="q3a1" style='margin-left:
0px;'><b> <input type="radio" name="q3" id="q3a1" value='0' /> Nem um
pouco</b></label>
                                <label for="q3a2" style='margin-left:
30px;'><b> <input type="radio" name="q3" id="q3a2" value='1' /> Um
pouco</b></label>
                                <label for="q3a3" style='margin-left:
30px;'><b> <input type="radio" name="q3" id="q3a3" value='2' />
Moderadamente</b></label>
                                <label for="q3a4" style='margin-left:
30px;'><b> <input type="radio" name="q3" id="q3a4" value='3' />
Bastante</b></label>
                                <label for="q3a5" style='margin-left:
30px;'><b> <input type="radio" name="q3" id="q3a5" value='4' />
Extremamente</b></label>


                                <br/><br/>
                                <h6>4 - Quanto a saúde dos seus pés
limita você a andar?</h6>
                                <label for="q4a1" style='margin-left:
0px;'><b> <input type="radio" name="q4" id="q4a1" value='0' /> Nem um
pouco</b></label>
                                <label for="q4a2" style='margin-left:
30px;'><b> <input type="radio" name="q4" id="q4a2" value='1' /> Um
pouco</b></label>
                                <label for="q4a3" style='margin-left:
30px;'><b> <input type="radio" name="q4" id="q4a3" value='2' />
Moderadamente</b></label>
                                <label for="q4a4" style='margin-left:
30px;'><b> <input type="radio" name="q4" id="q4a4" value='3' />
Bastante</b></label>
                                <label for="q4a5" style='margin-left:
30px;'><b> <input type="radio" name="q4" id="q4a5" value='4' />
Extremamente</b></label>


                                <br/><br/>
                                <h6>5 - Quanto a saúde dos seus pés
limita você a subir escadas?</h6>
                                <label for="q5a1" style='margin-left:
0px;'><b> <input type="radio" name="q5" id="q5a1" value='0' /> Nem um
pouco</b></label>
                                <label for="q5a2" style='margin-left:
30px;'><b> <input type="radio" name="q5" id="q5a2" value='1' /> Um
pouco</b></label>
```

```
                                <label for="q5a3" style='margin-left:
30px;'><b> <input type="radio" name="q5" id="q5a3" value='2' />
Moderadamente</b></label>
                                <label for="q5a4" style='margin-left:
30px;'><b> <input type="radio" name="q5" id="q5a4" value='3' />
Bastante</b></label>
                                <label for="q5a5" style='margin-left:
30px;'><b> <input type="radio" name="q5" id="q5a5" value='4' />
Extremamente</b></label>


                                <br/><br/>
                                <h6>6 - Como você classificaria a saúde
geral dos seus pés?</h6>
                                <label for="q6a1" style='margin-left:
0px;'><b> <input type="radio" name="q6" id="q6a1" value='0' />
Excelente</b></label>
                                <label for="q6a2" style='margin-left:
30px;'><b> <input type="radio" name="q6" id="q6a2" value='1' /> Muito
boa</b></label>
                                <label for="q6a3" style='margin-left:
30px;'><b> <input type="radio" name="q6" id="q6a3" value='2' />
Boa</b></label>
                                <label for="q6a4" style='margin-left:
30px;'><b> <input type="radio" name="q6" id="q6a4" value='3' />
Razoável</b></label>
                                <label for="q6a5" style='margin-left:
30px;'><b> <input type="radio" name="q6" id="q6a5" value='4' />
Ruim</b></label>


                                <br/><br/><b>As perguntas a seguir são
sobre as atividades que você poderia fazer em um <span style="color:
#428BCA;">dia normal</span>.</b><br/><br/><br/>

                                <h6>7 - Seus pés te limitam a participar
de atividades pesadas, como correr, levantar objetos ou participar de
esportes desgastantes?</h6>
                                <label for="q7a1" style='margin-left:
0px;'><b> <input type="radio" name="q7" id="q7a1" value='4' /> Sim,
limita muito</b></label>
                                <label for="q7a2" style='margin-left:
30px;'><b> <input type="radio" name="q7" id="q7a2" value='2' /> Sim,
limita um pouco</b></label>
                                <label for="q7a3" style='margin-left:
30px;'><b> <input type="radio" name="q7" id="q7a3" value='0' /> Não, não
limita em nada</b></label>
                                <br/><br/>

                                <h6>8 - Seus pés te limitam a participar
de atividades moderadas, como limpar a casa, levantar uma cadeira ou
nadar?</h6>
                                <label for="q8a1" style='margin-left:
0px;'><b> <input type="radio" name="q8" id="q8a1" value='4' /> Sim,
limita muito</b></label>
                                <label for="q8a2" style='margin-left:
30px;'><b> <input type="radio" name="q8" id="q8a2" value='2' /> Sim,
limita um pouco</b></label>
                                <label for="q8a3" style='margin-left:
30px;'><b> <input type="radio" name="q8" id="q8a3" value='0' /> Não, não
limita em nada</b></label>
                                <br/><br/>

                                <h6>9 - Seus pés te limitam a subir uma
ladeira?</h6>
```

```
                                <label for="q9a1" style='margin-left:
0px;'><b> <input type="radio" name="q9" id="q9a1" value='4' /> Sim,
limita muito</b></label>
                                <label for="q9a2" style='margin-left:
30px;'><b> <input type="radio" name="q9" id="q9a2" value='2' /> Sim,
limita um pouco</b></label>
                                <label for="q9a3" style='margin-left:
30px;'><b> <input type="radio" name="q9" id="q9a3" value='0' /> Não, não
limita em nada</b></label>
                                <br/><br/>

                                <h6>10 - Seus pés te limitam a subir um
lance de escadas?</h6>
                                <label for="q10a1" style='margin-left:
0px;'><b> <input type="radio" name="q10" id="q10a1" value='4' /> Sim,
limita muito</b></label>
                                <label for="q10a2" style='margin-left:
30px;'><b> <input type="radio" name="q10" id="q10a2" value='2' /> Sim,
limita um pouco</b></label>
                                <label for="q10a3" style='margin-left:
30px;'><b> <input type="radio" name="q10" id="q10a3" value='0' /> Não,
não limita em nada</b></label>
                                <br/><br/>

                                <h6>11 - Seus pés te limitam a levantar-
se de uma posição sentado?</h6>
                                <label for="q11a1" style='margin-left:
0px;'><b> <input type="radio" name="q11" id="q11a1" value='4' /> Sim,
limita muito</b></label>
                                <label for="q11a2" style='margin-left:
30px;'><b> <input type="radio" name="q11" id="q11a2" value='2' /> Sim,
limita um pouco</b></label>
                                <label for="q11a3" style='margin-left:
30px;'><b> <input type="radio" name="q11" id="q11a3" value='0' /> Não,
não limita em nada</b></label>
                                <br/><br/>

                                <h6>12 - Seus pés te limitam a caminhar
mais do que um quilômetro?</h6>
                                <label for="q12a1" style='margin-left:
0px;'><b> <input type="radio" name="q12" id="q12a1" value='4' /> Sim,
limita muito</b></label>
                                <label for="q12a2" style='margin-left:
30px;'><b> <input type="radio" name="q12" id="q12a2" value='2' /> Sim,
limita um pouco</b></label>
                                <label for="q12a3" style='margin-left:
30px;'><b> <input type="radio" name="q12" id="q12a3" value='0' /> Não,
não limita em nada</b></label>
                                <br/><br/>

                                <div id='msg3' style='color:
#cc2233;'></div>


                                <div class="clear"></div><br/>
                                <input class="button submit"
onclick="javascript:ValidateHeath();" value="Última Etapa" type="button">

                        </div>

                        <div id='etapa4'  style='display: <?php if($heath
== 1) echo "inline"; else echo "none"; ?>; height: 800px;'>
                                        <h2>Parabéns! Você respondeu em
<?php echo "<b style='color: #428BCA;'>$date</b>"; ?> todos os
questionários!</h2>
```

```
                                        <b style="color: 
#428BCA;">Próximos passos</b><br/>
                                        &bull; Questionários de 
ocorrências sobre os pés;<br/>
                                        &bull; Exercícios;<br/>
                                        &bull; Tirar suas dúvidas com os 
especialistas;<br/>

                                        <br/><br/><h2>Sua próxima 
avaliação: <b style="color: #428BCA;"><?php echo $next; ?></b> <?php 
$days = 30-intval($days); if($days < 0) $days = 0; echo " - Falta(m) 
$days dia(s)"; ?></h2>
                        </div>

                        </div>
                </div>


        </div>
</div>






<div  class="content_row row vc_row wpb_row vc_row-fluid default-style 
fullwidth" style="position:relative;overflow:hidden;">
        <div class="rt-parallax-background" data-rt-parallax-
direction="1" data-rt-parallax-effect="horizontal" style="background-
image: url(images/portfolio_header.jpg);background-repeat: 
repeat;background-size: cover;background-position: right 
top;width:100%;height:100%;top:0;"></div>
        <div class="content_row_wrapper  default" style="padding-
top:25px;padding-bottom:15px;">
                <div class="vc_col-sm-12 wpb_column vc_column_container"
>
                        <div class="wpb_wrapper">
                                <div class="rt_heading_wrapper style-4">
                                        <h1 class="rt_heading  style-4"
>Política de privacidade</h1>
                                </div>
                        </div>
                </div>
        </div>
</div>

<div  class="content_row row vc_row wpb_row vc_row-fluid default-style 
fullwidth border_grid fixed_heights" >
        <div class="content_row_wrapper  default" style="padding-
top:50px;padding-bottom:50px; min-height: 500px;">
                <div class="vc_col-sm-12 wpb_column vc_column_container"
>
                        <div class="wpb_wrapper">
                                <div class="rt_heading_wrapper style-6">
                                        <h4 class="rt_heading  style-6"
><span class="icon-glyph-15 heading_icon"></span>POLÍTICA DE PRIVACIDADE 
DO SOPED</h4>
                                </div>
                                <div class="wpb_text_column 
wpb_content_element ">
                                        <div class="wpb_wrapper">
                                                <p>Última Atualização: 
Julho de 2017</b></p>
```

```
                                        </div>
                                </div>


                                <p>
Sua privacidade é algo importante para nós. Esta política de privacidade
explica quais dados pessoais coletamos e de que forma eles são usados.
Sugerimos e incentivamos que você leia atentamente ao nosso Termo de Uso.
<br/><br/>
<hr><br/>
<h2>Dados Pessoais que Coletamos</h2>
O SOPED é uma ferramenta oriunda de muito estudo para proporcionar à
você, usuário, condições para manter os seus pés saudáveis. Alguns desses
dados são fornecidos diretamente por você, por exemplo, quando você cria
a sua conta, passamos a ter registrado os seus dados cadastrais assim
como as avaliações feitas por você. Ainda é possível registrar e guardar
dados como desempenho na ferramenta e acompanhar a sua evolução.
Gostaríamos de deixar claro que mesmo que algum de seus dados seja
utilizado para pesquisa, a sua identidade será mantida em sigilo.
<br/><br/>

<hr/><br/>
<h2>Compartilhamos Dados Pessoais com outros sites?</h2>
Não! O SOPED é fruto de pesquisas realizadas no Laboratório de
Biomecânica da USP. Os dados recebidos só serão utilizados em pesquisas
dentro da própria instituição.<br/><br/>

<hr/><br/>
<h2>Como Acessar e Controlar seus Dados Pessoais?</h2>
Você pode visualizar, editar ou deletar seus dados pessoais. A sua
evolução e sugestões propostas nas avaliações e exercícios dependem
unicamente da sua sinceridade no preenchimento. Lembre-se, você controla
os seus dados. Para um bom desempenho e funcionamento da ferramenta seja
o mais honesto possível.  Ao aceitar a participar da ferramenta, você se
sujeita a aceitar os e-mails de notificação presentes para um bom
resultado no tratamento proposto. <br/><br/>
<hr/><br/>
<h2>Outras informações importantes:</h2><br/>
Para um funcionamento adequado do software, é necessário que o seu
navegador esteja atualizado (recomenados Google Chrome, firefox ou opera
em suas últimas versões), com javascript habilitado e suporte a html 5 e
css 3.

<hr/><br/>
<h2>Loja:</h2>
A Loja é um serviço para proporcionar interatividade a ferramenta, que
disponibiliza benefícios aos usuários a partir de sua dedicação nos
exercícios propostos. É importante salientar que ela é somente mais um
atrativo da ferramenta e que o uso dela não influencia no conteúdo já
disponível no software. <br/><br/>
<hr/>

<strong>Na eventualidade de um conflito entre uma política de
privacidade, os termos de uso e contrato entre o SOPED e o usuário. Favor
nos contactar.</strong>

                                </p>
                        </div>
                </div>
        </div>
</div>

<div  class="content_row row vc_row wpb_row vc_row-fluid default-style
fullwidth" style="position:relative;overflow:hidden;">
```

```
        <div class="rt-parallax-background" data-rt-parallax-
direction="1" data-rt-parallax-effect="horizontal" style="background-
image: url(images/portfolio_header.jpg);background-repeat:
repeat;background-size: cover;background-position: right
top;width:100%;height:100%;top:0;"></div>
        <div class="content_row_wrapper  default" style="padding-
top:25px;padding-bottom:15px;">
                <div class="vc_col-sm-12 wpb_column vc_column_container"
>
                        <div class="wpb_wrapper">
                                <div class="rt_heading_wrapper style-4">
                                        <h1 class="rt_heading  style-4"
>Cadastro SoPeD</h1>
                                </div>
                        </div>
                </div>
        </div>
</div>

<style type="text/css">
.default-style input[type="button"]:hover {background-color: #FC9D0D;}
.default-style input[type="button"] {background-color: #428bca; font-
weight: bold;}

.default-style input[type="button"]:hover.extra  {background-color:
#BBB;}
.default-style input[type="button"].extra  {background-color: #888; font-
weight: bold;}
</style>


<div  class="content_row row vc_row wpb_row vc_row-fluid default-style
fullwidth border_grid fixed_heights" >
        <div class="content_row_wrapper  default" style="padding-
top:50px;padding-bottom:50px; min-height: 1200px;">
                <div class="vc_col-sm-12 wpb_column vc_column_container"
>
                        <div class="wpb_wrapper">

                        <?php

        require_once('php/MysqlDB.php');

        require_once('php/email.php');

                                                $Obj = new ScriptDB();

                                                $Obj->Set('TableName',
'saedd_users');
                                                $Obj->Set('FieldsName',
'name|birthday|gender|cpf|email|pass|diabetic|insulin|medicines|year|canc
er|cellphone|homephone|state|city|address|origin|origin_desc|protocol|dis
eases|registration_date|status');
                                                $Obj-
>BeforeInsert("registration_date","NOW()");
                                                $Obj-
>BeforeInsert("status","'1'");


                                                $sql = $Obj-
>CreateInsert();


                                                $Obj->Connect();
                                                if($Obj-
>ExecQuery(($sql)))
                                                {
```

```
                                                        $sql = "INSERT
INTO saedd_quests(user_id,quest_datetime,quest) VALUES((SELECT
saedd_users.id FROM saedd_users WHERE email = '".$_POST['email']."' LIMIT
1),NOW(),'registration');";
                                                        $Obj-
>ExecQuery($sql);

                                                        echo
"<h2>Cadastro efetuado com sucesso!</div>";
                                                        echo
"<br/><br/><b>Verifique seu e-mail e seu lixo eletrônico.</b> Uma
mensagem foi enviada com as instruções de acesso ao sistema!<br/>Utilize
a senha e o email informados para acessar sua área de exercícios
personalizados e auto-avaliações.";

        SendPHPMail($_POST['email'],"","", "", "SoPeD - Confirmação de
Cadastro", "Prezado(a) <b>".$_POST['name']."</b><br/>Esse e-mail confirma
seu cadastro no SoPeD.<br/><br/>Utilize a senha e o email informados para
acessar sua área de exercícios personalizados e auto-avaliações, através
do menu <b>Iniciar Exercícios</b>.<br/><br/>Os dados de acesso
são:<br/><br/> <b>E-mail:</b> ".$_POST['email']."<br/><b>Senha:</b>
".$_POST['pass']."<br/><br/>Esperamos que o software promova uma melhora
significativa da saúde dos seus pés!<br/><br/><b>Obrigado por
experimentar o software!</b><br/>Equipe SoPeD");
                                                }
                                                else
                                                {
                                                        $Obj-
>ExecQuery("SELECT COUNT(*) FROM saedd_users WHERE email =
'".str_replace("'","''",$_POST['email'])."'");
                                                        if($Obj-
>GetQueryValue(0, 0) != 0)
                                                        {
                                                                echo
"<h2>Cadastro efetuado com sucesso!</div>";
                                                        echo
"<br/><br/><b>Verifique seu e-mail e seu lixo eletrônico.</b> Uma
mensagem foi enviada com as instruções de acesso ao sistema!<br/>Utilize
a senha e o email informados para acessar sua área de exercícios
personalizados e auto-avaliações.";
                                                        }
                                                        else
                                                        {
                                                          echo "<h2>Erro
ao efetuar a inscrição!</h2>";
                                                          echo
"<br/><br/><b>Entre em contato com o gestor do site pela <a
href='index.php?page=contato'>área de contato</a>.</b>";
                                                        }
                                                }

                                                $Obj->CloseDB();


                        ?>

                                        <br/><br/>

                                        <p><b>Sempre que quiser utilizar
a ferramenta clique em <a href='index.php?page=start'>INICIE AQUI</a> e
informe o email e a senha gerados durante o cadastro.</b></p>

                                        <div class="submit-button">
                                                <input class="button
submit" onclick="javascript:window.location = 'index.php?page=start' "
```

```
value="Clique aqui e informe seu email e senha para iniciar o uso da
ferramenta!" type="button"><span class="loading"></span>
                                        </div>

                        </div>
                </div>
        </div>
</div>


<div  class="content_row row vc_row wpb_row vc_row-fluid default-style
fullwidth" style="position:relative;overflow:hidden;">
        <div class="rt-parallax-background" data-rt-parallax-
direction="1" data-rt-parallax-effect="horizontal" style="background-
image: url(images/portfolio_header.jpg);background-repeat:
repeat;background-size: cover;background-position: right
top;width:100%;height:100%;top:0;"></div>
        <div class="content_row_wrapper  default" style="padding-
top:25px;padding-bottom:15px;">
                <div class="vc_col-sm-12 wpb_column vc_column_container"
>
                        <div class="wpb_wrapper">
                                <div class="rt_heading_wrapper style-4">
                                        <h1 class="rt_heading  style-4"
>Cadastre-se no SoPeD!</h1>
                                </div>
                        </div>
                </div>
        </div>
</div>
<link rel='stylesheet' href='css/myform.css' type='text/css' media='all'
/>

<style type="text/css">
.alt-style-1 input[type="button"]:hover {background-color: #FC9D0D;}
.alt-style-1 input[type="button"] {background-color: #428bca; font-
weight: bold;}

.alt-style-1 input[type="button"]:hover.extra  {background-color: #BBB;}
.alt-style-1 input[type="button"].extra  {background-color: #888; font-
weight: bold;}

#myform input[type="text"]:disabled {
    background: #aaa;
}
#myform span{color: #ff0000; font-size: 16px;}
</style>





        <script src="js/jquery.maskedinput.js"
type="text/javascript"></script>


        <script type='text/javascript'>
                jQuery(document).ready(function() {
                        jQuery('.phone').mask('(99) 9999-9999');
                        jQuery('.cellphone').mask('(99) 9 9999-9999');
                        jQuery('.date').mask('99/99/9999');
                        jQuery('.year').mask('9999');
                        jQuery('.cpf').mask('999.999.999-99');
                });
```

```
function validar_cpf(str)
{
        str=str.replace(".","");
        str=str.replace(".","");
        str=str.replace(".","");
        str=str.replace("-","");


        if (str.length != 11 || str == "00000000000" || str ==
"11111111111" || str == "22222222222" || str == "33333333333" || str ==
"44444444444" || str == "55555555555" || str == "66666666666" || str ==
"77777777777" || str == "88888888888" || str == "99999999999")
                return false;

        soma = 0;
        for (i=0; i < 9; i ++)
                soma += parseInt(str.charAt(i)) * (10 - i);

        resto = 11 - (soma % 11);
        if (resto == 10 || resto == 11)
                resto = 0;

        if (resto != parseInt(str.charAt(9)))
                return false;

        soma = 0;

        for (i = 0; i < 10; i ++)
                soma += parseInt(str.charAt(i)) * (11 - i);

        resto = 11 - (soma % 11);

        if (resto == 10 || resto == 11)
                resto = 0;

        if (resto != parseInt(str.charAt(10)))
                return false;

        return true;
}


        function CheckEmail(mail)

        {

                var er = new RegExp(/^[A-Za-z0-9_\-\.]+@[A-Za-z0-9_\-
\.]{2,}\.[A-Za-z0-9]{2,}(\.[A-Za-z0-9])?/);

                if(typeof(mail) == "string"){

                        if(er.test(mail)){ return true; }

                }else if(typeof(mail) == "object"){

                        if(er.test(mail.value)){

                                          return true;

                                     }

                }else{

                        return false;
```

```
                }

        }



                jQuery(document).ready(function()
                {
                        jQuery('#state').change(function(e){
                        jQuery.ajax({
                                        type: "POST",
                                        url: "php/admin/cidades.php",
                                        data: {estado:
jQuery('#state').val()}
                                })
                                .done(function(result)
                                {
                                        var option = result;

        jQuery('#city').html(option).show();
                                });
                        });
                });

                function ValidarData(data)
                {
                        var day   = parseInt(data.substring(0,2));
                        var month = parseInt(data.substring(3,5));
                        var year  = parseInt(data.substring(6,10));

                        if(month > 12 || month < 1 || day < 1 || year <
1900)
                        return false;

                        if((month == 1 || month == 3 || month == 5 ||
month == 7 || month == 8 || month == 10 || month == 12) && day > 31)
                                 return false;
                        else if((month == 4 || month == 6 || month == 9
|| month == 11) && day > 30)
                                 return false;
                        else if(month == 2)
                        {
                                if(!((year%4 == 0 && year%100 != 0) ||
year%400 == 0) && day > 29)
                                        return false;
                                else if(day > 28)
                                        return false;
                        }
                        return true;
                }

                function RegisterUser()
                {
                        jQuery("#error").html("");

                        if(jQuery("#name").val().length < 5 ||
jQuery("#name").val().indexOf(" ") == -1)
                        {
                                jQuery("#error").html("Informe seu nome
completo.");
                                return false;
                        }
```

```
			if(jQuery("#birthday").val().length != 10 ||
!ValidarData(jQuery("#birthday").val()))
			{
				jQuery("#error").html("Informe uma data
de nascimento válida.");
				return false;
			}

			if(!validar_cpf(jQuery("#cpf").val()))
			{
				jQuery("#error").html("Esse CPF é
inválido!");
				return false;
			}

			if(jQuery("#cellphone").val() == "")
			{
				jQuery("#error").html("Informe o número
do seu celular.");
				return false;
			}


			if(jQuery("#state").val().length == 0)
			{
				jQuery("#error").html("Informe o seu
estado de residência.");
				return false;
			}

			if(jQuery("#city").val().length == 0)
			{
				jQuery("#error").html("Informe a sua
cidade de residência.");
				return false;
			}

			if(jQuery("#address").val().length < 3)
			{
				jQuery("#error").html("Informe o seu
endereço.");
				return false;
			}


			if(!CheckEmail(jQuery("#email").val()) ||
jQuery("#email").val() == "")
			{
				jQuery("#error").html("Informe um email
válido.");
				return false;
			}

			if(jQuery("#pass").val().length < 3)
			{
				jQuery("#error").html("Informe uma senha
de pelo menos 3 dígitos.");
				return false;
			}


			if(jQuery("#pass").val() !=
jQuery("#pass2").val())
```

```
                        {
                                jQuery("#error").html("As senhas
informadas são diferentes.");
                                return false;
                        }

                        if(jQuery("#origin").prop("selectedIndex") > 2 &&
jQuery("#desc").val() == "")
                        {
                                jQuery("#error").html("Informe como
chegou ao SoPeD.");
                                return false;
                        }

                        if(jQuery("#diabetic").prop("selectedIndex") == 1
&& jQuery("#year").val() == "")
                        {
                                jQuery("#error").html("Qual o ano que
você descobriu ser portador de diabestes?");
                                return false;
                        }

                        if(jQuery("#cancerdis").prop("checked") &&
jQuery("#cancer").val() == "")
                        {
                                jQuery("#error").html("De qual Câncer
você já foi operado?");
                                return false;
                        }


                        var dis = "";
                        for(i=1; i<=12; i++)
                        {
                           if(jQuery("#dis"+i).prop("checked"))
                             dis += jQuery("#dis"+i).val()+"|";
                        }

                        dis = dis.substring(0, dis.length - 1);
                        jQuery("#diseases").val(dis);



                        document.myform.submit();
                }

        </script>


<div  class="content_row row vc_row wpb_row vc_row-fluid alt-style-1
fullwidth" >
        <div class="content_row_wrapper  default" >
                <div class="vc_col-sm-12 wpb_column vc_column_container"
>
                        <div class="wpb_wrapper">



                                        <form
action="index.php?page=registration" name="myform" id='myform'
class="rt_form" method="post" id="myform" style="margin-left: -15px;">



                                <div class="rt_heading_wrapper style-6">
```

```
                                        <h5 class="rt_heading  style-6"
>Dados Pessoais</h5>
                                </div>

                                                <div class="vc_col-sm-8
wpb_column vc_column_container" >
                                                        <label>Nome
completo <span>*</span></label>
                                                        <input
type="text" name="name" id='name' />
                                                </div>

                                                <div class="vc_col-sm-2
wpb_column vc_column_container" >
                                                        <label>Data de
Nascimento <span>*</span></label>
                                                        <input
type="text" class="date" name="birthday" id="birthday" />
                                                </div>
                                                <div class="vc_col-sm-2
wpb_column vc_column_container" >
                                                        <label>Sexo
<span>*</span></label>
                                                        <select size="1"
name="gender">
                                                                <option
value="M">Masculino</option>
                                                                <option
value="F">Feminino</option>
                                                        </select>
                                                </div>


                                                <div
class="clear"></div><br/>

                                                <div class="vc_col-sm-4
wpb_column vc_column_container" >
                                                        <label>CPF
<span>*</span></label>
                                                        <input
type="text" class="cpf" name="cpf" id="cpf" />
                                                </div>


                                                <div class="vc_col-sm-4
wpb_column vc_column_container" >
                                                        <label>Telefone
Celular <span>*</span></label>
                                                        <input
type="text" class="cellphone" name="cellphone" id="cellphone" />
                                                </div>

                                                <div class="vc_col-sm-4
wpb_column vc_column_container" >
                                                        <label>Telefone
Residencial</label>
```

```
                                                                <input
type="text" class="phone" name="homephone"  id="homephone" />
                                                        </div>

                                                        <div
class="clear"></div><br/>

                                                        <div class="vc_col-sm-4
wpb_column vc_column_container" >
                                                                <label>Estado
(UF) <span>*</span></label>
                                                                <select size="1"
name="state" id="state">
                                                                                        <option
                                                                                        <option
                                                                                        <option
                                                                                        <option
                                                                                        <option
                                                                                        <option
                                                                                        <option
                                                                                        <option
                                                                                        <option
                                                                                        <option
                                                                                        <option
                                                                                        <option
                                                                                        <option
                                                                                        <option
                                                                                        <option
                                                                                        <option
                                                                                        <option
                                                                                        <option
                                                                                        <option
                                                                                        <option
                                                                                        <option
                                                                                        <option
                                                                                        <option
                                                                                        <option
                                                                                        <option
                                                                                        <option
                                                                                        <option
                                                                                        <option
                                                                                        <option
                                                                        </select>
                                                        </div>

                                                        <div class="vc_col-sm-4
wpb_column vc_column_container" >
                                                                <label>Cidade
<span>*</span></label>
                                                                <select size="1"
name="city" id="city">
                                                                </select>
                                                        </div>

                                                        <div class="vc_col-sm-4
wpb_column vc_column_container" >
                                                                <label>Endereço
<span>*</span></label>
```

```
                                                        <input
type="text" name="address" id="address"  />
                                                </div>


                                                <div
class="clear"></div><br/><br/>


                                <div class="rt_heading_wrapper style-6">
                                        <h5 class="rt_heading  style-6"
>Dados para acesso / Pesquisa rápida</h5>
                                </div>

                                                <div class="vc_col-sm-4
wpb_column vc_column_container" >
                                                        <label>E-mail
<span>*</span></label>
                                                        <input
type="email" name="email" id="email" />
                                                </div>

                                                <div class="vc_col-sm-4
wpb_column vc_column_container" >
                                                        <label>Crie uma
senha para acessar o SoPeD <span>*</span></label>
                                                        <input
type="password" name="pass"  id="pass" />
                                                </div>

                                                <div class="vc_col-sm-4
wpb_column vc_column_container" >
                                                        <label>Redigite a
senha <span>*</span></label>
                                                        <input
type="password" name="pass2"  id="pass2" />
                                                </div>



                                                <div
class="clear"></div><br/>

                                                <div class="vc_col-sm-4
wpb_column vc_column_container" >
                                                        <label>Como
chegou ao SoPeD? <span>*</span></label>
                                                        <select size="1"
name="origin" id="origin" onchange="javascript:if(this.selectedIndex > 2)
jQuery('#origin_desc').prop( 'disabled', false); else{
jQuery('#origin_desc').prop( 'disabled', true);
jQuery('#origin_desc').val('');}">

        <option>Indicação de um amigo</option>

        <option>Indicação de um profissional da saúde</option>

        <option>Pesquisa no google</option>

        <option>Através de outro site...</option>
```

```
        <option>De algum material impresso...</option>

        <option>Outros...</option>
                                                        </select>
                                                </div>

                                                <div class="vc_col-sm-4
wpb_column vc_column_container" >
                                                        <label>Qual?
<span>*</span></label>
                                                        <input
type="text" name="origin_desc" disabled id="origin_desc" />
                                                </div>

                                                <div class="vc_col-sm-4
wpb_column vc_column_container" >
                                                        <label>Projeto /
Protocolo<span>*</span></label>
                                                        <select size="1"
name="protocol" id="protocol">

                                                                <?php

        require_once('php/MysqlDB.php');

        $Obj = new ScriptDB();

        $Obj->ExecQuery("SELECT id, title FROM saedd_projects ORDER BY
1");

        $count = $Obj->Get("RecordCount");

        for($i=0; $i<$count; $i++)

echo "<option value='".$Obj->GetQueryValue($i,0)."'>".$Obj-
>GetQueryValue($i,1)."</option>";
                                                                ?>

                                                        </select>
                                                </div>

                                                <div
class="clear"></div><br/><br/>

                                <div class="rt_heading_wrapper style-6">
                                        <h5 class="rt_heading  style-6"
>Informações sobre a saúde do usuário</h5>
                                </div>


                                                <div class="vc_col-sm-2
wpb_column vc_column_container" >
                                                        <label>Portador
de Diabetes? <span>*</span></label>
                                                        <select size="1"
name="diabetic" id="diabetic" onchange="javascript:if(this.selectedIndex
== 1) jQuery('#year').prop( 'disabled', false); else{
jQuery('#year').prop( 'disabled', true); jQuery('#year').val('');}">

        <option>Não</option>

        <option>Sim</option>
                                                        </select>
                                                </div>
```

```
                                                <div class="vc_col-sm-2
wpb_column vc_column_container" >
                                                        <label>Ano de
Diagnostico <span>*</span></label>
                                                        <input
type="text" class="year" name="year" disabled id="year" />
                                                </div>

                                                <div class="vc_col-sm-2
wpb_column vc_column_container" >
                                                        <label>Faz uso de
insulina? <span>*</span></label>
                                                        <select size="1"
name="insulin" id="insulin">
        <option>Não</option>
        <option>Sim</option>
                                                        </select>
                                                </div>

                                                <div class="vc_col-sm-2
wpb_column vc_column_container" >
                                                        <label>Usa
medicamentos? <span>*</span></label>
                                                        <select size="1"
name="medicines" id="diabetic">
                                                                <option
value='0'>Não</option>
                                                                <option
value='1'>Sim, 1 medicamento</option>
                                                                <option
value='2'>Sim, 2 medicamentos</option>
                                                                <option
value='3'>Sim, 3 medicamentos</option>
                                                                <option
value='4'>Sim, 4 medicamentos</option>
                                                                <option
value='5'>Sim, 5 medicamentos</option>
                                                                <option
value='6'>Sim, 6 medicamentos</option>
                                                                <option
value='7'>Sim, 7 medicamentos</option>
                                                                <option
value='8'>Sim, 8 medicamentos</option>
                                                                <option
value='9'>Sim, 9 medicamentos</option>
                                                                <option
value='10'>Sim, 10 ou mais</option>
                                                        </select>
                                                </div>


                                                <div class="vc_col-sm-4
wpb_column vc_column_container" >
                                                        <label
for="cancerdis"><input type='checkbox' id='cancerdis' value=''
onclick="javascript:if(this.checked) jQuery('#cancer').prop( 'disabled',
false); else{ jQuery('#cancer').prop( 'disabled', true);
jQuery('#cancer').val('');}" /> Câncer Operado? Onde?
<span>*</span></label>
                                                        <input
type="text" name="cancer" id="cancer" disabled />
                                                </div>
```

```
                                                <div
class="clear"></div><br/><br/>

                                <div class="rt_heading_wrapper style-6">
                                        <h5 class="rt_heading  style-6"
>É portador de outras doenças? Quais?</h5>
                                </div>



                        <style>

                        </style>


                                <div id="diseases2">
                                                <input type='hidden'
id='diseases' name='diseases' value='' />

                                                <div class="vc_col-sm-3
wpb_column vc_column_container" >
                                                        <label for="dis1"
data-toggle="tooltip"  title="É uma doença caracterizada pela elevação
dos níveis tensionais no sangue."><input type='checkbox' id='dis1'
value='Hipertensão' /> Hipertensão</label>

                                                </div>

                                                <div class="vc_col-sm-3
wpb_column vc_column_container" >
                                                        <label for="dis2"
data-toggle="tooltip" title="É um distúrbio nos níveis de lipídios e/ou
lipoproteínas no sangue."><input type='checkbox' id='dis2'
value='Dislipidemia'   /> Dislipidemia </label>
                                                </div>

                                                <div class="vc_col-sm-3
wpb_column vc_column_container" >
                                                        <label for="dis3"
data-toggle="tooltip" title="O glaucoma refere-se a um grupo de doenças
oculares que provocam danos irreparáveis no nervo óptico." ><input
type='checkbox' id='dis3' value='Glaucoma' /> Glaucoma </label>
                                                </div>

                                                <div class="vc_col-sm-3
wpb_column vc_column_container" >
                                                        <label for="dis4"
data-toggle="tooltip" title="Para pessoas que têm catarata tem a visão
nublada, como se olhassem por uma janela embaçada ou enevoada. Essa visão
nublada pode tornar mais difíceis tarefas como ler, dirigir um carro ou
interpretar a expressão das pessoas."><input type='checkbox' id='dis4'
value='Catarata' /> Catarata</label>
                                                </div>

                                                <div
class="clear"></div><br/>

                                                <div class="vc_col-sm-3
wpb_column vc_column_container" >
                                                        <label for="dis5"
data-toggle="tooltip" title="É uma doença óssea caracterizada pela baixa
```

```
regeneração do tecido esquelético, gerando ossos fracos e
quebradiços."><input type='checkbox' id='dis5' value='Osteoporose' />
Osteoporose </label>
                                                </div>

                                                <div class="vc_col-sm-3
wpb_column vc_column_container" >
                                                        <label for="dis6"
data-toggle="tooltip" title="É a condição em que a quantidade de
hormônios produzidos pela tireóide, em nosso organismo, está ABAIXO do
normal."><input type='checkbox' id='dis6' value='Hipotiroidismo' />
Hipotiroidismo</label>
                                                </div>

                                                <div class="vc_col-sm-3
wpb_column vc_column_container" >
                                                        <label for="dis7"
data-toggle="tooltip" title="É a condição em que a quantidade de
hormônios produzidos pela tireóide, em nosso organismo, está ACIMA do
normal."><input type='checkbox' id='dis7' value='Hipertiroidismo' />
Hipertiroidismo</label>
                                                </div>

                                                <div class="vc_col-sm-3
wpb_column vc_column_container" >
                                                        <label for="dis8"
data-toggle="tooltip" title="Inflamação de uma ou mais articulações que
causa dor e rigidez, sintomas que podem piorar com o
envelhecimento."><input type='checkbox' id='dis8' value='Artrite' />
Artrite </label>
                                                </div>



                                                <div
class="clear"></div><br/>


                                                <div class="vc_col-sm-3
wpb_column vc_column_container" >
                                                        <label for="dis9"
data-toggle="tooltip" title="Tipo de artrite que ocorre quando o tecido
flexível nas extremidades dos ossos se desgasta."><input type='checkbox'
id='dis9' value='Artrose' /> Artrose </label>
                                                </div>

                                                <div class="vc_col-sm-3
wpb_column vc_column_container" >
                                                        <label
for="dis10" data-toggle="tooltip" title="O acidente vascular cerebral, ou
derrame cerebral, ocorre quando há um entupimento ou o rompimento dos
vasos que levam sangue ao cérebro provocando a paralisia da área cerebral
que ficou sem circulação sanguínea adequada."><input type='checkbox'
id='dis10' value='AVC prévios ' /> AVC prévios </label>
                                                </div>

                                                <div class="vc_col-sm-3
wpb_column vc_column_container" >
                                                        <label
for="dis11" data-toggle="tooltip" title="Condições cardíacas que incluem
vasos doentes, problemas estruturais e coágulos sanguíneos."><input
type='checkbox' id='dis11' value='Doenças Cardíacas' /> Doenças
Cardíacas</label>
                                                </div>
```

```
                                                <div class="vc_col-sm-3
wpb_column vc_column_container" >
                                                        <label
for="dis12" data-toggle="tooltip" title="Distúrbio do humor que leva à
persistente sensação de tristeza e perda de interesse."><input
type='checkbox' id='dis12' value='Depressão' /> Depressão </label>
                                                </div>

                                        </div>



                                        </form>

                                        <div class="clear"></div><br/>

                                        <input type='checkbox'
id='mycheck' /> <label for='mycheck'>Li os <a href="index.php?page=terms"
target='_blank'>Termos do Cadastro com a opção de Treinamento</a> da
ferramenta.</label>

                                        <div class="clear"></div><br/>

                                        <b style='color: #FF0000;'
id='error'></b>

                                        <div class="submit-button"
style='text-align: center;'>
                                                <input class="button
submit" onclick="javascript:
if(!jQuery('#mycheck').prop('checked')){alert('Você precisa concordar com
os termos de uso do software.'); return false;} RegisterUser()"
value="Finalizar Cadastro" type="button"><span class="loading"></span>
                                        </div>

                                <div class="clear"></div>

                        </div>
                </div>
        </div>
</div>




<div  class="content_row row vc_row wpb_row vc_row-fluid default-style
fullwidth border_grid fixed_heights" >

        <div class="content_row_wrapper  default" style="padding-
top:40px;padding-bottom:40px;"><div class="vc_col-sm-4 wpb_column
vc_column_container vc_custom_1421091415717" >
                <div class="wpb_wrapper">
                        <article  class="icon-content-box content-box
icon-top icon-style-3"><div class="icon-holder"><a
href="index.php?page=start" title="" target="_self"><span class="icon-
new-link-1"></span></a></div><div class="text-holder">
        <h4 class="heading">
                <a href="index.php?page=start" title="Box Heading"
target="_self">
                        Por que utilizar?
                </a>
        </h4> <p class="aligncenter">Além de ser fácil e gratuito a
ferramenta se ajusta aos horários do paciente e promove uma melhora do
bem estar dos pés.</p>
```

```
</div></article>
                </div>
        </div>

        <div class="vc_col-sm-4 wpb_column vc_column_container" >
                <div class="wpb_wrapper">
                        <article  class="icon-content-box content-box
icon-top icon-style-3"><div class="icon-holder"><a
href="index.php?page=perguntar" title="" target="_self"><span
class="icon-new-user-1"></span></a></div><div class="text-holder">
        <h4 class="heading">
                <a href="index.php?page=perguntar" title="Box Heading"
target="_self">
                        Fale com um Especialista!
                </a>
        </h4> <p class="aligncenter">Em caso de dúvidas consulte nossos
especialistas online. Estamos sempre prontos para atendê-lo!</p>
</div></article>
                </div>
        </div>

        <div class="vc_col-sm-4 wpb_column vc_column_container" >
                <div class="wpb_wrapper">
                        <article  class="icon-content-box content-box
icon-top icon-style-3"><div class="icon-holder"><a
href="index.php?page=compartilhar2" title="" target="_self"><span
class="icon-new-heart-1"></span></a></div><div class="text-holder">
        <h4 class="heading">
                <a href="index.php?page=compartilhar2" title="Box
Heading" target="_self">
                        Gostou? Compartilhe!
                </a>
        </h4> <p class="aligncenter">Indique o SoPeD para amigos e
pessoas da sua rede de contato. Você estará ajudando a melhorar a
ferramenta.</p>
</div></article>
                </div>
        </div>
</div>
</div>




<div  class="content_row row vc_row wpb_row vc_row-fluid default-style
fullwidth" style="position:relative;overflow:hidden;">
        <div class="rt-parallax-background" data-rt-parallax-
direction="1" data-rt-parallax-effect="horizontal" style="background-
image: url(images/portfolio_header.jpg);background-repeat:
repeat;background-size: cover;background-position: right
top;width:100%;height:100%;top:0;"></div>
        <div class="content_row_wrapper  default" style="padding-
top:25px;padding-bottom:15px;">
                <div class="vc_col-sm-12 wpb_column vc_column_container"
>
                        <div class="wpb_wrapper">
                                <div class="rt_heading_wrapper style-4">
                                        <h1 class="rt_heading  style-4"
>Sobre a polineuropatia</h1>
                                </div>
                        </div>
                </div>
        </div>
</div>
```

```html
<div  class="content_row row vc_row wpb_row vc_row-fluid default-style
fullwidth border_grid fixed_heights" >
        <div class="content_row_wrapper  default" style="padding-
top:50px;padding-bottom:50px; min-height: 500px;">
                <div class="vc_col-sm-12 wpb_column vc_column_container"
>
                        <div class="wpb_wrapper">
                                <div class="rt_heading_wrapper style-6">
                                        <h4 class="rt_heading  style-6"
><span class="icon-glyph-15 heading_icon"></span>Conheça mais sobre o pé
diabético</h4>
                                </div>
                                <div class="wpb_text_column
wpb_content_element ">
                                        <div class="wpb_wrapper">
                                                <p>Gostaria de saber um
pouco mais sobre o pé diabético? <b>Clique nas abas para detalhar as
informações.</b></p>
                                        </div>
                                </div>
                                <div id="Unique-ID" class="rt-toggle CSS-
Class-Name">
                                        <ol>
                                                <li  class="">
                                                        <div
class="toggle-head">
                                                                <div
class="toggle-number">1</div>
                                                                <div
class="toggle-title">O que é polineuropatia?</div>
                                                        </div>
                                                        <div
class="toggle-content">
                                                                <div
class="wpb_text_column wpb_content_element ">

        <div class="wpb_wrapper">
                                                                                <p>A pol
para a qualidade de vida dos pacientes, já que o desfecho mais devastador
da doença são as úlceras plantares e amputações, quando não tratada
adequadamente. Dados recentes da Federação Internacional de Diabetes (8ª
ed. 2017) mostram que a cada 30 segundos, uma pessoa em algum lugar no
mundo sofre uma amputação em membro inferior como consequência do
diabetes. Esses dados reforçam ainda mais a necessidade da prevenção e
cuidados contínuos para evitar estes agravos. A polineuropatia gera
perdas progressivas de sensibilidade vibratória, térmica, tátil e
proprioceptiva.</p>

        </div>
                                                                </div>
                                                        </div>
                                                </li>

                                                <li  class="">
                                                        <div
class="toggle-head">
                                                                <div
class="toggle-number">3</div>
                                                                <div
class="toggle-title">Causas e sintomas da polineuropatia</div>
                                                        </div>
                                                        <div
class="toggle-content">
                                                                <div
class="wpb_text_column wpb_content_element ">
```

```
        <div class="wpb_wrapper">
                                                                                            <p>Os si
extremidades), problemas musculares (cãimbras, fraqueza), perda
progressiva de sensibilidade vibratória, térmica, tátil e
propriocepção.</p>

        </div>
                                                                            </div>
                                                                    </div>
                                                            </li>

                                                            <li  class="">
                                                                    <div
class="toggle-head">
                                                                            <div
class="toggle-number">4</div>
                                                                            <div
class="toggle-title">Tratamento da polineuropatia</div>
                                                                    </div>
                                                                    <div
class="toggle-content">
                                                                            <div
class="wpb_text_column wpb_content_element ">

        <div class="wpb_wrapper">
                                                                                            <p>O tra
exercícios para pés e tornozelos.</p>

        </div>
                                                                            </div>
                                                                    </div>
                                                            </li>

                                                            <li  class="">
                                                                    <div
class="toggle-head">
                                                                            <div
class="toggle-number">5</div>
                                                                            <div
class="toggle-title">Como o SoPeD pode lhe ajudar?</div>
                                                                    </div>
                                                                    <div
class="toggle-content">
                                                                            <div
class="wpb_text_column wpb_content_element ">

        <div class="wpb_wrapper">
                                                                                            <p>O Sof
incluindo uma série de exercícios personalizados de fácil realização em
um ambiente de escolha do indivíduo, sem a necessidade constante de um
profissional por perto. Esses exercícios geram uma intervenção precoce
nas principais causas de formação de úlcera e proporciona então uma
melhor qualidade de vida da pessoa com diabetes.<br/><br/><b
style='color: #CC2233;'>Atenção: Este software não exclui o
acompanhamento de um especialista.</b></p>

        </div>
                                                                            </div>
                                                                    </div>
                                                            </li>
                                                    </ol>
                                            </div>
                                    </div>
                            </div>
```

```
        </div>
</div>
<div  class="content_row row vc_row wpb_row vc_row-fluid alt-style-1
fullwidth border_grid fixed_heights" >
        <div class="content_row_wrapper  default" style="padding-
top:40px;padding-bottom:40px;">
                <div class="vc_col-sm-4 wpb_column vc_column_container
vc_custom_1421091415717" >
                        <div class="wpb_wrapper">
                                <article  class="icon-content-box
content-box icon-top icon-style-3">
                                        <div class="icon-holder"><a
href="#" title="" target="_self"><span class="icon-new-link-
1"></span></a></div>
                                        <div class="text-holder">
                                                <h4 class="heading">
                                                        <a href="#"
title="Box Heading" target="_self">
                                                        Por que utilizar?
                                                        </a>
                                                </h4>
                                                <p
class="aligncenter">Além de ser fácil e gratuito a ferramenta se ajustar
aos horários do paciente e promove uma percepível melhora do bem estar
dos pés.</p>
                                        </div>
                                </article>
                        </div>
                </div>
                <div class="vc_col-sm-4 wpb_column vc_column_container" >
                        <div class="wpb_wrapper">
                                <article  class="icon-content-box
content-box icon-top icon-style-3">
                                        <div class="icon-holder"><a
href="#" title="" target="_self"><span class="icon-new-user-
1"></span></a></div>
                                        <div class="text-holder">
                                                <h4 class="heading">
                                                        <a href="#"
title="Box Heading" target="_self">
                                                        Fale com o
Doutor!
                                                        </a>
                                                </h4>
                                                <p class="aligncenter">Em
caso de dúvidas consulte nossos especialistas online. Estamos sempre
pronto para atendê-lo!</p>
                                        </div>
                                </article>
                        </div>
                </div>
                <div class="vc_col-sm-4 wpb_column vc_column_container" >
                        <div class="wpb_wrapper">
                                <article  class="icon-content-box
content-box icon-top icon-style-3">
                                        <div class="icon-holder"><a
href="#" title="" target="_self"><span class="icon-new-heart-
1"></span></a></div>
                                        <div class="text-holder">
                                                <h4 class="heading">
                                                        <a href="#"
title="Box Heading" target="_self">
                                                        Gostou?
Compartilhe!
                                                        </a>
```

```
                                                </h4>
                                                <p
class="aligncenter">Indique o SoPeD para amigos e pessoas do sua rede de
contato. Você estará ajudando a melhorar a ferramenta.</p>
                                        </div>
                                </article>
                        </div>
                </div>
        </div>
</div>
<div  class="content_row row vc_row wpb_row vc_row-fluid default-style
fullwidth" style="position:relative;overflow:hidden;">
        <div class="rt-parallax-background" data-rt-parallax-
direction="1" data-rt-parallax-effect="horizontal" style="background-
image: url(images/portfolio_header.jpg);background-repeat:
repeat;background-size: cover;background-position: right
top;width:100%;height:100%;top:0;"></div>
        <div class="content_row_wrapper  default" style="padding-
top:25px;padding-bottom:15px;">
                <div class="vc_col-sm-12 wpb_column vc_column_container"
>
                        <div class="wpb_wrapper">
                                <div class="rt_heading_wrapper style-4">
                                        <h1 class="rt_heading  style-4"
>Compartilhamento o SoPed</h1>
                                </div>
                        </div>
                </div>
        </div>
</div>




        <style>
                .icircle img {-webkit-border-radius: 124px;
                        -moz-border-radius: 124px;
                        border-radius: 124px;
                        width: 120px;}
                .wpb_wrapper input[type="button"] {background: #428BCA;}
                .wpb_wrapper input[type="button"]:hover {background:
#317AB9;}
        </style>

<div  class="content_row row vc_row wpb_row vc_row-fluid alt-style-1
fullwidth border_grid fixed_heights" >
        <div class="content_row_wrapper  default" style="padding-
top:50px;padding-bottom:50px; min-height: 500px;">
                <div class="vc_col-sm-12 wpb_column vc_column_container"
>
                        <div class="wpb_wrapper">

                                                <div class="icircle">


                                        <div
class="addthis_inline_share_toolbox" ></div>

                                        <script type="text/javascript"
src="//s7.addthis.com/js/300/addthis_widget.js#pubid=ra-
58fa5601eaa0c0e1"></script>



    </script>
```

```
                        </div>
                </div>
        </div>
</div>

<div  class="content_row row vc_row wpb_row vc_row-fluid default-style
fullwidth" style="position:relative;overflow:hidden;">
        <div class="rt-parallax-background" data-rt-parallax-
direction="1" data-rt-parallax-effect="horizontal" style="background-
image: url(images/portfolio_header.jpg);background-repeat:
repeat;background-size: cover;background-position: right
top;width:100%;height:100%;top:0;"></div>
        <div class="content_row_wrapper  default" style="padding-
top:25px;padding-bottom:15px;">
                <div class="vc_col-sm-12 wpb_column vc_column_container"
>
                        <div class="wpb_wrapper">
                                <div class="rt_heading_wrapper style-4">
                                        <h1 class="rt_heading  style-4"
>Notícias SoPeD</h1>
                                </div>
                        </div>
                </div>
        </div>
</div>



<div  class="content_row row vc_row wpb_row vc_row-fluid default-style
fullwidth overlap" >

        <div class="content_row_wrapper  default" >
        <div class="vc_col-sm-12 wpb_column vc_column_container" >
                <div class="wpb_wrapper" style="min-height: 700px;">



<article class="loop post-2505 post type-post status-publish format-
standard has-post-thumbnail hentry category-post-types category-standart-
posts" id="post-2505">



        <section class="featured_image featured_media">


                                <a href="" title="Standard Post Type"
class="featured_image imgeffect link"><img
src="files/noticias/noticias.jpg" alt="" class="" /></a>


        </section>


        <section class="date">19 de Abril de 2016</section><br/>
        <section class="text">

                <h2><a href="" rel="bookmark">Cuidados com o pé
diabético: novos achados da ciência</a></h2>

                <div class="post_data" style="font-size: 11px;">
```

 Administrador SoPeD

Post Types, Saúde | Diabetes

8 Visualizações

</div>

<p>

• Examine seus pés todos os dias: preste atenção em mudanças de coloração e temperatura; olhe cada um dos dedos e entre eles; procure por infecções, cortes, bolhas, calos, feridas. Se não conseguir fazer isso sozinho, peça ajuda a um familiar ou amigo, use um espelho para olhar a sola dos pés.

• Para a higienização dos pés, use água morna. Nunca verifique a temperatura da água diretamente com os pés. Faça isso com o cotovelo. Deixe a água cair devagar, passe o sabonete delicadamente e enxágue. Na hora de secar, não esfregue, enxugue entre os dedos, aplique creme hidratante ou óleo sobre a pele do dorso, nunca entre os dedos, na sola dos pés e em feridas e cortes. Evite talco, pois pode causar ressecamento da pele.

• Aproveite para cortar as unhas após o banho, pois elas estarão amolecidas. Faça isso em um local com boa iluminação. Se não puder ver as unhas ou apresentar problemas de sensibilidade nos pés, peça alguém para fazer por você. Dê preferência a cortadores de unhas ou a tesouras com pontas redondas, pois a possibilidade de se machucar diminui bastante. Corte as unhas retas, nunca corte os cantos das unhas, assim você evita a unha encravada e muitas infecções. Não use objetos afiados e pontiagudos, pois eles podem machucar. Não corte calos com gilete, no máximo use lixas próprias. Consulte um podólogo para tratar os calos e consulte um médico em caso de micose nas unhas e entre os dedos.

• Nunca ande descalço! Lembre-se que pessoas com diabetes pode apresentar diminuição da sensibilidade dos pés, o que facilita o trauma e o aparecimento de feridas e infecções indesejadas.

• Na hora de escolher o calçado, vá sempre na parte da tarde, que é quando o pé está do tamanho certo e não vai ficar apertado depois. O uso do calçado de tamanho adequado, evita calos, bolhas, joanetes, feridas e infecções. Procure sapatos macios, sem costuras, sem bico fino ou com salto muito alto; evite sandálias que tenham tiras ou fivelas.

• Em relação às meias, prefira aquelas que deixam a pele dos pés respirarem. Troque as meias quando sentir que estão molhadas de suor. Prefira aquelas de algodão e que não tenham costuras. Evite as meias apertadas e as meias de nylon.

• Caminhar é a vocação dos pés e seu melhor exercício! Procure caminhar com um calçado adequado e confortável e em superfície plana. Evite caminhar se apresentar feridas ou dor e procure o seu médico nesse caso.

```
    <br/><br/>&bull; Evite cruzar as pernas quando estiver sentado, pois
piora a circulação do sangue nos pés. Se for viajar, evite ficar sentado
por muito tempo. Levante-se, ande um pouco. Se isso não for possível,
estique os pés, movimente-os para cima, para baixo e para os lados, mova
os dedos, faça círculos com os pés. Hidrate-se adequadamente durante as
viagens, isso evita desidratação e embolias.

    <br/><br/>&bull; Quando estiver sentado use um banquinho para manter
os pés elevados, isso auxilia o retorno do sangue, fazendo com que as
toxinas sejam eliminadas mais rapidamente.

    <br/><br/>&bull; Lembre-se também que tudo isso funcionará muito
melhor se estiver acompanhado de um bom controle glicêmico!!! O cuidado
com os pés começa com o uso adequado da medicação e com o cumprimento da
dieta alimentar adequada para cada pessoa.
.</p>




        </section>

</article>


</div>
        </div>

</div>
</div>



<?php include("internal.php"); ?>
<!--
<div  class="content_row row vc_row wpb_row vc_row-fluid  alt-style-1
fullwidth border_grid fixed_heights" >
        <div class="content_row_wrapper  default" style="padding-
top:0px;padding-bottom:20px; margin-bottom: 30px;">

                <div class="vc_col-sm-2 wpb_column vc_column_container
vc_custom_1421091415717" >
                        <div class="wpb_wrapper">
                                <article  class="icon-content-box
content-box icon-top icon-style-3">
                                        <div class="icon-holder"><a
href="index.php?page=user_start" title="" target="_self"><img
src="images/icons/back.png" style='width: 64px;' /></a></div>
                                        <div class="text-holder">
                                                <b class="heading">
                                                        <a
href="index.php?page=user_start" title="Box Heading" target="_self">
                                                        Voltar para menu
                                                        </a>
                                                </b>

                                        </div>
                                </article>
                        </div>
                </div>

        </div>
</div>
-->
```

```
<div  class="content_row row vc_row wpb_row vc_row-fluid default-style
fullwidth" style="position:relative;overflow:hidden;">
        <div class="rt-parallax-background" data-rt-parallax-
direction="1" data-rt-parallax-effect="horizontal" style="background-
image: url(images/portfolio_header.jpg);background-repeat:
repeat;background-size: cover;background-position: right
top;width:100%;height:100%;top:0;"></div>
        <div class="content_row_wrapper  default" style="padding-
top:25px;padding-bottom:15px;">
                <div class="vc_col-sm-12 wpb_column vc_column_container"
>
                        <div class="wpb_wrapper">
                                <div class="rt_heading_wrapper style-4">
                                        <h1 class="rt_heading  style-4"
>Colaboradores SoPeD</h1>
                                </div>
                        </div>
                </div>
        </div>
</div>





<div  class="content_row row vc_row wpb_row vc_row-fluid default-style
fullwidth" >
        <div class="content_row_wrapper  default" >
                <div class="vc_col-sm-12 wpb_column vc_column_container"
>
                        <div class="wpb_wrapper">
                                <section id="staff-700135" class="team
border_grid clearfix  " style="min-height: 700px;">
                                        <h2>Conheça nossos
especialistas</h2>
                                        <div class="row clearfix">
                                                <div class="col col-sm-
4">
                                                        <div class="loop
post-1873 staff type-staff status-publish has-post-thumbnail hentry"
id="person-1873">
                                                                <div
class="person_image">

        <div class="person_image_wrapper">
                                                                                                <a href=

        </div>
                                                                </div>
                                                                <h5
class="person_name"><a href="">Nutricionista</a></h5>
                                                                <span
class="position">Colaborador</span>
                                                                <div
class="profile">

                                                                </div>
                                                        </div>
                                                </div>

                                                <div class="col col-sm-
4">
```

```
                                                        <div class="loop
post-1873 staff type-staff status-publish has-post-thumbnail hentry"
id="person-1873">
                                                                <div
class="person_image">

        <div class="person_image_wrapper">
                                                                                <a href=

        </div>
                                                                </div>
                                                                <h5
class="person_name"><a href="">Endocrinologista</a></h5>
                                                                <span
class="position">Colaborador</span>
                                                                <div
class="profile">

                                                                </div>
                                                        </div>
                                                </div>


                                                <div class="col col-sm-
4">
                                                        <div class="loop
post-1873 staff type-staff status-publish has-post-thumbnail hentry"
id="person-1873">
                                                                <div
class="person_image">

        <div class="person_image_wrapper">
                                                                                <a href=

        </div>
                                                                </div>
                                                                <h5
class="person_name"><a href="">Fisioterapeuta</a></h5>
                                                                <span
class="position">Colaborador</span>
                                                                <div
class="profile">

                                                                </div>
                                                        </div>
                                                </div>



                                        </div>
                                </section>
                        </div>
                </div>
        </div>
</div>




<div  class="content_row row vc_row wpb_row vc_row-fluid default-style
fullwidth" style="position:relative;overflow:hidden;">
        <div class="rt-parallax-background" data-rt-parallax-
direction="1" data-rt-parallax-effect="horizontal" style="background-
image: url(images/portfolio_header.jpg);background-repeat:
```

```
repeat;background-size: cover;background-position: right
top;width:100%;height:100%;top:0;"></div>
        <div class="content_row_wrapper  default" style="padding-
top:25px;padding-bottom:15px;">
                <div class="vc_col-sm-12 wpb_column vc_column_container"
>
                        <div class="wpb_wrapper">
                                <div class="rt_heading_wrapper style-4">
                                        <h1 class="rt_heading  style-4"
>Equipe SoPeD</h1>
                                </div>
                        </div>
                </div>
        </div>
</div>


<div  class="content_row row vc_row wpb_row vc_row-fluid default-style
fullwidth" >
        <div class="content_row_wrapper  default" >
                <div class="vc_col-sm-12 wpb_column vc_column_container"
>
                        <div class="wpb_wrapper">
                                <section id="staff-241017" class="team
border_grid clearfix  ">
                                        <div class="row clearfix">
                                                <div class="col col-sm-
4">
                                                        <div class="loop
post-1872 staff type-staff status-publish has-post-thumbnail hentry"
id="person-1872">
                                                                <div
class="person_image">

        <div class="person_image_wrapper">
                                                                                               <a href=

        </div>
                                                                </div>
                                                                <h5
class="person_name"><a href="http://lattes.cnpq.br/9500627847688925"
target="_blank">Isabel de Camargo Neves Sacco </a></h5>
                                                                <span
class="position">Profissional de Educação Física</span>
                                                                <div
class="profile">

        Professora da Faculdade de Medicina da Universidade de São Paulo.
Os temas de seus trabalhos finais de graduação e pós-graduação foram
focados na neuropatia diabética.
                                                                </div>
                                                                <div
class="person_links_wrapper">

        <a href="http://lattes.cnpq.br/9500627847688925"
target="_blank"><img src="images/lattes.png" /></a>
                                                                </div>
                                                        </div>
                                                </div>

                                                <div class="col col-sm-
4">
                                                        <div class="loop
post-1872 staff type-staff status-publish has-post-thumbnail hentry"
id="person-1872">
```

```
                                                                    <div
class="person_image">

        <div class="person_image_wrapper">
                                                                                <a href=

        </div>
                                                                    </div>
                                                                    <h5
class="person_name"><a href="http://lattes.cnpq.br/8085566363801137"
target="_blank">Cristina D. Sartor</a></h5>
                                                                    <span
class="position">Fisioterapeuta</span>
                                                                    <div
class="profile">

        Doutora em Ciências da Reabilitação pela Universidade de São
Paulo (FMUSP). Tem experiência na investigação clínica e biomecânica de
disfunções musculoesqueléticas e do movimento humano.
                                                                    </div>
                                                                    <div
class="person_links_wrapper">

        <a href="http://lattes.cnpq.br/8085566363801137"
target="_blank"><img src="images/lattes.png" /></a>
                                                                    </div>
                                                            </div>
                                                    </div>



                                                    <div class="col col-sm-
4">
                                                            <div class="loop
post-1872 staff type-staff status-publish has-post-thumbnail hentry"
id="person-1872">
                                                                    <div
class="person_image">

        <div class="person_image_wrapper">
                                                                                <a href=

        </div>
                                                                    </div>
                                                                    <h5
class="person_name"><a href="http://lattes.cnpq.br/8900442529085071"
target="_blank">Jane Suelen S. P. Ferreira </a></h5>
                                                                    <span
class="position">Fisioterapeuta</span>
                                                                    <div
class="profile" style="height: 130px;">

        Fisioterapia,  Mestranda do Programa de Ciências da Reabilitação
da Faculdade de Medicina da USP e integrante do Laboratório de
Biomecânica do Movimento e Postura Humana.
                                                                    </div>
                                                                    <div
class="person_links_wrapper">

        <a href="http://lattes.cnpq.br/8900442529085071"
target="_blank"><img src="images/lattes.png" /></a>
                                                                    </div>
                                                            </div>
                                                    </div>
```

```
                                        </div>
                                </section>
                        </div>
                </div>
        </div>
</div>


<div  class="content_row row vc_row wpb_row vc_row-fluid alt-style-1
fullwidth" >
        <div class="content_row_wrapper  default" >
                <div class="vc_col-sm-12 wpb_column vc_column_container"
>
                        <div class="wpb_wrapper">
                                <section id="staff-700135" class="team
border_grid clearfix  ">
                                        <h2>Conheça nossos
especialistas</h2>
                                        <div class="row clearfix">
                                                <div class="col col-sm-
4">
                                                        <div class="loop
post-1873 staff type-staff status-publish has-post-thumbnail hentry"
id="person-1873">
                                                                <div
class="person_image">

        <div class="person_image_wrapper">
                                                                                <a href=

        </div>
                                                                </div>
                                                                <h5
class="person_name"><a href="">Nutricionista</a></h5>
                                                                <span
class="position">Colaborador</span>
                                                                <div
class="profile">

                                                                </div>
                                                        </div>
                                                </div>

                                                <div class="col col-sm-
4">
                                                        <div class="loop
post-1873 staff type-staff status-publish has-post-thumbnail hentry"
id="person-1873">
                                                                <div
class="person_image">

        <div class="person_image_wrapper">
                                                                                <a href=

        </div>
                                                                </div>
                                                                <h5
class="person_name"><a href="">Endocrinologista</a></h5>
                                                                <span
class="position">Colaborador</span>
                                                                <div
class="profile">

                                                                </div>
```

```
                                                                </div>
                                                        </div>


                                                        <div class="col col-sm-
4">
                                                                <div class="loop
post-1873 staff type-staff status-publish has-post-thumbnail hentry"
id="person-1873">
                                                                        <div
class="person_image">

        <div class="person_image_wrapper">
                                                                                                <a href=

        </div>
                                                                        </div>
                                                                        <h5
class="person_name"><a href="">Fisioterapeuta</a></h5>
                                                                        <span
class="position">Colaborador</span>
                                                                        <div
class="profile">

                                                                        </div>
                                                                </div>
                                                        </div>



                                                </div>
                                        </section>
                                </div>
                        </div>
                </div>
</div>


<?php
        if(isset($_POST['action']))
        {
                $action = $_POST['action'];

                if($action == "question")
                {

        require_once('../MysqlDB.php');
                                                        $Obj = new ScriptDB();

                                                        $Obj->Set("TableName",
"saedd_questions");
                                                        $Obj->Set("FieldsName",
"question|origin|attach|expert_id|user_id|registration_date");
                                                        $Obj-
>BeforeInsert("registration_date","NOW()");

                                                        $sql = $Obj-
>CreateInsert();

                                                        $Obj->Connect();

                                                        if($Obj->ExecQuery($sql))
                                                        {
                                                                echo "1";
```

```
                                                            }
                                                            else
                                                            {
                                                                    echo $Obj-
>GetLastError();
                                                            }

                                                            $Obj->CloseDB();


                        }
                        else if($action == "answer")
                        {

        require_once('../MysqlDB.php');
                                                            $Obj = new ScriptDB();

                                                            $Obj->Set("TableName",
"saedd_questions");
                                                            $Obj->Set("FieldsName",
"question|origin|attach|expert_id|user_id|registration_date");
                                                            $Obj-
>BeforeInsert("registration_date","NOW()");

                                                            $sql = $Obj-
>CreateInsert();

                                                            $Obj->Connect();
                                                            if($Obj->ExecQuery($sql))
                                                            {
                                                                    echo "1";
                                                            }
                                                            else
                                                            {
                                                                    echo $Obj-
>GetLastError();
                                                            }

                                                            $Obj->CloseDB();


                        }
                        return;
        }
?>

<?php include("php/pages/back.php"); ?>

<link rel='stylesheet' href='css/myform.css' type='text/css' media='all'
/>

<style type="text/css">
#myform input[type="text"],
#myform input[type="date"],
#myform input[type="datetime"],
#myform input[type="email"],
#myform input[type="number"],
#myform input[type="search"],
#myform input[type="time"],
#myform input[type="url"],
#myform input[type="password"],
#myform textarea,
#myform select {

    border-top: 1px solid #f0f0f0;
```

```
    border-left: 1px solid #f0f0f0;
    border-bottom: 1px solid #FAFAFA;
    border-right: 1px solid #FAFAFA;
    background: #e0e0e0;
}

.default-style input[type="button"]:hover {background-color: #FC9D0D;}
.default-style input[type="button"] {background-color: #428bca; font-
weight: bold;}

.default-style input[type="button"]:hover.extra  {background-color:
#BBB;}
.default-style input[type="button"].extra  {background-color: #888; font-
weight: bold;}

</style>




<script type="text/javascript">

        function ValidateQuestion()
        {
           jQuery("#msg").html("");

           if(jQuery.trim(jQuery("#question").val()) == "")
           {
                jQuery("#msg").html("<b style='color: #cc2233;'>Digite a
pergunta que será feita ao especialista.</b>");
                return;
           }

           jQuery("#msg").html("Aguarde...");

           jQuery.ajax({
                type: "POST",
                url: "php/pages/questions.php",
                data: {question : jQuery("#question").val(), origin : 0,
attach :  jQuery("#attach").val(), user_id : <?php echo $_SESSION['id'];
?>, action : "question"}
           })
           .done(function(html) {
                if(html == "1")
                {
                        var result = "";
                        jQuery("#question").val("");
                        jQuery("#msg").html(result);

                        window.location = 'index.php?page=questions';

                }
                else
                  jQuery("#msg").html("Erro: "+html);
           });

        }


function openInNewTab(url) {
  var win = window.open(url, '_blank');
  win.focus();
}

</script>
```

```
<div  class="content_row default-style no-composer overlap fullwidth">
        <div class="content_row_wrapper clearfix " style='min-height:
800px;'>
                <div class="col col-sm-12 col-xs-12 ">
                        <div class="single post-1872 staff type-staff
status-publish has-post-thumbnail hentry" id="person-1872">




                                        <form
action="index.php?page=registration" name="myform" id='myform'
class="validate_form rt_form" method="post" id="myform" style="margin-
left: -15px;">


                                                <div
class="rt_heading_wrapper style-6">
                                                        <h5
class="rt_heading  style-6" >Esqueceu as regras do exercício?</h5>
                                                </div>

                                                <input class="button"
value="Clique aqui para repassar as regras dos exercícios"
onclick="javascript:openInNewTab('index.php?page=introducao');"
style="background: #428bca;" type="button">
                                                <div class="clear
clearfix"></div><br/><br/>




                                                <div
class="rt_heading_wrapper style-6">
                                                        <h5
class="rt_heading  style-6" >Gostaria de rever o tutorial de
introdução?</h5>
                                                </div>

                                                <input class="button"
value="Clique aqui para exibir o tutorial"
onclick="javascript:window.location =
'index.php?page=user_start&tutorial=1';" style="background: #428bca;"
type="button">
                                                <div class="clear
clearfix"></div><br/><br/>




                                                <div
class="rt_heading_wrapper style-6">
                                                        <h5
class="rt_heading  style-6" >Está com dúvidas? Pergunte aos
especialistas!</h5>
                                                </div>


                                                <div class="vc_col-sm-12
wpb_column vc_column_container" >
```

```
        <label>Pergunta</label>
                                                        <textarea
id='question' name='question' style='width: 100%; height: 120px;'
placeholder="Digite aqui sua pergunta"></textarea>


                                                        <br/><a
href="javascript:void(0);" id='upbt'>Anexar arquivo à pergunta...</a>

                                                        <div
class="clear"></div><br/>
                                                        <div
id='msg'></div>
                                                        <br/>
                                                        <input
class="button submit" onclick="javascript:ValidateQuestion();"
value="Enviar pergunta" type="button" />

                                                </div>

                                        </form>



<?php
                        require_once('php/MysqlDB.php');
                        $id = $_SESSION['id'];

                        $Obj = new ScriptDB();
                        $Obj->Connect();
                        $Obj->ExecQuery("SELECT question,
DATE_FORMAT(registration_date,'%d/%m/%Y %H:%i'), attach FROM
saedd_questions WHERE user_id = '$id' ORDER BY registration_date DESC");


                        $count = $Obj->Get('RecordCount');

                        echo "<div
class='clear'></div><br/><br/><br/><h2>Você fez $count Pergunta(s)
</h2><br/><div id='Unique-ID' class='rt-toggle CSS-Class-Name'>
                                <ol>";


                        for($i=0; $i<$count; $i++)
                        {
                                $num = $i+1;
                                $title = "Pergunta realizada em ".$Obj-
>GetQueryValue($i, 1);
                                $content = utf8_decode($Obj-
>GetQueryValue($i, 0));
                                $attach = $Obj->GetQueryValue($i, 2);

                                if($attach != "")
                                   $attach =
"<br/><br/><strong>Anexo:</strong> <a href='files/questions/$attach'
target='_blank'>Anexo da pergunta</a>";

                                echo "<li>
                                        <div class='toggle-head'><div
class='toggle-number'>$num</div>
                                        <div class='toggle-
title'>$title</div></div>
                                        <div class='toggle-content'><div
class='wpb_text_column wpb_content_element '>
```

```
                                        <div class='wpb_wrapper'>

        <p>$content$attach</p></div></div>
                                        </div>
                                </li>";

                        }
                        echo "</ol>
                                </div>";

?>

                        </div>
                </div>

        </div>
</div>

<?php $myid = uniqid(""); echo "<input type='hidden' value='' id='attach'
/>" ?>

<script type="text/javascript"
src="js/plupload/plupload.full.min.js"></script>

<script type="text/javascript">



var uploader = new plupload.Uploader({
  browse_button: 'upbt',
  url : 'js/plupload/upload.php?path=',
filters: {
  max_file_size: "10mb"
}
});



uploader.init();

uploader.bind('FilesAdded', function(up, files) {

  var html = '';
  plupload.each(files, function(file) {

  var fileName = file.name;
  var ext = fileName.split('.').pop();
  uploader.settings.url =
"js/plupload/upload.php?path=files/questions/&myfile=<?php echo $myid;
?>."+ext;

   document.getElementById('msg').innerHTML += "Enviando <b>"
+file.name+"</b> (<b id='"+file.id+"'></b> de
"+plupload.formatSize(file.size)+")";

  });

  document.getElementById('msg').innerHTML += html;
  uploader.start();
});

uploader.bind('UploadProgress', function(up, file) {
        document.getElementById(file.id).innerHTML = file.percent + "%";
});
```

```
uploader.bind('Error', function(up, err) {
    if(err.code == '-601')
        document.getElementById('msg').innerHTML = "Arquivo enviado não é
um formato válido: <b>"+err.file.name+"</b>.<br/>Envie em '.JPG'";
    else if(err.code == '-600')
        document.getElementById('msg').innerHTML = "Arquivo enviado é
grande demais: <b>"+err.file.name+"</b> O limite é '2 MB'.";
    else
        document.getElementById('msg').innerHTML = err.code + ": " +
err.message;
});

uploader.bind('FileUploaded', function(up, file, info){
        var fileName = file.name;
        var ext = fileName.split('.').pop();
        jQuery('#msg').html(file.name+" enviado com sucesso!");
        jQuery("#attach").val("<?php echo $myid; ?>."+ext);
});

</script>



<div  class="content_row row vc_row wpb_row vc_row-fluid default-style
fullwidth" style="position:relative;overflow:hidden;">
        <div class="rt-parallax-background" data-rt-parallax-
direction="1" data-rt-parallax-effect="horizontal" style="background-
image: url(images/portfolio_header.jpg);background-repeat:
repeat;background-size: cover;background-position: right
top;width:100%;height:100%;top:0;"></div>
        <div class="content_row_wrapper  default" style="padding-
top:25px;padding-bottom:15px;">
                <div class="vc_col-sm-12 wpb_column vc_column_container"
>
                        <div class="wpb_wrapper">
                                <div class="rt_heading_wrapper style-4">
                                        <h1 class="rt_heading  style-4"
>Eventos</h1>
                                </div>
                        </div>
                </div>
        </div>
</div>


<div  class="content_row row vc_row wpb_row vc_row-fluid default-style
fullwidth border_grid fixed_heights" >
        <div class="content_row_wrapper  default" style="padding-
top:50px;padding-bottom:50px;">
                <div class="vc_col-sm-12 wpb_column vc_column_container"
>
                        <div class="wpb_wrapper">
                                <div class="rt_heading_wrapper style-6">
                                        <h4 class="rt_heading  style-6"
><span class="icon-glyph-2 heading_icon"></span>Próximos eventos</h4>
                                </div>
                                <section class="latest_news clearfix
style-1">
                                        <article>
                                                <div class="date">
                                                        <span
class="day">16<br/></span>
                                                        <span
class="year">Nov 2017</span>
                                                </div>
```

```
                                                <div class="text">
                                                        <h5
class="clean_heading"><a class="title" href="" title=""
rel="bookmark"><b>Congresso da Sociedade Brasileira de
Diabetes</b></a></h5>
                                                        <p><b>Data:</b>
16-18 de Novembro de 2017<br/><b>Local:</b> São Paulo -
SP<br/><b>Site:</b> <a href="http://www.diabetes2017.com.br/"
target="_blank">www.diabetes2017.com.br/</a></p>
                                                </div>
                                        </article>
                                        <article>
                                                <div class="date">
                                                        <span
class="day">13</span>
                                                        <span
class="year">Nov 2017</span>
                                                </div>
                                                <div class="text">
                                                        <h5
class="clean_heading"><a class="title" href="" title=""
rel="bookmark"><b>20ª Campanha Nacional Gratuita em Diabetes de Prevenção
das Complicações, Detecção, Orientação e Educação</b></a></h5>
                                                        <p><b>Data:</b>
13-18 de Novembro de 2017<br/><b>Local:</b> São Paulo -
SP<br/><b>Site:</b> <a href="http://www.anad.org.br/06-campanha/"
target="_blank">www.anad.org.br/06-campanha/</a></p>
                                                </div>
                                        </article>

                                </section>
                        </div>
                </div>
        </div>
</div>


<div  class="content_row row vc_row wpb_row vc_row-fluid alt-style-1
fullwidth border_grid fixed_heights" >
        <div class="content_row_wrapper  default" style="padding-
top:50px;padding-bottom:50px;">
                <div class="vc_col-sm-12 wpb_column vc_column_container"
>
                        <div class="wpb_wrapper">
                                <div class="rt_heading_wrapper style-6">
                                        <h4 class="rt_heading  style-6"
><span class="icon-glyph-2 heading_icon"></span>Outros eventos</h4>
                                </div>
                                <section class="latest_news clearfix
style-1">
                                        <article>
                                                <div class="date">
                                                        <span
class="day">10</span>
                                                        <span
class="year">Jun 2016</span>
                                                </div>
                                                <div class="text">
                                                        <h5
class="clean_heading"><a class="title" href="" title=""
rel="bookmark"><b>Palestra: Atividade Física, Diabetes e
Saúde</b></a></h5>
                                                        <p><b>Local:</b>
Auditório do LabiMPH / USP<br/><b>Horário:</b> 08:00 - 10:00</p>
                                                </div>
```

```
                                        </article>
                                        <article>
                                                <div class="date">
                                                        <span
class="day">17</span>
                                                        <span
class="year">Jul 2016</span>
                                                </div>
                                                <div class="text">
                                                        <h5
class="clean_heading"><a class="title" href="" title="Audio Post"
rel="bookmark"><b>Seleção de voluntários</b></a></h5>
                                                        <p><b>Local:</b>
Quadra poliesportiva da USP<br/><b>Horário:</b> 18:00 - 19:00</p>
                                                </div>
                                        </article>

                                </section>
                        </div>
                </div>
        </div>
</div>

<?php

        function GetRealIp()
        {
                $ip = "Not found";

                if (!empty($_SERVER['HTTP_CLIENT_IP']))
                    $ip = $_SERVER['HTTP_CLIENT_IP'];
                 elseif (!empty($_SERVER['HTTP_X_FORWARDED_FOR']))
                    $ip = $_SERVER['HTTP_X_FORWARDED_FOR'];
                 else
                    $ip = $_SERVER['REMOTE_ADDR'];

                return $ip;
        }

        if(isset($_POST['action']))
        {
                $ac = $_POST['action'];

                if($ac == "login")
                {
                        require_once('../MysqlDB.php');
                        $email =
utf8_decode(str_replace("'","''",$_POST['email']));
                        $pass =
utf8_decode(str_replace("'","''",$_POST['pass']));

                        $Obj = new ScriptDB();
                        $Obj->Connect();
                        $Obj->ExecQuery("SELECT name, id FROM saedd_users
WHERE email = '$email' AND pass = '$pass'");

                        $count = $Obj->Get('RecordCount');

                        /*
                        if($email != "alisson-amorim@hotmail.com")
                        {
                           echo utf8_encode("Sem conexão com o banco de
dados...");
                           return;
                        }
```

```
                        */

                        if($count == 1)
                        {
                           session_start();
                           $_SESSION['email'] = $email;
                           $_SESSION['pass'] = $pass;
                           $_SESSION['name'] = $Obj->GetQueryValue(0, 0);
                           $_SESSION['id'] = $Obj->GetQueryValue(0, 1);
                           $id = $Obj->GetQueryValue(0, 1);
                           $ip = GetRealIp();
                           $nome = $Obj->GetQueryValue(0,0);

                           echo utf8_encode("ok|Olá $nome,<br/>Seja bem
vindo! Aguarde...");

                           $Obj->ExecQuery("INSERT INTO
saedd_access(user_id, access_datetime, ip) VALUES('$id', NOW(), '$ip')");
                        }
                        else
                          echo utf8_encode("Usuário não enconcontrado");
                }
                else if($ac == "recovery")
                {
                        require_once('../MysqlDB.php');
                        require_once('../email.php');
                        $email =
utf8_decode(str_replace("'","''",$_POST['email']));

                        $Obj = new ScriptDB();
                        $Obj->Connect();
                        $Obj->ExecQuery("SELECT name, pass FROM
saedd_users WHERE email = '$email'");

                        $count = $Obj->Get('RecordCount');

                        if($count == 1)
                        {
                                $nome = $Obj->GetQueryValue(0,0);
                                $senha = $Obj->GetQueryValue(0,1);

                                SendPHPMail($email,"","", "", "SoPeD -
Recuperação de Senha", "Prezado(a) <b>$nome</b><br/>Foi solicitado uma
recuperação de senha para esse e-mail. Por favor ignore caso não tenha
pedido.<br/><br/>Os dados de acesso são:<br/><br/> <b>E-mail:</b>
".$email."<br/><b>Senha:</b> ".$senha."<br/><br/>Esperamos que o software
promova uma melhora significativa da saúde dos seus
pés!<br/><br/><b>Obrigado por experimentar o software!</b><br/>Equipe
SoPeD");
                                echo "$nome,<br/>enviamos a senha para o
seu e-mail.";
                        }
                        else
                          echo utf8_encode("Usuário não enconcontrado");
                }

                return;
        }
?>

<div  class="content_row row vc_row wpb_row vc_row-fluid default-style
fullwidth" style="position:relative;overflow:hidden;">
        <div class="rt-parallax-background" data-rt-parallax-
direction="1" data-rt-parallax-effect="horizontal" style="background-
image: url(images/portfolio_header.jpg);background-repeat:
```

```
repeat;background-size: cover;background-position: right
top;width:100%;height:100%;top:0;"></div>
        <div class="content_row_wrapper  default" style="padding-
top:25px;padding-bottom:15px;">
                <div class="vc_col-sm-12 wpb_column vc_column_container"
>
                        <div class="wpb_wrapper">
                                <div class="rt_heading_wrapper style-4">
                                        <h1 class="rt_heading  style-4"
>Autoavaliação SoPeD</h1>
                                </div>
                        </div>
                </div>
        </div>
</div>
<link rel='stylesheet' href='css/myform.css' type='text/css' media='all'
/>

<style type="text/css">
#myform input[type="text"],
#myform input[type="date"],
#myform input[type="datetime"],
#myform input[type="email"],
#myform input[type="number"],
#myform input[type="search"],
#myform input[type="time"],
#myform input[type="url"],
#myform input[type="password"],
#myform textarea,
#myform select {

    border-top: 1px solid #f0f0f0;
    border-left: 1px solid #f0f0f0;
    border-bottom: 1px solid #FAFAFA;
    border-right: 1px solid #FAFAFA;
    background: #e0e0e0;
}

.alt-style-1 input[type="button"]:hover {background-color: #FC9D0D;}
.alt-style-1 input[type="button"] {background-color: #428bca; font-
weight: bold;}

.alt-style-1 input[type="button"]:hover.extra  {background-color: #BBB;}
.alt-style-1 input[type="button"].extra  {background-color: #888; font-
weight: bold;}


</style>


<script type="text/javascript">

                function MakeLogin()
                {
                        jQuery('#msg').html("");
                        if(jQuery('#email').val() == "" ||
jQuery('#pass').val().length < 3)
                        {
                                jQuery('#msg').html("Informe o email e a
senha corretamente.");
                                return;
                        }
                        jQuery('#msg').html("Aguarde...");

                        jQuery.ajax({
```

```
                                        type: "POST",
                                        url: "php/pages/start.php",
                                        data: {email:
jQuery('#email').val(), pass :  jQuery('#pass').val(), action : 'login'}
                                })
                                .done(function(result)
                                {
                                        var str = result;
                                        str = str.split("|");
                                        if(str.length == 2 && str[0] ==
"ok")
                                        {
                                           jQuery('#msg').html(str[1]);
                                           document.myform.submit();
                                        }
                                        else

jQuery('#msg').html(result).show();
                                });
                }

                function RecoveryPassword()
                {
                        jQuery('#msg').html("");
                        if(jQuery('#email').val() == "")
                        {
                                jQuery('#msg').html("Informe o email no
qual você fez o cadastro.");
                                return;
                        }
                        jQuery('#msg').html("Aguarde...");

                        jQuery.ajax({
                                        type: "POST",
                                        url: "php/pages/start.php",
                                        data: {email:
jQuery('#email').val(), action : 'recovery'}
                                })
                                .done(function(result)
                                {
                                        jQuery('#msg').html(result);
                                });
                }

</script>

<div  class="content_row row vc_row wpb_row vc_row-fluid alt-style-1
fullwidth border_grid fixed_heights" >
        <div class="content_row_wrapper  default" >
                <div class="vc_col-sm-6 wpb_column vc_column_container" >
                        <div class="wpb_wrapper">
                                <div class="rt_heading_wrapper style-1">
                                        <h4 class="rt_heading  style-1"
>Já é cadastrado? Acesse!</h4>
                                </div>
                                <div class="wpb_text_column
wpb_content_element ">
                                        <div class="wpb_wrapper">
                                                <p>Informe o email criado
durante o seu cadastro.<br/><br/></p>
                                        </div>
```

```
                                        <form action="index.php"
name="myform" class="validate_form rt_form" method="post" id="myform"
style='width: 75%;'>


                                                        <label>E-
mail</label>
                                                        <input
type="text" name="email" id='email' style='margin-bottom: 20px;'/>


        <label>Senha</label>
                                                        <input
type="password" name="pass" id='pass' style='margin-bottom: 20px;' />
                                                        <input
type='hidden' name='page' value='start' />
                                                <div id="msg"
style='color: #FF2233;'></div><br/>

                                                <div class="submit-
button">
                                                        <input
class="button submit" onclick="javascript:MakeLogin();" value="Acessar"
type="button">
                                                        <input
class="button submit extra" onclick="javascript:RecoveryPassword();"
value="Esqueci" type="button">
                                                </div>

                                        </form>
                                </div>


                        </div>
                </div>
                <div class="vc_col-sm-6 wpb_column vc_column_container" >
                        <div class="wpb_wrapper">
                                <div class="rt_heading_wrapper style-1">
                                        <h4 class="rt_heading  style-1"
>Novo usuário? Cadastre-se agora!</h4>
                                </div>
                                <div class="wpb_text_column
wpb_content_element ">
                                        <div class="wpb_wrapper">
                                                <p style='text-align:
justify;'>O cadastro é rápido e fácil. Ao se cadastrar no SoPeD cada
usuário pode salvar e recuperar os avanços de uso na ferramenta em uma
área personalizada, que o ajudará a executar exercícios e emitir
lembretes.</p>


                                        <div class="submit-button">
                                                <input class="button
submit" value="Iniciar cadastro" onclick="javascript:location.href =
'index.php?page=register';" type="button">
                                        </div>


                                        </div>
                                </div>
                        </div>
                </div>
        </div>
</div>
```

```
<!--
<div  class="content_row row vc_row wpb_row vc_row-fluid alt-style-2
fullwidth border_grid fixed_heights" >
        <div class="content_row_wrapper  default" style="padding-
top:100px;padding-bottom:140px;">
                <div class="vc_col-sm-3 wpb_column vc_column_container" >
                        <div class="wpb_wrapper">
                                <div  class="rt_counter"><span
class="number">174</span>Web Design Projects</div>
                        </div>
                </div>
                <div class="vc_col-sm-3 wpb_column vc_column_container" >
                        <div class="wpb_wrapper">
                                <div  class="rt_counter"><span
class="number">299</span>Video Productions</div>
                        </div>
                </div>
                <div class="vc_col-sm-3 wpb_column vc_column_container" >
                        <div class="wpb_wrapper">
                                <div  class="rt_counter"><span
class="number">150</span>Animations</div>
                        </div>
                </div>
                <div class="vc_col-sm-3 wpb_column vc_column_container" >
                        <div class="wpb_wrapper">
                                <div  class="rt_counter"><span
class="number">1100</span>Graphic Designs</div>
                        </div>
                </div>
        </div>
</div>
-->


<div  class="content_row row vc_row wpb_row vc_row-fluid default-style
fullwidth" >
        <div class="content_row_wrapper  default" style="padding-
top:60px;padding-bottom:0px;">
                                <h2>Conheça algumas das vantagens de usar
o SoPeD</h2>
                <div class="vc_col-sm-4 wpb_column vc_column_container" >
                        <div class="wpb_wrapper">
                                <div class="vc_empty_space"
style="height: 60px" ><span class="vc_empty_space_inner"></span></div>
                                <div  class="chained_contents style-2
right" data-rt-animation-group="group">
                                        <div  class="" data-rt-
animate="animate" data-rt-animation-type="fadeInDown">
                                                <span
class="number">1</span>
                                                <div class="list-
content">
                                                        <p>Melhora da
saúde dos seus pés tornando-o mais saudável e funcional.</p>
                                                </div>
                                        </div>
                                        <div  class="" data-rt-
animate="animate" data-rt-animation-type="fadeInDown">
                                                <span
class="number">2</span>
                                                <div class="list-
content">
                                                        <p>Proporciona um
programa de exercícios personalizados e uma série de autoavaliações.</p>
```

```
                                                </div>
                                        </div>
                                        <div  class="" data-rt-
animate="animate" data-rt-animation-type="fadeInDown">
                                                <span
class="number">3</span>
                                                <div class="list-
content">
                                                        <p>Notificação
para a realização de exercícios via e-mail.</p>
                                                </div>
                                        </div>
                                </div>
                        </div>
                </div>
                <div class="vc_col-sm-4 wpb_column vc_column_container" >
                        <div class="wpb_wrapper">
                                <div class="wpb_single_image
wpb_content_element vc_align_center">
                                        <br/><br/><br/>
                                        <figure class="wpb_wrapper
vc_figure">
                                                <div
class="vc_single_image-wrapper   vc_box_border_grey"><img width="400"
height="658" src="images/avatar.png" class="vc_single_image-img
attachment-full" alt="wonen" /></div>
                                        </figure>
                                </div>
                        </div>
                </div>
                <div class="vc_col-sm-4 wpb_column vc_column_container" >
                        <div class="wpb_wrapper">
                                <div class="vc_empty_space"
style="height: 60px" ><span class="vc_empty_space_inner"></span></div>
                                <div  class="chained_contents style-2
left" data-rt-animation-group="group">
                                        <div  class="" data-rt-
animate="animate" data-rt-animation-type="fadeInDown">
                                                <span
class="number">4</span>
                                                <div class="list-
content">
                                                        <p>A ferramenta é
gratuita e online.</p>
                                                </div>
                                        </div>
                                        <div  class="" data-rt-
animate="animate" data-rt-animation-type="fadeInDown">
                                                <span
class="number">5</span>
                                                <div class="list-
content">
                                                        <p>Tire suas
dúvidas com os especialistas e fique por dentro das últimas notícias.</p>
                                                </div>
                                        </div>
                                        <div  class="" data-rt-
animate="animate" data-rt-animation-type="fadeInDown">
                                                <span
class="number">6</span>
                                                <div class="list-
content">
                                                        <p>Possibilidade
de acompanhar a sua evolução e trocar experiências com outros
usuários.</p>
```

```
                                                  </div>
                                          </div>
                                  </div>
                          </div>
                  </div>
          </div>
  </div>


          <script type="text/javascript"
  src="js/plupload/plupload.full.min.js"></script>



  <link rel='stylesheet' href='css/myform.css' type='text/css' media='all'
  />

  <style type="text/css">
  #myform input[type="text"],
  #myform input[type="date"],
  #myform input[type="datetime"],
  #myform input[type="email"],
  #myform input[type="number"],
  #myform input[type="search"],
  #myform input[type="time"],
  #myform input[type="url"],
  #myform input[type="password"],
  #myform textarea,
  #myform select {

      border-top: 1px solid #f0f0f0;
      border-left: 1px solid #f0f0f0;
      border-bottom: 1px solid #FAFAFA;
      border-right: 1px solid #FAFAFA;
      background: #e0e0e0;
  }

  .alt-style-1 input[type="button"]:hover {background-color: #FC9D0D;}
  .alt-style-1 input[type="button"] {background-color: #428bca; font-
  weight: bold;}

  .alt-style-1 input[type="button"]:hover.extra  {background-color: #BBB;}
  .alt-style-1 input[type="button"].extra  {background-color: #888; font-
  weight: bold;}
  b span{color: #428bca;}



  .default-style input[type="button"]:hover {background-color: #FC9D0D;}
  .default-style input[type="button"] {background-color: #428bca; font-
  weight: bold;}

  .default-style input[type="button"]:hover.extra  {background-color:
  #BBB;}
  .default-style input[type="button"].extra  {background-color: #888; font-
  weight: bold;}

  </style>


          <?php include("php/pages/internal.php"); ?>
```

```
<script type="text/javascript">


        function solicitarAmizade(id) {

                window.location = 'index.php?page=comunicacao&id='+id;

        }
</script>

        <style>
                .icircle img {-webkit-border-radius: 124px;
                        -moz-border-radius: 124px;
                        border-radius: 124px;
                        width: 120px;}
                .wpb_wrapper input[type="button"] {background: #428BCA;}
                .wpb_wrapper input[type="button"]:hover {background:
#317AB9;}
        </style>


<div  class="content_row default-style no-composer overlap fullwidth">
        <div class="content_row_wrapper clearfix ">
                <div class="vc_col-sm-12 wpb_column vc_column_container"
>
                        <div class="wpb_wrapper">




                                <table style="width: 100%;">
                                        <tr>


                                                <td>


                                        <?php

                                $myName = "";
                        require_once('php/MysqlDB.php');

                        $id = $_SESSION['id'];

                        $Obj = new ScriptDB();
                        $Obj->Connect();
                        $Obj->ExecQuery("SELECT name, coins, medals,
trophies, gender, saedd_users.id FROM saedd_users LEFT JOIN saedd_awards
ON saedd_users.id = user_id WHERE saedd_users.id = '$id' ORDER BY
4,3,2,1");

                        $count = $Obj->Get('RecordCount');

                        for($i=0; $i<$count; $i++) {
                           $nome = $Obj->GetQueryValue($i, 0);
                           $coins = $Obj->GetQueryValue($i, 1);
                           $medals = $Obj->GetQueryValue($i, 2);
                           $trophies = $Obj->GetQueryValue($i, 3);
                           $gender = $Obj->GetQueryValue($i, 4);
                           $user = $Obj->GetQueryValue($i, 5);
                           $myName = $nome;

                            if(file_exists("files/users/$user.jpg")) {
                                echo "
```

```
                                                <td class='icircle'
style='width: 140px;'>
                                                        <br/><img
src='files/users/$user.jpg' >
                                                </td>
                                ";
                            }
                            else if($gender == "M") {
                                echo "
                                                <td class='icircle'
style='width: 140px;'>
                                                        <br/><img
src='images/t2.png' >
                                                </td>
                                ";
                            }
                            else {
                                echo "
                                                <td class='icircle'
style='width: 140px;'>
                                                        <br/><img
src='images/t1.png' >
                                                </td>
                                ";
                            }

                           echo "               <td>

        <br/><h2>$nome</h2><br/>
                                                        <img
src='images/coin.png' style='height: 60px;'> x $coins
                                                        <img
src='images/medal.png' style='height: 60px;'> x $medals
                                                        <img
src='images/trophy.png' style='height: 60px;'> x $trophies<br/><br/>
                                                </td>
                                                <td  style='width:
120px;'>
                                                        <br/><br/><input
type='button' onclick=\"window.location = 'index.php?page=lojinha'; \"
value='Trocar pontos por brindes' />

                                                        <br/><br/><input
type='button' onclick=\" window.open('forum', '_blank',
'toolbar=yes,scrollbars=yes,resizable=yes');  \" value='Forum SoPeD' />
                                                </td>

                                ";

                        }


                        ?>



                                          </tr>

                                </table>
                                          <br/>
                                          <div
class="addthis_inline_share_toolbox" ></div>
                                          <script type="text/javascript"
src="//s7.addthis.com/js/300/addthis_widget.js#pubid=ra-
58fa5601eaa0c0e1"></script>
```

```
                        </div>
                </div>
        </div>
</div>




<div  class="content_row row vc_row wpb_row vc_row-fluid alt-style-1
fullwidth border-top border-bottom" >
        <div class="content_row_wrapper  default" style="padding-
top:60px;padding-bottom:60px;">
                <div class="vc_col-sm-12 wpb_column vc_column_container"
>
                        <div class="wpb_wrapper">
                                                        <input
type='button' onclick=" window.open('forum', '_blank',
'toolbar=yes,scrollbars=yes,resizable=yes');  " value='Forum SoPeD' />
        <br/><br/><br/><br/>
                                <input type="text" placeholder="Procurar
pessoa" style="width: 100%;  border-radius: 20px; background:
#FFF;"/><br/><br/><br/>
                                <table style="width: 100%;">



                                        <?php


                        $Obj->ExecQuery("SELECT name, coins, medals,
trophies, gender, saedd_users.id FROM saedd_users LEFT JOIN saedd_awards
ON saedd_users.id = user_id WHERE saedd_users.id != '$id' ORDER BY
4,3,2,1");

                        $count = $Obj->Get('RecordCount');

                        for($i=0; $i<$count; $i++) {
                           $nome = $Obj->GetQueryValue($i, 0);
                           $coins = $Obj->GetQueryValue($i, 1);
                           $medals = $Obj->GetQueryValue($i, 2);
                           $trophies = $Obj->GetQueryValue($i, 3);
                           $gender = $Obj->GetQueryValue($i, 4);
                           $user = $Obj->GetQueryValue($i, 5);


                                echo "<tr>";
                            if(file_exists("files/users/$user.jpg")) {
                                echo "
                                                <td class='icircle'
style='width: 140px;'>
                                                        <br/><img
src='files/users/$user.jpg' >
                                                </td>
                                ";
                            }
                            else if($gender == "M") {
                                echo "
                                                <td class='icircle'
style='width: 140px;'>
                                                        <br/><img
src='images/t2.png' >
                                                </td>
                                ";
                            }
                            else {
```

```
                                echo "
                                                <td class='icircle'
style='width: 140px;'>
                                                        <br/><img
src='images/t1.png' >
                                                </td>
                                ";
                            }

                           echo "               <td>

        <br/><h2>$nome</h2><br/>
                                                        <img
src='images/coin.png' style='height: 60px;'> x $coins
                                                        <img
src='images/medal.png' style='height: 60px;'> x $medals
                                                        <img
src='images/trophy.png' style='height: 60px;'> x $trophies<br/><br/>
                                                </td>


                                ";

                        }
                                echo "</tr>";

                        ?>






                                </table>
                        </div>
                </div>
        </div>
</div>
<div  class="content_row row vc_row wpb_row vc_row-fluid default-style
fullwidth" style="position:relative;overflow:hidden;">
        <div class="rt-parallax-background" data-rt-parallax-
direction="1" data-rt-parallax-effect="horizontal" style="background-
image: url(images/portfolio_header.jpg);background-repeat:
repeat;background-size: cover;background-position: right
top;width:100%;height:100%;top:0;"></div>
        <div class="content_row_wrapper  default" style="padding-
top:25px;padding-bottom:15px;">
                <div class="vc_col-sm-12 wpb_column vc_column_container"
>
                        <div class="wpb_wrapper">
                                <div class="rt_heading_wrapper style-4">
                                        <h1 class="rt_heading  style-4"
>Conheça o Projeto</h1>
                                </div>
                        </div>
                </div>
        </div>
</div>


<div  class="content_row row vc_row wpb_row vc_row-fluid default-style
fullwidth" >
        <div class="content_row_wrapper  default" style="padding-
top:40px;padding-bottom:0px; min-height: 800px;">
                <div class="vc_col-sm-8 wpb_column vc_column_container" >
```

```
                        <div class="wpb_wrapper">
                                <div class="rt_quote" >
                                        <p style="text-align: justify;
font-size: 15px;">
                                                <span class="icon-quote-
left"></span>
                                                        O sistema é fruto
de uma linha de pesquisa do <a href="http://www.usp.br/labimph/"
target="_blank">Laboratório de Biomecânica do Movimento e Postura Humana
- (LABIMPH)</a>, desenvolvido com a intenção de disponibilizar
gratuitamente diversas informações sobre o Diabetes Mellitus e a maneira
como a doença afeta os pés dos indivíduos com essa doença. Através de
recursos interativos é possível registrar sua evolução de autoavaliação e
de exercícios específicos para manter a saúde dos pés.
                                                <span class="icon-quote-
right"></span>
                                        </p>
                                </div>

                                <div class="vc_empty_space"
style="height: 32px" ><span class="vc_empty_space_inner"></span></div>
                        </div>
                </div>
                <div class="vc_col-sm-4 wpb_column vc_column_container" >
                        <div class="wpb_wrapper">
                                <div class="wpb_single_image
wpb_content_element vc_align_center">
                                        <figure class="wpb_wrapper
vc_figure">
                                                <div
class="vc_single_image-wrapper vc_box_border_grey"><img
src="http://biton.uspnet.usp.br/labimph/wp-
content/themes/tema_Labimph/imagens/Logo.png" class="vc_single_image-img
attachment-full"  /></div>
                                        </figure>
                                </div>
                        </div>
                </div>
        </div>
</div>


<div  class="content_row row vc_row wpb_row vc_row-fluid default-style
fullwidth" style="position:relative;overflow:hidden;">
        <div class="rt-parallax-background" data-rt-parallax-
direction="1" data-rt-parallax-effect="horizontal" style="background-
image: url(images/portfolio_header.jpg);background-repeat:
repeat;background-size: cover;background-position: right
top;width:100%;height:100%;top:0;"></div>
        <div class="content_row_wrapper  default" style="padding-
top:25px;padding-bottom:15px;">
                <div class="vc_col-sm-12 wpb_column vc_column_container"
>
                        <div class="wpb_wrapper">
                                <div class="rt_heading_wrapper style-4">
                                        <h1 class="rt_heading  style-4"
>Regras do Jogo</h1>
                                </div>
                        </div>
                </div>
        </div>
</div>
```

```
<script type="text/javascript">

        var pos = 3;

        function showArea() {

                for(var i=1; i<=6; i++)
                  jQuery("#area"+i).css("display","none");

                jQuery("#area"+pos ).css("display","inline");
                if(pos <= 6) {
                  pos++;
                return;
                }

                alert("Essas são todas as regras.");
        }
</script>


<div  class="content_row row vc_row wpb_row vc_row-fluid alt-style-1
fullwidth border_grid fixed_heights" >
        <div class="content_row_wrapper  default" style="padding-
top:50px;padding-bottom:50px; min-height: 500px;">
                <div class="vc_col-sm-12 wpb_column vc_column_container"
>
                        <div class="wpb_wrapper">




                                        <div id='area2'>
                                           <h2>RESUMO DE UMA PARTIDA -OU
SESSÃO</h2>
                                           Cada partida consiste na
realização de <b>8 exercícios para pés e tornozelos</b>.
                                           <br/>As partidas não
ultrapassam 20 minutos e podem ser executadas em qualquer ambiente de sua
escolha. <b>Recomendamos de 2 à 3 vezes por semana, sendo que não é
possível realizar 2 sessões no mesmo dia!</b><br/><br/>


                                           <h4>1 partida = 8 exercícios
(No máximo 20 minutos)</h4>

                                               <img
src="images/exercicio/man.png" style="width: 70px;">
                                               <img
src="images/exercicio/man.png" style="width: 70px;">
                                               <img
src="images/exercicio/man.png" style="width: 70px;">
                                               <img
src="images/exercicio/man.png" style="width: 70px;">
                                               <img
src="images/exercicio/man.png" style="width: 70px;">
                                               <img
src="images/exercicio/man.png" style="width: 70px;">
                                               <img
src="images/exercicio/man.png" style="width: 70px;">
                                               <img
src="images/exercicio/man.png" style="width: 70px;">
                                               <img
src="images/exercicio/right.png" >
```

```
                                                <img
src="images/date.png" >
                                                <br/><br/><br/>
                                        </div>



                                <div id='area3' style="display: none;">
                                        <h2>PREPARAÇÃO</h2>
                                        Para a realização dos exercícios
propostos tenha sempre por perto uma <b>toalha de mão, uma bolinha
pequena (que poderá ser de tênis, lisa ou cravo), uma bexiga (que deverá
previamente ser preenchida com maisena ou farinha de trigo), lápis,
algodão, uma faixa elástica (comumente utilizadas na fisioterapia ou um
elástico grosso)</b> e <span style='#color: #428BCA;'>muita
disposição</span>!<br/><br/>
                                                <h4>Reserve antes de uma
partida</h4>
                                                <img
src="images/exercicio/toalha.png" style="height: 80px;">
                                                <img
src="images/exercicio/tenis.png" style="height: 80px;">
                                                <img
src="images/exercicio/bexiga.png" style="height: 80px;">
                                                <img
src="images/exercicio/lapis.png" style="height: 80px;">
                                                <img
src="images/exercicio/thera.png" style="height: 80px;">
                                                <img
src="images/exercicio/algodao.png" style="height: 80px;">
                                                <img
src="images/exercicio/right.png" >
                                                <img
src="images/exercicio/sorriso.png" style="height: 80px;">
                                        <br/><br/><br/>
                                </div>

                                <div id='area4' style="display: none;">
                                        <h2>OBJETIVO</h2>
                                        Este programa de exercícios tem
como objetivo promover a realização de exercícios com foco nos
<strong>pés e tornozelos</strong>, com intuito de garantir uma boa
funcionalidade dos músculos e articulações que são comprometidos nas
pessoas com diabetes, especialmente as pessoas com polineuropatia.
                                        <br/><br/><strong>Funciona como
um jogo</strong> onde a chave para dominar o sistema depende de você!
Vamos entender como este jogo funciona?<br/><br/><br/>
                                </div>

                                <div id='area5' style="display: none;">
                                        <h2>Como funciona?</h2>
                                        Ao final de cada exercício você
mesmo avaliará a dificuldade de realizar o exercício, através de uma
escala de 0 à 10, sendo que 0 siginifia <b>NADA CANSATIVO</b> e 10
<b>MUITO CANSATIVO</b>.
                                        <br/><br/>
                                        <img src="images/ruler.png"
/><br/><br/><br/>
                                </div>

                                <div id='area6' style="display: none;">
                                        <h2>Recompensas</h2>
                                        A <strong>cada exercício</strong>
realização de um você será <strong>premiado com uma ficha</strong>,
independente se você achou fácil ou difícil.<br/>
```

```
                                        A cada exercício executado
<strong>sem dificuldade</strong> (Valores de 0 a 2 na régua), você
ganhará <strong>1 moeda</strong>.<br/>
                                        Com o acúmulo de <strong>8
moedas</strong>, você ganhará <strong>1 medalha</strong> e quando estiver
com <strong>2 medalhas</strong>, você ganhará <strong>um troféu</strong>
que lhe mudará de aumentará seu nível de experiência.<br/><br/>
                                        <strong>É de extrema importância
ser honesto consigo mesmo nas avaliações.</strong>
                                        <br/><br/>
                                        Dê o seu melhor e seja um expert
e compartilhe o seu desempenho com os seus amigos. Pronto para
começar?<br/><br/><br/>
                                        <br/><br/>
                                        <h4>Cada exercício = 1 ficha</h4>
                                        <img
src="images/exercicio/man.png" style="height: 80px;">
                                        <img
src="images/exercicio/right.png" >
                                        <img src="images/try.png"
style="height: 80px;"><br/><br/>

                                        <h4>Cada exercício sem
dificuldade = 1 moeda</h4>
                                        <img
src="images/exercicio/sorriso.png" style="height: 80px;">
                                        <img
src="images/exercicio/right.png" >
                                        <img src="images/coin.png"
style="height: 60px;"><br/><br/>

                                        <h4>Cada 8 moedas = 1
medalha</h4>
                                        <img src="images/coin.png"
style="height: 60px;">
                                        <img src="images/coin.png"
style="height: 60px;">
                                        <img src="images/coin.png"
style="height: 60px;">
                                        <img src="images/coin.png"
style="height: 60px;">
                                        <img src="images/coin.png"
style="height: 60px;">
                                        <img src="images/coin.png"
style="height: 60px;">
                                        <img src="images/coin.png"
style="height: 60px;">
                                        <img src="images/coin.png"
style="height: 60px;">
                                        <img
src="images/exercicio/right.png" >
                                        <img src="images/medal.png"
style="height: 60px;">
                                        <br/><br/>

                                        <h4>Cada 2 medalhas = 1 trofeu
(sobe de nível)</h4>
                                        <img src="images/medal.png"
style="height: 60px;">
                                        <img src="images/medal.png"
style="height: 60px;">
                                        <img
src="images/exercicio/right.png" >
                                        <img src="images/trophy.png"
style="height: 60px;">
```

```
                                        <img
src="images/exercicio/right.png" >
                                        <img
src="images/exercicio/levelup.png" style="height: 80px;">

                                        <br/><br/><br/>
                                </div>




                                <input type="button"
onclick="javascript:showArea();" value="Ok! Entendi e quero continuar..."
/>
                        </div>
                </div>
        </div>
</div>
<?php
                        function base64_to_file($base64, $id)
                        {
                            $ifp =
fopen("../../files/3dfoot/".$id.".png", "wb");
                            $data = explode(',', $base64);
                            fwrite($ifp, base64_decode($data[1]));
                            fclose($ifp);
                        }

        if(isset($_POST['action']))
        {
                $action = $_POST['action'];

                if($action == "step1")
                {

        require_once('../MysqlDB.php');
                                                $Obj = new ScriptDB();

                                                $Obj->Set("TableName",
"saedd_occur_01");
                                                $Obj->Set("FieldsName",
"fr_q1|fr_q2|fr_q3|fr_q4|fl_q1|fl_q2|fl_q3|fl_q4|user_id|registration_dat
e");
                                                $Obj-
>BeforeInsert("registration_date","NOW()");

                                                $sql = $Obj-
>CreateInsert();

                                                $Obj->Connect();

                                                $Obj->ExecQuery("DELETE
FROM saedd_occur_01 WHERE user_id = '".intval($_POST['user_id'])."'");
                                                $Obj->ExecQuery("DELETE
FROM saedd_occur_02 WHERE user_id = '".intval($_POST['user_id'])."'");
                                                $Obj->ExecQuery("DELETE
FROM saedd_occur_03 WHERE user_id = '".intval($_POST['user_id'])."'");


                                                if($Obj->ExecQuery($sql))
                                                {
                                                        $sql = "INSERT
INTO saedd_quests(user_id,quest_datetime,quest)
VALUES('".$_POST['user_id']."',NOW(),'occurrence_1');";
```

```
                                                            $Obj-
>ExecQuery($sql);

                                                            echo "1";

                                                    }
                                                    else
                                                    {
                                                              echo $Obj-
>GetLastError();
                                                    }

                                                    $Obj->CloseDB();


                }
                else if($action == "step2")
                {

        require_once('../MysqlDB.php');
                                                    $Obj = new ScriptDB();

                                                    $Obj->Set("TableName",
"saedd_occur_02");
                                                    $Obj->Set("FieldsName",
"fr_q1|fr_q2|fr_q3|fr_q4|fr_q5|fr_q6|fl_q1|fl_q2|fl_q3|fl_q4|fl_q5|fl_q6|
user_id|registration_date");
                                                    $Obj-
>BeforeInsert("registration_date","NOW()");

                                                    $sql = $Obj-
>CreateInsert();

                                                    $Obj->Connect();
                                                    if($Obj->ExecQuery($sql))
                                                    {
                                                            $sql = "INSERT
INTO saedd_quests(user_id,quest_datetime,quest)
VALUES('".$_POST['user_id']."',NOW(),'occurrence_2');";
                                                            $Obj-
>ExecQuery($sql);

                                                            echo "1";

                                                    }
                                                    else
                                                    {
                                                              echo $Obj-
>GetLastError();
                                                    }

                                                    $Obj->CloseDB();


                }
                else if($action == "step3")
                {

        require_once('../MysqlDB.php');
                                                    $Obj = new ScriptDB();

                                                    $Obj->Set("TableName",
"saedd_occur_03");
```

```
                                                            $Obj->Set("FieldsName",
"fr_q1|fr_q2|fr_q3|fr_q4|fr_q5|fr_q6|fr_q7|fl_q1|fl_q2|fl_q3|fl_q4|fl_q5|
fl_q6|fl_q7|user_id|registration_date");
                                                            $Obj-
>BeforeInsert("registration_date","NOW()");

                                                            $sql = $Obj-
>CreateInsert();

                                                            $Obj->Connect();
                                                            if($Obj->ExecQuery($sql))
                                                            {
                                                                    $sql = "INSERT
INTO saedd_quests(user_id,quest_datetime,quest)
VALUES('".$_POST['user_id']."',NOW(),'occurrence_3');";
                                                                    $Obj-
>ExecQuery($sql);
                                                                    $sql = "UPDATE
saedd_users SET stars = (stars+1) WHERE id =
'".trim($_POST['user_id'])."'";
                                                                    $Obj-
>ExecQuery($sql);
                                                                    echo "1";

                                                            }
                                                            else
                                                            {
                                                                      echo $Obj-
>GetLastError();
                                                            }

                                                            $Obj->CloseDB();


                }
                else if($action == "step4")
                {
                        base64_to_file($_POST['data'],
$_POST['user_id']."_scene_".$_POST['pos']);
                        echo "1";
                }
                else if($action == "conclude")
                {

        require_once('../MysqlDB.php');
                                                            $Obj = new ScriptDB();
                                                            $Obj->Connect();

                                                            $sql = "INSERT INTO
saedd_quests(user_id,quest_datetime,quest)
VALUES('".$_POST['user_id']."',NOW(),'occurrence_4');";
                                                            $Obj->ExecQuery($sql);
                                                            echo "1";

                                                            $Obj->CloseDB();
                }
                return;
        }
?>

<?php include("php/pages/back.php"); ?>

<link rel='stylesheet' href='css/myform.css' type='text/css' media='all'
/>
```

```
<style type="text/css">
#myform input[type="text"],
#myform input[type="date"],
#myform input[type="datetime"],
#myform input[type="email"],
#myform input[type="number"],
#myform input[type="search"],
#myform input[type="time"],
#myform input[type="url"],
#myform input[type="password"],
#myform textarea,
#myform select {

    border-top: 1px solid #f0f0f0;
    border-left: 1px solid #f0f0f0;
    border-bottom: 1px solid #FAFAFA;
    border-right: 1px solid #FAFAFA;
    background: #e0e0e0;
}

.default-style input[type="button"]:hover {background-color: #FC9D0D;}
.default-style input[type="button"] {background-color: #428bca; font-
weight: bold;}

.default-style input[type="button"]:hover.extra  {background-color:
#BBB;}
.default-style input[type="button"].extra  {background-color: #888; font-
weight: bold;}

h4 span {color: #428BCA;}

</style>


<script type="text/javascript">


        function ValidateStep1()
        {
           var leftFoot = ["0","0","0","0"];
           var rightFoot = ["0","0","0","0"];

           jQuery("#msg").html("");

           for(i=1; i<=3; i++)
           {
              pos = i-1;

              if(jQuery("#oc_"+i+"_fl").is(':checked') == true)
              {
                 leftFoot[pos] = "1";

                 if(jQuery("#fl_"+i+"_d1").is(':checked') == true)
                   leftFoot[pos] += "1";
                 else
                   leftFoot[pos] += "0";

                 if(jQuery("#fl_"+i+"_d2").is(':checked') == true)
                   leftFoot[pos]+= "1";
                 else
                   leftFoot[pos] += "0";

                 if(jQuery("#fl_"+i+"_d3").is(':checked') == true)
                   leftFoot[pos]+= "1";
                 else
```

```
                      leftFoot[pos] += "0";

                    if(jQuery("#fl_"+i+"_d4").is(':checked') == true)
                      leftFoot[pos] += "1";
                    else
                      leftFoot[pos] += "0";

                    if(jQuery("#fl_"+i+"_d5").is(':checked') == true)
                      leftFoot[pos] += "1";
                    else
                      leftFoot[pos] += "0";

                    if(leftFoot[pos] == "100000")
                    {
                       jQuery("#msg").html("Informe os dedos do pé esquerdo
referente a pergunta "+i);
                       return;
                    }

                 }

                 if(jQuery("#oc_"+i+"_fr").is(':checked') == true)
                 {
                    rightFoot[pos] = "1";

                    if(jQuery("#fr_"+i+"_d1").is(':checked') == true)
                      rightFoot[pos] += "1";
                    else
                      rightFoot[pos] += "0";

                    if(jQuery("#fr_"+i+"_d2").is(':checked') == true)
                      rightFoot[pos]+= "1";
                    else
                      rightFoot[pos] += "0";

                    if(jQuery("#fr_"+i+"_d3").is(':checked') == true)
                      rightFoot[pos]+= "1";
                    else
                      rightFoot[pos] += "0";

                    if(jQuery("#fr_"+i+"_d4").is(':checked') == true)
                      rightFoot[pos] += "1";
                    else
                      rightFoot[pos] += "0";

                    if(jQuery("#fr_"+i+"_d5").is(':checked') == true)
                      rightFoot[pos] += "1";
                    else
                      rightFoot[pos] += "0";

                    if(rightFoot[pos] == "100000")
                    {
                       jQuery("#msg").html("Informe os dedos do pé direto
referente a pergunta "+i);
                       return;
                    }

                 }

              }

              if(jQuery("#oc_4_fl").is(':checked') == true)
                 leftFoot[3] = "1";

              if(jQuery("#oc_4_fr").is(':checked') == true)
```

```
                rightFoot[3] = "1";



            jQuery.ajax({
                 type: "POST",
                 url: "php/pages/checklist.php",
                 data: {fr_q1 : rightFoot[0], fr_q2 : rightFoot[1], fr_q3
: rightFoot[2], fr_q4 : rightFoot[3], fl_q1 : leftFoot[0], fl_q2 :
leftFoot[1], fl_q3 : leftFoot[2], fl_q4 : leftFoot[3], user_id : <?php
echo $_SESSION['id']; ?>,  action : "step1"}
           })
          .done(function(html) {
                 if(html == "1")
                 {
                         var result = "<h2>Questionário de Ocorrências 1/3
respondido com sucesso!</h2><br/>";
                         result += "<p>Agradecemos a paciência! Continue o
cadastro para que possamos conhecer mais do seu pé.</p>";
                         result += "<br/><br/><div class='submit-
button'><input class='button submit' onclick=\"javascript:window.location
= 'index.php?page=checklist';\" value='Clique aqui para continuar...'
type='button'><span class='loading'></span></div>";


                         jQuery("#etapa1").html(result);
                         jQuery("html, body").animate({ scrollTop: 0 },
"slow");
                 }
                 else
                   jQuery("#msg").html("Erro: "+html);
           });
      }



        function ValidateStep2()
        {
           var leftFoot = ["0","0","0","0","0","0"];
           var rightFoot = ["0","0","0","0","0","0"];

           jQuery("#msg2").html("");

           for(i=5; i<=10; i++)
           {
              pos = i-1;

              if(jQuery("#oc_"+i+"_fl").is(':checked') == true)
              {
                 leftFoot[pos] = "1";

                 if(jQuery("#fl_"+i+"_d1").is(':checked') == true)
                   leftFoot[pos] += "1";
                 else
                   leftFoot[pos] += "0";

                 if(jQuery("#fl_"+i+"_d2").is(':checked') == true)
                   leftFoot[pos]+= "1";
                 else
                   leftFoot[pos] += "0";

                 if(jQuery("#fl_"+i+"_d3").is(':checked') == true)
                   leftFoot[pos]+= "1";
                 else
                   leftFoot[pos] += "0";
```

```
                    if(jQuery("#fl_"+i+"_d4").is(':checked') == true)
                      leftFoot[pos] += "1";
                    else
                      leftFoot[pos] += "0";

                    if(jQuery("#fl_"+i+"_d5").is(':checked') == true)
                      leftFoot[pos] += "1";
                    else
                      leftFoot[pos] += "0";

                    if(jQuery("#fl_"+i+"_d6").is(':checked') == true)
                      leftFoot[pos] += "1";
                    else
                      leftFoot[pos] += "0";

                    if(jQuery("#fl_"+i+"_d7").is(':checked') == true)
                      leftFoot[pos] += "1";
                    else
                      leftFoot[pos] += "0";

                    if(jQuery("#fl_"+i+"_d8").is(':checked') == true)
                      leftFoot[pos] += "1";
                    else
                      leftFoot[pos] += "0";

                    if(leftFoot[pos] == "100000000")
                    {
                       jQuery("#msg2").html("Informe os dedos ou região do
pé esquerdo referente a pergunta "+i);
                       return;
                    }

                 }

                 if(jQuery("#oc_"+i+"_fr").is(':checked') == true)
                 {
                    rightFoot[pos] = "1";

                    if(jQuery("#fr_"+i+"_d1").is(':checked') == true)
                      rightFoot[pos] += "1";
                    else
                      rightFoot[pos] += "0";

                    if(jQuery("#fr_"+i+"_d2").is(':checked') == true)
                      rightFoot[pos]+= "1";
                    else
                      rightFoot[pos] += "0";

                    if(jQuery("#fr_"+i+"_d3").is(':checked') == true)
                      rightFoot[pos]+= "1";
                    else
                      rightFoot[pos] += "0";

                    if(jQuery("#fr_"+i+"_d4").is(':checked') == true)
                      rightFoot[pos] += "1";
                    else
                      rightFoot[pos] += "0";

                    if(jQuery("#fr_"+i+"_d5").is(':checked') == true)
                      rightFoot[pos] += "1";
                    else
                      rightFoot[pos] += "0";

                    if(jQuery("#fr_"+i+"_d6").is(':checked') == true)
```

```
                    rightFoot[pos] += "1";
                  else
                    rightFoot[pos] += "0";

                  if(jQuery("#fr_"+i+"_d7").is(':checked') == true)
                    rightFoot[pos] += "1";
                  else
                    rightFoot[pos] += "0";

                  if(jQuery("#fr_"+i+"_d8").is(':checked') == true)
                    rightFoot[pos] += "1";
                  else
                    rightFoot[pos] += "0";

                  if(rightFoot[pos] == "100000000")
                  {
                     jQuery("#msg2").html("Informe os dedos ou região do
pé direto referente a pergunta "+i);
                     return;
                  }

               }

            }

            jQuery.ajax({
                 type: "POST",
                 url: "php/pages/checklist.php",
                 data: {fr_q1 : rightFoot[0], fr_q2 : rightFoot[1], fr_q3
: rightFoot[2], fr_q4 : rightFoot[3], fr_q5 : rightFoot[4], fr_q6 :
rightFoot[5], fl_q1 : leftFoot[0], fl_q2 : leftFoot[1], fl_q3 :
leftFoot[2], fl_q4 : leftFoot[3], fl_q5 : leftFoot[4], fl_q6 :
leftFoot[5], user_id : <?php echo $_SESSION['id']; ?>,  action : "step2"}
            })
          .done(function(html) {
                 if(html == "1")
                 {
                         var result = "<h2>Questionário de Ocorrências 2/3
respondido com sucesso!</h2><br/>";
                         result += "<p>Agradecemos a paciência! Continue o
cadastro para que possamos conhecer mais do seu pé.</p>";
                         result += "<br/><br/><div class='submit-
button'><input class='button submit' onclick=\"javascript:window.location
= 'index.php?page=checklist';\" value='Clique aqui para continuar...'
type='button'><span class='loading'></span></div>";


                         jQuery("#etapa2").html(result);
                         jQuery("html, body").animate({ scrollTop: 0 },
"slow");
                 }
                 else
                   jQuery("#msg2").html("Erro: "+html);
            });
      }



        function ValidateStep3()
        {
           var leftFoot = ["0","0","0","0","0","0","0"];
           var rightFoot = ["0","0","0","0","0","0","0"];

           var occNum = 0;
```

```
            jQuery("#msg2").html("");

            for(i=11; i<=16; i++)
            {
               pos = i-1;

               if(jQuery("#oc_"+i+"_fl").is(':checked') == true)
               {
                  occNum++;
                  leftFoot[pos] = "1";

                  if(jQuery("#fl_"+i+"_d1").is(':checked') == true)
                    leftFoot[pos] += "1";
                  else
                    leftFoot[pos] += "0";

                  if(jQuery("#fl_"+i+"_d2").is(':checked') == true)
                    leftFoot[pos]+= "1";
                  else
                    leftFoot[pos] += "0";

                  if(jQuery("#fl_"+i+"_d3").is(':checked') == true)
                    leftFoot[pos]+= "1";
                  else
                    leftFoot[pos] += "0";

                  if(jQuery("#fl_"+i+"_d4").is(':checked') == true)
                    leftFoot[pos] += "1";
                  else
                    leftFoot[pos] += "0";

                  if(jQuery("#fl_"+i+"_d5").is(':checked') == true)
                    leftFoot[pos] += "1";
                  else
                    leftFoot[pos] += "0";

                  if(jQuery("#fl_"+i+"_d6").is(':checked') == true)
                    leftFoot[pos] += "1";
                  else
                    leftFoot[pos] += "0";

                  if(jQuery("#fl_"+i+"_d7").is(':checked') == true)
                    leftFoot[pos] += "1";
                  else
                    leftFoot[pos] += "0";

                  if(jQuery("#fl_"+i+"_d8").is(':checked') == true)
                    leftFoot[pos] += "1";
                  else
                    leftFoot[pos] += "0";

                  if(leftFoot[pos] == "100000000")
                  {
                     jQuery("#msg3").html("Informe os dedos ou região do
pé esquerdo referente a pergunta "+i);
                     return;
                  }

               }

               if(jQuery("#oc_"+i+"_fr").is(':checked') == true)
               {
                  occNum++;
                  rightFoot[pos] = "1";
```

```
                    if(jQuery("#fr_"+i+"_d1").is(':checked') == true)
                      rightFoot[pos] += "1";
                    else
                      rightFoot[pos] += "0";

                    if(jQuery("#fr_"+i+"_d2").is(':checked') == true)
                      rightFoot[pos]+= "1";
                    else
                      rightFoot[pos] += "0";

                    if(jQuery("#fr_"+i+"_d3").is(':checked') == true)
                      rightFoot[pos]+= "1";
                    else
                      rightFoot[pos] += "0";

                    if(jQuery("#fr_"+i+"_d4").is(':checked') == true)
                      rightFoot[pos] += "1";
                    else
                      rightFoot[pos] += "0";

                    if(jQuery("#fr_"+i+"_d5").is(':checked') == true)
                      rightFoot[pos] += "1";
                    else
                      rightFoot[pos] += "0";

                    if(jQuery("#fr_"+i+"_d6").is(':checked') == true)
                      rightFoot[pos] += "1";
                    else
                      rightFoot[pos] += "0";

                    if(jQuery("#fr_"+i+"_d7").is(':checked') == true)
                      rightFoot[pos] += "1";
                    else
                      rightFoot[pos] += "0";

                    if(jQuery("#fr_"+i+"_d8").is(':checked') == true)
                      rightFoot[pos] += "1";
                    else
                      rightFoot[pos] += "0";

                    if(rightFoot[pos] == "100000000")
                    {
                       jQuery("#msg3").html("Informe os dedos ou região do
pé direto referente a pergunta "+i);
                       return;
                    }

                 }

              }

              jQuery.ajax({
                   type: "POST",
                   url: "php/pages/checklist.php",
                   data: {fr_q1 : rightFoot[0], fr_q2 : rightFoot[1], fr_q3
: rightFoot[2], fr_q4 : rightFoot[3], fr_q5 : rightFoot[4], fr_q6 :
rightFoot[5], fr_q7 : rightFoot[6], fl_q1 : leftFoot[0], fl_q2 :
leftFoot[1], fl_q3 : leftFoot[2], fl_q4 : leftFoot[3], fl_q5 :
leftFoot[4], fl_q6 : leftFoot[5], fl_q7 : leftFoot[6], user_id : <?php
echo $_SESSION['id']; ?>,  action : "step3"}
             })
           .done(function(html) {
                   if(html == "1")
                   {
```

```
                        var result = "<h2>Questionário de Ocorrências 3/3
respondido com sucesso!</h2><br/>";

                        if(occNum > 0)
                          result += "<p>Agradecemos a paciência! <b
style='#color: #cc2233;'>Infelizmente você não está apto a realizar os
exercícios de forma autônoma, pois as ocorrências relatadas requerem
cuidados profissionais.</b> Mostre essa ferramenta ao seu médido e
questione-o sobre como deverá proceder o uso da mesma. Agradecemos a sua
parcipação e esperamos em breve tê-lo de volta ao SAEDD.<br/><br/>Para
finalizar, Gostaríamos de pedir que fizesse um modelo 3D das ocorrências
em seu pé na próxima etapa. É fácil e divertido!</p>";
                        else
                          result += "<p>Agradecemos a paciência!
Aparentemente, você está apto a utilizar a ferramenta, entretanto, a
opnião profissional é muito importante. Consulte seu médico para mais
detalhes.<br/><br/>Para finalizar, Gostaríamos de pedir que fizesse um
modelo 3D das ocorrências em seu pé na próxima etapa. É fácil e
divertido!</p>";

                          var result = "<h2>Questionário de Ocorrências
respondido com sucesso!</h2><br/>";
                          result += "<p>Parabéns! Seu questionário de
exercícios está completo! Agora você poderá partir para os exercícios,
caso não tenhado obtido indicação contrária na ferramenta.</p>";
                          result += "<br/><br/><div class='submit-
button'><input class='button submit' onclick=\"javascript:window.location
= 'index.php?page=user_start';\" value='Concluir questionário'
type='button'><span class='loading'></span></div>";
                          result += "<br/><br/><h2>Obrigado por responder
todas as etapas!</h2><p>Você está pronto para avançar no SAEDD. Por
responder os questionários você ganhou uma estrela! <h2><img
src='images/star.png' /> x 1</h2><br/><b style='color: #428BCA;'>As
estrelas liberam ferramentas no software que auxiliam no cuidado dos seus
pés. Quanto mais estrelas, melhor a saúde do seu pé!</b></p>";



                        jQuery("#etapa3").html(result);
                        jQuery("html, body").animate({ scrollTop: 0 },
"slow");
                }
                else
                  jQuery("#msg3").html("Erro: "+html);
          });
     }



</script>

<?php
                        require_once('php/MysqlDB.php');
                        $id = $_SESSION['id'];

                        $Obj = new ScriptDB();
                        $Obj->Connect();

                        $Obj->ExecQuery("SELECT (SELECT COUNT(*) FROM
saedd_occur_01 WHERE user_id = '$id'), (SELECT COUNT(*) FROM
saedd_occur_02 WHERE user_id = '$id'), (SELECT COUNT(*) FROM
saedd_occur_03 WHERE user_id = '$id'), (SELECT
DATE_FORMAT(registration_date, '%d/%m/%Y') FROM saedd_occur_03 WHERE
user_id = '$id'), COALESCE((SELECT DATEDIFF(NOW(), registration_date)
```

```
FROM saedd_occur_03 WHERE user_id = '$id'),0), (SELECT
DATE_FORMAT(DATE_ADD(DATE(registration_date), interval 30 day),
'%d/%m/%Y') FROM saedd_occur_03 WHERE user_id = '$id')");

                        $oc1 = $Obj->GetQueryValue(0, 0);
                        $oc2 = $Obj->GetQueryValue(0, 1);
                        $oc3 = $Obj->GetQueryValue(0, 2);
                        $oc4 = 0;

                        $date = $Obj->GetQueryValue(0, 3);
                        $days = intval($Obj->GetQueryValue(0, 4));
                        $next = ($Obj->GetQueryValue(0, 5));

                        if($days >= 30) {
                                $oc1 = 0;
                                $oc2 = 0;
                                $oc3 = 0;
                        }

?>



        <script type='text/javascript' src='js/pixi.min.js'></script>
        <script type='text/javascript' src='js/main.js'></script>


                                <?php

                                        $occorrency = array(
                                                            "Unha solta",
"A unha de um dedo do pé pode acidentalmente se soltar se for atingida
por alguma coisa dura, como derrubar um objeto pesado sobre ela. Algumas
vezes, ela não se solta completamente de uma vez, mas vagarosamente se
solta do dedo. A unha crescerá novamente, mas até lá você precisa cuidar
do dedo sem unha para que ele não se infecte ou machuque ainda mais.",
"unha.png", "Você tem alguma unha solta nos pés? Em qual? Em qual dedo?",
                                                            "Dedos em
martelo", "É uma deformidade do pé em que os dedos estão dobrados nas
articulações mais distantes das unhas e esticados nas articulações mais
próximas das unhas (então ela fica voltada para cima).",
"dedo_martelo.png", "Você tem dedos em martelo? Em qual pé?",
                                                            "Dedos em
garra", "É uma deformidade do pé em que os dedos estão dobrados por
inteiro, ficando as unhas voltadas para frente.", "garras.png", "Você tem
dedos em garras? Em qual pé?",
                                                            "Joanete -
Hálux Valgo", "É uma deformidade do pé, mais conhecida como joanete, em
que o dedão (hálux) encontra-se desviado e vai ao encontro aos outros
dedos menores.", "halux.png", "Você tem Joanente (hálux Valgo)? Em qual
pé?",


                                                            "Dor",
"Incômodo, sem motivo físico aparente, que provoca a sensação de mal
estar, mesmo sem contato física", "dor.png", "Você sente dor nos pés? Em
qual? Onde é a dor?",
                                                            "Calos", "São
sinais que nos dizem que o pé está tendo pontos de pressão de uma forma
desigual. O corpo reage a esses pontos de maior pressão, como uma forma
de defesa, produzindo mais queratina, uma substância bastante presente na
planta (sola) do pé, mas que, quando aumentada, deixa a pele mais grossa
nesses locais.", "calo.png", "Você tem calos em algum dos pés? Em qual?
Onde estão os calos?",
                                                            "Bolha","É
uma bolsa de água localizada sobre a pele. Ocorre um aumento do espaço
```

entre as camadas da pele, gerado pelo deslocamento entre elas e geralmente preenchido com líquido incolor. Esse deslocamento ocorre muitas vezes por atrito excessivo, como um sapato muito apertado ou muito largo.", "bolha.png", "Você tem bolhas em algum dos pés? Em qual? Onde estão as bolhas?",

"Rachaduras", "São fissuras (pequenos cortes) nos pés, geralmente com a sensação de estarem secos. Ocorrem pelo aumento de espessura da pele e a consequente perda de elasticidade, podendo inclusive, ser bastante dolorosas e causar sangramentos. Elas surgem na maioria das vezes nos calcanhares, mas podem surgir em outras áreas.", "rachadura.png", "Você tem rachaduras em algum dos pés? Em qual? Onde estão as rachaduras?",

"Micoses", "São infecções causadas por fungos que, nos pés, pode afetar a pele (principalmente entre os dedos) e as unhas. Na pele são percebidas manchas brancas ou vermelhas, além de pequenas vesículas (bolhas cheias de líquido) e prurido (coceira). Quando descamam, podem gerar fissuras dolorosas. Nas unhas, as manifestações podem ser manchas brancas na superfície ou embaixo da unha e alterações da coloração, que vão desde branco-amarelado até o acastanhado ou preto.", "micose.png", "Você tem micose em algum dos pés? Em qual? Onde estão as micoses?",

"Olho de Peixe", "O Olho de Peixe é o nome popular de uma enfermidade que tem a aparência de um calo e origina-se de uma verruga. É um 'ferimento' profundo que aponta apenas uma pontinha preta para fora da pele, mas na verdade está bem profundo.", "olho_peixe.png", "Você tem Olho de Peixe nos pés? Em qual? Onde?",

"Pús", "É uma secreção (líquido seroso) de coloração amarelada ou amarelo-esverdeada, geralmente malcheirosa. Ela contém restos de células, proteínas e bactérias envolvidas num processo infeccioso. Procure atendimento médico. Pode representar uma condição grave e que inspira cuidados.", "pus.png", "Há pús em seus pés? Em qual? Onde está o pús?",

"Ferida aberta", "É qualquer interrupção da integridade e continuidade da pele. A pele é uma importante barreira protetora contra microrganismos causadores de doenças. Por isso quando há uma ferida abeta, o risco de infecções aumenta.", "ferida.png", "Você tem feridas em algum dos pés? Em qual? Onde estão as feridas?",

"Saída Líquido", "Se houver saída de líquido de qualquer cor (vermelha, amarela ou transparente), pode significar uma infecção. Procure atendimento médico. Pode representar uma condição grave e que inspira cuidados.", "liquido.png", "Há saída de líquido nos seus pé? Em qual? Onde estão as saídas de líquido?",

"Sangramento", "Se houver saída de sangue em algum local do pé, procure atendimento médico. Pode representar uma condição grave e que inspira cuidados.", "sangramento.png", "Há sangramento nos seus pé? Em qual? Onde está o sangramento?",

"Pé com coloração preta", "O pé de coloração preta indica que já houve morte das células do tecido por falta de oxigenação. Procure atendimento médico. Pode representar uma condição grave e que inspira cuidados.", "preto.png", "Há áreas pretas no seu pé? Em qual? Onde está a coloração preta?",

"Pé com coloração vermelha", "A vermelhidão (ou rubor) é um dos primeiros sinais de inflamação. Portanto, se presente, o pé está provavelmente na fase aguda de um processo inflamatório, decorrente de alguma agressão ao tecido (seja por infecção ou não). Procure atendimento médico. Pode representar uma condição grave e que inspira cuidados.", "vermelho.png", "Há áreas vermelhas no seu pé? Em qual? Onde está a coloração vermelha?",

```
                                                            "Pé com
coloração branca", "Juntamente com sensação de queda da temperatura, o pé
de coloração mais branca que o normal indica diminuição do fluxo
sanguíneo. Procure atendimento médico. É uma situação de emergência, pois
pode haver obstrução de alguma importante artéria.", "branco.png", "Há
coloração branca no seu pé? Em qual? Onde está a coloração branca?"


                                        );

                                ?>


<div  class="content_row default-style no-composer overlap fullwidth">
        <div class="content_row_wrapper clearfix ">
                <div class="col col-sm-12 col-xs-12 " style='min-height:
800px;'>
                        <div class="single post-1872 staff type-staff
status-publish has-post-thumbnail hentry" id="person-1872">


                        <div id='etapa1' style='display: <?php if($oc1 ==
0) echo "inline"; else echo "none"; ?>'>

                                <h2>Questionário sobre ocorrências - <b
style='color: #428BCA;'>Etapa 1 de 3</b></h2><br/><br/>


                                <p><b>Esse questionário será o mapa dos
seus pés e nos ajudará a pescrever os melhores exercícios</b>, além de
servir como apoio para descrever o grau de neuropatia do indivíduo. É
importante relembrar que ele não dispensa uma visita com profissionais de
saúde da área (médicos, fisioterapeutas, enfermeiros, terapeutas
ocupacionais, etc).</p>
                                <br/><br/><b  style='color:
#428BCA;'>Seja paciente e marque cuidadosamente as
ocorrências.</b><br/><hr/><br/>




                                <?php
                                        for($i=0; $i<3; $i++)
                                        {
                                           $pos = $i+1;
                                           if($occorrency[$i*4+2] != "")
                                                echo "<img
src='files/3d_occorency/".$occorrency[$i*4+2]."' style='float: left;
width: 200px;' />";
                                           echo
"<br/><h2>".$occorrency[$i*4].":</h2>".$occorrency[$i*4+1]."<div
class='clear'></div><br/><br/><h4><span>$pos -
".$occorrency[$i*4+3]."</span></h4><br/>";

                                           echo "
                                                <label
for='oc_".$pos."_fl'><input type='checkbox'
onclick=\"javascript:if(this.checked)
jQuery('#op_l_$pos').css('display','inline'); else
jQuery('#op_l_$pos').css('display','none');\" id='oc_".$pos."_fl' /> Pé
esquerdo</label>
                                                <div style='display:
none; color: #428BCA;' id='op_l_".$pos."'>
```

```
                                                            <label 
for='fl_".$pos."_d1' style='margin-left: 10px;'><input type='checkbox' 
id='fl_".$pos."_d1' /> Dedão (Halux)</label>
                                                            <label 
for='fl_".$pos."_d2' style='margin-left: 10px;'><input type='checkbox' 
id='fl_".$pos."_d2' /> 2º Dedo</label>
                                                            <label 
for='fl_".$pos."_d3' style='margin-left: 10px;'><input type='checkbox' 
id='fl_".$pos."_d3' /> 3º Dedo</label>
                                                            <label 
for='fl_".$pos."_d4' style='margin-left: 10px;'><input type='checkbox' 
id='fl_".$pos."_d4' /> 4º Dedo</label>
                                                            <label 
for='fl_".$pos."_d5' style='margin-left: 10px;'><input type='checkbox' 
id='fl_".$pos."_d5' /> 5º Dedo (Mindinho)</label>
                                                    </div>
                                                    <br/>

                                                    <label 
for='oc_".$pos."_fr'><input type='checkbox' id='oc_".$pos."_fr' 
onclick=\"javascript:if(this.checked) 
jQuery('#op_r_".$pos."').css('display','inline'); else 
jQuery('#op_r_".$pos."').css('display','none');\" /> Pé direito</label>
                                                    <div style='display: 
none; color: #428BCA;' id='op_r_".$pos."'>
                                                            <label 
for='fr_".$pos."_d1' style='margin-left: 27px;'><input type='checkbox' 
id='fr_".$pos."_d1' /> Dedão (Halux)</label>
                                                            <label 
for='fr_".$pos."_d2' style='margin-left: 10px;'><input type='checkbox' 
id='fr_".$pos."_d2' /> 2º Dedo</label>
                                                            <label 
for='fr_".$pos."_d3' style='margin-left: 10px;'><input type='checkbox' 
id='fr_".$pos."_d3' /> 3º Dedo</label>
                                                            <label 
for='fr_".$pos."_d4' style='margin-left: 10px;'><input type='checkbox' 
id='fr_".$pos."_d4' /> 4º Dedo</label>
                                                            <label 
for='fr_".$pos."_d5' style='margin-left: 10px;'><input type='checkbox' 
id='fr_".$pos."_d5' /> 5º Dedo (Mindinho)</label>

        </div><br/><hr/><br/><br/><br/>
                                                    ";
                                            }


                                            for($i=3; $i<4; $i++)
                                            {
                                               $pos = $i+1;
                                               if($occorrency[$i*4+2] != "")
                                                    echo "<img 
src='files/3d_occorency/".$occorrency[$i*4+2]."' style='float: left; 
width: 200px;' />";
                                               echo 
"<br/><h2>".$occorrency[$i*4].":</h2>".$occorrency[$i*4+1]."<div 
class='clear'></div><br/><br/><h4><span>4 - 
".$occorrency[$i*4+3]."</span></h4><br/>";

                                               echo "
                                                    <label 
for='oc_".$pos."_fl'><input type='checkbox' id='oc_".$pos."_fl' /> Dedão 
do Pé esquerdo</label>
                                                    <br/>
```

```
                                                <label
for='oc_".$pos."_fr'><input type='checkbox' id='oc_".$pos."_fr'  /> Dedão
do Pé direito</label>
                                                <br/><hr/><br/><br/><br/>
                                                ";
                                        }
                                ?>

                                <div class="clear"></div><br/>
                                <div id='msg' style='color:
#CC2233;'></div>
                                <br/>
                                <input class="button submit"
onclick="javascript:ValidateStep1();" value="Proxima Etapa"
type="button">

                        </div>



                        <div id='etapa2' style='display: <?php if($oc1 ==
1 && $oc2 == 0) echo "inline"; else echo "none"; ?>'>

                                <h2>Questionário sobre ocorrências - <b
style='color: #428BCA;'>Etapa 2 de 3</b></h2><br/><br/>
                                <b>Para responder as perguntas da segunda
etapa, por favor considere as imagens abaixo:</b>
                                <table>
                                        <tr>
                                                <td style='text-align:
center;'><img src="files/3d_occorency/superior.png" style='width: 200px;'
/><br/><strong>Região Superior</strong></td>
                                                <td style='text-align:
center;'><img src="files/3d_occorency/inferior.png" style='width: 200px;'
/><br/><strong>Região Inferior</strong></td>
                                                <td style='text-align:
center;'><img src="files/3d_occorency/posterior.png" style='width:
200px;' /><br/><strong>Região Posterior</strong></td>
                                        </tr>
                                </table>
                                <br/><br/><b  style='color:
#428BCA;'>Seja paciente e marque cuidadosamente as
ocorrências.</b><br/><hr/><br/>




                                <?php
                                        for($i=4; $i<10; $i++)
                                        {
                                           $pos = $i+1;
                                           if($occorrency[$i*4+2] != "")
                                                echo "<img
src='files/3d_occorency/".$occorrency[$i*4+2]."' style='float: left;
width: 200px;' />";
                                           echo
"<br/><h2>".$occorrency[$i*4].":</h2>".$occorrency[$i*4+1]."<div
class='clear'></div><br/><br/><h4><span>$pos -
".$occorrency[$i*4+3]."</span></h4><br/>";

                                           echo "
                                                <label
for='oc_".$pos."_fl'><input type='checkbox'
onclick=\"javascript:if(this.checked)
jQuery('#op_l_$pos').css('display','inline'); else
```

```
jQuery('#op_l_$pos').css('display','none');\" id='oc_".$pos."_fl' /> Pé
esquerdo</label>
                                                <div style='display:
none; color: #428BCA;' id='op_l_".$pos."'>
                                                        <label
for='fl_".$pos."_d1' style='margin-left: 10px;'><input type='checkbox'
id='fl_".$pos."_d1' /> Dedão</label>
                                                        <label
for='fl_".$pos."_d2' style='margin-left: 10px;'><input type='checkbox'
id='fl_".$pos."_d2' /> 2º Dedo</label>
                                                        <label
for='fl_".$pos."_d3' style='margin-left: 10px;'><input type='checkbox'
id='fl_".$pos."_d3' /> 3º Dedo</label>
                                                        <label
for='fl_".$pos."_d4' style='margin-left: 10px;'><input type='checkbox'
id='fl_".$pos."_d4' /> 4º Dedo</label>
                                                        <label
for='fl_".$pos."_d5' style='margin-left: 10px;'><input type='checkbox'
id='fl_".$pos."_d5' /> Mindinho</label>
                                                        <label
for='fl_".$pos."_d6' style='margin-left: 10px;'><input type='checkbox'
id='fl_".$pos."_d6' /> Região superior</label>
                                                        <label
for='fl_".$pos."_d7' style='margin-left: 10px;'><input type='checkbox'
id='fl_".$pos."_d7' /> Região inferior</label>
                                                        <label
for='fl_".$pos."_d8' style='margin-left: 10px;'><input type='checkbox'
id='fl_".$pos."_d8' /> Região Posterior</label>
                                                </div>
                                                <br/>

                                                <label
for='oc_".$pos."_fr'><input type='checkbox' id='oc_".$pos."_fr'
onclick=\"javascript:if(this.checked)
jQuery('#op_r_".$pos."').css('display','inline'); else
jQuery('#op_r_".$pos."').css('display','none');\" /> Pé direito</label>
                                                <div style='display:
none; color: #428BCA;' id='op_r_".$pos."'>
                                                        <label
for='fr_".$pos."_d1' style='margin-left: 27px;'><input type='checkbox'
id='fr_".$pos."_d1' /> Dedão</label>
                                                        <label
for='fr_".$pos."_d2' style='margin-left: 10px;'><input type='checkbox'
id='fr_".$pos."_d2' /> 2º Dedo</label>
                                                        <label
for='fr_".$pos."_d3' style='margin-left: 10px;'><input type='checkbox'
id='fr_".$pos."_d3' /> 3º Dedo</label>
                                                        <label
for='fr_".$pos."_d4' style='margin-left: 10px;'><input type='checkbox'
id='fr_".$pos."_d4' /> 4º Dedo</label>
                                                        <label
for='fr_".$pos."_d5' style='margin-left: 10px;'><input type='checkbox'
id='fr_".$pos."_d5' /> Mindinho</label>
                                                        <label
for='fr_".$pos."_d6' style='margin-left: 10px;'><input type='checkbox'
id='fr_".$pos."_d6' /> Região superior</label>
                                                        <label
for='fr_".$pos."_d7' style='margin-left: 10px;'><input type='checkbox'
id='fr_".$pos."_d7' /> Região inferior</label>
                                                        <label
for='fr_".$pos."_d8' style='margin-left: 10px;'><input type='checkbox'
id='fr_".$pos."_d8' /> Região Posterior</label>

        </div><br/><hr/><br/><br/><br/>
                                                ";
```

```
                                    }

                            ?>

                            <div class="clear"></div><br/>
                            <div id='msg2' style='color:
#CC2233;'></div>
                            <br/>
                            <input class="button submit"
onclick="javascript:ValidateStep2();" value="Proxima Etapa"
type="button">

                        </div>




                        <div id='etapa3' style='display: <?php if($oc2 ==
1 && $oc3 == 0) echo "inline"; else echo "none"; ?>'>

                            <h2>Questionário sobre ocorrências - <b
style='color: #428BCA;'>Etapa 3 de 3</b></h2><br/><br/>
                            <b>Essa é a última parte do questionário
com marcação. Por favor, informe honestamente as respostas abaixo.
Respostas erradas poderá levar a uma piora da situação do seu pé.</b>
                            <br/><br/><b  style='color:
#428BCA;'>Seja paciente e marque cuidadosamente as
ocorrências.</b><br/><hr/><br/>


                            <?php
                                    for($i=10; $i<17; $i++)
                                    {
                                       $pos = $i+1;
                                       if($occorrency[$i*4+2] != "")
                                            echo "<img
src='files/3d_occorency/".$occorrency[$i*4+2]."' style='float: left;
width: 200px;' />";
                                       echo
"<br/><h2>".$occorrency[$i*4]."</h2>".$occorrency[$i*4+1]."<div
class='clear'></div><br/><br/><h4><span>$pos -
".$occorrency[$i*4+3]."</span></h4><br/>";

                                       echo "
                                            <label
for='oc_".$pos."_fl'><input type='checkbox'
onclick=\"javascript:if(this.checked)
jQuery('#op_l_$pos').css('display','inline'); else
jQuery('#op_l_$pos').css('display','none');\" id='oc_".$pos."_fl' /> Pé
esquerdo</label>
                                            <div style='display:
none; color: #428BCA;' id='op_l_".$pos."'>
                                                    <label
for='fl_".$pos."_d1' style='margin-left: 10px;'><input type='checkbox'
id='fl_".$pos."_d1' /> Dedão</label>
                                                    <label
for='fl_".$pos."_d2' style='margin-left: 10px;'><input type='checkbox'
id='fl_".$pos."_d2' /> 2º Dedo</label>
                                                    <label
for='fl_".$pos."_d3' style='margin-left: 10px;'><input type='checkbox'
id='fl_".$pos."_d3' /> 3º Dedo</label>
                                                    <label
for='fl_".$pos."_d4' style='margin-left: 10px;'><input type='checkbox'
id='fl_".$pos."_d4' /> 4º Dedo</label>
```

```
                                                        <label
for='fl_".$pos."_d5' style='margin-left: 10px;'><input type='checkbox'
id='fl_".$pos."_d5' /> Mindinho</label>
                                                        <label
for='fl_".$pos."_d6' style='margin-left: 10px;'><input type='checkbox'
id='fl_".$pos."_d6' /> Região superior</label>
                                                        <label
for='fl_".$pos."_d7' style='margin-left: 10px;'><input type='checkbox'
id='fl_".$pos."_d7' /> Região inferior</label>
                                                        <label
for='fl_".$pos."_d8' style='margin-left: 10px;'><input type='checkbox'
id='fl_".$pos."_d8' /> Região Posterior</label>
                                                </div>
                                                <br/>

                                                <label
for='oc_".$pos."_fr'><input type='checkbox' id='oc_".$pos."_fr'
onclick=\"javascript:if(this.checked)
jQuery('#op_r_".$pos."').css('display','inline'); else
jQuery('#op_r_".$pos."').css('display','none');\" /> Pé direito</label>
                                                <div style='display:
none; color: #428BCA;' id='op_r_".$pos."'>
                                                        <label
for='fr_".$pos."_d1' style='margin-left: 27px;'><input type='checkbox'
id='fr_".$pos."_d1' /> Dedão</label>
                                                        <label
for='fr_".$pos."_d2' style='margin-left: 10px;'><input type='checkbox'
id='fr_".$pos."_d2' /> 2º Dedo</label>
                                                        <label
for='fr_".$pos."_d3' style='margin-left: 10px;'><input type='checkbox'
id='fr_".$pos."_d3' /> 3º Dedo</label>
                                                        <label
for='fr_".$pos."_d4' style='margin-left: 10px;'><input type='checkbox'
id='fr_".$pos."_d4' /> 4º Dedo</label>
                                                        <label
for='fr_".$pos."_d5' style='margin-left: 10px;'><input type='checkbox'
id='fr_".$pos."_d5' /> Mindinho</label>
                                                        <label
for='fr_".$pos."_d6' style='margin-left: 10px;'><input type='checkbox'
id='fr_".$pos."_d6' /> Região superior</label>
                                                        <label
for='fr_".$pos."_d7' style='margin-left: 10px;'><input type='checkbox'
id='fr_".$pos."_d7' /> Região inferior</label>
                                                        <label
for='fr_".$pos."_d8' style='margin-left: 10px;'><input type='checkbox'
id='fr_".$pos."_d8' /> Região Posterior</label>

        </div><br/><hr/><br/><br/><br/>
                                                ";
                                        }

                                ?>

                                <div class="clear"></div><br/>
                                <div id='msg3' style='color:
#CC2233;'></div>
                                <br/>
                                <input class="button submit"
onclick="javascript:ValidateStep3();" value="Proxima Etapa"
type="button">

                        </div>
```

```
                        <div id='etapa5'  style='display: <?php if($oc3
== 1) echo "inline"; else echo "none"; ?>; height: 800px;'>
                                        <h2>Parabéns! Você respondeu em
<?php echo "<b style='color: #428BCA;'>$date</b>"; ?> todos os
questionários de ocorrência!</h2><br/><br/>

                                        <b style="color:
#428BCA;">Próximos passos</b><br/>
                                        &bull; Exercícios;<br/>
                                        &bull; Tirar suas dúvidas com os
especialistas;<br/>

                                        <br/><br/><h2>Sua próxima
avaliação: <b style="color: #428BCA;"><?php echo $next; ?></b> <?php
$days = 30-intval($days); if($days < 0) $days = 0; echo " - Falta(m)
$days dia(s)"; ?></h2>
                        </div>





                        </div>
                </div>
        </div>
</div>









<script type="text/javascript">
        jQuery(document).ready(function() { Preload(); });
</script>




<div  class="content_row row vc_row wpb_row vc_row-fluid default-style
fullwidth" style="position:relative;overflow:hidden;">
        <div class="rt-parallax-background" data-rt-parallax-
direction="1" data-rt-parallax-effect="horizontal" style="background-
image: url(images/portfolio_header.jpg);background-repeat:
repeat;background-size: cover;background-position: right
top;width:100%;height:100%;top:0;"></div>
        <div class="content_row_wrapper  default" style="padding-
top:25px;padding-bottom:15px;">
                <div class="vc_col-sm-12 wpb_column vc_column_container"
>
                        <div class="wpb_wrapper">
                                <div class="rt_heading_wrapper style-4">
                                        <h1 class="rt_heading  style-4"
>Termos de uso do SoPed</h1>
                                </div>
                        </div>
                </div>
        </div>
</div>

<div  class="content_row row vc_row wpb_row vc_row-fluid default-style
fullwidth border_grid fixed_heights" >
        <div class="content_row_wrapper  default" style="padding-
top:50px;padding-bottom:50px; min-height: 500px;">
                <div class="vc_col-sm-12 wpb_column vc_column_container"
>
```

```
                        <div class="wpb_wrapper">
                                <div class="rt_heading_wrapper style-6">
                                        <h4 class="rt_heading  style-6"
><span class="icon-glyph-15 heading_icon"></span>TERMO DE USO E
SERVIÇO</h4>
                                </div>
                                <div class="wpb_text_column
wpb_content_element ">
                                        <div class="wpb_wrapper">
                                                <p>Última Atualização:
Julho de 2017</b></p>
                                        </div>
                                </div>


                                <p>
Estes são os termos de uso do SOPeD; usando este software, você concorda
em aceitar e seguir estes termos.
<hr><br/>
<h2>Dados Pessoais que Coletamos</h2>
O SOPED é uma ferramenta oriunda de muito estudo para proporcionar à
você, usuário, condições para manter os seus pés saudáveis. Alguns desses
dados são fornecidos diretamente por você, por exemplo, quando você cria
a sua conta, passamos a ter registrado os seus dados cadastrais assim
como as avaliações feitas por você. Ainda é possível registrar e guardar
dados como desempenho na ferramenta e acompanhar a sua evolução.
Gostaríamos de deixar claro que mesmo que algum de seus dados seja
utilizado para pesquisa, a sua identidade será mantida em sigilo.
<br/><br/>

<hr/><br/>
<h2>Site personalizado </h2>
Estou ciente de que o SOPeD é uma ferramenta que personaliza uma rotina
de exercícios  para  pés  e  tornozelos,  visando  melhorias
individuais.  O  meu  não comprometimento  ou  descaso  no  momento  de
execução  de  cada  exercício implicará  no  não  sucesso  para  a  minha
evolução.  Estou  ciente  também  que cada  pessoa  responde  de
maneiras  diferentes  a  cada  tratamento  e  que  cada pessoa  está  num
estágio  diferente  da  doença,  portanto  o  fato  de  fazer
corretamente  os  exercícios  aqui  propostos,  assim  como  uma
assiduidade  não me garantem a isenção de complicações futuras da
neuropatia, caso ainda não seja neuropata. Este software não substitui a
consulta regular ao fisioterapeuta e ao médico.<br/><br/>

<hr/><br/>
<h2>Termos de aceitação</h2>
Você  não  pode  usar  nosso  software  para  difamar  ou  prejudicar
terceiros  ou  o próprio  software.  Tentativas  de  fraudes  serão
punidas  e  se  necessário  dados serão fornecidos as autoridades caso
sejam requisitados.
<br/><br/>
<hr/><br/>

<h2>Acesso restrito</h2><br/>
Algumas  áreas  de  nossa  ferramenta  têm  acesso  restrito  devido  ao
fato  da personalização. Caso você não tenha condições para manuseá-lo,
peça ajuda a um cuidador, familiar ou profissional da área da saúde. O
nome de usuário e senha  que  lhe  fornecemos  devem  ser  para  seu
próprio  uso  ficando  sob  sua responsabilidade  a  divulgação  a
terceiros.  Nos  reservamos  o  direito  de  banir sua conta sem prévio
aviso caso desrespeite nossos termos de uso.

<hr/><br/>
<h2>Conteúdo de usuário</h2>
```

```
Nos reservamos o direito de modificar algum conteúdo caso necessário. Se
for identificado que o usuário está agindo de má fé, com o intuito de
prejudicar ou espionar  tanto  o software  quanto  os  membros,  este
usuário  terá  sua  conta banida.
<br/><br/>

<hr/><br/>
<h2>Garantias</h2>
Não  garantimos  que  seu  problema  seja  rapidamente  solucionado.
Como  já foi dito,  cada  caso  responde  diferente  a  um  tipo  de
tratamento  e  isto  depende muito  do  quadro  que  o  paciente se
encontra  e  de  seu  engajamento  no tratamento proposto.
<hr/>

<br/><br/>

<hr/><br/>
<h2>Também não podemos garantir que</h2>
&bull; Este  software  ficará  100%  disponível  e  com  todas  as
funcionalidades  em perfeito estado sempre; <br/><br/>
&bull; Você seja "curado"; <br/><br/>
&bull;  Você  esteja  isento  a  danos  causados  pelo  mau   uso   e
interpretação inadequada dos exercícios. Em caso de dúvidas, contate-nos!
Esta política foi atualizada em, 09/07/2017<br/><br/>
<hr/>


                                </p>
                        </div>
                </div>
        </div>
</div>

<link rel="stylesheet" href="css/introjs.css" type="text/css"
media="screen" />

<script src="js/intro.js"></script>

<div  class="content_row row vc_row wpb_row vc_row-fluid alt-style-1
fullwidth border_grid fixed_heights" >
        <div class="content_row_wrapper  default" style="padding-
top:50px;padding-bottom:50px; min-height: 500px;">
                <div class="vc_col-sm-12 wpb_column vc_column_container"
>
                        <div class="wpb_wrapper">


                                   <div class="jumbotron">
        <h1 data-step="1" data-intro="Esse é um aviso de
tutorial!">Exemplo com Bullet</h1>
        <p class="lead" data-step="4" data-intro="Último passo...">Esse
exemplo é autocarregável.</p>
        <a class="btn btn-large btn-success" href="javascript:void(0);"
onclick="javascript:introJs().setOption({ 'nextLabel': 'Próximo',
'prevLabel': 'Anterior', 'skipLabel': 'Pular tutorial', 'doneLabel':
'Finalizar' }).start();">Ver novamente</a>
      </div>

      <hr>

      <div class="row-fluid marketing">
        <div class="span6" data-step="2" data-intro="Ok, Seria assim o
funcionamento?" data-position='top'>
          <h4>Parte 1</h4>
```

```
          <p>Lorem ipsum dolor sit amet, consectetur adipiscing elit.
Duis mollis augue a neque cursus ac blandit orci faucibus. Phasellus nec
metus purus.</p>

          <h4>Parte 2</h4>
          <p>Lorem ipsum dolor sit amet, consectetur adipiscing elit.
Duis mollis augue a neque cursus ac blandit orci faucibus. Phasellus nec
metus purus.</p>

          <h4>Parte 3</h4>
          <p>Lorem ipsum dolor sit amet, consectetur adipiscing elit.
Duis mollis augue a neque cursus ac blandit orci faucibus. Phasellus nec
metus purus.</p>
          </div>

        <div class="span6" data-step="3" data-intro="Podemos colocar até
terminar"  data-position='top'>
          <h4>Parte 4</h4>
          <p>Lorem ipsum dolor sit amet, consectetur adipiscing elit.
Duis mollis augue a neque cursus ac blandit orci faucibus. Phasellus nec
metus purus.</p>

          <h4>Parte 5</h4>
          <p>Lorem ipsum dolor sit amet, consectetur adipiscing elit.
Duis mollis augue a neque cursus ac blandit orci faucibus. Phasellus nec
metus purus.</p>

          <h4>Parte 6</h4>
          <p>Lorem ipsum dolor sit amet, consectetur adipiscing elit.
Duis mollis augue a neque cursus ac blandit orci faucibus. Phasellus nec
metus purus.</p>

        </div>
      </div>



                  </div>
            </div>
        </div>
</div>

<script>
                jQuery(document).ready(function() {
                        introJs().setOption({'nextLabel': 'Próximo',
'prevLabel': 'Anterior', 'skipLabel': 'Pular tutorial', 'doneLabel':
'Finalizar'}).start();
                });
</script>
```

```
<link rel='stylesheet' href='css/myform.css' type='text/css' media='all'
/>

<style type="text/css">
#myform input[type="text"],
#myform input[type="date"],
#myform input[type="datetime"],
#myform input[type="email"],
#myform input[type="number"],
#myform input[type="search"],
#myform input[type="time"],
#myform input[type="url"],
```

```
#myform input[type="password"],
#myform textarea,
#myform select {

    border-top: 1px solid #f0f0f0;
    border-left: 1px solid #f0f0f0;
    border-bottom: 1px solid #FAFAFA;
    border-right: 1px solid #FAFAFA;
    background: #e0e0e0;
}

.alt-style-1 input[type="button"]:hover {background-color: #FC9D0D;}
.alt-style-1 input[type="button"] {background-color: #428bca; font-
weight: bold;}

.alt-style-1 input[type="button"]:hover.extra  {background-color: #BBB;}
.alt-style-1 input[type="button"].extra  {background-color: #888; font-
weight: bold;}
b span{color: #428bca;}



.default-style input[type="button"]:hover {background-color: #FC9D0D;}
.default-style input[type="button"] {background-color: #428bca; font-
weight: bold;}

.default-style input[type="button"]:hover.extra  {background-color:
#BBB;}
.default-style input[type="button"].extra  {background-color: #888; font-
weight: bold;}

</style>


        <?php include("php/pages/internal.php"); ?>




<script type="text/javascript">


        function enviarMsg(id) {

                        jQuery.ajax({
                                type: "POST",
                                url: "php/pages/solicitar.php",
                                data: {action : "sol", id: id, pedido :
jQuery('#pedido').val()}
                        })
                        .done(function(html) {
                                jQuery("#msg").append("<strong
class='you'>JANE SUELEN:</strong> "+jQuery('#pedido').val()+"<br/>");
                                jQuery('#pedido').val("");
                        });

        }
</script>

        <style>
                .icircle img {-webkit-border-radius: 124px;
                        -moz-border-radius: 124px;
                        border-radius: 124px;
                        width: 120px;}
                .wpb_wrapper input[type="button"] {background: #428BCA;}
```

```
                .wpb_wrapper input[type="button"]:hover {background:
#317AB9;}
        </style>



<div  class="content_row row vc_row wpb_row vc_row-fluid alt-style-1
fullwidth border-top border-bottom" >
        <div class="content_row_wrapper  default" style="padding-
top:60px;padding-bottom:60px;">
                <div class="vc_col-sm-12 wpb_column vc_column_container"
>
                        <div class="wpb_wrapper">




<img src="images/t1.png" style="float: right; width: 80px; border-radius:
200px; border: 2px solid #FFF;" />
<h1>Conversa com Alisson Amorim</h1>
<div class="clerfix"></div><br/><br/>

                                <div style="border: 2px solid #CCC;
overflow-y: auto; height: 200px; padding: 20px;" id='talk'>
                                        <!--<strong class="me">Alisson
Amorim - 21:50: </strong> Oi Jane, recebeu minha mensagem?<br/>-->
                                        <div id='msg'></div>

                                </div>
                                <div class="clerfix"></div><br/>
                                <label>Enviar mensagem</label>
                                <textarea style="width: 100%; height:
50px; background: #FFF;" id='pedido'></textarea>

                                <br/><br/><input type="button"
onclick="javascript:enviarMsg();" value="Enviar" />
                                <input type="button"
onclick="javascript:alert('Você não tem pontos suficientes.');"
style="margin-right: 200px;" value="Anexar arquivo" />
                        </div>
                </div>
        </div>
</div>


<script type="text/javascript">
        jQuery("#talk").scrollTop(jQuery("#talk")[0].scrollHeight);
</script>
<?php


        if(isset($_POST['action'])) {

                if($_POST['action'] == "sol") {
                        //require_once("../email.php");
                        //SendPHPMail("alisson-amorim@hotmail.com","","",
"", "Mensagem", $_POST["pedido"]);
                }

                return;
        }

?>
```

```
<link rel='stylesheet' href='css/myform.css' type='text/css' media='all'
/>

<style type="text/css">
#myform input[type="text"],
#myform input[type="date"],
#myform input[type="datetime"],
#myform input[type="email"],
#myform input[type="number"],
#myform input[type="search"],
#myform input[type="time"],
#myform input[type="url"],
#myform input[type="password"],
#myform textarea,
#myform select {

    border-top: 1px solid #f0f0f0;
    border-left: 1px solid #f0f0f0;
    border-bottom: 1px solid #FAFAFA;
    border-right: 1px solid #FAFAFA;
    background: #e0e0e0;
}

.alt-style-1 input[type="button"]:hover {background-color: #FC9D0D;}
.alt-style-1 input[type="button"] {background-color: #428bca; font-
weight: bold;}

.alt-style-1 input[type="button"]:hover.extra  {background-color: #BBB;}
.alt-style-1 input[type="button"].extra  {background-color: #888; font-
weight: bold;}
b span{color: #428bca;}



.default-style input[type="button"]:hover {background-color: #FC9D0D;}
.default-style input[type="button"] {background-color: #428bca; font-
weight: bold;}

.default-style input[type="button"]:hover.extra  {background-color:
#BBB;}
.default-style input[type="button"].extra  {background-color: #888; font-
weight: bold;}

</style>


        <?php include("php/pages/internal.php"); ?>



<script type="text/javascript">

                function solicitarAmizade(id)
                {
                        jQuery("#saida").html(ShowInfo("Aguarde..."));

                        if(jQuery('#pedido').val() == "") {
                                alert("Escreva um pedido.");
                                return;
                        }

                        jQuery.ajax({
                                type: "POST",
                                url: "php/pages/solicitar.php",
```

```
                                data: {action : "sol", id: id, pedido :
jQuery('#pedido').val()}
                        })
                        .done(function(html) {

                                        alert("Pedido enviado com
sucesso!");
                                        window.location =
'index.php?page=social'


                        });
                }

</script>


        <style>
                .icircle img {-webkit-border-radius: 124px;
                        -moz-border-radius: 124px;
                        border-radius: 124px;
                        width: 120px;}
                .wpb_wrapper input[type="button"] {background: #428BCA;}
                .wpb_wrapper input[type="button"]:hover {background:
#317AB9;}
        </style>


<div  class="content_row default-style no-composer overlap fullwidth">
        <div class="content_row_wrapper clearfix ">
                <div class="vc_col-sm-12 wpb_column vc_column_container"
>
                        <div class="wpb_wrapper">


                                <label>Escreva uma mensagem para pedir a
solicitação</label>
                                <textarea style="width: 100%; height:
200px;" id="pedido"></textarea>

                                <br/><br/><input type="button"
onclick="javascript:solicitarAmizade(<?php echo $_GET["id"]; ?>);"
value="Solicitar amizade" />
                        </div>
                </div>
        </div>
</div>

        <script type="text/javascript"
src="js/plupload/plupload.full.min.js"></script>



<link rel='stylesheet' href='css/myform.css' type='text/css' media='all'
/>

<style type="text/css">
#myform input[type="text"],
#myform input[type="date"],
#myform input[type="datetime"],
#myform input[type="email"],
#myform input[type="number"],
#myform input[type="search"],
#myform input[type="time"],
#myform input[type="url"],
```

```
#myform input[type="password"],
#myform textarea,
#myform select {

    border-top: 1px solid #f0f0f0;
    border-left: 1px solid #f0f0f0;
    border-bottom: 1px solid #FAFAFA;
    border-right: 1px solid #FAFAFA;
    background: #e0e0e0;
}

.alt-style-1 input[type="button"]:hover {background-color: #FC9D0D;}
.alt-style-1 input[type="button"] {background-color: #428bca; font-
weight: bold;}

.alt-style-1 input[type="button"]:hover.extra  {background-color: #BBB;}
.alt-style-1 input[type="button"].extra  {background-color: #888; font-
weight: bold;}
b span{color: #428bca;}



.default-style input[type="button"]:hover {background-color: #FC9D0D;}
.default-style input[type="button"] {background-color: #428bca; font-
weight: bold;}

.default-style input[type="button"]:hover.extra  {background-color:
#BBB;}
.default-style input[type="button"].extra  {background-color: #888; font-
weight: bold;}

</style>


        <?php include("php/pages/internal.php"); ?>




<script type="text/javascript">


        function comprarItem() {

                 alert('Você não tem pontos suficientes.');

        }
</script>

        <style>
                .icircle img {-webkit-border-radius: 124px;
                        -moz-border-radius: 124px;
                        border-radius: 124px;
                        width: 120px;}
                .wpb_wrapper input[type="button"] {background: #428BCA;}
                .wpb_wrapper input[type="button"]:hover {background:
#317AB9;}
        </style>


<div  class="content_row default-style no-composer overlap fullwidth"
style='min-height: 800px;'>
        <div class="content_row_wrapper clearfix ">
                <div class="vc_col-sm-12 wpb_column vc_column_container"
>
```

```
                        <div class="wpb_wrapper">


                                <table style="width: 100%;">
                                        <tr>





                                        <?php

                        require_once('php/MysqlDB.php');
                        $id = $_SESSION['id'];

                        $Obj = new ScriptDB();
                        $Obj->Connect();
                        $Obj->ExecQuery("SELECT name, coins, medals,
trophies, gender, saedd_users.id FROM saedd_users LEFT JOIN saedd_awards
ON saedd_users.id = user_id WHERE saedd_users.id = '$id' ORDER BY
4,3,2,1");

                        $count = $Obj->Get('RecordCount');

                        for($i=0; $i<$count; $i++) {
                           $nome = $Obj->GetQueryValue($i, 0);
                           $coins = $Obj->GetQueryValue($i, 1);
                           $medals = $Obj->GetQueryValue($i, 2);
                           $trophies = $Obj->GetQueryValue($i, 3);
                           $gender = $Obj->GetQueryValue($i, 4);
                           $user = $Obj->GetQueryValue($i, 5);



                           echo "               <td>

        <br/><h2>$nome</h2><br/>
                                                        <img
src='images/coin.png' style='height: 60px;'> x $coins
                                                        <img
src='images/medal.png' style='height: 60px;'> x $medals
                                                        <img
src='images/trophy.png' style='height: 60px;'> x $trophies<br/><br/>
                                                </td>


                                ";

                        }


                        ?>


                                        </tr>

                                </table> <br/><br/>
                        <h1>Loja SOPED</h1><br/>




                        <strong>E-book: Diabetes e saúde</strong>  <img
src="images/coin.png" style="height: 60px;"> x 20<br/><br/>
```

```
<input type="button" onclick="javascript:comprarItem();" value="Comprar"
/>      <hr/>
                        <br/><br/><br/><strong>Documentário: A histórica
da genética e a diabetes</strong>  <img src="images/medal.png"
style="height: 60px;"> x 20<br/><br/>
<input type="button" onclick="javascript:comprarItem();" value="Comprar"
/>       <hr/>

                        <br/><br/><br/><strong>Upgrade: Envie arquivos de
até 2 MBs</strong>  <img src="images/trophy.png" style="height: 60px;"> x
20<br/><br/>
<input type="button" onclick="javascript:comprarItem();" value="Comprar"
/>      <hr/>

                </div>
        </div>
</div>
<div  class="content_row row vc_row wpb_row vc_row-fluid default-style
fullwidth" style="position:relative;overflow:hidden;">
        <div class="rt-parallax-background" data-rt-parallax-
direction="1" data-rt-parallax-effect="horizontal" style="background-
image: url(images/portfolio_header.jpg);background-repeat:
repeat;background-size: cover;background-position: right
top;width:100%;height:100%;top:0;"></div>
        <div class="content_row_wrapper  default" style="padding-
top:25px;padding-bottom:15px;">
                <div class="vc_col-sm-12 wpb_column vc_column_container"
>
                        <div class="wpb_wrapper">
                                <div class="rt_heading_wrapper style-4">
                                        <h1 class="rt_heading  style-4"
>Introdução ao SoPed</h1>
                                </div>
                        </div>
                </div>
        </div>
</div>

<div  class="content_row row vc_row wpb_row vc_row-fluid default-style
fullwidth border_grid fixed_heights" >
        <div class="content_row_wrapper  default" style="padding-
top:50px;padding-bottom:50px; min-height: 500px;">
                <div class="vc_col-sm-12 wpb_column vc_column_container"
>
                        <div class="wpb_wrapper">




        <script type='text/javascript' src='js/pixi.min.js'></script>
        <script type='text/javascript' src='js/avatar.js'></script>


<script type="text/javascript">

        function addoccurencies() {

        }

        jQuery(document).ready(function() {
                        onRunning = addoccurencies;
                        Preload("game");
                });
```

```
</script>



                <canvas id="game"></canvas><br/><br/>


                        </div>
                </div>
        </div>
</div>



<link rel='stylesheet' href='css/myform.css' type='text/css' media='all'
/>

<style type="text/css">
.myform input[type="text"],
.myform input[type="date"],
.myform input[type="datetime"],
.myform input[type="email"],
.myform input[type="number"],
.myform input[type="search"],
.myform input[type="time"],
.myform input[type="url"],
.myform input[type="password"],
.myform textarea,
.myform select {

    border-top: 1px solid #f0f0f0;
    border-left: 1px solid #f0f0f0;
    border-bottom: 1px solid #FAFAFA;
    border-right: 1px solid #FAFAFA;
    background: #e0e0e0;
}

.default-style input[type="button"]:hover {background-color: #FC9D0D;}
.default-style input[type="button"] {background-color: #428bca; font-
weight: bold;}

.default-style input[type="button"]:hover.extra  {background-color:
#BBB;}
.default-style input[type="button"].extra  {background-color: #888; font-
weight: bold;}
</style>


        <script type='text/javascript' src='js/pixi.min.js'></script>
        <script src="js/sound.js"></script>
        <script type='text/javascript' src='js/video.js'></script>









<div  class="content_row default-style no-composer overlap fullwidth"
style='min-height: 1200px;'>
        <div class="content_row_wrapper clearfix ">
```

```
                <div class="col col-sm-12 col-xs-12 ">
                        <div class="single post-1872 staff type-staff
status-publish has-post-thumbnail hentry" id="person-1872">
                                <br/><br/><br/><br/>
                        <h2>Abaular o pé - <span style='color:
#428bca'>Execução 1</span></h2>
                        <p>Sentado, “encurvar” o pé como se fosse
encolhê-lo, mantendo o calcanhar e as pontas dos dedos dos pés no chão.
                                <br/><h4 style='color: #428bca'>1x10
repetições</h4>
                        </p>

                        <div><canvas id="game"></canvas></div>


                        </div>
                </div>

                <div class="col col-sm-4 col-xs-12 ">
                        <div class="single post-1872 staff type-staff
status-publish has-post-thumbnail hentry" id="person-1872">


                        </div>
                </div>

        </div>
</div>






<script type="text/javascript">
        jQuery(document).ready(function() { Preload("game"); });
</script>



<?php

        function ChangeUserAwards($type, $position, $block) {

           $user_id = 1;

           require_once('../MysqlDB.php');
           $Obj = new ScriptDB();
           $sql = "";

           if($position == "")
             $position = "last_position";
           else
             $position = "(CASE WHEN $position = (SELECT MAX(position)
FROM saedd_protocol WHERE user_id = '$user_id' AND block = $block) THEN 1
ELSE last_position+1 END)";

           if($type == 1)
             $sql = "UPDATE saedd_awards SET coins = coins+1, medals =
floor(coins/8), trophies = floor(coins/16), tries = tries+1,
last_position = $position WHERE user_id = '$user_id'";
           else if($type == 0)
             $sql = "UPDATE saedd_awards SET coins = coins+16, medals =
floor(coins/8), trophies = floor(coins/16), tries = tries+16 WHERE
user_id = '$user_id'";
```

```
            else if($type == 2)
              $sql = "UPDATE saedd_awards SET tries = tries+1,
last_position = $position WHERE user_id = '$user_id'";
            else if($type == 3)
              $sql = "UPDATE saedd_awards SET coins = coins+8, medals =
floor(coins/8), trophies = floor(coins/16), tries = tries+8 WHERE user_id
= '$user_id'";
            else if($type == 4)
              $sql = "UPDATE saedd_awards SET last_day = CURDATE() -
INTERVAL 1 DAY WHERE user_id = '$user_id'";

            if(!$Obj->ExecQuery($sql))
               echo "erro";

            if($type == 4)
              if(!$Obj->ExecQuery("UPDATE saedd_protocol SET
registration_date =  (CURDATE() - INTERVAL 1 DAY) WHERE
DATE(registration_date) = CURDATE() AND  user_id = '$user_id'"))
                echo "erro";

            return 1;

         }


         if(isset($_POST['action'])) {

            $user_id = 1;

            $action = intval($_POST['action']);
            require_once('../MysqlDB.php');
            $Obj = new ScriptDB();
            $html = "";

            if($action == 1)
                 ChangeUserAwards(1,"",1);
            else if($action == 0)
                 ChangeUserAwards(0,"",1);
            else if($action == 2)
                 ChangeUserAwards(2,"",1);
            else if($action == 3)
                 ChangeUserAwards(3,"",1);
            else if($action == 4)
                 ChangeUserAwards(4,"",1);

            else if($action == 5) {

                 if(isset($_POST["difficult"]) && $_POST["difficult"] !=
"") {

                     $hard =
floatval(str_replace(",",".",$_POST["difficult"]));
                     $day = intval($_POST["day"]);
                     $block = intval($_POST["block"]);
                     $position = intval($_POST["position"]);

                     $sql = "UPDATE saedd_protocol SET difficult =
'$hard', registration_date = NOW(), keep_day = keep_day-1 WHERE user_id =
'$user_id' AND position = '$position' AND block = '$block';";

                     if($Obj->ExecQuery($sql)) {
                        if($hard <= 2.0)
                           ChangeUserAwards(1,$position,1);
                        else
                           ChangeUserAwards(2,$position,1);
```

```
                    }
                    else
                      echo "ERROR: ".$sql." ".$Obj->GetLastError();

                    if($hard >= 2.1 && $hard <= 7.0) {
                        $sql = "UPDATE saedd_protocol SET keep_day = 2
WHERE user_id = '$user_id' AND position = '$position' AND block =
'$block';";
                    }
                    else if($hard <= 2.0) {
                        $sql = "UPDATE saedd_protocol SET keep_day = 1
WHERE user_id = '$user_id' AND position = '$position' AND block =
$block+1;";
                    }
                    else if($hard >= 7.1 && $block != 1) {
                        $Obj->ExecQuery("UPDATE saedd_protocol SET
keep_day = 2 WHERE user_id = '$user_id' AND position = '$position' AND
block = $block-1;");
                        $sql = "UPDATE saedd_protocol SET keep_day = NULL
WHERE user_id = '$user_id' AND position = '$position' AND block =
'$block';";
                    }
                    else if($hard >= 7.1 && $block == 1) {
                        $sql = "UPDATE saedd_protocol SET keep_day = 2
WHERE user_id = '$user_id' AND position = '$position' AND block =
$block;";
                    }

                    if(!$Obj->ExecQuery($sql))
                         echo "ERROR 2: ".$sql." ".$Obj->GetLastError();

                }



                $sql = "SELECT a.id, title, a.block, a.position,
a.number, difficult, evolution, description, video FROM saedd_protocol a,
saedd_exercises WHERE code = number AND user_id = '$user_id' AND position
= (SELECT last_position FROM saedd_awards WHERE user_id = a.user_id) AND
keep_day IS NOT NULL AND keep_day > 0 ORDER BY block, position LIMIT 1";
                $Obj->ExecQuery($sql);

                $count = $Obj->Get("RecordCount");

                if($count != 0) {
                   $id = $Obj->GetQueryValue(0, 0);
                   $block = $Obj->GetQueryValue(0, 2);
                   $position = $Obj->GetQueryValue(0, 3);
                   $title = $Obj->GetQueryValue(0, 4) . " - " . $Obj-
>GetQueryValue(0, 1);
                   $hard = $Obj->GetQueryValue(0, 5);
                   $evo = $Obj->GetQueryValue(0, 6);

                   $html .= "|".$block."|".$position."|<h2>Bloco $block -
Grupo  $position</h2>";
                   $html .= "<h4><span>Exercício $title</span></h4>";
                   $html .= "<h4><span style='color:
#22CC33;'>$evo</span></h4><br/>";

                   if($hard != "")
                     $html .= "<b>Última tentativa (Dificuldade):</b>
$hard<br/>";

                    $html .= "<br/><b>".$Obj->GetQueryValue(0, 7)."</b>";
                    $html .= "<br/>";
```

```
                    $html .= "|".$Obj->GetQueryValue(0, 8);
                }
                else {
                    $html .= "|0|0|<br/><br/><br/><h4><span>Parabéns você
concluiu todos os exercícios!</span></h4><br/><h3><span>Retorne essa
semana para dar continuidade ao seu programa de exercícios</span></h3>|--
|--";
                }
            }



            $sql = "UPDATE saedd_awards SET days = (CASE WHEN last_day =
CURDATE() THEN days ELSE days+1 END), last_day = CURDATE() WHERE user_id
= '$user_id'";
             if(!$Obj->ExecQuery($sql))
              echo "Erro!";

            $Obj->ExecQuery("SELECT coins, medals,
CONCAT(trophies,'<br/>', (SELECT title FROM saedd_trophies WHERE trophies
>= min_trophies AND trophies <= max_trophies limit 1)), tries, days, CASE
WHEN (SELECT COUNT(*) FROM saedd_protocol WHERE DATE(registration_date) =
last_day AND user_id = saedd_awards.user_id) >= (SELECT MAX(position)
FROM saedd_protocol WHERE user_id = saedd_awards.user_id) THEN 1 ELSE 0
END FROM saedd_awards WHERE user_id = '$user_id'");
            $count = $Obj->Get("RecordCount");

            if($count == 1)
              echo utf8_encode($Obj->GetQueryValue(0, 0)."|".$Obj-
>GetQueryValue(0, 1)."|".$Obj->GetQueryValue(0, 2)."|".$Obj-
>GetQueryValue(0, 3)."|".$Obj->GetQueryValue(0, 4)."|".$Obj-
>GetQueryValue(0, 5).$html);
           else
              echo "Erro!";
           return;
         }
?>

<script type='text/javascript'>

                function SetGrade(num) {
                        jQuery("#difficult").val(num);
                        updateAwards(5);
                }

                jQuery(document).ready(function() {
                        Preload();
                        updateAwards(5);
                });

                function updateAwards(acc)
                {
                        jQuery("#saida").html(ShowInfo("Aguarde..."));

                        jQuery.ajax({
                                type: "POST",
                                url: "php/pages/protocolo_final.php",
                                data: {action : acc, difficult :
jQuery("#difficult").val(), day : jQuery("#vday").val(), position :
jQuery("#vposition").val(), block : jQuery("#vblock").val()}
                        })
                        .done(function(html) {

                            jQuery("#saida").html("");
```

```
                            var item = html.split("|");
                            jQuery("#coin").html(item[0]);
                            jQuery("#medal").html(item[1]);
                            jQuery("#trophy").html(item[2]);
                            jQuery("#try").html(item[3]);
                            jQuery("#day").html(item[4]);

                           jQuery("#difficult").val("");

                            if(parseInt(item[5]) == 1) {

        jQuery("#rulerarea").css('display','none');

        jQuery("#videoarea").html("<br/><br/><h4><span>Parabéns você
concluiu todos os exercícios!</span></h4><br/><h3><span>Retorne essa
semana para dar continuidade ao seu programa de exercícios</span></h3>");
                                return;
                            }

                            if(acc == 5) {
                               jQuery("#exercise").html("B"+item[6]+"-
G"+item[7]);

                               jQuery("#videoarea").html(item[8]);
                               jQuery("#vday").val(item[4]);
                               jQuery("#vblock").val(item[6]);
                               jQuery("#vposition").val(item[7]);

                                if(item[9] != "")
                                 loadVideo(item[9]);

                                window.scrollTo(0, 0);
                            }

                          if(acc == 4) window.location =
'index.php?page=protocolo_final';


                        });
                }
</script>


<script type='text/javascript' src='js/pixi.min.js'></script>
<script type='text/javascript' src='js/ruler_video.js'></script>



<div  class="content_row row vc_row wpb_row vc_row-fluid default-style
fullwidth" style="position:relative;overflow:hidden;">
        <div class="rt-parallax-background" data-rt-parallax-
direction="1" data-rt-parallax-effect="horizontal" style="background-
image: url(images/portfolio_header.jpg);background-repeat:
repeat;background-size: cover;background-position: right
top;width:100%;height:100%;top:0;"></div>
        <div class="content_row_wrapper  default" style="padding-
top:25px;padding-bottom:15px;">
                <div class="vc_col-sm-12 wpb_column vc_column_container"
>
                        <div class="wpb_wrapper">
                                <div class="rt_heading_wrapper style-4">
                                        <h1 class="rt_heading  style-4"
>Simulação de Protocolo</h1>
                                </div>
                        </div>
                </div>
```

```
        </div>
</div>



<style type="text/css">
        h4 span {color: #428BCA;}
</style>



<div  class="content_row row vc_row wpb_row vc_row-fluid alt-style-1
fullwidth border_grid fixed_heights" >
        <div class="content_row_wrapper  default" style="padding-
top:50px;padding-bottom:50px; min-height: 500px;">
                <div class="vc_col-sm-12 wpb_column vc_column_container"
>
                        <div class="wpb_wrapper">


                                <?php
                                        $user_id = 1;

                                        require_once('php/MysqlDB.php');
                                        $Obj = new ScriptDB();


                                        if(isset($_GET['start'])) {

                                                $Obj->ExecQuery("DELETE
FROM saedd_awards WHERE user_id = '$user_id'");
                                                $Obj->ExecQuery("DELETE
FROM saedd_protocol WHERE user_id = '$user_id'");
                                        }

                                        $Obj->ExecQuery("SELECT COUNT(*)
FROM saedd_awards WHERE user_id = '$user_id'");


                                        if(intval($Obj->GetQueryValue(0,
0)) == 0) {

                                                $sql = "INSERT INTO
saedd_awards(coins, medals, trophies, last_day, tries, user_id) VALUES(0,
0, 0, CURDATE(), 0, '$user_id')";

                                                if(!$Obj-
>ExecQuery($sql))
                                                  echo "<h2>Erro ao
inserir dados de recompensa.</h2>";
                                        }


                                        $Obj->ExecQuery("SELECT COUNT(*)
FROM saedd_protocol WHERE user_id = '$user_id'");


                                        if(intval($Obj->GetQueryValue(0,
0)) == 0) {

                                                $sql = "INSERT INTO
saedd_protocol(protocol, block, position, number, difficult, user_id,
registration_date) SELECT protocol, block, position, exercise, NULL,
'$user_id', NULL FROM saedd_blocks WHERE protocol = COALESCE((SELECT
protocol FROM saedd_users WHERE id = '$user_id'), NULL, 1)";
```

```
                                                if(!$Obj-
>ExecQuery($sql))
                                                  echo "<h2>Erro ao
inserir dados de execícios.</h2>";

                                                $sql = "UPDATE
saedd_protocol SET keep_day = '1' WHERE block = '1' AND user_id =
'$user_id' ";

                                                if(!$Obj-
>ExecQuery($sql))
                                                  echo "<h2>Erro ao
inserir dados de execícios.</h2>";
                                        }

                                        $Obj->CloseDB();
                                ?>


                                <table>
                                        <tr style="text-align: center;
font-size: 18px; font-weight:
bold;"><td>Fichas</td><td>Moedas</td><td>Medalhas</td><td>Troféus</td><td
>Dia</td><td>Exercício</td></tr>
                                        <tr style="text-align:
center;"><td><img src='images/try.png' /></td><td><img
src='images/coin.png' /></td><td><img src='images/medal.png'
/></td><td><img src='images/trophy.png' /></td><td><img
src='images/date.png' /></td><td><img src='images/check.png' /></td></tr>
                                        <tr style="text-align: center;
font-size: 18px; font-weight: bold;"><td>x <span
id='try'>0</span></td><td>x <span id='coin'>0</span></td><td>x <span
id='medal'>0</span></td><td>x <span id='trophy'>0</span></td><td>x <span
id='day'>0</span></td><td><span id='exercise'>---</span></td></tr>
                                </table>

                                <div id='saida'></div><br/><br/>

                                <input type="button"
onclick="javascript:updateAwards(1);" value="+1 moeda" />
                                <input type="button" style="margin-left:
40px;" onclick="javascript:updateAwards(3);" value="+1 medalha" />
                                <input type="button" style="margin-left:
40px;" onclick="javascript:updateAwards(0);" value="+1 trofeu" />
                                <input type="button" style="margin-left:
40px;" onclick="javascript:updateAwards(2);" value="+1 tentativa
(Exercício difícil)" />
                                <input type="button" style="margin-left:
40px;"  onclick="javascript:updateAwards(4);" value="Mudar dia" />

                                <br/><br/>

                                <div id='videoarea'></div><br/>
                                <div id="rulerarea"><canvas id='game'
width="720" height="800"></canvas></div>


                                <input type="hidden" id="vday" value=""
/>
                                <input type="hidden" id="vblock" value=""
/>
                                <input type="hidden" id="vposition"
value="" />
```

```
                                <input type="hidden" id="difficult"
value="" />
                                <input type="hidden" id="banned" value=""
/>
                        </div>
                </div>
        </div>
</div>
<div  class="content_row row vc_row wpb_row vc_row-fluid default-style
fullwidth" style="position:relative;overflow:hidden;">
        <div class="rt-parallax-background" data-rt-parallax-
direction="1" data-rt-parallax-effect="horizontal" style="background-
image: url(images/portfolio_header.jpg);background-repeat:
repeat;background-size: cover;background-position: right
top;width:100%;height:100%;top:0;"></div>
        <div class="content_row_wrapper  default" style="padding-
top:25px;padding-bottom:15px;">
                <div class="vc_col-sm-12 wpb_column vc_column_container"
>
                        <div class="wpb_wrapper">
                                <div class="rt_heading_wrapper style-4">
                                        <h1 class="rt_heading  style-4"
>Regras do Jogo</h1>
                                </div>
                        </div>
                </div>
        </div>
</div>



<script type="text/javascript">

        var pos = 2;

        function showArea() {

                for(var i=1; i<=6; i++)
                  jQuery("#area"+i).css("display","none");

                jQuery("#area"+pos ).css("display","inline");
                if(pos <= 6) {
                  pos++;
                return;
                }

                alert("Começa o exercício!");
        }
</script>


<div  class="content_row row vc_row wpb_row vc_row-fluid alt-style-1
fullwidth border_grid fixed_heights" >
        <div class="content_row_wrapper  default" style="padding-
top:50px;padding-bottom:50px; min-height: 500px;">
                <div class="vc_col-sm-12 wpb_column vc_column_container"
>
                        <div class="wpb_wrapper">


                                        <div id='area1' >
                                                 <h2>PARABÉNS!</h2>
```

```
                                                        Estamos felizes que você
tenha chegado até aqui!<br/><strong>Isso mostra que você tem interesse em
melhorar a saúde e a qualidade de vida dos seus pés.</strong><br/>
                                                        Antes de iniciar os
exercícios gostaríamos de explicar algumas regras que tornarão o processo
mais divertido. <br/><br/><br/>
                                        </div>

                                        <div id='area2' style="display:
none;">
                                           <h2>RESUMO DE UMA PARTIDA -OU
SESSÃO</h2>
                                            Cada <strong>partida</strong>
consiste na realização de <strong>8 exercícios para pés e
tornozelos</strong>.
                                           <br/>As partidas não
ultrapassam 20 minutos e podem ser executadas em qualquer ambiente de sua
escolha.<br/><br/>

                                            <h4>1 partida = 8 exercícios
(No máximo 20 minutos)</h4>

                                                        <img
src="images/exercicio/man.png" style="width: 70px;">
                                                        <img
src="images/exercicio/man.png" style="width: 70px;">
                                                        <img
src="images/exercicio/man.png" style="width: 70px;">
                                                        <img
src="images/exercicio/man.png" style="width: 70px;">
                                                        <img
src="images/exercicio/man.png" style="width: 70px;">
                                                        <img
src="images/exercicio/man.png" style="width: 70px;">
                                                        <img
src="images/exercicio/man.png" style="width: 70px;">
                                                        <img
src="images/exercicio/man.png" style="width: 70px;">
                                                        <img
src="images/exercicio/right.png" >
                                                        <img
src="images/date.png" >
                                                        <br/><br/><br/>
                                        </div>


                                <div id='area3' style="display: none;">
                                        <hr/><hr/>
                                        <h2>PREPARAÇÃO</h2>
                                        Para a realização dos exercícios
propostos tenha sempre por perto <strong>uma toalha de mão, uma bolinha
de tênis, uma bexiga, lápis, algodão</strong> e <span style='#color:
#428BCA;'>muita disposição</span>!<br/><br/>
                                                        <h4>Reserve antes de uma
partida</h4>
                                                        <img
src="images/exercicio/toalha.png" style="height: 80px;">
                                                        <img
src="images/exercicio/tenis.png" style="height: 80px;">
                                                        <img
src="images/exercicio/bexiga.png" style="height: 80px;">
                                                        <img
src="images/exercicio/lapis.png" style="height: 80px;">
                                                        <img
src="images/exercicio/algodao.png" style="height: 80px;">
```

```
                                                <img
src="images/exercicio/right.png" >
                                                <img
src="images/exercicio/sorriso.png" style="height: 80px;">
                                        <br/><br/><br/>
                                </div>

                                <div id='area4' style="display: none;">
                                        <hr/><hr/>
                                        <h2>OBJETIVO</h2>
                                        Este programa de exercícios tem
como objetivo promover a realização de exercícios com foco nos
<strong>pés e tornozelos</strong>, com intuito de garantir uma boa
funcionalidade dos músculos e articulações que são comprometidos nas
pessoas com diabetes, especialmente as pessoas com polineuropatia.
                                        <br/><br/><strong>Funciona como
um jogo</strong> onde a chave para dominar o sistema depende de você!
Vamos entender como este jogo funciona?<br/><br/><br/>
                                </div>

                                <div id='area5' style="display: none;">
                                        <hr/><hr/>
                                        <h2>Como funciona?</h2>
                                        Ao final de cada exercício
<strong>você mesmo</strong> avaliará a dificuldade de realizar o
exercício, através de <strong>uma escala de 0 à 10</strong>, sendo que 0
significa pouco esforço e 10 muito esforço.
                                        <br/><br/>
                                        <img src="images/ruler.png"
/><br/><br/><br/>
                                </div>

                                <div id='area6' style="display: none;">
                                        <hr/><hr/>
                                        <h2>Recompensas</h2>
                                        A <strong>cada exercício</strong>
realização de um você será <strong>premiado com uma ficha</strong>,
independente se você achou fácil ou difícil.<br/>
                                        A cada exercício executado
<strong>sem dificuldade</strong> (Valores de 0 a 2 na régua), você
ganhará <strong>1 moeda</strong>.<br/>
                                        Com o acúmulo de <strong>8
moedas</strong>, você ganhará <strong>1 medalha</strong> e quando estiver
com <strong>2 medalhas</strong>, você ganhará <strong>um troféu</strong>
que lhe mudará de aumentará seu nível de experiência.<br/><br/>
                                        <strong>É de extrema importância
ser honesto consigo mesmo nas avaliações.</strong>
                                        <br/><br/>
                                        Dê o seu melhor e seja um expert
e compartilhe o seu desempenho com os seus amigos. Pronto para
começar?<br/><br/><br/>
                                        <br/><br/>
                                        <h4>Cada exercício = 1 ficha</h4>
                                        <img
src="images/exercicio/man.png" style="height: 80px;">
                                        <img
src="images/exercicio/right.png" >
                                        <img src="images/try.png"
style="height: 80px;"><br/><br/>

                                        <h4>Cada exercício sem
dificuldade = 1 moeda</h4>
                                        <img
src="images/exercicio/sorriso.png" style="height: 80px;">
```

```
                                        <img
src="images/exercicio/right.png" >
                                        <img src="images/coin.png"
style="height: 60px;"><br/><br/>

                                        <h4>Cada 8 moedas = 1
medalha</h4>
                                        <img src="images/coin.png"
style="height: 60px;">
                                        <img src="images/coin.png"
style="height: 60px;">
                                        <img src="images/coin.png"
style="height: 60px;">
                                        <img src="images/coin.png"
style="height: 60px;">
                                        <img src="images/coin.png"
style="height: 60px;">
                                        <img src="images/coin.png"
style="height: 60px;">
                                        <img src="images/coin.png"
style="height: 60px;">
                                        <img src="images/coin.png"
style="height: 60px;">
                                        <img
src="images/exercicio/right.png" >
                                        <img src="images/medal.png"
style="height: 60px;">
                                        <br/><br/>

                                        <h4>Cada 2 medalhas = 1 trofeu
(sobe de nível)</h4>
                                        <img src="images/medal.png"
style="height: 60px;">
                                        <img src="images/medal.png"
style="height: 60px;">
                                        <img
src="images/exercicio/right.png" >
                                        <img src="images/trophy.png"
style="height: 60px;">
                                        <img
src="images/exercicio/right.png" >
                                        <img
src="images/exercicio/levelup.png" style="height: 80px;">

                                        <br/><br/><br/>
                                </div>



                                <input type="button"
onclick="javascript:showArea();" value="Ok! Entendi e quero continuar..."
/>
                        </div>
                </div>
        </div>
</div>
<div  class="content_row row vc_row wpb_row vc_row-fluid default-style
fullwidth" style="position:relative;overflow:hidden;">
        <div class="rt-parallax-background" data-rt-parallax-
direction="1" data-rt-parallax-effect="horizontal" style="background-
image: url(images/portfolio_header.jpg);background-repeat:
repeat;background-size: cover;background-position: right
top;width:100%;height:100%;top:0;"></div>
```

```
        <div class="content_row_wrapper  default" style="padding-top:25px;padding-bottom:15px;">
                <div class="vc_col-sm-12 wpb_column vc_column_container" >
                        <div class="wpb_wrapper">
                                <div class="rt_heading_wrapper style-4">
                                        <h1 class="rt_heading  style-4" >Inserção de áudios</h1>
                                </div>
                        </div>
                </div>
        </div>
</div>

<div  class="content_row row vc_row wpb_row vc_row-fluid alt-style-1 fullwidth border_grid fixed_heights" >
        <div class="content_row_wrapper  default" style="padding-top:50px;padding-bottom:50px; min-height: 500px;">
                <div class="vc_col-sm-12 wpb_column vc_column_container" >
                        <div class="wpb_wrapper">
                                <div class="rt_heading_wrapper style-6">
                                        <h4 class="rt_heading  style-6" ><span class="icon-glyph-15 heading_icon"></span>Audio dos exercícios</h4>
                                </div>
                                <div class="wpb_text_column wpb_content_element ">
                                        <div class="wpb_wrapper">
                                                <p>Envio de áudio com descrição dos exercícios.</b></p>
                                        </div>
                                </div>
                                <link rel='stylesheet' href='css/mytable.css' type='text/css' media='all' />
        <script type='text/javascript' src='js/jquery.js'></script>
        <script src="js/plupload/plupload.full.min.js"></script>

<link rel='stylesheet' href='css/myform.css' type='text/css' media='all' />

<style type="text/css">
.alt-style-1 input[type="button"]:hover {background-color: #FC9D0D;}
.alt-style-1 input[type="button"] {background-color: #428bca; font-weight: bold;}

.alt-style-1 input[type="button"]:hover.extra  {background-color: #BBB;}
.alt-style-1 input[type="button"].extra  {background-color: #888; font-weight: bold;}

#myform input[type="text"]:disabled {
    background: #aaa;
}
#myform span{color: #ff0000; font-size: 16px;}
</style>

<?php

                function AddFiles($buttonText, $allowedExt, $codigo, $id, $rename = true)
                {
                        if($allowedExt != "")
                          $allowedExt = "mime_types : [{ title : 'Arquivos $allowedExt', extensions : '$allowedExt' }], ";
```

```
                        $html = "<input type='hidden' value='0'
id='submit_$id'/><input type='hidden' value='' id='path_$id'/><input
type='button' class='mybt' onclick='javascript:void(0);' id='$id'
value='$buttonText' /> <br/><br/><div id='msg_$id'></div>";
                        $html .= "<script type='text/javascript'>

                                        var MyFileName_$id = '';

                                        var uploader_$id = new
plupload.Uploader({
                                                browse_button: '$id',
                                                url :
'js/plupload/upload.php?path=',
                                                filters: {
                                                        $allowedExt
                                                        max_file_size:
'50mb'
                                                }
                                        });

                                        uploader_".$id.".init();


        uploader_".$id.".bind('FilesAdded', function(up, files) {

                                                var html = '';

                                                plupload.each(files,
function(file) {
                                                        var fileName =
file.name;


        uploader_".$id.".settings.url =
'js/plupload/upload.php?path=images/$id/';
                                                        MyFileName_$id =
'images/$id/'+fileName;

        document.getElementById('msg_$id').innerHTML +=
ShowInfo('Enviando <b>' +file.name+'</b> (<b id=\"'+file.id+'\"></b> de
'+plupload.formatSize(file.size)+')');
                                                });



        document.getElementById('msg_$id').innerHTML += html;
                                                uploader_".$id.".start();
                                        });


        uploader_".$id.".bind('UploadProgress', function(up, file) {

        document.getElementById(file.id).innerHTML = file.percent + '%';
                                        });

                                        uploader_".$id.".bind('Error',
function(up, err) {
                                                if(err.code == '-601')

        document.getElementById('msg_$id').innerHTML = ShowError('Arquivo
enviado não é um formato válido: '+err.file.name);
                                                else
```

```
        document.getElementById('msg_$id').innerHTML = ShowError(err.code
+ ': ' + err.message);

        document.getElementById('submit_$id').value = '0';
                                        });


        uploader_".$id.".bind('FileUploaded', function(up, file, info){

        document.getElementById('msg_$id').innerHTML =
ShowSuccess(file.name+' enviado com sucesso!')+'<br/><a
href=\"'+MyFileName_$id+'\" target=\"_blank\">Clique aqui</a> para baixar
o arquivo';

        document.getElementById('submit_$id').value = '1';

        document.getElementById('path_$id').value = MyFileName_$id;
                                        });
                                </script>";

                        $html .= "<br/><br/>";

                        return $html;
                }

?>

                                <div class="vc_col-sm-12 wpb_column
vc_column_container" >
                                                        <label>Selecione
o exercício <span>*</span></label><br/>
                                                        <select size="1"
name="exercicio">



<?php
                        require_once('php/MysqlDB.php');
                        $id = $_SESSION['id'];

                        $Obj = new ScriptDB();
                        $Obj->Connect();
                        $Obj->ExecQuery("SELECT CONCAT(code,' - ',title)
from saedd_exercises");

                        echo $Obj->QueryAsHtmlOptions();



?>

                                                        </select>
                                                </div>
                                        <div class='clear
clearfix'></div><br/><br/>

                                        <div class="submit-button"
style='text-align: left;'>
                                                <?php echo
AddFiles("Enviar imagem", "mp3", 1, "audios", false); ?>
                                        </div>
```

```
                                </div>
                        </div>
                </div>
</div>





<div  class="content_row row vc_row wpb_row vc_row-fluid default-style
fullwidth" style="position:relative;overflow:hidden;">
        <div class="rt-parallax-background" data-rt-parallax-
direction="1" data-rt-parallax-effect="horizontal" style="background-
image: url(images/portfolio_header.jpg);background-repeat:
repeat;background-size: cover;background-position: right
top;width:100%;height:100%;top:0;"></div>
        <div class="content_row_wrapper  default" style="padding-
top:25px;padding-bottom:15px;">
                <div class="vc_col-sm-12 wpb_column vc_column_container"
>
                        <div class="wpb_wrapper">
                                <div class="rt_heading_wrapper style-4">
                                        <h1 class="rt_heading  style-4"
>Compartilhamento de Status - SoPed</h1>
                                </div>
                        </div>
                </div>
        </div>
</div>





        <style>
                .icircle img {-webkit-border-radius: 124px;
                        -moz-border-radius: 124px;
                        border-radius: 124px;
                        width: 120px;}
                .wpb_wrapper input[type="button"] {background: #428BCA;}
                .wpb_wrapper input[type="button"]:hover {background:
#317AB9;}
        </style>

<div  class="content_row row vc_row wpb_row vc_row-fluid alt-style-1
fullwidth border_grid fixed_heights" >
        <div class="content_row_wrapper  default" style="padding-
top:50px;padding-bottom:50px; min-height: 500px;">
                <div class="vc_col-sm-12 wpb_column vc_column_container"
>
                        <div class="wpb_wrapper">

                                                <div class="icircle">


<h1>Fulano de Tal</h1><br/>
<img src="images/t1.png" style="float: left; margin: 20px; width: 150px;
border: 3px solid #FFF;" />

                                                        <img
src="images/coin.png" style="height: 60px; width: 60px;"> x 202
                                                        <img
src="images/medal.png" style="height: 60px; width: 60px;"> x 65
```

```
                                                        <img
src="images/trophy.png" style="height: 60px; width: 60px;"> x 9<br/><br/>
                                                        <h3>Agora sou um
virtuoso!</h3><br/><br/>


                                        <div
class="addthis_inline_share_toolbox" ></div>

                                        <script type="text/javascript"
src="//s7.addthis.com/js/300/addthis_widget.js#pubid=ra-
58fa5601eaa0c0e1"></script>



    </script>

                        </div>
                </div>
        </div>
</div>
<div  class="content_row row vc_row wpb_row vc_row-fluid default-style
fullwidth" style="position:relative;overflow:hidden;">
        <div class="rt-parallax-background" data-rt-parallax-
direction="1" data-rt-parallax-effect="horizontal" style="background-
image: url(images/portfolio_header.jpg);background-repeat:
repeat;background-size: cover;background-position: right
top;width:100%;height:100%;top:0;"></div>
        <div class="content_row_wrapper  default" style="padding-
top:25px;padding-bottom:15px;">
                <div class="vc_col-sm-12 wpb_column vc_column_container"
>
                        <div class="wpb_wrapper">
                                <div class="rt_heading_wrapper style-4">
                                        <h1 class="rt_heading  style-4"
>Cuidados com os pés</h1>
                                </div>
                        </div>
                </div>
        </div>
</div>

<div  class="content_row row vc_row wpb_row vc_row-fluid default-style
fullwidth border_grid fixed_heights" >
        <div class="content_row_wrapper  default" style="padding-
top:50px;padding-bottom:50px; min-height: 500px;">
                <div class="vc_col-sm-12 wpb_column vc_column_container"
>
                        <div class="wpb_wrapper">
                                <div class="rt_heading_wrapper style-6">
                                        <h4 class="rt_heading  style-6"
><span class="icon-glyph-15 heading_icon"></span>Alguns cuidados para seu
pé</h4>
                                </div>
                                <div class="wpb_text_column
wpb_content_element ">
                                        <div class="wpb_wrapper">
                                                <p>Gostaria de saber um
pouco mais sobre cuidados com os pés? <b>Clique nas abas para detalhar as
informações.</b></p>
                                        </div>
                                </div>
                                <div id="Unique-ID" class="rt-toggle CSS-
Class-Name">
                                        <ol>
```

```
                                                    <li  class="">
                                                            <div
class="toggle-head">
                                                                    <div
class="toggle-number">1</div>
                                                                    <div
class="toggle-title">Cuidados com os pés e as unhas</div>
                                                            </div>
                                                            <div
class="toggle-content">
                                                                    <div
class="wpb_text_column wpb_content_element ">

        <div class="wpb_wrapper">
                                                                                    <p>Verif
também evite essa região entre os dedos; use calçados confortáveis e
meias, ambos limpos; evite ficar descalço, principalmente em locais
quentes para evitar o risco de queimaduras e mesmo uma simples batida no
dedão pode acarretar algo mais grave; No aparecimento de um  ferimento,
trate-o imediatamente e procure um profissional especializado; cuidado ao
cortar as unhas e evite tirar as cutículas. O recomendado é que procure
um profissional habilitado, como podólogo; o ideal é não cortar os calos,
é recomendado que converse com um especialista no aparecimento deste.</p>

        </div>
                                                                    </div>
                                                            </div>
                                                    </li>
                                                    <li  class="">
                                                            <div
class="toggle-head">
                                                                    <div
class="toggle-number">2</div>
                                                                    <div
class="toggle-title">Orientações</div>
                                                            </div>
                                                            <div
class="toggle-content">
                                                                    <div
class="wpb_text_column wpb_content_element ">

        <div class="wpb_wrapper">
                                                                                    <p>É imp
mínimas, todo o cuidado é preciso, pois uma pequena úlcera, quando não
tratada, poderá infeccionar e causar um problema muito mais sério, como a
amputação. É muito importante o cuidado preventivo com os pés.</p>

        </div>
                                                                    </div>
                                                            </div>
                                                    </li>


                                            </ol>
                                    </div>
                            </div>
                    </div>
        </div>
</div>
<div  class="content_row row vc_row wpb_row vc_row-fluid alt-style-1
fullwidth border_grid fixed_heights" >
        <div class="content_row_wrapper  default" style="padding-
top:40px;padding-bottom:40px;">
                <div class="vc_col-sm-4 wpb_column vc_column_container
vc_custom_1421091415717" >
```

```
                        <div class="wpb_wrapper">
                                <article  class="icon-content-box
content-box icon-top icon-style-3">
                                        <div class="icon-holder"><a
href="#" title="" target="_self"><span class="icon-new-link-
1"></span></a></div>
                                        <div class="text-holder">
                                                <h4 class="heading">
                                                        <a href="#"
title="Box Heading" target="_self">
                                                        Por que utilizar?
                                                        </a>
                                                </h4>
                                                <p
class="aligncenter">Além de ser fácil e gratuito a ferramenta se ajustar
aos horários do paciente e promove uma percepível melhora do bem estar
dos pés.</p>
                                        </div>
                                </article>
                        </div>
                </div>
                <div class="vc_col-sm-4 wpb_column vc_column_container" >
                        <div class="wpb_wrapper">
                                <article  class="icon-content-box
content-box icon-top icon-style-3">
                                        <div class="icon-holder"><a
href="#" title="" target="_self"><span class="icon-new-user-
1"></span></a></div>
                                        <div class="text-holder">
                                                <h4 class="heading">
                                                        <a href="#"
title="Box Heading" target="_self">
                                                        Fale com o
Doutor!
                                                        </a>
                                                </h4>
                                                <p class="aligncenter">Em
caso de dúvidas consulte nossos especialistas online. Estamos sempre
pronto para atendê-lo!</p>
                                        </div>
                                </article>
                        </div>
                </div>
                <div class="vc_col-sm-4 wpb_column vc_column_container" >
                        <div class="wpb_wrapper">
                                <article  class="icon-content-box
content-box icon-top icon-style-3">
                                        <div class="icon-holder"><a
href="#" title="" target="_self"><span class="icon-new-heart-
1"></span></a></div>
                                        <div class="text-holder">
                                                <h4 class="heading">
                                                        <a href="#"
title="Box Heading" target="_self">
                                                        Gostou?
Compartilhe!
                                                        </a>
                                                </h4>
                                                <p
class="aligncenter">Indique o SoPeD para amigos e pessoas do sua rede de
contato. Você estará ajudando a melhorar a ferramenta.</p>
                                        </div>
                                </article>
                        </div>
                </div>
```

```
        </div>
</div>
<?php

        function ChangeUserAwards($type, $position, $block) {

           $user_id = 1;

           require_once('../MysqlDB.php');
           $Obj = new ScriptDB();
           $sql = "";

           if($position == "")
             $position = "last_position";
           else
             $position = "(CASE WHEN $position = (SELECT MAX(position)
FROM saedd_protocol WHERE user_id = '$user_id') THEN 1 ELSE
last_position+1 END)";

           if($type == 1)
             $sql = "UPDATE saedd_awards SET coins = coins+1, medals =
floor(coins/8), trophies = floor(coins/80), tries = tries+1,
last_position = $position WHERE user_id = '$user_id'";
           else if($type == 2)
             $sql = "UPDATE saedd_awards SET tries = tries+1,
last_position = $position WHERE user_id = '$user_id'";
           else if($type == 3)
             $sql = "UPDATE saedd_awards SET coins = coins+8, medals =
floor(coins/8), trophies = floor(coins/80), tries = tries+1 WHERE user_id
= '$user_id'";
           else if($type == 4)
             $sql = "UPDATE saedd_awards SET last_day = CURDATE() -
INTERVAL 1 DAY WHERE user_id = '$user_id'";

           if(!$Obj->ExecQuery($sql))
              echo "erro";

           if($type == 4)
             if(!$Obj->ExecQuery("UPDATE saedd_protocol SET
registration_date =  (CURDATE() - INTERVAL 1 DAY) WHERE
DATE(registration_date) = CURDATE() AND  user_id = '$user_id'"))
               echo "erro";

           return 1;

        }


        if(isset($_POST['action'])) {

           $user_id = 1;

           $action = intval($_POST['action']);
           require_once('../MysqlDB.php');
           $Obj = new ScriptDB();
           $html = "";

           if($action == 1)
                ChangeUserAwards(1,"",1);
           else if($action == 2)
                ChangeUserAwards(2,"",1);
           else if($action == 3)
                ChangeUserAwards(3,"",1);
           else if($action == 4)
                ChangeUserAwards(4,"",1);
```

```
          else if($action == 5) {

               if(isset($_POST["difficult"]) && $_POST["difficult"] !=
"") {

                    $hard =
floatval(str_replace(",",".",$_POST["difficult"]));
                    $day = intval($_POST["day"]);
                    $block = intval($_POST["block"]);
                    $position = intval($_POST["position"]);

                    $sql = "UPDATE saedd_protocol SET difficult =
'$hard', registration_date = NOW(), keep_day = keep_day-1 WHERE user_id =
'$user_id' AND position = '$position' AND block = '$block';";

                    if($Obj->ExecQuery($sql)) {
                       if($hard <= 2.0)
                          ChangeUserAwards(1,$position,1);
                       else
                          ChangeUserAwards(2,$position,1);
                    }
                    else
                      echo "ERROR: ".$sql." ".$Obj->GetLastError();

                    if($hard >= 2.1 && $hard <= 7.0) {
                        $sql = "UPDATE saedd_protocol SET keep_day = 2
WHERE user_id = '$user_id' AND position = '$position' AND block =
'$block';";
                    }
                    else if($hard <= 2.0) {
                        $sql = "UPDATE saedd_protocol SET keep_day = 1
WHERE user_id = '$user_id' AND position = '$position' AND block =
$block+1;";
                    }
                    else if($hard >= 7.1 && $block != 1) {
                        $sql = "UPDATE saedd_protocol SET keep_day = 1
WHERE user_id = '$user_id' AND position = '$position' AND block = $block-
1; UPDATE saedd_protocol SET keep_day = NULL, registration_date = NULL
WHERE user_id = '$user_id' AND position = '$position' AND block =
'$block';";
                    }
                    else if($hard >= 7.1 && $block == 1) {
                        $sql = "UPDATE saedd_protocol SET keep_day = 2
WHERE user_id = '$user_id' AND position = '$position' AND block =
$block;";
                    }

                    if(!$Obj->ExecQuery($sql))
                         echo "ERROR 2: ".$sql." ".$Obj->GetLastError();

               }



               $sql = "SELECT a.id, title, a.block, a.position,
a.number, difficult, evolution, description, video FROM saedd_protocol a,
saedd_exercises WHERE code = number AND user_id = '$user_id' AND position
= (SELECT last_position FROM saedd_awards WHERE user_id = a.user_id) AND
keep_day IS NOT NULL AND keep_day > 0 ORDER BY block, position LIMIT 1";
               $Obj->ExecQuery($sql);

               $count = $Obj->Get("RecordCount");

               if($count != 0) {
```

```
                   $id = $Obj->GetQueryValue(0, 0);
                   $block = $Obj->GetQueryValue(0, 2);
                   $position = $Obj->GetQueryValue(0, 3);
                   $title = $Obj->GetQueryValue(0, 4) . " - " . $Obj-
>GetQueryValue(0, 1);
                   $hard = $Obj->GetQueryValue(0, 5);
                   $evo = $Obj->GetQueryValue(0, 6);

                   $html .= "|".$block."|".$position."|<h2>Bloco $block -
Grupo  $position</h2>";
                   $html .= "<h4><span>Exercício $title</span></h4>";
                   $html .= "<h4><span style='color:
#22CC33;'>$evo</span></h4><br/>";

                   if($hard != "")
                     $html .= "<b>Última tentativa (Dificuldade):</b>
$hard ";

                    $html .= "<br/>";
                    $html .= "|".$Obj->GetQueryValue(0, 7)."|".$Obj-
>GetQueryValue(0, 8);
                }
                else {
                    $html .= "|0|0|<br/><br/><h4><span>Parabéns você
concluiu todos os exercícios!</span></h4>|--|--";
                }
           }



           $sql = "UPDATE saedd_awards SET days = (CASE WHEN last_day =
CURDATE() THEN days ELSE days+1 END), last_day = CURDATE() WHERE user_id
= '$user_id'";
            if(!$Obj->ExecQuery($sql))
              echo "Erro!";

           $Obj->ExecQuery("SELECT coins, medals, trophies, tries, days,
CASE WHEN (SELECT COUNT(*) FROM saedd_protocol WHERE
DATE(registration_date) = last_day AND user_id = saedd_awards.user_id) >=
(SELECT MAX(position) FROM saedd_protocol WHERE user_id =
saedd_awards.user_id) THEN 1 ELSE 0 END FROM saedd_awards WHERE user_id =
'$user_id'");
           $count = $Obj->Get("RecordCount");

           if($count == 1)
             echo utf8_encode($Obj->GetQueryValue(0, 0)."|".$Obj-
>GetQueryValue(0, 1)."|".$Obj->GetQueryValue(0, 2)."|".$Obj-
>GetQueryValue(0, 3)."|".$Obj->GetQueryValue(0, 4)."|".$Obj-
>GetQueryValue(0, 5).$html);
          else
             echo "Erro!";
          return;
        }
?>

<script type='text/javascript'>

                function SetGrade(num) {
                        jQuery("#difficult").val(num);
                        updateAwards(5);
                }

                jQuery(document).ready(function() {
                        updateAwards(5);
                        Preload();
```

```
                });

                function updateAwards(acc)
                {
                        jQuery("#saida").html(ShowInfo("Aguarde..."));

                        jQuery.ajax({
                                type: "POST",
                                url: "php/pages/recompensas.php",
                                data: {action : acc, difficult :
jQuery("#difficult").val(), day : jQuery("#vday").val(), position :
jQuery("#vposition").val(), block : jQuery("#vblock").val()}
                        })
                        .done(function(html) {

                            jQuery("#saida").html("");
                            var item = html.split("|");
                            jQuery("#coin").html(item[0]);
                            jQuery("#medal").html(item[1]);
                            jQuery("#trophy").html(item[2]);
                            jQuery("#try").html(item[3]);
                            jQuery("#day").html(item[4]);

                           jQuery("#difficult").val("");

                            if(parseInt(item[5]) == 1) {

        jQuery("#rulerarea").css('display','none');
                                jQuery("#videoarea").html("<h3>Você
concluiu todos os exercícios por hoje.");
                                return;
                            }

                            if(acc == 5) {
                               jQuery("#exercise").html("B"+item[6]+"-
G"+item[7]);

                               jQuery("#videoarea").html(item[8]);
                               jQuery("#vday").val(item[4]);
                               jQuery("#vblock").val(item[6]);
                               jQuery("#vposition").val(item[7]);

                               loadVideo(item[10],item[9]);
                            }

                          if(acc == 4) window.location =
'index.php?page=recompensas';


                        });
                }
</script>


<script type='text/javascript' src='js/pixi.min.js'></script>
<script type='text/javascript' src='js/ruler_video.js'></script>



<div  class="content_row row vc_row wpb_row vc_row-fluid default-style
fullwidth" style="position:relative;overflow:hidden;">
        <div class="rt-parallax-background" data-rt-parallax-
direction="1" data-rt-parallax-effect="horizontal" style="background-
image: url(images/portfolio_header.jpg);background-repeat:
repeat;background-size: cover;background-position: right
top;width:100%;height:100%;top:0;"></div>
```

```
        <div class="content_row_wrapper  default" style="padding-
top:25px;padding-bottom:15px;">
                <div class="vc_col-sm-12 wpb_column vc_column_container"
>
                        <div class="wpb_wrapper">
                                <div class="rt_heading_wrapper style-4">
                                        <h1 class="rt_heading  style-4"
>Simulação de Recompensas</h1>
                                </div>
                        </div>
                </div>
        </div>
</div>



<style type="text/css">
        h4 span {color: #428BCA;}
</style>



<div  class="content_row row vc_row wpb_row vc_row-fluid alt-style-1
fullwidth border_grid fixed_heights" >
        <div class="content_row_wrapper  default" style="padding-
top:50px;padding-bottom:50px; min-height: 500px;">
                <div class="vc_col-sm-12 wpb_column vc_column_container"
>
                        <div class="wpb_wrapper">


                                <?php
                                        $user_id = 1;

                                        require_once('php/MysqlDB.php');
                                        $Obj = new ScriptDB();


                                        if(isset($_GET['start'])) {

                                                $Obj->ExecQuery("DELETE
FROM saedd_awards WHERE user_id = '$user_id'");
                                                $Obj->ExecQuery("DELETE
FROM saedd_protocol WHERE user_id = '$user_id'");
                                        }

                                        $Obj->ExecQuery("SELECT COUNT(*)
FROM saedd_awards WHERE user_id = '$user_id'");


                                        if(intval($Obj->GetQueryValue(0,
0)) == 0) {

                                                $sql = "INSERT INTO
saedd_awards(coins, medals, trophies, last_day, tries, user_id) VALUES(0,
0, 0, CURDATE(), 0, '$user_id')";

                                                if(!$Obj-
>ExecQuery($sql))
                                                  echo "<h2>Erro ao
inserir dados de recompensa.</h2>";
                                        }
```

```
                                        $Obj->ExecQuery("SELECT COUNT(*)
FROM saedd_protocol WHERE user_id = '$user_id'");


                                        if(intval($Obj->GetQueryValue(0,
0)) == 0) {

                                                $sql = "INSERT INTO
saedd_protocol(protocol, block, position, number, difficult, user_id,
registration_date) SELECT protocol, block, position, exercise, NULL,
'$user_id', NULL FROM saedd_blocks WHERE protocol = COALESCE((SELECT
protocol FROM saedd_users WHERE id = '$user_id'), NULL, 1)";

                                                if(!$Obj-
>ExecQuery($sql))
                                                  echo "<h2>Erro ao
inserir dados de execícios.</h2>";

                                                $sql = "UPDATE
saedd_protocol SET keep_day = '1' WHERE block = '1' AND user_id =
'$user_id' ";

                                                if(!$Obj-
>ExecQuery($sql))
                                                  echo "<h2>Erro ao
inserir dados de execícios.</h2>";
                                        }

                                        $Obj->CloseDB();
                                ?>


                                <table>
                                        <tr style="text-align: center;
font-size: 18px; font-weight:
bold;"><td>Moedas</td><td>Medalhas</td><td>Troféus</td><td>Tentativas</td
><td>Dia</td><td>Exercício</td></tr>
                                        <tr style="text-align:
center;"><td><img src='images/coin.png' /></td><td><img
src='images/medal.png' /></td><td><img src='images/trophy.png'
/></td><td><img src='images/try.png' /></td><td><img
src='images/date.png' /></td><td><img src='images/check.png' /></td></tr>
                                        <tr style="text-align: center;
font-size: 18px; font-weight: bold;"><td>x <span
id='coin'>0</span></td><td>x <span id='medal'>0</span></td><td>x <span
id='trophy'>0</span></td><td>x <span id='try'>0</span></td><td>x <span
id='day'>0</span></td><td><span id='exercise'>---</span></td></tr>
                                </table>

                                <div id='saida'></div><br/><br/>

                                <input type="button"
onclick="javascript:updateAwards(1);" value="+1 moeda (Exercício OK)" />
                                <input type="button" style="margin-left:
80px;" onclick="javascript:updateAwards(2);" value="+1 tentativa
(Exercício difícil)" />
                                <input type="button" style="margin-left:
80px;" onclick="javascript:updateAwards(3);" value="+1 medalha" />
                                <input type="button" style="margin-left:
80px;"  onclick="javascript:updateAwards(4);" value="Mudar dia" />

                                <br/><br/>

                                <div id='videoarea'></div><br/>
```

```
                                <div id='rulerarea'><canvas id='game'
width='360' height='640'></canvas></div>

                                <input type="hidden" id="vday" value=""
/>
                                <input type="hidden" id="vblock" value=""
/>
                                <input type="hidden" id="vposition"
value="" />
                                <input type="hidden" id="difficult"
value="" />
                                <input type="hidden" id="banned" value=""
/>
                        </div>
                </div>
        </div>
</div>
<?php

if(isset($_POST["action"])) {

        $action = $_POST["action"];
        require_once('../MysqlDB.php');

        if($action == "add") {

                $Obj = new ScriptDB();

                $Obj->Set("TableName", "saedd_diabetes");
                $Obj->Set("FieldsName",
"px|py|path|title|content|registration_date|id");
                $Obj->BeforeInsert("registration_date","NOW()");

                $sql = utf8_decode($Obj->CreateInsert());

                $Obj->Connect();
                if($Obj->ExecQuery($sql)) {
                        echo "ok";
                }
                else
                  echo $Obj->GetLastError();

                $Obj->CloseDB();
        }
        else if($action == "update") {

                $Obj = new ScriptDB();

                $Obj->Set("TableName", "saedd_diabetes");
                $Obj->Set("FieldsName", "px|py|path|title|content");

                $Obj->Set('PrimaryKey', 'id');
                $sql = utf8_decode($Obj->CreateUpdate());
                echo $sql;
                $Obj->Connect();
                if($Obj->ExecQuery($sql)) {
                        echo "ok";
                }
                else
                  echo $Obj->GetLastError();

                $Obj->CloseDB();
        }
        else if($action == "delete") {
```

```
                $codigo = str_replace("'","''",$_POST["id"]);

                $Obj = new ScriptDB();
                $sql = "DELETE FROM saedd_diabetes WHERE id = '$codigo'";

                $Obj->Connect();
                if($Obj->ExecQuery($sql)) {
                        echo "ok";
                }
                else
                  echo $Obj->GetLastError();

                $Obj->CloseDB();
        }
        else if($action == "select") {

                $Obj = new ScriptDB();

                $Obj->Connect();
                $Obj->ExecQuery("SELECT id, title, content, path FROM
saedd_diabetes ORDER BY id");

                $count = $Obj->Get('RecordCount');

                $html = "<div id='Unique-ID' class='rt-toggle CSS-Class-
Name'><ol>";
                echo "$count registros<br/><br/>";
                for($i=0; $i<$count; $i++) {
                        $id = $Obj->GetQueryValue($i, 0);
                        $title= $Obj->GetQueryValue($i, 1);
                        $content = $Obj->GetQueryValue($i, 2);
                        $path = $Obj->GetQueryValue($i, 3);
                        $html .= "
                                        <li  class=''>
                                                <div
class='toggle-head'>
                                                        <div
class='toggle-number'>$id</div>
                                                        <div
class='toggle-title'>$title</div>
                                                </div>
                                                <div
class='toggle-content'>
                                                        <div
class='wpb_text_column wpb_content_element'>

        <div class='wpb_wrapper'>
                                                                        <p>




modificações necessárias e clique em Atualizar registro.'); \"
value='Atualizar registro' />  <input type='button' class='mybt'
style='margin-left: 300px;'
onclick=\"javascript:jQuery('#codigo').val('$id'); deleteRecord();\"
value='Deletar registro' /><br/><br/><br/>
                                                                        </p>

        </div>
                                                        </div>
                                                </div>
                                        </li>
```

```
                                ";
                }
                $html .= "</ol></div>";
                $Obj->CloseDB();
                echo utf8_encode($html);
        }
        return;
}

?>


<div  class="content_row row vc_row wpb_row vc_row-fluid default-style
fullwidth" style="position:relative;overflow:hidden;">
        <div class="rt-parallax-background" data-rt-parallax-
direction="1" data-rt-parallax-effect="horizontal" style="background-
image: url(images/portfolio_header.jpg);background-repeat:
repeat;background-size: cover;background-position: right
top;width:100%;height:100%;top:0;"></div>
        <div class="content_row_wrapper  default" style="padding-
top:25px;padding-bottom:15px;">
                <div class="vc_col-sm-12 wpb_column vc_column_container"
>
                        <div class="wpb_wrapper">
                                <div class="rt_heading_wrapper style-4">
                                        <h1 class="rt_heading  style-4"
>Administração - Cadastro de Complicações</h1>
                                </div>
                        </div>
                </div>
        </div>
</div>

<link rel='stylesheet' href='css/myform.css' type='text/css' media='all'
/>

<style type="text/css">

.mybt {background-color: #428bca; font-weight: bold; color: #FFF;}
.mybt:hover {background-color: #FC9D0D; color: #FFF;}

</style>


<div  class="content_row row vc_row wpb_row vc_row-fluid fullwidth
border_grid fixed_heights" style='background: #FFF;'>
        <div class="content_row_wrapper  default" style="padding-
top:50px;padding-bottom:50px; min-height: 500px;">
                <div class="vc_col-sm-12 wpb_column vc_column_container"
>
                        <div class="wpb_wrapper">
                                <div class="rt_heading_wrapper style-6">
                                        <h4 class="rt_heading  style-6"
><span class="icon-glyph-15 heading_icon"></span>Cadastro de
Complicações</h4>
                                </div>
                                <div class="wpb_text_column
wpb_content_element ">
                                        <div class="wpb_wrapper">
                                                <p>Clique no local onde
ocorre a complicação e preencha os dados.</b></p>
                                        </div>
                                </div>
```

```
        <script type='text/javascript' src='js/pixi.min.js'></script>
        <script type='text/javascript'
src='js/admin_complicacoes.js'></script>
        <script type='text/javascript' src='js/jquery.js'></script>

        <script src="js/tinymce/tinymce.min.js"></script>
        <script src="js/plupload/plupload.full.min.js"></script>

        <script type="text/javascript">

                function getWordCount(id) {
                    var body = tinymce.get(id).getBody(), text =
tinymce.trim(body.innerText || body.textContent);
                    return text.trim().split(/\s+/).length;
                }

                function updateRecord(acc)
                {
                        if(jQuery('#titulo').val().length == 0)
                        {
                                jQuery("#saida").html(ShowError("Digite o
título da ocorrência"));
                                return;
                        }

                        if(getWordCount('descricao') < 5) {
                                jQuery("#saida").html(ShowError("Informe
pelo menos 5 palavras para a descrição."));
                                return;
                        }

                        if(jQuery("#path_ocorrencias").val() == "") {
                                jQuery("#saida").html(ShowError("É
obrigatório o cadastro da imagem da complicação."));
                                return;
                        }

                        jQuery("#saida").html(ShowInfo("Aguarde..."));

                        jQuery.ajax({
                                type: "POST",
                                url: "php/pages/admin_complicacoes.php",
                                data: {id : jQuery("#codigo").val(), px :
occurency.x, py : occurency.y, title : jQuery('#titulo').val(), content :
tinyMCE.get('descricao').getContent(), path :
jQuery("#path_ocorrencias").val(), action : acc}
                        })
                        .done(function(html) {
                                if(html == "ok")

jQuery("#saida").html(ShowSuccess("Dados salvos com sucesso!"));
                                else
                                   jQuery("#saida").html(ShowError("Erro
ao tentar inserir dados: "+html));
                                ListRecords();
                        });
                }

                function deleteRecord()
                {
                        if(!confirm('Tem certeza que deseja excluir esse
registro? Não será possível recuperar.'))
```

```
                        return;

                      jQuery("#saida").html(ShowInfo("Aguarde..."));

                      jQuery.ajax({
                              type: "POST",
                              url: "php/pages/admin_complicacoes.php",
                              data: {id : jQuery("#codigo").val(),
action : "delete"}
                      })
                      .done(function(html) {
                              if(html == "ok")

jQuery("#saida").html(ShowSuccess("Dados salvos com sucesso!"));
                              else
                                 jQuery("#saida").html(ShowError("Erro
ao tentar inserir dados: "+html));
                              ListRecords();
                      });
              }

              function ListRecords() {

                      jQuery.ajax({
                              type: "POST",
                              url: "php/pages/admin_complicacoes.php",
                              data: {action : "select"}
                      })
                      .done(function(html) {
                              jQuery("#records").html(html);

        $(".rt-toggle .toggle-content").hide();
        $(".rt-toggle .open .toggle-content").show();

        $(".rt-toggle ol li .toggle-head").click(function(){

                clearTimeout("accordion_timeout");

                var element = $(this).parent("li"),
                        content = element.find(".toggle-content");

                if( element.hasClass("open")){
                        element.removeClass("open");
                        content.stop().slideUp(300);

                }else{


        $(this).parents("ol").find("li.open").removeClass("open").find(".
toggle-content").stop().slideUp(300);

                        element.addClass("open");
                        content.stop().slideDown(300,function(){
                                fix_accordion_pos();
                        });

                        //fixed heights

        content.find('.fixed_heights').rt_fixed_rows("load");

                        //fixed footers
                        $('[data-
footer="fixed_footer"]').rt_fixed_footers();

                }
```

```
                function fix_accordion_pos(){
                        if( $(window).scrollTop() > element.offset().top
){
                                var accordion_timeout =
setTimeout(function() {
                                        var add =
$("#wpadminbar").outerHeight() + $(".top-header.stuck").outerHeight();
                                        rt_scroll_to(
element.offset().top - add, "");
                                }, 100 );
                        }
                }

        });
                        });
                }
        </script>




                <canvas id="game" style='border: 1px solid
#555'></canvas>

                <br/><br/>
                <label>Envie uma imagem da ocorrência</label><br/>
                <?php echo AddFiles("Enviar imagem",
"jpg,jpeg,png,gif,bmp", 1, "ocorrencias", false); ?>

                <label>Titulo da ocorrência</label><br/>
                <input type="text" id='titulo' style='width:
800px;'/><br/><br/>

                <label>Descrição da ocorrência</label><br/>
                <div style='width: 800px;' id='area'><textarea
id='descricao' class='area'></textarea></div>
                <br/><br/>



                <input type='hidden' value='0' id='codigo' />
                <input type='button' class='mybt'
onclick="javascript:updateRecord('add');" value='Adicionar Complicação'
/> <input style='margin-left: 300px; display: none;' class='mybt'
id='btup' type='button' onclick="javascript:updateRecord('update');"
value='Atualizar Complicação' /><br/><br/>
                <div id='saida'></div>
                <br/><br/>
                <div id='records'></div>



                                </div>
                        </div>
                </div>
        </div>
</div>


  <script type='text/javascript'>

        jQuery(document).ready(function() {
                onRunning = ListRecords; Preload();
        });
```

```
tinymce.init({
  selector: '.area',
  height: 300,
  menubar: false,
  plugins: [
    'link image charmap preview pagebreak',
    'wordcount lists contextmenu',
    'table',
    'paste imagetools'
  ],
  language: 'pt_BR',
  image_title: false,
  automatic_uploads: true,
  images_upload_url: 'postAcceptor.php',
  file_picker_types: 'image',
  file_picker_callback: function(cb, value, meta) {
    var input = document.createElement('input');
    input.setAttribute('type', 'file');
    input.setAttribute('accept', 'image/*');


    input.onchange = function() {
      var file = this.files[0];

      var id = 'blobid' + (new Date()).getTime();
      var blobCache = tinymce.activeEditor.editorUpload.blobCache;
      var blobInfo = blobCache.create(id, file);
      blobCache.add(blobInfo);

      cb(blobInfo.blobUri(), { title: file.name });
    };

    input.click();
  },

  toolbar: 'undo redo | cut copy paste | bold italic underline subscript
superscript | alignleft aligncenter alignright alignjustify | bullist |
table link unlink | charmap | preview',
  content_css: 'js/tinymce/codepen.min.css?bogus=' + new Date().getTime()
});


</script>


<?php

                function AddFiles($buttonText, $allowedExt, $codigo, $id,
$rename = true)
                {
                        if($allowedExt != "")
                          $allowedExt = "mime_types : [{ title :
'Arquivos $allowedExt', extensions : '$allowedExt' }], ";

                        $html = "<input type='hidden' value='0'
id='submit_$id'/><input type='hidden' value='' id='path_$id'/><input
type='button' class='mybt' onclick='javascript:void(0);' id='$id'
value='$buttonText' /> <br/><br/><div id='msg_$id'></div>";
                        $html .= "<script type='text/javascript'>

                                        var MyFileName_$id = '';
```

```
                                        var uploader_$id = new
plupload.Uploader({
                                                browse_button: '$id',
                                                url :
'js/plupload/upload.php?path=',
                                                filters: {
                                                        $allowedExt
                                                        max_file_size:
'50mb'
                                                }
                                        });

                                        uploader_".$id.".init();



        uploader_".$id.".bind('FilesAdded', function(up, files) {

                                                var html = '';

                                                plupload.each(files,
function(file) {
                                                        var fileName =
file.name;


        uploader_".$id.".settings.url =
'js/plupload/upload.php?path=images/$id/';
                                                        MyFileName_$id =
'images/$id/'+fileName;

        document.getElementById('msg_$id').innerHTML +=
ShowInfo('Enviando <b>' +file.name+'</b> (<b id=\"'+file.id+'\"></b> de
'+plupload.formatSize(file.size)+')');
                                                });



        document.getElementById('msg_$id').innerHTML += html;
                                                uploader_".$id.".start();
                                        });


        uploader_".$id.".bind('UploadProgress', function(up, file) {

        document.getElementById(file.id).innerHTML = file.percent + '%';
                                        });

                                        uploader_".$id.".bind('Error',
function(up, err) {
                                                if(err.code == '-601')

        document.getElementById('msg_$id').innerHTML = ShowError('Arquivo
enviado não é um formato válido: '+err.file.name);
                                                else

        document.getElementById('msg_$id').innerHTML = ShowError(err.code
+ ': ' + err.message);

        document.getElementById('submit_$id').value = '0';
                                        });


        uploader_".$id.".bind('FileUploaded', function(up, file, info){
```

```
        document.getElementById('msg_$id').innerHTML =
ShowSuccess(file.name+' enviado com sucesso!')+'<br/><a
href=\"'+MyFileName_$id+'\" target=\"_blank\">Clique aqui</a> para baixar
o arquivo';

        document.getElementById('submit_$id').value = '1';

        document.getElementById('path_$id').value = MyFileName_$id;
                                        });
                                </script>";

                        $html .= "<br/><br/>";

                        return $html;
                }

?>
```

```
<div  class="content_row row vc_row wpb_row vc_row-fluid default-style
fullwidth" style="position:relative;overflow:hidden;">
        <div class="rt-parallax-background" data-rt-parallax-
direction="1" data-rt-parallax-effect="horizontal" style="background-
image: url(images/portfolio_header.jpg);background-repeat:
repeat;background-size: cover;background-position: right
top;width:100%;height:100%;top:0;"></div>
        <div class="content_row_wrapper  default" style="padding-
top:25px;padding-bottom:15px;">
                <div class="vc_col-sm-12 wpb_column vc_column_container"
>
                        <div class="wpb_wrapper">
                                <div class="rt_heading_wrapper style-4">
                                        <h1 class="rt_heading  style-4"
>Conheça o SoPeD!</h1>
                                </div>
                        </div>
                </div>
        </div>
</div>
<link rel='stylesheet' href='css/myform.css' type='text/css' media='all'
/>

<style type="text/css">
#myform input[type="text"],
#myform input[type="date"],
#myform input[type="datetime"],
#myform input[type="email"],
#myform input[type="number"],
#myform input[type="search"],
#myform input[type="time"],
#myform input[type="url"],
#myform input[type="password"],
#myform textarea,
#myform select {
```

```
    border-top: 1px solid #f0f0f0;
    border-left: 1px solid #f0f0f0;
    border-bottom: 1px solid #FAFAFA;
    border-right: 1px solid #FAFAFA;
    background: #e0e0e0;
}

.default-style input[type="button"]:hover {background-color: #FC9D0D;}
.default-style input[type="button"] {background-color: #428bca; font-
weight: bold;}

.default-style input[type="button"]:hover.extra  {background-color:
#BBB;}
.default-style input[type="button"].extra  {background-color: #888; font-
weight: bold;}


</style>


<div  class="content_row row vc_row wpb_row vc_row-fluid default-style
fullwidth" >
        <div class="content_row_wrapper  default" style="padding-
top:60px;padding-bottom:0px;">
                                <h2>Conheça algumas das vantagens de usar
o SoPeD</h2>
                <div class="vc_col-sm-4 wpb_column vc_column_container" >
                        <div class="wpb_wrapper">
                                <div class="vc_empty_space"
style="height: 60px" ><span class="vc_empty_space_inner"></span></div>
                                <div  class="chained_contents style-2
right" data-rt-animation-group="group">
                                        <div  class="" data-rt-
animate="animate" data-rt-animation-type="fadeInDown">
                                                <span
class="number">1</span>
                                                <div class="list-
content">
                                                        <p>Melhora
significativa da saúde dos pés já nos primeiros dias</p>
                                                </div>
                                        </div>
                                        <div  class="" data-rt-
animate="animate" data-rt-animation-type="fadeInDown">
                                                <span
class="number">2</span>
                                                <div class="list-
content">
                                                        <p>Acompanhamento
dos seus exercícios e evolução por especialistas.</p>
                                                </div>
                                        </div>
                                        <div  class="" data-rt-
animate="animate" data-rt-animation-type="fadeInDown">
                                                <span
class="number">3</span>
                                                <div class="list-
content">
                                                        <p>Monte seu
cronograma de exercícios e seja notificado automaticamente.</p>
                                                </div>
                                        </div>
                                </div>
                        </div>
```

```
                </div>
                <div class="vc_col-sm-4 wpb_column vc_column_container" >
                        <div class="wpb_wrapper">
                                <div class="wpb_single_image
wpb_content_element vc_align_center">
                                        <figure class="wpb_wrapper
vc_figure">
                                                <div
class="vc_single_image-wrapper   vc_box_border_grey"><img width="400"
height="658" src="images/avatar.png" class="vc_single_image-img
attachment-full" alt="wonen" /></div>
                                        </figure>



                                        <div class="submit-button">
                                                <input class="button
submit" value="Entendi! Mostre me mais..."  type="button">
                                        </div>

                                        <br/><br/>

                                </div>
                        </div>
                </div>
                <div class="vc_col-sm-4 wpb_column vc_column_container" >
                        <div class="wpb_wrapper">
                                <div class="vc_empty_space"
style="height: 60px" ><span class="vc_empty_space_inner"></span></div>
                                <div  class="chained_contents style-2
left" data-rt-animation-group="group">
                                        <div  class="" data-rt-
animate="animate" data-rt-animation-type="fadeInDown">
                                                <span
class="number">4</span>
                                                <div class="list-
content">
                                                        <p>A ferramenta é
completamente <strong>gratuita</strong> e online, ao seu alcance.</p>
                                                </div>
                                        </div>
                                        <div  class="" data-rt-
animate="animate" data-rt-animation-type="fadeInDown">
                                                <span
class="number">5</span>
                                                <div class="list-
content">
                                                        <p>Tire dúvida
com especialistas e fique por dentro das últimas descobertas.</p>
                                                </div>
                                        </div>
                                        <div  class="" data-rt-
animate="animate" data-rt-animation-type="fadeInDown">
                                                <span
class="number">6</span>
                                                <div class="list-
content">
                                                        <p>Compare o seu
rendimento e evolução com outros participantes.</p>
                                                </div>
                                        </div>
                                </div>
                        </div>
                </div>
```

```
        </div>
</div>


<div  class="content_row row vc_row wpb_row vc_row-fluid default-style
fullwidth" style="position:relative;overflow:hidden;">
        <div class="rt-parallax-background" data-rt-parallax-
direction="1" data-rt-parallax-effect="horizontal" style="background-
image: url(images/portfolio_header.jpg);background-repeat:
repeat;background-size: cover;background-position: right
top;width:100%;height:100%;top:0;"></div>
        <div class="content_row_wrapper  default" style="padding-
top:25px;padding-bottom:15px;">
                <div class="vc_col-sm-12 wpb_column vc_column_container"
>
                        <div class="wpb_wrapper">
                                <div class="rt_heading_wrapper style-4">
                                        <h1 class="rt_heading  style-4"
>Área interna do SoPeD</h1><br/>
                                </div>
                        </div>
                </div>
        </div>
</div>





<link rel='stylesheet' href='css/myform.css' type='text/css' media='all'
/>

<style type="text/css">
.myform input[type="text"],
.myform input[type="date"],
.myform input[type="datetime"],
.myform input[type="email"],
.myform input[type="number"],
.myform input[type="search"],
.myform input[type="time"],
.myform input[type="url"],
.myform input[type="password"],
.myform textarea,
.myform select {

    border-top: 1px solid #f0f0f0;
    border-left: 1px solid #f0f0f0;
    border-bottom: 1px solid #FAFAFA;
    border-right: 1px solid #FAFAFA;
    background: #e0e0e0;
}

.alt-style-1 input[type="button"]:hover {background-color: #FC9D0D;}
.alt-style-1 input[type="button"] {background-color: #428bca; font-
weight: bold;}

.alt-style-1 input[type="button"]:hover.extra  {background-color:
#FC9D0D;}
.alt-style-1 input[type="button"].extra  {background-color: #00AA00;
font-weight: bold;}
</style>
```

```
<?php include("php/pages/internal.php"); ?>



<div  class="content_row alt-style-1 no-composer overlap fullwidth">
        <div class="content_row_wrapper clearfix ">
                <div class="col col-sm-12 col-xs-12 ">
                        <div class="single post-1872 staff type-staff 
status-publish has-post-thumbnail hentry" id="person-1872">

                                <h2>Lista de exercícios disponíveis - 3
</h2><br/>

                                <div id="mytable">
                                <table>
                                        <tr>
                                                <th>Exercício</th>
                                                <th>Descrição e vantagens
de executar</th>
                                                <th>Histórico de
execução</th>
                                        </tr>


                                        <tr>
                                                <td style='width: 100px;
vertical-align: middle;'>
                                                        <img
src="images/exercicios/exercicio_03.png" />
                                                        <br/><br/><input
class="button submit" onclick="javascript:alert('Bloqueado');"
value="Refazer..." type="button">
                                                </td>
                                                <td style='width:
600px;'>
                                                        <h4 style='color:
#428BCA;'>Alternar o apoio dos dedos</h4>
                                                        <b>Como
executar:</b> Sentado em uma cadeira, com os pés apoiados no chão,
encoste alternadamente o primeiro e o quinto dedos (dedinho e dedão) no
chão.<br/><br/>

                                                        <b style='color:
#428BCA;'>Vantagens:</b><br/>

        <img src="images/exercicios/saude.png" > Relaxamento dos dedos e
liberação de tensão;<br/>

        <img src="images/exercicios/saude.png" > Aumento da sensibilidade
local;
                                                </td>
                                                <td style='vertical-
align: middle;'>
                                                        <b>Repetição
1:</b> <img src="images/exercicios/emotion_cool.png" data-
toggle="tooltip" title="25/04/2016 - Muito fácil"  /><br/><br/>
                                                        <b>Repetição
2:</b> <img src="images/exercicios/emotion_smile.png" data-
toggle="tooltip" title="26/04/2016 - Fácil" /><br/><br/>
```

```
                                                            <b>Repetição
3:</b> <img src="images/exercicios/emotion_smile.png" data-
toggle="tooltip" title="27/04/2016 - fácil" />
                                                    </td>
                                            </tr>


                                            <tr>
                                                    <td style='width: 100px;
vertical-align: middle; text-align: center;'>
                                                            <img
src="images/exercicios/exercicio_01.png" />
                                                            <br/><br/><input
class="button submit" onclick="javascript:alert('Bloqueado');"
value="Refazer..." type="button">
                                                    </td>
                                                    <td style='width:
600px;'>
                                                            <h4 style='color:
#428BCA;'>Subir na ponta dos pés</h4>
                                                            <b>Como
executar:</b> Utilizando um apoio, cadeira ou qualquer outro móvel
estável, fique na ponta dos pés e volte para a posição inicial.<br/><br/>
                                                            <b style='color:
#428BCA;'>Vantagens:</b><br/>

        <img src="images/exercicios/saude.png" > Aumento visível da
musculatura e da força do pé;<br/>

        <img src="images/exercicios/saude.png" > Diminuição da sensação
de dormência;
                                                    </td>
                                                    <td style='vertical-
align: middle;'>
                                                            <b>Repetição
1:</b> <img src="images/exercicios/emotion_smile.png" data-
toggle="tooltip" title="02/05/2016 - Fácil" /><br/><br/>
                                                            <b>Repetição
2:</b> <img src="images/exercicios/emotion_amazing.png" data-
toggle="tooltip" title="03/04/2016 - Razoável" /><br/><br/>
                                                            <b>Repetição
3:</b> <img src="images/exercicios/emotion_amazing.png" data-
toggle="tooltip" title="25/04/2016 - Muito Difícil" />

                                                    </td>
                                            </tr>

                                            <tr>
                                                    <td style='width: 100px;
vertical-align: middle;'>
                                                            <img
src="images/exercicios/exercicio_02.png" />
                                                            <br/><br/><input
class="button submit extra "  onclick="javascript:alert('Bloqueado');"
value="Executar..." type="button">

                                                    </td>
                                                    <td style='width:
600px;'>
                                                            <h4 style='color:
#428BCA;'>Abaular o pé</h4>
                                                            <b>Como
executar:</b> Sentado, “encurvar” o pé como se fosse encolhê-lo, mantendo
o calcanhar e as pontas dos dedos dos pés no chão.<br/><br/>
```

```
                                                                <b style='color:
#428BCA;'>Vantagens:</b><br/>

        <img src="images/exercicios/saude.png" > Diminuição da dores no
canto inferior do pé;<br/>

        <img src="images/exercicios/saude.png" > Diminuição da sensação
de dormência;

                                                        </td>
                                                        <td style='vertical-
align: middle;'>
                                                                <b>Repetição
1:</b> <img src="images/exercicios/emotion_question.png" data-
toggle="tooltip" title="Pendente"  /><br/><br/>
                                                                <b>Repetição
2:</b> <img src="images/exercicios/emotion_question.png" data-
toggle="tooltip" title="Pendente" /><br/><br/>
                                                                <b>Repetição
3:</b> <img src="images/exercicios/emotion_question.png" data-
toggle="tooltip" title="Pendente" />
                                                        </td>
                                                </tr>




                                </table>
                                </div>

                        </div>
                </div>


        </div>
</div>







<scri



<div  class="content_row row vc_row wpb_row vc_row-fluid default-style
fullwidth" style="position:relative;overflow:hidden;">
        <div class="rt-parallax-background" data-rt-parallax-
direction="1" data-rt-parallax-effect="horizontal" style="background-
image: url(images/portfolio_header.jpg);background-repeat:
repeat;background-size: cover;background-position: right
top;width:100%;height:100%;top:0;"></div>
        <div class="content_row_wrapper  default" style="padding-
top:25px;padding-bottom:15px;">
                <div class="vc_col-sm-12 wpb_column vc_column_container"
>
                        <div class="wpb_wrapper">
                                <div class="rt_heading_wrapper style-4">
                                        <h1 class="rt_heading  style-4"
>Questionário para avaliação dos especialistas</h1>
                                </div>
                        </div>
                </div>
        </div>
```

```
</div>


<style>
        label{font-weight: normal;}
</style>



<style type="text/css">


.alt-style-1 input[type="button"]:hover {background-color: #FC9D0D;}
.alt-style-1 input[type="button"] {background-color: #428bca; font-
weight: bold;}

.alt-style-1 input[type="button"]:hover.extra  {background-color: #BBB;}
.alt-style-1 input[type="button"].extra  {background-color: #888; font-
weight: bold;}

b span {color: #428BCA;}


</style>

<div  class="content_row default-style no-composer overlap fullwidth">
        <div class="content_row_wrapper clearfix " style='min-height:
800px;'>
                <div class="col col-sm-12 col-xs-12 ">
                        <div class="single post-1872 staff type-staff
status-publish has-post-thumbnail hentry" id="person-1872">




                                <br/><h2>Questionário respondido com
sucesso!</h2><br/>


                                <p>Agradecemos por ajudar a tornar o
SoPeD cada melhor! Suas respostas será enviada para nossa equipe de
desenvolvimento.</p>




                        </div>
                </div>


        </div>
</div>




<div  class="content_row row vc_row wpb_row vc_row-fluid default-style
fullwidth" style="position:relative;overflow:hidden;">
        <div class="rt-parallax-background" data-rt-parallax-
direction="1" data-rt-parallax-effect="horizontal" style="background-
image: url(images/portfolio_header.jpg);background-repeat:
repeat;background-size: cover;background-position: right
top;width:100%;height:100%;top:0;"></div>
        <div class="content_row_wrapper  default" style="padding-
top:25px;padding-bottom:15px;">
```

```
                <div class="vc_col-sm-12 wpb_column vc_column_container"
>
                        <div class="wpb_wrapper">
                                <div class="rt_heading_wrapper style-4">
                                        <h1 class="rt_heading  style-4"
>Sign up for SoPeD!</h1>
                                </div>
                        </div>
                </div>
        </div>
</div>
<link rel='stylesheet' href='css/myform.css' type='text/css' media='all'
/>

<style type="text/css">
.alt-style-1 input[type="button"]:hover {background-color: #FC9D0D;}
.alt-style-1 input[type="button"] {background-color: #428bca; font-
weight: bold;}

.alt-style-1 input[type="button"]:hover.extra  {background-color: #BBB;}
.alt-style-1 input[type="button"].extra  {background-color: #888; font-
weight: bold;}
</style>


        <script src="js/jquery.maskedinput.js"
type="text/javascript"></script>


        <script type='text/javascript'>
                jQuery(document).ready(function() {
                        jQuery('.date').mask('9999/99/99');
                        jQuery('.year').mask('9999');
                });
        </script>


<div  class="content_row row vc_row wpb_row vc_row-fluid alt-style-1
fullwidth" >
        <div class="content_row_wrapper  default" >
                <div class="vc_col-sm-12 wpb_column vc_column_container"
>
                        <div class="wpb_wrapper">
                                <div class="rt_heading_wrapper style-6">
                                        <h5 class="rt_heading  style-6"
>Fill the form below to start with SoPeD</h5>
                                </div>
                                Signing up is quick and easy. No e-mail
confirmation is required.

                                <br/><br/>
                                <div class="clear"></div>


                                        <form action="#"
name="contact_form" class="validate_form rt_form" method="post"
id="myform" style="margin-left: -15px;">

                                                <div class="vc_col-sm-8
wpb_column vc_column_container" >
                                                        <label>Full
Name</label>
                                                        <input
type="text" name="nome" />
                                                </div>
```

```
<div class="vc_col-sm-2 wpb_column vc_column_container" >
        <label>Birthday date (yyyy/mm/dd)</label>
        <input type="email" class="date" name="nome" />
</div>
<div class="vc_col-sm-2 wpb_column vc_column_container" >
        <label>Gender</label>
        <select size="1" name="tipo">
                <option value="M">Male</option>
                <option value="F">Female</option>
        </select>
</div>
<div class="clear"></div><br/>

<div class="vc_col-sm-4 wpb_column vc_column_container" >
        <label>E-mail</label>
        <input type="email" name="nome" />
</div>
<div class="vc_col-sm-4 wpb_column vc_column_container" >
        <label>Password for access</label>
        <input type="password" name="telefone" />
</div>
<div class="vc_col-sm-4 wpb_column vc_column_container" >
        <label>Type your Password again:</label>
        <input type="password" name="telefone" />
</div>
<div class="clear"></div><br/>
<div class="vc_col-sm-2 wpb_column vc_column_container" >
        <label>Diabetic?</label>
        <select size="1" name="tipo">
                <option value="Não">No</option>
                <option value="Sim">Yes</option>
        </select>
```

```
</div>
<div class="vc_col-sm-2
wpb_column vc_column_container" >
<label>Year of
Diagnosis</label>
<input
type="text" class="year" name="ano" />
</div>

<div class="vc_col-sm-4
wpb_column vc_column_container" >
<label>Cell
phone</label>
<input
type="email" class="phone" name="nome" />
</div>

<div class="vc_col-sm-4
wpb_column vc_column_container" >
<label>Home
phone</label>
<input
type="email" class="phone" name="nome" />
</div>

<div
class="clear"></div><br/>

<div class="vc_col-sm-4
wpb_column vc_column_container" >
<label>Country
</label>
<select size="1" name="endereco" id="endereco" class="input-text"
style="height: 40px; width: 100%">
<option>Afghanistan</option>

<option>Albania</option>

<option>Algeria</option>

<option>American Samoa</option>

<option>Andorra</option>

<option>Angola</option>

<option>Anguilla</option>

<option>Antarctica</option>

<option>Antigua and Barbuda</option>

<option>Argentina</option>

<option>Armenia</option>

<option>Aruba</option>

<option>Australia</option>
```

```
<option>Austria</option>
<option>Azerbaijan</option>
<option>Bahamas</option>
<option>Bahrain</option>
<option>Bangladesh</option>
<option>Barbados</option>
<option>Belarus</option>
<option>Belgium</option>
<option>Belize</option>
<option>Benin</option>
<option>Bermuda</option>
<option>Bhutan</option>
<option>Bolivia</option>
<option>Bosnia and Herzegovina</option>
<option>Botswana</option>
<option>Bouvet Island</option>

<option>British Indian Ocean Territory</option>
<option>Brunei Darussalam</option>
<option>Bulgaria</option>
<option>Burkina Faso</option>
<option>Burundi</option>
<option>Cambodia</option>
<option>Cameroon</option>
<option>Canada</option>
<option>Cape Verde</option>
<option>Cayman Islands</option>
<option>Central African Republic</option>
<option>Chad</option>
<option>Chile</option>
<option>China</option>
<option>Christmas Island</option>
<option>Cocos Islands</option>
```

```
<option>Colombia</option>
<option>Comoros</option>
<option>Congo</option>
<option>Congo, Democratic Republic of the</option>
<option>Cook Islands</option>
<option>Costa Rica</option>
<option>Cote d'Ivoire</option>
<option>Croatia</option>
<option>Cuba</option>
<option>Cyprus</option>
<option>Czech Republic</option>
<option>Denmark</option>
<option>Djibouti</option>
<option>Dominica</option>
<option>Dominican Republic</option>
<option>Ecuador</option>
<option>Egypt</option>
<option>El Salvador</option>
<option>Equatorial Guinea</option>
<option>Eritrea</option>
<option>Estonia</option>
<option>Ethiopia</option>
<option>Falkland Islands</option>
<option>Faroe Islands</option>
<option>Fiji</option>
<option>Finland</option>
<option>France</option>
<option>French Guiana</option>
<option>French Polynesia</option>
<option>Gabon</option>
<option>Gambia</option>
<option>Georgia</option>
<option>Germany</option>
```

```
<option>Ghana</option>
<option>Gibraltar</option>
<option>Greece</option>
<option>Greenland</option>
<option>Grenada</option>
<option>Guadeloupe</option>
<option>Guam</option>
<option>Guatemala</option>
<option>Guinea</option>
<option>Guinea-Bissau</option>
<option>Guyana</option>
<option>Haiti</option>
<option>Heard Island and McDonald Islands</option>
<option>Honduras</option>
<option>Hong Kong</option>
<option>Hungary</option>
<option>Iceland</option>
<option>India</option>
<option>Indonesia</option>
<option>Iran</option>
<option>Iraq</option>
<option>Ireland</option>
<option>Israel</option>
<option>Italy</option>
<option>Jamaica</option>
<option>Japan</option>
<option>Jordan</option>
<option>Kazakhstan</option>
<option>Kenya</option>
<option>Kiribati</option>
<option>Kuwait</option>
<option>Kyrgyzstan</option>
```

```
<option>Laos</option>
<option>Latvia</option>
<option>Lebanon</option>
<option>Lesotho</option>
<option>Liberia</option>
<option>Libya</option>
<option>Liechtenstein</option>
<option>Lithuania</option>
<option>Luxembourg</option>
<option>Macao</option>
<option>Madagascar</option>
<option>Malawi</option>
<option>Malaysia</option>
<option>Maldives</option>
<option>Mali</option>
<option>Malta</option>
<option>Marshall Islands</option>
<option>Martinique</option>
<option>Mauritania</option>
<option>Mauritius</option>
<option>Mayotte</option>
<option>Mexico</option>
<option>Micronesia</option>
<option>Moldova</option>
<option>Monaco</option>
<option>Mongolia</option>
<option>Montenegro</option>
<option>Montserrat</option>
<option>Morocco</option>
<option>Mozambique</option>
<option>Myanmar</option>
<option>Namibia</option>
<option>Nauru</option>
```

```
<option>Nepal</option>
<option>Netherlands</option>
<option>Netherlands Antilles</option>
<option>New Caledonia</option>
<option>New Zealand</option>
<option>Nicaragua</option>
<option>Niger</option>
<option>Nigeria</option>
<option>Norfolk Island</option>
<option>North Korea</option>
<option>Norway</option>
<option>Oman</option>
<option>Pakistan</option>
<option>Palau</option>
<option>Palestinian Territory</option>
<option>Panama</option>
<option>Papua New Guinea</option>
<option>Paraguay</option>
<option>Peru</option>
<option>Philippines</option>
<option>Pitcairn</option>
<option>Poland</option>
<option>Portugal</option>
<option>Puerto Rico</option>
<option>Qatar</option>
<option>Romania</option>
<option>Russian Federation</option>
<option>Rwanda</option>
<option>Saint Helena</option>
<option>Saint Kitts and Nevis</option>
<option>Saint Lucia</option>
<option>Saint Pierre and Miquelon</option>
```

```
<option>Saint Vincent and the Grenadines</option>
<option>Samoa</option>
<option>San Marino</option>
<option>Sao Tome and Principe</option>
<option>Saudi Arabia</option>
<option>Senegal</option>
<option>Serbia</option>
<option>Seychelles</option>
<option>Sierra Leone</option>
<option>Singapore</option>
<option>Slovakia</option>
<option>Slovenia</option>
<option>Solomon Islands</option>
<option>Somalia</option>
<option>South Africa</option>
<option>South Georgia</option>
<option>South Korea</option>
<option>Spain</option>
<option>Sri Lanka</option>
<option>Sudan</option>
<option>Suriname</option>
<option>Svalbard and Jan Mayen</option>
<option>Swaziland</option>
<option>Sweden</option>
<option>Switzerland</option>
<option>Syrian Arab Republic</option>
<option>Taiwan</option>
<option>Tajikistan</option>
<option>Tanzania</option>
<option>Thailand</option>
<option>The Former Yugoslav Republic of Macedonia</option>
<option>Timor-Leste</option>
<option>Togo</option>
```

```
<option>Tokelau</option>
<option>Tonga</option>
<option>Trinidad and Tobago</option>
<option>Tunisia</option>
<option>Turkey</option>
<option>Turkmenistan</option>
<option>Tuvalu</option>
<option>Uganda</option>
<option>Ukraine</option>
<option>United Arab Emirates</option>
<option>United Kingdom</option>
<option>United States</option>
<option>United States Minor Outlying Islands</option>
<option>Uruguay</option>
<option>Uzbekistan</option>
<option>Vanuatu</option>
<option>Vatican City</option>
<option>Venezuela</option>
<option>Vietnam</option>
<option>Virgin Islands, British</option>
<option>Virgin Islands, U.S.</option>
<option>Wallis and Futuna</option>
<option>Western Sahara</option>
<option>Yemen</option>
<option>Zambia</option>
<option>Zimbabwe</option>
</select>
                                                </div>

                                                <div class="vc_col-sm-4
wpb_column vc_column_container" >

                                                        <label>Province /
State</label>
                                                        <input
type="text" name="estado" />
```

```
                                                            </select>
                                                    </div>


                                                    <div class="vc_col-sm-4
wpb_column vc_column_container" >

        <label>City</label>
                                                            <input
type="text" name="cidade" />
                                                    </div>

                                                    <div
class="clear"></div><br/>

                                                    <div class="vc_col-sm-12
wpb_column vc_column_container" >
                                                            <label>Are you
the bearer of another diseases?</label>
                                                            <textarea
style="height: 150px;"></textarea>
                                                    </div>
                                            </form>

                                            <div class="clear"></div><br/>

                                            By clicking 'Sign up for SoPeD'
you agree all <a href="">registration with the training option</a> of the
SoPeD tool.

                                            <div class="clear"></div><br/>

                                            <div class="submit-button"
style='text-align: center;'>
                                                    <input class="button
submit" value="Sign up for SoPeD" type="button"><span
class="loading"></span>
                                            </div>

                                    <div class="clear"></div>

                            </div>
                    </div>
            </div>
    </div>




<div  class="content_row row vc_row wpb_row vc_row-fluid default-style
fullwidth border_grid fixed_heights" >

        <div class="content_row_wrapper  default" style="padding-
top:40px;padding-bottom:40px;"><div class="vc_col-sm-4 wpb_column
vc_column_container vc_custom_1421091415717" >
                <div class="wpb_wrapper">
                        <article  class="icon-content-box content-box
icon-top icon-style-3"><div class="icon-holder"><a href="#" title=""
target="_self"><span class="icon-new-link-1"></span></a></div><div
class="text-holder">
        <h4 class="heading">
                <a href="#" title="Box Heading" target="_self">
                        Why use SoPeD?
                </a>
```

```
        </h4> <p class="aligncenter">Besides being an easy and free tool,
SoPeD adjusts to the patient schedules and promotes notable improving
wellbeing of the feet.</p>
</div></article>
                </div>
        </div>

        <div class="vc_col-sm-4 wpb_column vc_column_container" >
                <div class="wpb_wrapper">
                        <article  class="icon-content-box content-box
icon-top icon-style-3"><div class="icon-holder"><a href="#" title=""
target="_self"><span class="icon-new-user-1"></span></a></div><div
class="text-holder">
        <h4 class="heading">
                <a href="#" title="Box Heading" target="_self">
                        Talk to the doctor!
                </a>
        </h4> <p class="aligncenter">In case of doubt get in touch with
our online experts and ask your questions. We are always ready to serve
you!</p>
</div></article>
                </div>
        </div>

        <div class="vc_col-sm-4 wpb_column vc_column_container" >
                <div class="wpb_wrapper">
                        <article  class="icon-content-box content-box
icon-top icon-style-3"><div class="icon-holder"><a href="#" title=""
target="_self"><span class="icon-new-heart-1"></span></a></div><div
class="text-holder">
        <h4 class="heading">
                <a href="#" title="Box Heading" target="_self">
                        Do you liked it? Share it!
                </a>
        </h4> <p class="aligncenter">Share SoPeD to friends and people in
your personal network. You will be helping to improve the tool.</p>
</div></article>
                </div>
        </div>
</div>
</div>



<div  class="content_row row vc_row wpb_row vc_row-fluid default-style
fullwidth" style="position:relative;overflow:hidden;">
        <div class="rt-parallax-background" data-rt-parallax-
direction="1" data-rt-parallax-effect="horizontal" style="background-
image: url(images/portfolio_header.jpg);background-repeat:
repeat;background-size: cover;background-position: right
top;width:100%;height:100%;top:0;"></div>
        <div class="content_row_wrapper  default" style="padding-
top:25px;padding-bottom:15px;">
                <div class="vc_col-sm-12 wpb_column vc_column_container"
>
                        <div class="wpb_wrapper">
                                <div class="rt_heading_wrapper style-4">
                                        <h1 class="rt_heading  style-4"
>Pergunte aos especialistas</h1>
                                </div>
                        </div>
                </div>
        </div>
</div>
```

```
<link rel='stylesheet' href='css/myform.css' type='text/css' media='all'
/>

<style type="text/css">
#myform input[type="text"],
#myform input[type="date"],
#myform input[type="datetime"],
#myform input[type="email"],
#myform input[type="number"],
#myform input[type="search"],
#myform input[type="time"],
#myform input[type="url"],
#myform input[type="password"],
#myform textarea,
#myform select {

    border-top: 1px solid #f0f0f0;
    border-left: 1px solid #f0f0f0;
    border-bottom: 1px solid #FAFAFA;
    border-right: 1px solid #FAFAFA;
    background: #e0e0e0;
}
</style>


<div  class="content_row row vc_row wpb_row vc_row-fluid default-style
fullwidth" >
        <div class="content_row_wrapper  default" >
                <div class="vc_col-sm-12 wpb_column vc_column_container"
>
                        <div class="wpb_wrapper">
                                <div class="rt_heading_wrapper style-6">
                                        <h5 class="rt_heading  style-6"
>Dúvidas? Envie para nossos especialistas!</h5>
                                </div>
                                 Agradecemos o seu interesse no projeto!
Escreva-nos e entraremos em contato o mais breve possível.

                                <br/><br/>
                                <div class="clear"></div>


                                        <form action="#"
name="contact_form" class="validate_form rt_form" method="post"
id="myform" style="margin-left: -15px;">

                                                <div class="vc_col-sm-4
wpb_column vc_column_container" >
                                                        <label>Nome
completo</label>
                                                        <input
type="text" name="nome" />
                                                </div>

                                                <div class="vc_col-sm-4
wpb_column vc_column_container" >
                                                        <label>E-
mail</label>
                                                        <input
type="email" name="nome" />
                                                </div>

                                                <div class="vc_col-sm-4
wpb_column vc_column_container" >
```

```
                                                        <label>Tipo de
comentário</label>
                                                        <select size="1"
name="tipo">
        <option>Público</option>
        <option>Anônimo</option>
                                                        </select>
                                                </div>

                                                <div
class="clear"></div><br/>

                                                <div class="vc_col-sm-12
wpb_column vc_column_container" >
                                                        <label>Digite sua
pergunta</label>
                                                        <textarea
style="height: 150px;"></textarea>
                                                </div>
                                        </form>

                                        <div
class="clear"></div><br/><br/>
                                        <div class="submit-button">
                                                <input class="button
submit" value="Enviar Pergunta" type="button"><span
class="loading"></span>
                                        </div>

                                <div class="clear"></div>

                        </div>
                </div>
        </div>
</div>



<div  class="content_row row vc_row wpb_row vc_row-fluid alt-style-1
fullwidth" >
        <div class="content_row_wrapper  default" >
                <div class="vc_col-sm-12 wpb_column vc_column_container"
>
                        <div class="wpb_wrapper">
                                <section id="staff-700135" class="team
border_grid clearfix  ">
                                        <h2>Conheça nossos
especialistas</h2>
                                        <div class="row clearfix">
                                                <div class="col col-sm-
4">
                                                        <div class="loop
post-1873 staff type-staff status-publish has-post-thumbnail hentry"
id="person-1873">
                                                                <div
class="person_image">
        <div class="person_image_wrapper">
                                                                                <a href=
        </div>
                                                                </div>
```

```
                                                                <h5
class="person_name"><a href="">Nutricionista</a></h5>
                                                                <span
class="position">Colaborador</span>
                                                                <div
class="profile">

                                                                </div>
                                                        </div>
                                                </div>

                                                <div class="col col-sm-
4">
                                                        <div class="loop
post-1873 staff type-staff status-publish has-post-thumbnail hentry"
id="person-1873">
                                                                <div
class="person_image">

        <div class="person_image_wrapper">
                                                                                <a href=

        </div>
                                                                </div>
                                                                <h5
class="person_name"><a href="">Endocrinologista</a></h5>
                                                                <span
class="position">Colaborador</span>
                                                                <div
class="profile">

                                                                </div>
                                                        </div>
                                                </div>


                                                <div class="col col-sm-
4">
                                                        <div class="loop
post-1873 staff type-staff status-publish has-post-thumbnail hentry"
id="person-1873">
                                                                <div
class="person_image">

        <div class="person_image_wrapper">
                                                                                <a href=

        </div>
                                                                </div>
                                                                <h5
class="person_name"><a href="">Fisioterapeuta</a></h5>
                                                                <span
class="position">Colaborador</span>
                                                                <div
class="profile">

                                                                </div>
                                                        </div>
                                                </div>



                                        </div>
                                </section>
                        </div>
```

```
                </div>
        </div>
</div>
<div  class="content_row row vc_row wpb_row vc_row-fluid default-style
fullwidth" style="position:relative;overflow:hidden;">
        <div class="rt-parallax-background" data-rt-parallax-
direction="1" data-rt-parallax-effect="horizontal" style="background-
image: url(images/portfolio_header.jpg);background-repeat:
repeat;background-size: cover;background-position: right
top;width:100%;height:100%;top:0;"></div>
        <div class="content_row_wrapper  default" style="padding-
top:25px;padding-bottom:15px;">
                <div class="vc_col-sm-12 wpb_column vc_column_container"
>
                        <div class="wpb_wrapper">
                                <div class="rt_heading_wrapper style-4">
                                        <h1 class="rt_heading  style-4"
>Simulação de Protocolo</h1>
                                </div>
                        </div>
                </div>
        </div>
</div>



<style type="text/css">
        h4 span {color: #428BCA;}
</style>



<div  class="content_row row vc_row wpb_row vc_row-fluid alt-style-1
fullwidth border_grid fixed_heights" >
        <div class="content_row_wrapper  default" style="padding-
top:50px;padding-bottom:50px; min-height: 500px;">
                <div class="vc_col-sm-12 wpb_column vc_column_container"
>
                        <div class="wpb_wrapper">

                                <!--
                                <div class="rt_heading_wrapper style-6">
                                        <h4 class="rt_heading  style-6"
><span class="icon-glyph-15 heading_icon"></span>Protocolo de
Execícios</h4>
                                </div>
                                <div class="wpb_text_column
wpb_content_element ">
                                        <div class="wpb_wrapper">
                                                <p><b>Execute o exercício
e informe a dificuldade.</b></p>
                                        </div>
                                </div>
                                -->

                                <?php
                                        $exCount = 0;

                                        $user_id = 1;
                                        $position = 1;

                                        require_once('php/MysqlDB.php');
                                        $Obj = new ScriptDB();
```

```
                                        if(isset($_GET['start']))
                                        {
                                                $Obj->ExecQuery("DELETE
FROM saedd_protocol WHERE user_id = '$user_id'");
                                        }

                                        $Obj->ExecQuery("SELECT COUNT(*)
FROM saedd_protocol WHERE user_id = '$user_id'");


                                        if(intval($Obj->GetQueryValue(0,
0)) == 0)
                                        {
                                                $sql = "INSERT INTO
saedd_protocol(block_id, position, number, difficult, user_id,
registration_date) SELECT block, saedd_blocks.position, exercise, NULL,
'$user_id', NULL FROM saedd_blocks WHERE target = COALESCE((SELECT target
FROM saedd_users WHERE id = '$user_id'), NULL, 1)";

                                                if(!$Obj-
>ExecQuery($sql))
                                                  echo "<h2>Erro ao
inserir dados de execícios.</h2>";
                                        }

                                        $Obj->ExecQuery("SELECT
MAX(position) FROM saedd_protocol WHERE user_id = '$user_id'");
                                        $exCount = intval($Obj-
>GetQueryValue(0, 0));

                                        if(isset($_POST['difficult']))
                                        {
                                                $Obj->Set("TableName",
"saedd_protocol");
                                                $Obj->Set("FieldsName",
"difficult|registration_date|id");
                                                $Obj-
>BeforeUpdate("registration_date","NOW()");
                                                $Obj->Set("PrimaryKey",
"id");

                                                $sql = $Obj-
>CreateUpdate();

                                                $Obj->Connect();
                                                $Obj->ExecQuery($sql);
                                        }

                                        $sql = "SELECT position,
block_id, (SELECT max(position) FROM saedd_protocol a WHERE a.block_id =
b.block_id) FROM saedd_protocol b WHERE user_id = '$user_id' AND
registration_date IS NOT NULL ORDER BY registration_date DESC, block_id,
position LIMIT 1";
                                        $Obj->ExecQuery($sql);
                                        $count = $Obj-
>Get("RecordCount");

                                        if($count == 0 || intval($Obj-
>GetQueryValue(0, 0)) == intval($Obj->GetQueryValue(0, 2)))
                                          $position = 1;
                                        else
                                          $position = $Obj-
>GetQueryValue(0, 0)+1;
```

```
                                        $sql = "SELECT id,  (SELECT title
FROM saedd_exercises WHERE code = number), block_id, number, difficult,
(SELECT evolution FROM saedd_exercises WHERE code = number) FROM
saedd_protocol WHERE user_id = '$user_id' AND position = '$position' AND
block_id = (SELECT MIN(block_id) FROM saedd_protocol WHERE (difficult IS
NULL OR difficult >= 5.0) AND position = '$position') ORDER BY block_id,
position";
                                        $Obj->ExecQuery($sql);

                                        $count = $Obj-
>Get("RecordCount");

                                        if($count != 0)
                                        {
                                           $id = $Obj->GetQueryValue(0,
0);
                                           $block = $Obj-
>GetQueryValue(0, 2);
                                           $title = $Obj-
>GetQueryValue(0, 3) . " - " . $Obj->GetQueryValue(0, 1);
                                           $hard = $Obj->GetQueryValue(0,
4);
                                           $evo = $Obj->GetQueryValue(0,
5);

                                           echo "<h2>Bloco $block - Grupo
$position</h2>";
                                           echo "<h4><span>Exercício
$title</span></h4>";
                                           echo "<h4><span style='color:
#22CC33;'>$evo</span></h4><br/><br/>";
                                           if($hard != "")
                                             echo "<b>Última tentativa
(Dificuldade):</b> $hard ";
                                            echo "<br/><br/>";
                                        }
                                        else
                                        {
                                            echo "<h4><span>Parabéns você
concluiu todos os exercícios!</span></h4>
                                                <p>Você está pronto para
avançar no SoPeD. Por responder os questionários você ganhou uma estrela!
<h2><img src='images/star.png' /> x 1</h2><br/><b style='color:
#428BCA;'>As estrelas liberam ferramentas no software que auxiliam no
cuidado dos seus pés. Quanto mais estrelas, melhor a saúde do seu
pé!</b></p>
                                                ";
                                        }
                                        $Obj->CloseDB();

                                ?>



                                <script type='text/javascript'>

                                        function SetGrade(num)
                                        {
        jQuery("#difficult").val(num);
                                                document.myform.submit();
                                        }

                                </script>
```

```
                                <form method="post" id="myform"
name="myform" action="index.php">

                                        <input type="hidden" name="page"
id="page" value="protocolo" />

                                        <input type="hidden"
name="position" id="position" value="<?php echo $position; ?>" />
                                        <input type="hidden"
name="difficult" id="difficult" value="" />
                                        <input type="hidden" name="id"
id="id" value="<?php echo $id; ?>" />

                                </form>


                                <script type='text/javascript'
src='js/pixi.min.js'></script>
                                <script type='text/javascript'
src='js/ruler.js'></script>
                                <link rel='stylesheet'
href='css/mytable.css' type='text/css' media='screen' />

                                <?php

                                        if($count != 0)
                                        {
                                                echo "<div
align='center'>
                                                        <canvas id='game'
width='360' height='640'></canvas>
                                                 </div>";
                                        }


                                        $sql = "SELECT block_id,
position, number, difficult,
DATE_FORMAT(registration_date,'%d/%m<br/>%H:%i') FROM saedd_protocol
WHERE user_id = '$user_id' ORDER BY 1,2";
                                        $Obj->ExecQuery($sql);

                                        $count = $Obj-
>Get("RecordCount");

                                        $row = 0;
                                        $col = 0;
                                        $lastRow = 0;
                                        $lastcol = 0;

                                        $qicon = "<img
src='images/exercicios/emotion_question.png' >";
                                        echo "<br/><br/><h4>Histórico de
Execução</h4><br/><table class='bordered'><thead><tr><th>Bloco</th>";

                                        for($i=1; $i<=$exCount; $i++)
                                          echo "<th>G$i</th>";

                                        echo "</tr></thead><tbody>";

                                        for($i=0; $i<$count; $i++)
                                        {

                                           $lastRow = $Obj-
>GetQueryValue($i, 0);
```

```
                                        echo "<tr>";
                                        echo "<td>Bloco
$lastRow</td>";

                                        while($i<$count)
                                        {
                                             $num = $Obj-
>GetQueryValue($i, 2);
                                             $dif = $Obj-
>GetQueryValue($i, 3);
                                             $row = $Obj-
>GetQueryValue($i, 0);
                                             $col = $Obj-
>GetQueryValue($i, 1);
                                             $dat = $Obj-
>GetQueryValue($i, 4);

                                             if($row == $lastRow)
                                               $i++;
                                             else
                                             {
                                               $i--;

for($k=intval($lastcol); $k<$exCount; $k++)
                                                 echo "<td>--</td>";
                                               break;

                                             }

                                             if($dif != "")
                                             {
                                                if($dif< 2.5)
                                                  $icon = "<img
src='images/exercicios/emotion_cool.png' >";

                                                else if($dif< 5.0)
                                                  $icon = "<img
src='images/exercicios/emotion_confuse.png' >";

                                                else if($dif< 7.5)
                                                  $icon = "<img
src='images/exercicios/emotion_sweat.png' >";

                                                else
                                                  $icon = "<img
src='images/exercicios/emotion_beaten.png' >";

                                                 echo "<td>Exerc.
$num<br/>$icon<br/>$dat</td>";
                                             }
                                             else
                                                 echo
"<td> <br/>$qicon</td>";

                                             $lastcol = $col;
                                          }

                                           echo "</tr>";
                                        }


                                     echo "</tbody></table>";


                                     /*
```

```
                                        $sql = "SELECT (SELECT block FROM
saedd_blocks WHERE saedd_blocks.id = block_id), position, number,
difficult FROM saedd_protocol WHERE difficult IS NOT NULL AND user_id =
'$user_id' ORDER BY 1,2";
                                        $Obj->ExecQuery($sql);

                                        $count = $Obj-
>Get("RecordCount");



                                                $row = "";
                                                $col = 0;
                                                $lastRow = 0;
                                                $lastcol = 0;


                                                $qicon = "<img
src='images/exercicios/emotion_question.png' >";
                                                echo
"<br/><br/><h4>Histórico de Execução</h4><br/><table
class='bordered'><thead><tr><th>Bloco</th>";

                                                for($i=1; $i<=$exCount;
$i++)
                                                  echo "<th>G$i</th>";

                                                echo
"</tr></thead><tbody>";

                                                for($i=0; $i<$count;
$i++)
                                                {
                                                   $row = $Obj-
>GetQueryValue($i, 0);
                                                   $col = $Obj-
>GetQueryValue($i, 1);
                                                   $num = $Obj-
>GetQueryValue($i, 2);
                                                   $dif = $Obj-
>GetQueryValue($i, 3);

                                                   if($lastRow != $row)
                                                   {
                                                      if($i != 0)
                                                      {
                                                        if($lastCol !=
$exCount)

for($j=$lastCol+1; $j<=$exCount; $j++)
                                                            echo
"<td> <br/>$qicon</td>";

                                                        echo
"</tr><tr><td><br/>$row</td>";
                                                      }
                                                      else
                                                        echo
"<tr><td><br/>$row</td>";
                                                   }


                                                   if($dif< 2.5)
                                                     $icon = "<img
src='images/exercicios/emotion_cool.png' >";
```

```
      else if($dif< 5.0)
        $icon = "<img
src='images/exercicios/emotion_confuse.png' >";
      else if($dif< 7.5)
        $icon = "<img
src='images/exercicios/emotion_sweat.png' >";
      else
        $icon = "<img
src='images/exercicios/emotion_beaten.png' >";


      for($j=1;
$j<=$exCount; $j++)
      {
           if($col == $j)
           {
              echo
"<td>Exerc. $num<br/>$icon</td>";
              break;
           }
           else if(($col-1)
!= $lastCol && $j > $lastCol)
           {
              echo
"<td> <br/>$qicon</td>";
           }

      }

      $lastRow = $row;
      $lastCol = $col;
   }

   if($row == "")
     echo
"<tr><td><br/>Bloco 1</td>";

   if($col != $exCount)
   {
      for($j=$col+1;
$j<=$exCount; $j++)
        echo
"<td> <br/>$qicon</td>";
   }


   echo
"</tr></tbody></table>";
   */

?>


<script type="text/javascript">
        jQuery(document).ready(function() {
                Preload();
        });
</script>
```

```
                        </div>
                </div>
        </div>
</div>

<div  class="content_row row vc_row wpb_row vc_row-fluid default-style
fullwidth" style="position:relative;overflow:hidden;">
        <div class="rt-parallax-background" data-rt-parallax-
direction="1" data-rt-parallax-effect="horizontal" style="background-
image: url(images/portfolio_header.jpg);background-repeat:
repeat;background-size: cover;background-position: right
top;width:100%;height:100%;top:0;"></div>
        <div class="content_row_wrapper  default" style="padding-
top:25px;padding-bottom:15px;">
                <div class="vc_col-sm-12 wpb_column vc_column_container"
>
                        <div class="wpb_wrapper">
                                <div class="rt_heading_wrapper style-4">
                                        <h1 class="rt_heading  style-4"
>Entre em contato!</h1>
                                </div>
                        </div>
                </div>
        </div>
</div>
<link rel='stylesheet' href='css/myform.css' type='text/css' media='all'
/>

<div  class="content_row row vc_row wpb_row vc_row-fluid default-style
fullwidth border_grid fixed_heights" >
        <div class="content_row_wrapper  default" >
                <div class="vc_col-sm-6 wpb_column vc_column_container" >
                        <div class="wpb_wrapper">
                                <div class="rt_heading_wrapper style-6">
                                        <h5 class="rt_heading  style-6"
>Informações para contato</h5>
                                </div>
                                <div  class="with_icons style-3 ">
                                        <div>
                                                <span class="icon-address
icon"></span>
                                                <div class="list-
content">Rua Cipotânea 51, Cidade Universitária, São Paulo - SP</div>
                                        </div>
                                        <div>
                                                <span class="icon-phone
icon"></span>
                                                <div class="list-
content">+55 (11) 3091-8426</div>
                                        </div>
                                        <div>
                                                <span class="icon-mail-1
icon"></span>
                                                <div class="list-
content">labimphfofito@gmail.com</div>
                                        </div>
                                </div>
                        </div>
                </div>
                <div class="vc_col-sm-6 wpb_column vc_column_container" >
                        <div class="wpb_wrapper">
                                <div class="rt_heading_wrapper style-6">
```

```
                                        <h5 class="rt_heading  style-6"
>Somos sociais!</h5>
                                </div>
                                <div  class="with_icons style-3 ">
                                        <div  >
                                                <span class="icon-
facebook icon"></span>
                                                <div class="list-
content"><a href="">Siga-nos!</a></div>
                                        </div>
                                        <div  >
                                                <span class="icon-
youtube-play icon"></span>
                                                <div class="list-
content"><a href="">Vídeos de exercícios</a></div>
                                        </div>
                                        <div  >
                                                <span class="icon-twitter
icon"></span>
                                                <div class="list-
content"><a href="">Siga-nos!</a></div>
                                        </div>
                                </div>
                        </div>
                </div>
        </div>
</div>


<div  class="content_row row vc_row wpb_row vc_row-fluid alt-style-1
fullwidth" >
        <div class="content_row_wrapper  default" >
                <div class="vc_col-sm-12 wpb_column vc_column_container"
>
                        <div class="wpb_wrapper">
                                <div class="rt_heading_wrapper style-6">
                                        <h5 class="rt_heading  style-6"
>Preencha o formulário abaixo</h5>
                                </div>
                                 Agradecemos o seu interesse no projeto!
Escreva-nos e entraremos em contato o mais breve possível.

                                <br/><br/>
                                <div class="clear"></div>


                                        <form action="#"
name="contact_form" class="validate_form rt_form" method="post"
id="myform" style="margin-left: -15px;">

                                                <div class="vc_col-sm-3
wpb_column vc_column_container" >
                                                        <label>Nome
completo</label>
                                                        <input
type="text" name="nome" />
                                                </div>

                                                <div class="vc_col-sm-3
wpb_column vc_column_container" >
                                                        <label>E-
mail</label>
                                                        <input
type="email" name="nome" />
                                                </div>
```

```
                                                <div class="vc_col-sm-3
wpb_column vc_column_container" >

        <label>Telefone</label>
                                                        <input
type="text" name="telefone" />
                                                </div>

                                                <div class="vc_col-sm-3
wpb_column vc_column_container" >

        <label>Assunto</label>
                                                        <input
type="text" name="telefone" />
                                                </div>

                                                <div
class="clear"></div><br/>

                                                <div class="vc_col-sm-12
wpb_column vc_column_container" >

        <label>Mensagem</label>
                                                        <textarea
style="height: 150px;"></textarea>
                                                </div>
                                        </form>

                                        <div
class="clear"></div><br/><br/>
                                        <div class="submit-button">
                                                <input class="button
submit" value="Enviar Mensagem" type="button" style="background:
#428bca;" ><span class="loading"></span>
                                        </div>

                                <div class="clear"></div>

                        </div>
                </div>
        </div>
</div>




<div  class="content_row row vc_row wpb_row vc_row-fluid default-style
fullwidth" >
        <div class="content_row_wrapper  default" >
                <div class="vc_col-sm-12 wpb_column vc_column_container"
>
                        <div class="wpb_wrapper">

                        <h2>Encontre-nos no mapa</h2><br/><br/>

        <img src="files/pages/como_Chegar_03.png" /><br/><br/>

    &bull; Siga pela Av.Corifeu de Azevedo Marques para altura do No
3100.<br/>
    &bull; Entrar pela Portaria 3 da USP.<br/>
    &bull; Virar a esquerda na primeira rotatória.<br/>
    &bull; A Rua Cipotânea fica próxima ao ponto de
ônibus.<br/><br/><br/>
```

```
<iframe src="http://maps.google.com.br/maps?hl=pt-
BR&amp;q=rua+cipot%C3%A2nea+51&amp;ie=UTF8&amp;hq=&amp;hnear=R.+Cipot%C3%
A2nea,+51+-+Butant%C3%A3,+S%C3%A3o+Paulo,+05360-160&amp;ll=-23.565566,-
46.741477&amp;spn=0.010857,0.013518&amp;t=m&amp;z=14&amp;vpsrc=6&amp;outp
ut=embed" width="550" height="350" frameborder="0" marginwidth="0"
marginheight="0" scrolling="no">  </iframe>


                        </div>
                </div>
        </div>
</div>



                                <div  class="content_row row vc_row
wpb_row vc_row-fluid default-style fullwidth" >
        <br/><br/>
        <div class="content_row_wrapper nopadding fullwidth" >
        <div class="vc_col-sm-12 wpb_column vc_column_container" >
                <div class="wpb_wrapper">


                </div>
                </div>
        </div>
</div>
<br/><br/>

</div>

<link rel='stylesheet' href='css/myform.css' type='text/css' media='all'
/>

<style type="text/css">
.myform input[type="text"],
.myform input[type="date"],
.myform input[type="datetime"],
.myform input[type="email"],
.myform input[type="number"],
.myform input[type="search"],
.myform input[type="time"],
.myform input[type="url"],
.myform input[type="password"],
.myform textarea,
.myform select {

    border-top: 1px solid #f0f0f0;
    border-left: 1px solid #f0f0f0;
    border-bottom: 1px solid #FAFAFA;
    border-right: 1px solid #FAFAFA;
    background: #e0e0e0;
}

.default-style input[type="button"]:hover {background-color: #FC9D0D;}
.default-style input[type="button"] {background-color: #428bca; font-
weight: bold;}

.default-style input[type="button"]:hover.extra  {background-color:
#BBB;}
.default-style input[type="button"].extra  {background-color: #888; font-
weight: bold;}
</style>
```

```
<?php include("php/pages/internal.php"); ?>



<div  class="content_row default-style no-composer overlap fullwidth">
        <div class="content_row_wrapper clearfix ">
                <div class="col col-sm-12 col-xs-12 ">
                        <div class="single post-1872 staff type-staff
status-publish has-post-thumbnail hentry" id="person-1872">


                        <h2>Quão difícil foi executar esse
exercício?</h2><br/>
                        <label for="aval1" style='margin-right:
50px;'><img src="images/exercicios/emotion_cool.png" /><br/><input
type="radio" name="avaliacao" value="5" id="aval1" /> Muito fácil</label>
                        <label for="aval2" style='margin-right:
50px;'><img src="images/exercicios/emotion_smile.png" /><br/><input
type="radio" name="avaliacao" value="4" id="aval2" /> Fácil</label>
                        <label for="aval3" style='margin-right:
50px;'><img src="images/exercicios/emotion_amazing.png" /><br/><input
type="radio" name="avaliacao" value="3" id="aval3" /> Razoável</label>
                        <label for="aval4" style='margin-right:
50px;'><img src="images/exercicios/emotion_sweat.png" /><br/><input
type="radio" name="avaliacao" value="2" id="aval4" /> Difícil</label>
                        <label for="aval5" style='margin-right:
50px;'><img src="images/exercicios/emotion_beaten.png" /><br/><input
type="radio" name="avaliacao" value="1" id="aval5" /> Muito
Difícil</label>
                        <br/><br/>


                        <label>Precisa de ajuda? Envie um comentário para
os especialistas</label>
                        <textarea style='width: 100%; height: 100px;
background: #efefef;' placeholder="Digite a sua mensagem
(Opcional)"></textarea>

                        <br/><br/><input class="button submit"
onclick="javascript:location.href = 'index.php?page=program';"
value="Continuar..." type="button">

                        </div>
                </div>

                <div class="col col-sm-4 col-xs-12 ">
                        <div class="single post-1872 staff type-staff
status-publish has-post-thumbnail hentry" id="person-1872">


                        </div>
                </div>

        </div>
</div>
```

```
<div  class="content_row row vc_row wpb_row vc_row-fluid default-style
fullwidth" style="position:relative;overflow:hidden;">
        <div class="rt-parallax-background" data-rt-parallax-
direction="1" data-rt-parallax-effect="horizontal" style="background-
image: url(images/portfolio_header.jpg);background-repeat:
repeat;background-size: cover;background-position: right
top;width:100%;height:100%;top:0;"></div>
        <div class="content_row_wrapper  default" style="padding-
top:25px;padding-bottom:15px;">
                <div class="vc_col-sm-12 wpb_column vc_column_container"
>
                        <div class="wpb_wrapper">
                                <div class="rt_heading_wrapper style-4">
                                        <h1 class="rt_heading  style-4"
>Questionário para avaliação dos especialistas</h1>
                                </div>
                        </div>
                </div>
        </div>
</div>


<style>
        label{font-weight: normal;}
</style>



<style type="text/css">


.alt-style-1 input[type="button"]:hover {background-color: #FC9D0D;}
.alt-style-1 input[type="button"] {background-color: #428bca; font-
weight: bold;}

.alt-style-1 input[type="button"]:hover.extra  {background-color: #BBB;}
.alt-style-1 input[type="button"].extra  {background-color: #888; font-
weight: bold;}

b span {color: #428BCA;}


</style>

<div  class="content_row default-style no-composer overlap fullwidth">
        <div class="content_row_wrapper clearfix ">
                <div class="col col-sm-12 col-xs-12 ">
                        <div class="single post-1872 staff type-staff
status-publish has-post-thumbnail hentry" id="person-1872">




                                <br/><h2>Formulário de
avaliação</h2><br/>



                                <p>Considere sua avaliação para as duas
ferramentas disponíveis: <b>Software e Aplicativo Móvel</b>
                                <br/>Caso julgue que há alguma diferença
entre os dois, por favor comente nos espaços indicados.</p><br/><br/>
```

```
                </div>
            </div>


        </div>
</div>

<div  class="content_row alt-style-1 no-composer overlap fullwidth">
        <div class="content_row_wrapper clearfix " style='min-height:
800px;'>
                <div class="col col-sm-12 col-xs-12 ">
                        <div class="single post-1872 staff type-staff
status-publish has-post-thumbnail hentry" id="person-1872">



                                <form id='questions' name='questions'
method="post" action='index.php'>
                                <?php
                                        $perguntas = array("",
                                        "O objetivo da pesquisa
(Elaborar, desenvolver e validar o conteúdo de um software para
diabéticos que individualiza a evolução de exercícios para pés e
tornozelos e podem ser realizados em qualquer ambiente à escolha do
indivíduo) foi contemplado.",
                                        "A linguagem, que compõe o
conteúdo do software, é adequada para a população com diabetes
mellitus.",
                                        "A navegação pelo software e/ou
aplicativo está claro, intuitivo e fácil de navegar.",
                                        "O software tem um componente
motivacional e convidativo, o que facilita a sua adesão. Estimulando a
prática cotidiana dos exercícios propostos.",
                                        "O material deixa claro questões
que situem o usuário para que serve o material e a quem se destina. Como
por exemplo, questões como : sobre o que é o software; Por que quem tem
diabetes deve realizar exercícios para os pés; e O que é o pé
diabético.",
                                        "O material fornece informações
essenciais para que o usuário se conscientize da importância da
realização dos exercícios disponíveis no software.",
                                        "O material disponível é
suficiente para que o usuário realize os exercícios sem o auxilio de um
profissional.",
                                        "Em caso de dúvidas na realização
dos exercícios ou nas avaliações contidas no software, o usuário tem
suporte de esclarecimento disponível.",
                                        "Os exercícios contidos no
Software contribuem para diminuição dos déficits causados pela neuropatia
diabética, com relação à função dos pés.",
                                        "O software realmente dispõe de
um treinamento personalizado.",
                                        "O software dispõe de recursos
que dão suporte nas explicações de como utilizar o software, preencher
avaliações e realizar os exercícios.",
                                        "O software dá um feedback
personalizado, assim que o usuário responde as questões disponíveis no
software, como o MNSI e o questionário de saúde dos pés.",
                                        "O software permite que o usuário
expresse sua opinião acerca de um exercício que o mesmo tenha dito
dificuldades em realizar. Ou qualquer outra colocação do usuário.",
                                        "O software se preocupa com
questões de acessibilidade, permitindo ao usuário que tenha dificuldades
```

```
para ler (devidos a problemas visuais) que o mesmo escute o passo a
passo, facilitando à sua compreensão.",
                                        "O software é capaz de enviar um
aviso ao indivíduo caso o mesmo deixe de realizar os exercícios, como o
previsto.",
                                        "O software dispõe de informações
sobre o pé diabético, dando dicas de cuidados aos usuários, e eventos
relacionados.",
                                        "O software pode ser adotado
pelos serviços de saúde (público e privado), bem como serve de material
de apoio para o profissional fisioterapeuta que cuida do pé diabético bem
como qualquer outro profissional que cuida da atenção primária à saúde.",
                                        "O software deixa claro aos
usuários que as avaliações feitas pelos mesmos poderão ser utilizadas
como banco de dados em pesquisas desenvolvidas no Laboratório de
Biomecânica da USP - LABIMPH na Faculdade de Medicina da USP.",
                                        "Existem situações, como por
exemplo, uma ulceração plantar ativa, em que o usuário é impossibilitado
de realizar os exercícios propostos pelo software. Nestes casos, após
responder ao questionário de avaliação, o usuário é avisado a não
realizar os exercícios e informados os procedimentos que deve realizar.",
                                        "Os itens que compõem o software,
como cor, linguagem e disposição de imagens estão adequados ao público a
que se destina.",
                                        "As avaliações feitas no software
são suficientes para quantificar até que ponto o usuário é permitido
realizar os exercícios propostos."
                                        );



                                        for($i=1; $i<count($perguntas);
$i++)
                                        {
                                           echo "<b>Pergunta $i:
".$perguntas[$i]."</b><br/>
                                                        <label
for='quest_".$i."_r1' style='margin-right: 20px;'><input type='radio'
value='1' name='quest_$i' id='quest_".$i."_r1' /> Concordo
plenamente</label>
                                                        <label
for='quest_".$i."_r2' style='margin-right: 20px;'><input type='radio'
value='0' name='quest_$i' id='quest_".$i."_r2' /> Concordo</label>
                                                        <label
for='quest_".$i."_r3' style='margin-right: 20px;'><input type='radio'
value='0' name='quest_$i' id='quest_".$i."_r3' /> Não concordo e nem
discordo</label>
                                                        <label
for='quest_".$i."_r4' style='margin-right: 20px;'><input type='radio'
value='0' name='quest_$i' id='quest_".$i."_r4' /> Discordo</label>
                                                        <label
for='quest_".$i."_r5' ><input type='radio' value='0' name='quest_$i'
id='quest_".$i."_r5' /> Discordo plenamente</label>
                                                <textarea
placeholder='Justifique sua resposta' style='background: #FFF; width:
100%; height: 100px;'></textarea><br/><br/><br/>";
                                        }


                                        /*
                                        for($i=1; $i<count($perguntas);
$i++)
                                        {
```

```
                                                echo "<b>Pergunta $i:
".$perguntas[$i]."</b><br/><div class='vc_col-sm-4 wpb_column
vc_column_container' ><div class='wpb_wrapper'>
                                                             <label
for='quest_".$i."_r1'><input type='radio' value='1' name='quest_$i'
id='quest_".$i."_r1' /> Concordo plenamente</label><br/>
                                                             <label
for='quest_".$i."_r2'><input type='radio' value='0' name='quest_$i'
id='quest_".$i."_r2' /> Concordo</label><br/>
                                                             <label
for='quest_".$i."_r3'><input type='radio' value='0' name='quest_$i'
id='quest_".$i."_r3' /> Não concordo e nem discordo</label><br/>
                                                             <label
for='quest_".$i."_r4'><input type='radio' value='0' name='quest_$i'
id='quest_".$i."_r4' /> Discordo</label><br/>
                                                             <label
for='quest_".$i."_r5'><input type='radio' value='0' name='quest_$i'
id='quest_".$i."_r5' /> Discordo plenamente</label><br/><br/><br/>
                                                     </div></div><div
class='vc_col-sm-4 wpb_column vc_column_container' ><div
class='wpb_wrapper'><textarea placeholder='Justifique sua
resposta'></textarea></div></div><div class='clear'></div>";
                                          }
                                          */
                                ?>

                                <div class="clear"></div>
                                <div id='msg' style='color:
#CC2233;'></div><br/>

                                <input type='hidden' value='0'
id='points' name='points' />

                                </form>

                                <input class="button submit"
onclick="javascript:window.location = 'index.php?page=questionarios';"
value="Enviar Respostas" type="button" />


                        </div>
                </div>


        </div>
</div>




<div  class="content_row row vc_row wpb_row vc_row-fluid default-style
fullwidth" style="position:relative;overflow:hidden;">
        <div class="rt-parallax-background" data-rt-parallax-
direction="1" data-rt-parallax-effect="horizontal" style="background-
image: url(images/portfolio_header.jpg);background-repeat:
repeat;background-size: cover;background-position: right
top;width:100%;height:100%;top:0;"></div>
        <div class="content_row_wrapper  default" style="padding-
top:25px;padding-bottom:15px;">
                <div class="vc_col-sm-12 wpb_column vc_column_container"
>
                        <div class="wpb_wrapper">
                                <div class="rt_heading_wrapper style-4">
                                        <h1 class="rt_heading  style-4"
>Publicações</h1>
```

```
                                </div>
                        </div>
                </div>
        </div>
</div>


<div  class="content_row row vc_row wpb_row vc_row-fluid default-style
fullwidth" >
        <div class="content_row_wrapper  default" style="padding-
top:40px;padding-bottom:0px;">
                <div class="vc_col-sm-8 wpb_column vc_column_container" >
                        <div class="wpb_wrapper" style='min-height:
714px;'>

                        <p><b>Os estudos contidos nas publicações abaixo
serviram de base para o desenvolvimento dessa ferramenta:</b></p>
<div  class="with_icons style-3 ">

                <div>
                <span class="icon-ok icon"></span><div class="list-
content">Effects of strengthening, stretching and functional training on
foot function in patients with diabetic neuropathy: results of a
randomized controlled trial.</div>

                </div>

                <div>
                        <span class="icon-eye icon"></span><div
class="list-content"><a href="http://www.biomedcentral.com/1471-
2474/15/137" target="_blank">Clique aqui</a> para visualizar.</div>
                </div>


                <hr/>
                <br/>

                <div>
                <span class="icon-ok icon"></span><div class="list-
content">Effects of a combined strengthening, stretching and functional
training program versus usual-care on gait biomechanics and foot function
for diabetic neuropathy: a randomized controlled trial.</div>
                </div>


                <div>
                        <span class="icon-eye icon"></span><div
class="list-content"><a href="http://www.biomedcentral.com/1471-
2474/13/36" target="_blank">Clique aqui</a> para visualizar.</div>
                </div>

                <hr/>
                <br/>


                <div>
                <span class="icon-ok icon"></span><div class="list-
content">From treatment to preventive actions: improving function in
patients with diabetic polyneuropathy.</div>
                </div>

                <div>
                        <span class="icon-eye icon"></span><div
class="list-content"><a
```

```
href="http://www.ncbi.nlm.nih.gov/pubmed/26452065" target="_blank">Clique
aqui</a> para visualizar.</div>
                </div>


                <hr/>
                <br/>

                <div>
                <span class="icon-ok icon"></span><div class="list-
content">Influência da intervenção cinesioterapêutica em tornozelo e pé
na biomecânica da marcha de diabéticos neuropatas: um ensaio clínico
randomizado.</div>
                </div>
                <div>
                        <span class="icon-eye icon"></span><div
class="list-content"><a
href="http://www.teses.usp.br/teses/disponiveis/5/5170/tde-01082013-
145303/en.php" target="_blank">Clique aqui</a> para visualizar.</div>
                </div>


                <hr/>
                <br/>

                </div>

                        </div>
                </div>
        </div>
</div>

<div  class="content_row row vc_row wpb_row vc_row-fluid default-style
fullwidth" style="position:relative;overflow:hidden;">
        <div class="rt-parallax-background" data-rt-parallax-
direction="1" data-rt-parallax-effect="horizontal" style="background-
image: url(images/portfolio_header.jpg);background-repeat:
repeat;background-size: cover;background-position: right
top;width:100%;height:100%;top:0;"></div>
        <div class="content_row_wrapper  default" style="padding-
top:25px;padding-bottom:15px;">
                <div class="vc_col-sm-12 wpb_column vc_column_container"
>
                        <div class="wpb_wrapper">
                                <div class="rt_heading_wrapper style-4">
                                        <h1 class="rt_heading  style-4"
>Área interna do SAEDD</h1><br/>
                                </div>
                        </div>
                </div>
        </div>
</div>




<link rel='stylesheet' href='css/myform.css' type='text/css' media='all'
/>

<style type="text/css">
.myform input[type="text"],
.myform input[type="date"],
.myform input[type="datetime"],
.myform input[type="email"],
```

```
.myform input[type="number"],
.myform input[type="search"],
.myform input[type="time"],
.myform input[type="url"],
.myform input[type="password"],
.myform textarea,
.myform select {

    border-top: 1px solid #f0f0f0;
    border-left: 1px solid #f0f0f0;
    border-bottom: 1px solid #FAFAFA;
    border-right: 1px solid #FAFAFA;
    background: #e0e0e0;
}

.alt-style-1 input[type="button"]:hover {background-color: #FC9D0D;}
.alt-style-1 input[type="button"] {background-color: #428bca; font-
weight: bold;}

.alt-style-1 input[type="button"]:hover.extra  {background-color: #BBB;}
.alt-style-1 input[type="button"].extra  {background-color: #888; font-
weight: bold;}
</style>



<?php include("php/pages/internal.php"); ?>




<div  class="content_row alt-style-1 no-composer overlap fullwidth">
        <div class="content_row_wrapper clearfix ">
                <div class="col col-sm-12 col-xs-12 ">
                        <div class="single post-1872 staff type-staff
status-publish has-post-thumbnail hentry" id="person-1872">

                                <h2>Obrigado por responder o
questionário!
                                <br/><br/>Você ganhou <b style='color:
#428BCA;'>29 pontos</b>!</h2><br/><br/>

                                <b>Os <span style='color:
#428BCA;'>pontos liberam as ferramentas e exercícios</b> que irão
auxiliar o bem estar dos seus pés!</b>

                                <div class="clear"></div><br/>
                                <input class="button submit"
onclick="javascript:location.href = 'index.php?page=ocorrencias';"
value="Gostei, Quero continuar..." type="button">
                        </div>
                </div>


        </div>
</div>







<div  class="content_row row vc_row wpb_row vc_row-fluid default-style
fullwidth border_grid fixed_heights" >
```

```
        <div class="content_row_wrapper  default" style="padding-
top:40px;padding-bottom:40px; margin-bottom: 40px;">



                <div class="vc_col-sm-3 wpb_column vc_column_container
vc_custom_1421091415717" >
                        <div class="wpb_wrapper">
                                <article  class="icon-content-box
content-box icon-top icon-style-3">
                                        <div class="icon-holder"><a
href="index.php?page=user_auto01" title="" target="_self"><span
class="icon-gauge-1"></span></a></div>
                                        <div class="text-holder">
                                                <h4 class="heading">
                                                        <a
href="index.php?page=user_auto01" title="Box Heading" target="_self">
                                                        Auto-avaliação
                                                        </a>
                                                </h4>
                                                <p
class="aligncenter"><br/>Faça uma avaliação para receber exercícios
personalizados.</p>
                                        </div>
                                </article>
                        </div>
                </div>

                <div class="vc_col-sm-3 wpb_column vc_column_container" >
                        <div class="wpb_wrapper">
                                <article  class="icon-content-box
content-box icon-top icon-style-3">
                                        <div class="icon-holder"><a
href="index.php?page=ocorrencias" title="" target="_self"><span
class="icon-new-photo-1"></span></a></div>
                                        <div class="text-holder">
                                                <h4 class="heading">
                                                        <a
href="index.php?page=ocorrencias" title="Box Heading" target="_self">
                                                        Fotos e
Ocorrências

                                                        </a>
                                                </h4>
                                                <p
class="aligncenter"><br/>Use nosso modelo 3d para indicar ocorrências e
enviar fotos.</p>
                                        </div>
                                </article>
                        </div>
                </div>

                <div class="vc_col-sm-3 wpb_column vc_column_container" >
                        <div class="wpb_wrapper">
                                <article  class="icon-content-box
content-box icon-top icon-style-3">
                                        <div class="icon-holder"><a
href="index.php?page=program" title="" target="_self"><span class="icon-
bell"></span></a></div>
                                        <div class="text-holder">
                                                <h4 class="heading">
                                                        <a
href="index.php?page=program" title="Box Heading" target="_self">
                                                        Exercícios
                                                        </a>
                                                </h4>
```

```
                                                <p
class="aligncenter"><br/>Inicie a execução dos seus exercícios.</p>
                                        </div>
                                </article>
                        </div>
                </div>


                <div class="vc_col-sm-3 wpb_column vc_column_container" >
                        <div class="wpb_wrapper">
                                <article  class="icon-content-box
content-box icon-top icon-style-3">
                                        <div class="icon-holder"><a
href="#" title="" target="_self"><span class="icon-new-heart-
1"></span></a></div>
                                        <div class="text-holder">
                                                <h4 class="heading">
                                                        <a href="#"
title="Box Heading" target="_self">
                                                        Mostre sua
evolução!
                                                        </a>
                                                </h4>
                                                <p
class="aligncenter"><br/>Compartilhe sua pontuação em sua rede social
preferida!</p>
                                        </div>
                                </article>
                        </div>
                </div>

        </div>
</div>







<div  class="content_row row vc_row wpb_row vc_row-fluid default-style
fullwidth" style="position:relative;overflow:hidden;">
        <div class="rt-parallax-background" data-rt-parallax-
direction="1" data-rt-parallax-effect="horizontal" style="background-
image: url(images/portfolio_header.jpg);background-repeat:
repeat;background-size: cover;background-position: right
top;width:100%;height:100%;top:0;"></div>
        <div class="content_row_wrapper  default" style="padding-
top:25px;padding-bottom:15px;">
                <div class="vc_col-sm-12 wpb_column vc_column_container"
>
                        <div class="wpb_wrapper">
                                <div class="rt_heading_wrapper style-4">
                                        <h1 class="rt_heading  style-4"
>Área interna do SAEDD</h1><br/>
                                </div>
                        </div>
                </div>
        </div>
</div>
```

```
<link rel='stylesheet' href='css/myform.css' type='text/css' media='all'
/>

<style type="text/css">
#myform input[type="text"],
#myform input[type="date"],
#myform input[type="datetime"],
#myform input[type="email"],
#myform input[type="number"],
#myform input[type="search"],
#myform input[type="time"],
#myform input[type="url"],
#myform input[type="password"],
#myform textarea,
#myform select {

    border-top: 1px solid #f0f0f0;
    border-left: 1px solid #f0f0f0;
    border-bottom: 1px solid #FAFAFA;
    border-right: 1px solid #FAFAFA;
    background: #e0e0e0;
}

.alt-style-1 input[type="button"]:hover {background-color: #FC9D0D;}
.alt-style-1 input[type="button"] {background-color: #428bca; font-
weight: bold;}

.alt-style-1 input[type="button"]:hover.extra  {background-color: #BBB;}
.alt-style-1 input[type="button"].extra  {background-color: #888; font-
weight: bold;}


</style>


        <?php include("php/pages/internal.php"); ?>



<div  class="content_row alt-style-1 no-composer overlap fullwidth">
        <div class="content_row_wrapper clearfix ">
                <div class="col col-sm-12 col-xs-12 ">
                        <div class="single post-1872 staff type-staff
status-publish has-post-thumbnail hentry" id="person-1872">
                                <div class="entry-thumbnail alignleft">
                                        <img src="images/face-
luciano.jpg" alt="" class="" />
                                        <div
class="person_links_wrapper">
                                                <h5>Nível de aptidão:
</h5> <img src="images/star.png" /><img src="images/star.png" /><img
src="images/star.png" /><img src="images/star.png" /><img
src="images/star.png" />
                                        </div>
                                </div>
                                <div id="idTextPanel">
                                        <h4><strong>Luciano Campelo, seja
bem vindo ao SAEDD!</strong></h4>
                                        <br/><p><strong>Seu último
acesso:</strong> 14/05/2016</p>
                                        <p><strong>Pontos essa
semana:</strong> 376 (Média dos usuários: 141)</p>
```

```
                                        <p><strong>Pontos esse
mês:</strong> 976 (Média dos usuários: 421)</p>


                                        <br/><h5>Lembretes:</h5><br/>
                                        <p><strong>Próximo
Exercício:</strong> Vídeo 19 / Repetição 3 <b style='color:
#FF2233;'>(Atrasado 2 dias)</b></p>
                                        <p><strong>Próxima
avaliação:</strong> 13/06/2016 <b style='color: #229933;'>(Falta 21
dias)</b></p>



                                </div>
                        </div>
                </div>


        </div>
</div>





<div  class="content_row row vc_row wpb_row vc_row-fluid default-style
fullwidth border-top border-bottom" >
        <div class="content_row_wrapper  default" style="padding-
top:60px;padding-bottom:60px;">
                <div class="vc_col-sm-12 wpb_column vc_column_container"
>
                        <div class="wpb_wrapper">
                                <div class="rt_heading_wrapper style-4">
                                        <span class="punchline">Linha do
Tempo</span>
                                        <h4 class="rt_heading
with_punchline style-4" >Minha história no SAEDD</h4>
                                </div>
                                <section  class="timeline  ">
                                        <div  >
                                                <span class="event-date">
                                                <span
class="day">16</span>
                                                <span class="month">de
Maio</span>
                                                <span
class="year">2016</span>
                                                </span>
                                                <div class="event-
details">
                                                        <p>Avancei para o
nível 5. Fui o 2º melhor usuário avaliada na semana.</p>
                                                </div>
                                        </div>
                                        <div  >
                                                <span class="event-date">
                                                <span
class="day">30</span>
                                                <span class="month">de
março</span>
                                                <span
class="year">2015</span>
                                                </span>
```

```
                                                <div class="event-
details">
                                                        <p>Cheguei ao
nível intermediário e fui classificado como o melhor usuário da
semana.</p>
                                                </div>
                                        </div>
                                        <div  >
                                                <span class="event-date">
                                                <span
class="day">10</span>
                                                <span class="month">de
Fevereiro</span>
                                                <span
class="year">2015</span>
                                                </span>
                                                <div class="event-
details">
                                                        <p>Avancei meu
primeiro nível.</p>
                                                </div>
                                        </div>
                                        <div  >
                                                <span class="event-date">
                                                <span
class="day">18</span>
                                                <span class="month">de
Janeiro</span>
                                                <span
class="year">2015</span>
                                                </span>
                                                <div class="event-
details">
                                                        <p>Respondi minha
primeira avaliação.</p>
                                                </div>
                                        </div>
                                        <div  >
                                                <span class="event-date">
                                                <span
class="day">17</span>
                                                <span class="month">de
Janeiro</span>
                                                <span
class="year">2015</span>
                                                </span>
                                                <div class="event-
details">
                                                        <p>Fiz meu
cadastro no SAEDD.</p>
                                                </div>
                                        </div>
                                </section>
                        </div>
                </div>
        </div>
</div>


<div  class="content_row row vc_row wpb_row vc_row-fluid default-style
fullwidth" style="position:relative;overflow:hidden;">
        <div class="rt-parallax-background" data-rt-parallax-
direction="1" data-rt-parallax-effect="horizontal" style="background-
image: url(images/portfolio_header.jpg);background-repeat:
```

```
repeat;background-size: cover;background-position: right
top;width:100%;height:100%;top:0;"></div>
        <div class="content_row_wrapper  default" style="padding-
top:25px;padding-bottom:15px;">
                <div class="vc_col-sm-12 wpb_column vc_column_container"
>
                        <div class="wpb_wrapper">
                                <div class="rt_heading_wrapper style-4">
                                        <h1 class="rt_heading  style-4"
>Protocolo de exercícios</h1>
                                </div>
                        </div>
                </div>
        </div>
</div>

<div  class="content_row row vc_row wpb_row vc_row-fluid alt-style-1
fullwidth border_grid fixed_heights" >
        <div class="content_row_wrapper  default" style="padding-
top:50px;padding-bottom:50px; min-height: 500px;">
                <div class="vc_col-sm-12 wpb_column vc_column_container"
>
                        <div class="wpb_wrapper">
                                <div class="rt_heading_wrapper style-6">
                                        <h4 class="rt_heading  style-6"
><span class="icon-glyph-15 heading_icon"></span>Lista de exercícios</h4>
                                </div>
                                <div class="wpb_text_column
wpb_content_element ">
                                        <div class="wpb_wrapper">
                                                <p>Listagem dos
exercícios e dos blocos dos protocolos.</b></p>
                                        </div>
                                </div>
                                <link rel='stylesheet'
href='css/mytable.css' type='text/css' media='all' />

<?php
                        require_once('php/MysqlDB.php');
                        $id = $_SESSION['id'];

                        $Obj = new ScriptDB();
                        $Obj->Connect();
                        $Obj->ExecQuery("SELECT * from saedd_exercises");

                        echo $Obj->QueryToTable(" class='bordered' ");

                        echo "<br/><br/>";
                        $Obj->ExecQuery("SELECT * from saedd_blocks");

                        echo $Obj->QueryToTable(" class='bordered' ");


?>






                        </div>
                </div>
        </div>
</div>
```
