# Supplementary material for: Rehabilitation technology for self-care: Customised foot and ankle exercise software for people with diabetes
Source: PLoS One. 2019 Jun 20;14(6):e0218560. doi: 10.1371/journal.pone.0218560 (PMC6586406; doi:10.1371/journal.pone.0218560)
Supplement: S2 Appendix — (PDF) [file pone.0218560.s002.pdf]

- **forum**
  - **bin**
    - phpbbcli.php
  - **phpbb**
    - **filesystem**
      - filesystem_interface.php
      - **exception**
        - filesystem_exception.php
      - filesystem.php
    - user_loader.php
    - **install**
      - task_interface.php
      - **exception**
        - invalid_dbms_exception.php
        - jump_to_restart_point_exception.php
        - resource_limit_reached_exception.php
        - cannot_build_container_exception.php
        - user_interaction_required_exception.php
        - installer_config_not_writable_exception.php
        - file_updater_failure_exception.php
        - installer_exception.php
      - **controller**
        - install.php
        - helper.php
        - installer_index.php
        - archive_download.php
        - timeout_check.php
        - update.php
      - updater_configuration.php
      - module_base.php
      - task_base.php
      - **event**
        - kernel_exception_subscriber.php
      - **helper**
        - container_factory.php
        - **iohandler**
          - iohandler_interface.php
          - **exception**
            - iohandler_not_implemented_exception.php
          - factory.php
          - cli_iohandler.php
          - ajax_iohandler.php
          - iohandler_base.php
        - database.php
        - **file_updater**

        - file_updater_interface.php
        - file_updater.php
        - ftp_file_updater.php
        - compression_file_updater.php
        - factory.php
    - config.php
    - install_helper.php
    - **navigation**
        - navigation_provider.php
        - navigation_interface.php
        - convertor_navigation.php
        - update_navigation.php
        - install_navigation.php
        - main_navigation.php
    - update_helper.php
- installer.php
- module_interface.php
- **module**
    - **install_database**
        - module.php
        - **task**
            - add_default_data.php
            - set_up_database.php
            - create_schema_file.php
            - create_schema.php
            - add_tables.php
            - add_config_settings.php
    - **install_data**
        - module.php
        - **task**
            - add_modules.php
            - add_bots.php
            - create_search_index.php
            - add_languages.php
    - **requirements**
        - abstract_requirements_module.php
        - install_module.php
        - **task**
            - check_server_environment.php
            - check_update.php
            - check_filesystem.php
        - update_module.php
    - **install_finish**
        - module.php
        - **task**

            install_extensions.php
            populate_migrations.php
            notify_user.php
    update_filesystem
        module.php
        task
            file_check.php
            update_files.php
            show_file_status.php
            diff_files.php
            download_updated_files.php
    obtain_data
        install_module.php
        task
            obtain_database_data.php
            obtain_admin_data.php
            obtain_update_ftp_data.php
            obtain_board_data.php
            obtain_server_data.php
            obtain_update_files.php
            obtain_update_settings.php
            obtain_email_data.php
            obtain_imagick_path.php
            obtain_file_updater_method.php
        update_module.php
    install_filesystem
        module.php
        task
            create_config_file.php
    update_database
        module.php
        task
            update_extensions.php
            update.php
installer_configuration.php
console
    command
        install
            install.php
            config
                validate.php
                show.php
        update
            config
                validate.php

show.php
update.php
textreparser
base.php
row_based_plugin.php
reparser_interface.php
plugins
pm_text.php
post_text.php
group_description.php
forum_description.php
user_signature.php
contact_admin_info.php
poll_title.php
forum_rules.php
poll_option.php
manager.php
group
helper.php
datetime.php
tree
tree_interface.php
nestedset_forum.php
nestedset.php
passwords
helper.php
driver
rehashable_driver_interface.php
base.php
helper.php
driver_interface.php
bcrypt_wcf2.php
phpass.php
md5_vb.php
md5_phpbb2.php
sha1.php
sha1_smf.php
md5_mybb.php
bcrypt.php
convert_password.php
salted_md5.php
sha1_wcf1.php
sha_xf1.php
bcrypt_2y.php
manager.php

- **help**
  - **controller**
    - bbcode.php
    - help.php
    - controller.php
    - faq.php
  - manager.php
- **groupposition**
  - exception.php
  - groupposition_interface.php
  - legend.php
  - teampage.php
- viewonline_helper.php
- **exception**
  - version_check_exception.php
  - exception_interface.php
  - runtime_exception.php
  - http_exception.php
- **controller**
  - exception.php
  - helper.php
  - resolver.php
- permissions.php
- pagination.php
- **debug**
  - error_handler.php
  - debug.php
- **plupload**
  - plupload.php
- **config**
  - db_text.php
  - config.php
  - db.php
- content_visibility.php
- session.php
- finder.php
- **hook**
  - finder.php
- recursive_dot_prefix_filter_iterator.php
- config_php_file.php
- **event**
  - kernel_exception_subscriber.php
  - kernel_terminate_subscriber.php
  - recursive_event_filter_iterator.php
  - php_exporter.php

data.php
md_exporter.php
dispatcher.php
dispatcher_interface.php
attachment
resync.php
delete.php
upload.php
manager.php
routing
loader_resolver.php
helper.php
resources_locator
installer_resources_locator.php
resources_locator_interface.php
default_resources_locator.php
chained_resources_locator.php
router.php
file_locator.php
lock
flock.php
db.php
auth
auth.php
provider_collection.php
provider
base.php
oauth
oauth.php
token_storage.php
service
twitter.php
exception.php
base.php
bitly.php
facebook.php
google.php
service_interface.php
provider_interface.php
db.php
apache.php
index.htm
ldap.php
index.htm
report

  - report_handler_post.php
  - **exception**
    - report_permission_denied_exception.php
    - pm_reporting_disabled_exception.php
    - entity_not_found_exception.php
    - already_reported_exception.php
    - empty_report_exception.php
    - factory_invalid_argument_exception.php
    - invalid_report_exception.php
  - **controller**
    - report.php
  - report_reason_list_provider.php
  - report_handler_pm.php
  - handler_factory.php
  - report_handler.php
  - report_handler_interface.php
- **template**
  - **twig**
    - definition.php
    - twig.php
    - loader.php
    - **tokenparser**
      - event.php
      - includejs.php
      - includeparser.php
      - includephp.php
      - defineparser.php
      - includecss.php
      - php.php
    - environment.php
    - **node**
      - definenode.php
      - **expression**
        - **binary**
          - notequalequal.php
          - equalequal.php
      - event.php
      - includeasset.php
      - includejs.php
      - includephp.php
      - includecss.php
      - includenode.php
      - php.php
    - **extension**
      - routing.php

```
        extension.php
        lexer.php
    base.php
    exception
        user_object_not_available.php
    assets_bag.php
    asset.php
    template.php
    context.php
user.php
cache
    driver
        xcache.php
        dummy.php
        base.php
        wincache.php
        apc.php
        file.php
        driver_interface.php
        memory.php
        redis.php
        memcached.php
        eaccelerator.php
        memcache.php
    service.php
feed
    topics_active.php
    forum.php
    feed_interface.php
    post_base.php
    topics.php
    topic.php
    attachments_base.php
    base.php
    exception
        feed_exception.php
        unauthorized_topic_exception.php
        feed_unavailable_exception.php
        no_forum_exception.php
        no_feed_exception.php
        unauthorized_forum_exception.php
        no_topic_exception.php
        unauthorized_exception.php
    controller
        feed.php
```

- helper.php
- forums.php
- overall.php
- topic_base.php
- news.php

language

- language.php
- language_file_helper.php
- **exception**
  - language_file_not_found.php
  - invalid_plural_rule_exception.php
  - language_exception.php
- language_file_loader.php

search

- base.php
- fulltext_postgres.php
- fulltext_sphinx.php
- fulltext_mysql.php
- **sphinx**
  - config.php
  - config_variable.php
  - config_comment.php
  - config_section.php
- index.htm
- fulltext_native.php

composer.json

profilefields

- **type**
  - type_int.php
  - type_url.php
  - type_interface.php
  - type_date.php
  - type_dropdown.php
  - type_text.php
  - type_string.php
  - type_string_common.php
  - type_googleplus.php
  - type_bool.php
  - type_base.php
- lang_helper.php
- manager.php

log

- dummy.php
- log_interface.php
- log.php

- **di**
  - service_collection_iterator.php
  - **pass**
    - collection_pass.php
  - service_collection.php
  - proxy_instantiator.php
  - ordered_service_collection.php
  - container_builder.php
  - **extension**
    - config.php
    - container_configuration.php
    - core.php
- **message**
  - message.php
  - user_form.php
  - form.php
  - admin_form.php
  - topic_form.php
- filesystem.php
- **module**
  - module_manager.php
  - **exception**
    - module_exception.php
    - module_not_found_exception.php
- json_response.php
- **files**
  - filespec.php
  - **types**
    - type_interface.php
    - base.php
    - form.php
    - local.php
    - remote.php
  - factory.php
  - upload.php
- **textformatter**
  - parser_interface.php
  - utils_interface.php
  - data_access.php
  - **s9e**
    - quote_helper.php
    - parser.php
    - factory.php
    - link_helper.php
    - renderer.php

    - utils.php
  - cache_interface.php
  - renderer_interface.php
- **php**
  - ini.php
- symfony_request.php
- **avatar**
  - **driver**
    - driver.php
    - driver_interface.php
    - gravatar.php
    - local.php
    - remote.php
    - upload.php
  - manager.php
- **extension**
  - provider.php
  - exception.php
  - base.php
  - metadata_manager.php
  - **di**
    - extension_base.php
  - manager.php
  - extension_interface.php
- class_loader.php
- **console**
  - application.php
  - **command**
    - **user**
      - delete.php
      - reclean.php
      - add.php
      - activate.php
    - **update**
      - check.php
    - **reparser**
      - reparse.php
      - list_all.php
    - **config**
      - delete.php
      - set_atomic.php
      - get.php
      - set.php
      - command.php
      - increment.php

- **cache**
  - purge.php
- **dev**
  - migration_tips.php
- **thumbnail**
  - recreate.php
  - delete.php
  - generate.php
- **extension**
  - enable.php
  - purge.php
  - disable.php
  - command.php
  - show.php
- **db**
  - list_command.php
  - revert.php
  - console_migrator_output_handler.php
  - migration_command.php
  - migrate.php
- command.php
- **cron**
  - cron_list.php
  - run.php
- **fixup**
  - fix_left_right_ids.php
  - recalculate_email_hash.php
  - update_hashes.php

exception_subscriber.php

db

- **tools**
  - tools.php
  - tools_interface.php
  - postgres.php
  - factory.php
  - mssql.php
- tools.php
- migrator.php
- **migration**
  - migration.php
  - schema_generator.php
  - exception.php
  - container_aware_migration.php
  - helper.php
  - **tool**

config_text.php
tool_interface.php
config.php
module.php
permission.php
profilefield_base_migration.php
data
v32x
user_notifications_table_index_p3.php
user_notifications_table_temp_index.php
user_notifications_table_unique_index.php
load_user_activity_limit.php
user_notifications_table_index_p1.php
.htaccess
v321rc1.php
user_notifications_table_reduce_column_sizes.php
user_notifications_table_remove_duplicates.php
v321.php
cookie_notice_p2.php
user_notifications_table_index_p2.php
v31x
add_smtp_ssl_context_config_options.php
add_jabber_ssl_context_config_options.php
m_softdelete_global.php
add_log_time_index.php
v316rc1.php
v313.php
v318.php
increase_size_of_emotion.php
m_pm_report.php
v317rc1.php
add_latest_topics_index.php
v311.php
v313rc2.php
remove_duplicate_migrations.php
.htaccess
update_hashes.php
increase_size_of_dateformat.php
v313rc1.php
v315.php
v3111.php
v314rc2.php
profilefield_remove_underscore_from_alpha.php
style_update.php
v314rc1.php

v316.php
v317pl1.php
v314.php
v319.php
plupload_last_gc_dynamic.php
v3110.php
v319rc1.php
v312rc1.php
v317.php
v312.php
profilefield_yahoo_update_url.php
v318rc1.php
v3111rc1.php
update_custom_bbcodes_with_idn.php
v3110rc1.php
v315rc1.php

v310

reset_missing_captcha_plugin.php
forgot_password.php
mysql_fulltext_drop.php
contact_admin_acp_module.php
acp_style_components_module.php
beta4.php
profilefield_wlm_cleanup.php
profilefield_icq.php
profilefield_website.php
ucp_popuppm_module.php
profilefield_aol_cleanup.php
jquery_update2.php
namespaces.php
board_contact_name.php
rc3.php
profilefield_occupation.php
timezone.php
gold.php
alpha3.php
rc5.php
extensions_version_check_force_unstable.php
profilefield_field_validation_length.php
migrations_table.php
allow_cdn.php
auth_provider_oauth2.php
profilefield_change_load_settings.php
notifications_schema_fix.php
beta2.php

beta1.php
profilefield_on_memberlist.php
search_type.php
profilefield_googleplus.php
profilefield_location.php
boardindex.php
avatars.php
captcha_plugins.php
.htaccess
acp_prune_users_module.php
profilefield_yahoo.php
timezone_p2.php
rc4.php
profilefield_youtube.php
profilefield_interests.php
remove_acp_styles_cache.php
soft_delete_mod_convert.php
profilefield_icq_cleanup.php
mod_rewrite.php
softdelete_mcp_modules.php
prune_shadow_topics.php
topic_sort_username.php
passwords.php
passwords_convert_p1.php
passwords_p2.php
dev.php
postgres_fulltext_drop.php
extensions.php
live_searches_config.php
signature_module_auth.php
rename_too_long_indexes.php
avatar_types.php
notifications_cron.php
alpha2.php
profilefield_contact_field.php
notifications.php
passwords_convert_p2.php
alpha1.php
profilefield_cleanup.php
notification_options_reconvert.php
profilefield_aol.php
bot_update.php
rc1.php
profilefield_website_cleanup.php
profilefield_location_cleanup.php

reported_posts_display.php
teampage.php
notifications_use_full_name.php
profilefield_facebook.php
notifications_cron_p2.php
profilefield_yahoo_cleanup.php
style_update_p2.php
profilefield_show_novalue.php
rc6.php
softdelete_p2.php
profilefield_twitter.php
plupload.php
profilefield_types.php
config_db_text.php
profilefield_wlm.php
profilefield_skype.php
jquery_update.php
beta3.php
rc2.php
style_update_p1.php
contact_admin_form.php
softdelete_p1.php
auth_provider_oauth.php
soft_delete_mod_convert2.php

v320

remove_profilefield_wlm.php
remove_outdated_media.php
add_help_phpbb.php
oauth_states.php
report_id_auto_increment.php
notifications_board.php
log_post_id.php
v320a2.php
allowed_schemes_links.php
icons_alt.php
.htaccess
v320b1.php
v320rc2.php
v320rc1.php
cookie_notice.php
dev.php
v320a1.php
font_awesome_update.php
v320b2.php
v320.php

announce_global_permission.php
remote_upload_validation.php
default_data_type_ids.php
text_reparser.php

v30x

release_3_0_12_rc1.php
local_url_bbcode.php
release_3_0_4.php
release_3_0_8_rc1.php
release_3_0_8.php
release_3_0_12.php
release_3_0_12_rc3.php
release_3_0_13_rc1.php
release_3_0_1.php
release_3_0_6.php
release_3_0_6_rc3.php
release_3_0_13_pl1.php
release_3_0_11_rc2.php
release_3_0_1_rc1.php
release_3_0_10_rc3.php
.htaccess
release_3_0_10_rc1.php
release_3_0_4_rc1.php
release_3_0_5.php
release_3_0_10_rc2.php
release_3_0_11.php
release_3_0_6_rc4.php
release_3_0_9_rc1.php
release_3_0_5_rc1part2.php
release_3_0_13.php
release_3_0_2_rc2.php
release_3_0_2_rc1.php
release_3_0_6_rc1.php
release_3_0_9.php
release_3_0_7_rc1.php
release_3_0_2.php
release_3_0_0.php
release_3_0_3_rc1.php
release_3_0_3.php
release_3_0_7_rc2.php
release_3_0_7.php
release_3_0_10.php
release_3_0_7_pl1.php
release_3_0_5_rc1.php
release_3_0_11_rc1.php

- release_3_0_6_rc2.php
      - release_3_0_14_rc1.php
      - release_3_0_9_rc3.php
      - release_3_0_9_rc2.php
      - release_3_0_14.php
      - release_3_0_9_rc4.php
      - release_3_0_12_rc2.php
    - migration_interface.php
  - sql_insert_buffer.php
  - **output_handler**
    - migrator_output_handler_interface.php
    - log_wrapper_migrator_output_handler.php
    - html_migrator_output_handler.php
    - installer_migrator_output_handler.php
    - null_migrator_output_handler.php
  - **driver**
    - sqlite3.php
    - driver.php
    - mssql_base.php
    - mysqli.php
    - postgres.php
    - driver_interface.php
    - factory.php
    - mysql.php
    - mysql_base.php
    - mssqlnative.php
    - mssql_odbc.php
    - oracle.php
  - **extractor**
    - extractor_interface.php
    - **exception**
      - extractor_not_initialized_exception.php
      - invalid_format_exception.php
    - base_extractor.php
    - postgres_extractor.php
    - factory.php
    - oracle_extractor.php
    - mssql_extractor.php
    - sqlite3_extractor.php
    - mysql_extractor.php
- **mimetype**
  - guesser_base.php
  - extension_guesser.php
  - guesser_interface.php
  - guesser.php

- content_guesser.php
- **captcha**
  - char_cube3d.php
  - factory.php
  - gd.php
  - colour_manager.php
  - **plugins**
    - recaptcha.php
    - gd.php
    - nogd.php
    - captcha_abstract.php
    - qa.php
    - gd_wave.php
  - non_gd.php
  - gd_wave.php
- **cron**
  - manager.php
  - **task**
    - base.php
    - parametrized.php
    - **text_reparser**
      - reparser.php
    - wrapper.php
    - task.php
    - **core**
      - prune_forum.php
      - tidy_plupload.php
      - queue.php
      - update_hashes.php
      - tidy_sessions.php
      - tidy_warnings.php
      - prune_notifications.php
      - prune_shadow_topics.php
      - tidy_search.php
      - prune_all_forums.php
      - tidy_database.php
      - tidy_cache.php
- **notification**
  - exception.php
  - **type**
    - post.php
    - post_in_queue.php
    - report_post_closed.php
    - report_pm_closed.php
    - type_interface.php

            topic.php
            pm.php
            base.php
            quote.php
            admin_activate_user.php
            bookmark.php
            group_request.php
            disapprove_post.php
            approve_post.php
            report_pm.php
            disapprove_topic.php
            approve_topic.php
            report_post.php
            group_request_approved.php
            topic_in_queue.php
        manager.php
        method
            method_interface.php
            jabber.php
            base.php
            messenger_base.php
            board.php
            email.php
    error_collector.php
    version_helper.php
    path_helper.php
    request
        request_interface.php
        request.php
        type_cast_helper.php
        type_cast_helper_interface.php
        deactivated_super_global.php
    file_downloader.php
web.config
composer.lock
posting.php
viewforum.php
report.php
images
    spacer.gif
    upload_icons
        txt.gif
        xls.gif
        rar.gif
        exe.gif

flash.gif
jpg.gif
bmp.gif
ppt.gif
mpg.gif
mov.gif
avi.gif
zip.gif
netscape.gif
gif.gif
doc.gif
html.gif
mp3.gif
mid.gif
pdf.gif
wav.gif
avatars
upload
.htaccess
index.htm
index.htm
gallery
index.htm
index.htm
smilies
icon_arrow.gif
icon_lol.gif
icon_neutral.gif
icon_e_wink.gif
icon_cool.gif
icon_e_smile.gif
icon_e_biggrin.gif
icon_redface.gif
icon_idea.gif
icon_e_ugeek.gif
icon_exclaim.gif
icon_eek.gif
icon_razz.gif
icon_mad.gif
icon_evil.gif
icon_cry.gif
icon_mrgreen.gif
icon_question.gif
icon_e_surprised.gif
icon_twisted.gif

    icon_e_geek.gif
    icon_e_sad.gif
    icon_e_confused.gif
    icon_rolleyes.gif
  icons
    smile
      redface.gif
      info.gif
      question.gif
      index.htm
      mrgreen.gif
      alert.gif
    index.htm
    misc
      fire.gif
      thinking.gif
      heart.gif
      star.gif
      radioactive.gif
      index.htm
  ranks
    index.htm
mcp.php
download
  file.php
  index.htm
.htaccess
config
  .htaccess
  production
    routing
      environment.yml
    container
      parameters.yml
      services.yml
      environment.yml
    config.yml
  installer
    routing
      installer.yml
      environment.yml
    container
      services_install_finish.yml
      services_install_requirements.yml
      services_update_filesystem.yml

parameters.yml
services_update_requirements.yml
services_update_database.yml
services_install_database.yml
services.yml
services_install_obtain_data.yml
services_install_data.yml
services_file_updater.yml
services_install_console.yml
environment.yml
services_update_obtain_data.yml
services_install_filesystem.yml
services_install_controller.yml
services_install_navigation.yml
services_installer.yml
config.yml
default
routing
routing.yml
feed.yml
help.yml
report.yml
container
services_migrator.yml
services_help.yml
services_files.yml
services_module.yml
services_db.yml
services_text_reparser.yml
services_hook.yml
services_report.yml
parameters.yml
services_attachment.yml
services_http.yml
services_event.yml
services_console.yml
services_user.yml
services.yml
services_profilefield.yml
services_cron.yml
services_content.yml
services_notification.yml
services_routing.yml
services_feed.yml
services_twig.yml

services_captcha.yml
services_php.yml
services_language.yml
services_password.yml
services_mimetype_guesser.yml
services_avatar.yml
tables.yml
services_auth.yml
services_text_formatter.yml
services_filesystem.yml
config.yml
config.php
store
io_lock.lock
.htaccess
index.htm
styles
prosilver
style.cfg
template
mcp_queue.html
mcp_message.html
ucp_agreement.html
posting_smilies.html
navbar_footer.html
mcp_topic.html
ucp_pm_message_header.html
memberlist_view.html
viewonline_body.html
mcp_warn_post.html
mcp_whois.html
forum_fn.js
faq_body.html
login_body_oauth.html
ucp_main_subscribed.html
ucp_profile_avatar.html
drafts.html
report_body.html
ucp_pm_history.html
captcha_qa.html
index_body.html
mcp_warn_list.html
display_options.html
posting_attach_body.html
mcp_logs.html

posting_topic_review.html
posting_editor.html
quickreply_editor.html
ajax.js
ucp_avatar_options_local.html
ucp_resend.html
ucp_main_front.html
memberlist_body.html
viewonline_whois.html
captcha_recaptcha.html
ucp_prefs_view.html
login_body.html
search_body.html
attachment.html
mcp_notes_user.html
ucp_pm_message_footer.html
memberlist_im.html
login_forum.html
ucp_auth_link.html
mcp_notes_front.html
overall_header.html
ucp_avatar_options_remote.html
ucp_footer.html
ucp_pm_viewmessage_print.html
mcp_reports.html
search_results.html
mcp_footer.html
ucp_zebra_foes.html
mcp_post.html
ucp_profile_reg_details.html
mcp_approve.html
mcp_move.html
overall_footer.html
ucp_pm_viewmessage.html
forumlist_body.html
ucp_prefs_post.html
posting_layout.html
ucp_auth_link_oauth.html
mcp_ban.html
posting_body.html
plupload.html
viewtopic_print.html
viewtopic_body.html
profilefields
    string.html

int.html
url.html
text.html
bool.html
dropdown.html
date.html
mcp_header.html
ucp_main_drafts.html
mcp_warn_user.html
posting_review.html
memberlist_email.html
ucp_avatar_options.html
ucp_pm_options.html
memberlist_search.html
posting_poll_body.html
ucp_main_bookmarks.html
ucp_groups_manage.html
posting_preview.html
jumpbox.html
ucp_profile_profile_info.html
ucp_profile_signature.html
ucp_zebra_friends.html
ucp_groups_membership.html
ucp_avatar_options_upload.html
timezone.js
index.htm
memberlist_team.html
mcp_warn_front.html
ucp_prefs_personal.html
posting_pm_layout.html
notification_dropdown.html
confirm_body.html
viewforum_body.html
pagination.html
navbar_header.html
ucp_pm_viewfolder.html
mcp_front.html
posting_pm_header.html
ucp_profile_autologin_keys.html
ucp_register.html
ucp_notifications.html
viewtopic_topic_tools.html
posting_buttons.html
confirm_delete_body.html
captcha_default.html

- bbcode.html
- ucp_avatar_options_gravatar.html
- ucp_attachments.html
- simple_header.html
- ucp_remind.html
- ucp_header.html
- mcp_forum.html
- message_body.html
- ucp_login_link.html
- timezone_option.html
- simple_footer.html

theme
- base.css
- plupload.css
- print.css
- **images**
  - topic_read_hot.gif
  - forum_read.gif
  - topic_unread_locked_mine.gif
  - icon_offline.gif
  - topic_read_locked_mine.gif
  - loading.gif
  - quote_rtl.gif
  - forum_read_subforum.gif
  - topic_read_locked.gif
  - forum_link.gif
  - topic_unread.gif
  - site_logo.gif
  - announce_read.gif
  - sticky_unread_mine.gif
  - no_avatar.gif
  - **plupload**
    - done.gif
    - error.gif
    - throbber.gif
  - announce_unread_mine.gif
  - sticky_read_locked.gif
  - icon_online.gif
  - topic_unread_locked.gif
  - icons_contact.png
  - topic_read_mine.gif
  - forum_unread.gif
  - topic_moved.gif
  - announce_unread_locked.gif
  - icon_download.gif

forum_read_locked.gif
forum_unread_locked.gif
sticky_read_mine.gif
icon_rate_good.gif
topic_unread_hot.gif
topic_unread_mine.gif
bg_list.gif
icon_rate_bad.gif
sticky_unread_locked.gif
sticky_read_locked_mine.gif
announce_unread_locked_mine.gif
announce_read_mine.gif
announce_unread.gif
topic_unread_hot_mine.gif
sticky_read.gif
announce_read_locked.gif
topic_read_hot_mine.gif
index.htm
topic_read.gif
bg_header.gif
announce_read_locked_mine.gif
sticky_unread.gif
forum_unread_subforum.gif
sticky_unread_locked_mine.gif
quote.gif

en

stylesheet.css
icon_user_online.gif
common.css
links.css
content.css
cp.css
icons.css
normalize.css
bidi.css
tweaks.css
forms.css
responsive.css
index.htm
stylesheet.css
buttons.css
colours.css
utilities.css

all

template

```
      feed.xml.twig
ucp.php
feed.php
docs
  update-config.sample.yml
  nginx.sample.conf
  lighttpd.sample.conf
  LICENSE.txt
  README.html
  INSTALL.html
  coding-guidelines.html
  CREDITS.txt
  assets
    images
      site_logo.gif
      icon_back_top.gif
      bg_header.gif
    css
      stylesheet.css
  CHANGELOG.html
  vagrant.md
  auth_api.html
  FAQ.html
  install-config.sample.yml
  events.md
  sphinx.sample.conf
install0
  convert
    convertor.php
    controller
      convertor.php
    convert.php
  phpbbcli.php
  schemas
    oracle_schema.sql
    schema.json
    postgres_schema.sql
    index.htm
    schema_data.sql
  startup.php
  index.html
  app.php
  convertors
    convert_phpbb20.php
    functions_phpbb20.php
```

phpinfo.php
cache
.htaccess
production
sql_6ccd874eeeb496ca0a1a4bdf6de96bff.php.lock
twig
2e
2ecd40bb4ba59424f8a425fd01d1f2138da12a37d40aeb386bc2357292a07c70.php
ff
ffb0d1a9c5f3d5e440339e264504bb3366be31054511b543a25efcbf532e10b6.php
ae
ae55498a99117bc25b573d77434df66f1d2947d5d98e0184785721c47445c1a6.php
d8
d84ae8f20cb1497944ec60b3559d71004f912072f1957e997862a15c6ef5264b.php
dc
dc90cd3cebc4d28ce6a228c31fb14340f6fd7e714580bcc1c550f031bddebd7b.php
66
66195dc2e6f21f4b32ba701fee5120022f8845663e822fa5f68cbf0aacc57237.php
cd
cd79fd4991f80a148ee441c86df3b0cbd22a7602f70912aa7c9f6af167f6a554.php
78
78db860397cc75ed63861f11236562f51a4a46edf3d36577c2428e9ac93371e6.php
12
12ac3868466979334cc1c7147946e9abbd54cfb246a3ea292c6e7d19df9c731b.php
8d
8d48836156feee4e76470d8cad88d970a02ad3033fe6eb3de1659281a3a1a844.php
73
73844f3add621f10fde7d77227cf6d5df189d47d008639dc59d55fc85e97a3a4.php
6c
6c87168b8bab7da6fb514f74c59527ec4d0bda347b14ac37cb3256f467955edb.php
b2
b2715fea92c5c6ff54b5609e3fe530ccb2fd3c69c79f6ad8d2af1a69540dc512.php
7f
7f26a145a8ba23af3e1bc5d2c8df2884fb30524dec33393b0b3d80eb32822910.php
e2
e287dc5369d824c26d593cd9492f098d607e2eb397d84d6da2db13622ec133c7.php
dd
dddfb2f2d5101fcd1b1752bfed7f6fba822dbf61842d2bcfe42969990d6fd889.php
05
05463c3a05c5ea9dd33c22e6567a7a1ed3a037be45670d7077897ae9252fa35f.php
c6
c6a13c9cd2be2929a17406ef0d3514fafc851826c7f6929050a007b177ffc92e.php
sql_b152bea2b86cc301f01da4d7125e73ba.php
data_hooks.php
s9e_renderer_862a96aeb5d77e490a8e75fbec6dc581f7952f76.php

data_ext.php
data_icons.php
data_acl_options.php
data_global.php.lock
url_matcher.php
sql_aedc7080a3f04c7f443929041f90a8eb.php
autoload_4335734bbdd20f586549a504dff5f80c.php
sql_efd26735c3bcbef398e23fb85e7ccd99.php
data_disallowed_usernames.php
data_cfg_prosilver.php
data_bots.php
container_4335734bbdd20f586549a504dff5f80c.php
sql_9dc91161799801b44926b824ccc5cf51.php
sql_6ccd874eeeb496ca0a1a4bdf6de96bff.php
url_matcher.php.meta
data_text_formatter_renderer.php
sql_819dc160ed61cca154e8c4eced23fc51.php
sql_819dc160ed61cca154e8c4eced23fc51.php.lock
container_4335734bbdd20f586549a504dff5f80c.php.meta
data_cron.lock_check.php
data_text_formatter_parser.php
data_cron.lock_check.php.lock
url_generator.php.meta
url_generator.php
data_global.php
data_role_cache.php.lock
data_role_cache.php
installer
index.htm
memberlist.php
language
en
posting.php
viewforum.php
help
bbcode.php
faq.php
migrator.php
install.php
groups.php
mcp.php
iso.txt
email
profile_send_im.txt
report_pm.txt

newtopic_notify.txt
contact_admin.txt
group_added.txt
post_disapproved.txt
topic_disapproved.txt
bookmark.txt
post_in_queue.txt
user_activate.txt
post_approved.txt
admin_welcome_inactive.txt
report_closed.txt
report_deleted.txt
user_welcome_inactive.txt
user_activate_inactive.txt
topic_in_queue.txt
coppa_resend_inactive.txt
privmsg_notify.txt
user_resend_inactive.txt
admin_welcome_activated.txt
user_activate_passwd.txt
pm_report_deleted.txt
quote.txt
admin_activate.txt
test.txt
report_post.txt
short
- report_pm.txt
- newtopic_notify.txt
- post_disapproved.txt
- topic_disapproved.txt
- bookmark.txt
- post_in_queue.txt
- post_approved.txt
- topic_in_queue.txt
- privmsg_notify.txt
- quote.txt
- report_post.txt
- topic_notify.txt
- topic_approved.txt

profile_send_email.txt
user_reactivate_account.txt
installed.txt
group_request.txt
pm_report_closed.txt
coppa_welcome_inactive.txt

email_notify.txt
user_welcome.txt
user_remind_inactive.txt
index.htm
forum_notify.txt
topic_notify.txt
topic_approved.txt
admin_send_email.txt
ucp.php
captcha_recaptcha.php
memberlist.php
search.php
cli.php
app.php
captcha_qa.php
index.htm
common.php
plupload.php
viewtopic.php
acp
language.php
posting.php
ban.php
groups.php
permissions.php
prune.php
database.php
modules.php
styles.php
profile.php
extensions.php
search.php
forums.php
bots.php
index.htm
board.php
common.php
email.php
attachments.php
users.php
permissions_phpbb.php
brazilian_portuguese_1_3_1
styles
prosilver
theme

pt_br
stylesheet.css
icon_user_online.gif
language
ext
phpbb
viglink
language
pt_br
viglink_module_acp.php
info_acp_viglink.php
pt_br
posting.php
viewforum.php
AUTHORS.md
help
bbcode.php
faq.php
migrator.php
install.php
groups.php
mcp.php
iso.txt
email
profile_send_im.txt
report_pm.txt
newtopic_notify.txt
contact_admin.txt
group_added.txt
post_disapproved.txt
topic_disapproved.txt
bookmark.txt
post_in_queue.txt
user_activate.txt
post_approved.txt
admin_welcome_inactive.txt
report_closed.txt
report_deleted.txt
user_welcome_inactive.txt
user_activate_inactive.txt
topic_in_queue.txt
coppa_resend_inactive.txt
privmsg_notify.txt
user_resend_inactive.txt
admin_welcome_activated.txt

user_activate_passwd.txt
pm_report_deleted.txt
quote.txt
admin_activate.txt
test.txt
report_post.txt
short
report_pm.txt
newtopic_notify.txt
post_disapproved.txt
topic_disapproved.txt
bookmark.txt
post_in_queue.txt
post_approved.txt
topic_in_queue.txt
privmsg_notify.txt
quote.txt
report_post.txt
topic_notify.txt
topic_approved.txt
profile_send_email.txt
user_reactivate_account.txt
installed.txt
group_request.txt
pm_report_closed.txt
coppa_welcome_inactive.txt
email_notify.txt
user_welcome.txt
user_remind_inactive.txt
index.htm
forum_notify.txt
topic_notify.txt
topic_approved.txt
admin_send_email.txt
ucp.php
captcha_recaptcha.php
memberlist.php
search.php
cli.php
app.php
LICENSE
captcha_qa.php
index.htm
common.php
plupload.php

    - viewtopic.php
    - **acp**
      - language.php
      - posting.php
      - ban.php
      - groups.php
      - permissions.php
      - prune.php
      - database.php
      - modules.php
      - styles.php
      - profile.php
      - extensions.php
      - search.php
      - forums.php
      - bots.php
      - index.htm
      - board.php
      - common.php
      - email.php
      - attachments.php
      - users.php
      - permissions_phpbb.php
  - index.htm
- index.php
- **assets**
  - **plupload**
    - plupload.full.min.js
  - **javascript**
    - editor.js
    - core.js
    - plupload.js
    - jquery.min.js
    - installer.js
  - **css**
    - font-awesome.min.css
  - **fonts**
    - fontawesome-webfont.woff
    - fontawesome-webfont.woff2
    - fontawesome-webfont.svg
    - FontAwesome.otf
    - fontawesome-webfont.eot
    - fontawesome-webfont.ttf
  - **cookieconsent**
    - cookieconsent.min.css

        cookieconsent.min.js
    viewonline.php
    search.php
    vendor
      zendframework
        zend-eventmanager
          composer.json
          src
            Filter
              FilterInterface.php
              FilterIterator.php
            AbstractListenerAggregate.php
            SharedEventAggregateAwareInterface.php
            SharedEventManager.php
            EventManagerAwareInterface.php
            Exception
              InvalidCallbackException.php
              DomainException.php
              ExceptionInterface.php
              InvalidArgumentException.php
            EventInterface.php
            Event.php
            ProvidesEvents.php
            SharedEventManagerAwareInterface.php
            EventManager.php
            EventsCapableInterface.php
            StaticEventManager.php
            SharedEventManagerInterface.php
            EventManagerInterface.php
            FilterChain.php
            EventManagerAwareTrait.php
            ListenerAggregateInterface.php
            ResponseCollection.php
            GlobalEventManager.php
            SharedListenerAggregateInterface.php
            ListenerAggregateTrait.php
          LICENSE.md
        zend-code
          composer.json
          src
            Scanner
              FileScanner.php
              DocBlockScanner.php
              ConstantScanner.php
              PropertyScanner.php

- DirectoryScanner.php
  - FunctionScanner.php
  - ClassScanner.php
  - ValueScanner.php
  - TokenArrayScanner.php
  - DerivedClassScanner.php
  - AnnotationScanner.php
  - CachingFileScanner.php
  - Util.php
  - MethodScanner.php
  - ScannerInterface.php
  - ParameterScanner.php
  - AggregateDirectoryScanner.php
- **Exception**
  - BadMethodCallException.php
  - RuntimeException.php
  - ExceptionInterface.php
  - InvalidArgumentException.php
- **Generic**
  - **Prototype**
    - PrototypeGenericInterface.php
    - PrototypeClassFactory.php
    - PrototypeInterface.php
- NameInformation.php
- **Generator**
  - FileGeneratorRegistry.php
  - ValueGenerator.php
  - ClassGenerator.php
  - BodyGenerator.php
  - **Exception**
    - RuntimeException.php
    - ExceptionInterface.php
    - InvalidArgumentException.php
  - GeneratorInterface.php
  - AbstractGenerator.php
  - TraitUsageGenerator.php
  - PropertyGenerator.php
  - ParameterGenerator.php
  - TraitUsageInterface.php
  - **DocBlock**
    - TagManager.php
    - **Tag**
      - TagInterface.php
      - ParamTag.php
      - AuthorTag.php

            ReturnTag.php
            LicenseTag.php
            AbstractTypeableTag.php
            GenericTag.php
            ThrowsTag.php
            MethodTag.php
            PropertyTag.php
        Tag.php
    FileGenerator.php
    DocBlockGenerator.php
    PropertyValueGenerator.php
    MethodGenerator.php
    AbstractMemberGenerator.php
    TraitGenerator.php
Reflection
    FunctionReflection.php
    ReflectionInterface.php
    DocBlockReflection.php
    MethodReflection.php
    Exception
        BadMethodCallException.php
        RuntimeException.php
        ExceptionInterface.php
        InvalidArgumentException.php
    FileReflection.php
    PropertyReflection.php
    DocBlock
        TagManager.php
        Tag
            TagInterface.php
            ParamTag.php
            AuthorTag.php
            ReturnTag.php
            LicenseTag.php
            GenericTag.php
            ThrowsTag.php
            MethodTag.php
            PhpDocTypedTagInterface.php
            PropertyTag.php
    ParameterReflection.php
    ClassReflection.php
Annotation
    Parser
        ParserInterface.php
        DoctrineAnnotationParser.php

GenericAnnotationParser.php
AnnotationCollection.php
AnnotationInterface.php
AnnotationManager.php
LICENSE.md
zend-stdlib
composer.json
src
Request.php
compatibility
autoload.php
Glob.php
InitializableInterface.php
ParameterObjectInterface.php
ErrorHandler.php
Guard
GuardUtils.php
EmptyGuardTrait.php
NullGuardTrait.php
ArrayOrTraversableGuardTrait.php
AllGuardsTrait.php
Exception
BadMethodCallException.php
InvalidCallbackException.php
ExtensionNotLoadedException.php
DomainException.php
RuntimeException.php
LogicException.php
ExceptionInterface.php
InvalidArgumentException.php
MessageInterface.php
JsonSerializable
PhpLegacyCompatibility.php
ArrayStack.php
StringWrapper
Native.php
Intl.php
Iconv.php
StringWrapperInterface.php
MbString.php
AbstractStringWrapper.php
ArraySerializableInterface.php
JsonSerializable.php
PriorityList.php
ArrayUtils

- MergeReplaceKey.php
- MergeReplaceKeyInterface.php
- MergeRemoveKey.php

ParametersInterface.php

PriorityQueue.php

SplPriorityQueue.php

Hydrator

- **Filter**
  - GetFilter.php
  - FilterInterface.php
  - FilterComposite.php
  - IsFilter.php
  - MethodMatchFilter.php
  - OptionalParametersFilter.php
  - NumberOfParameterFilter.php
  - FilterProviderInterface.php
  - HasFilter.php
- AbstractHydrator.php
- Reflection.php
- HydratorOptionsInterface.php
- HydratorAwareTrait.php
- ObjectProperty.php
- **Strategy**
  - DateTimeFormatterStrategy.php
  - ExplodeStrategy.php
  - **Exception**
    - ExceptionInterface.php
    - InvalidArgumentException.php
  - StrategyInterface.php
  - SerializableStrategy.php
  - BooleanStrategy.php
  - StrategyChain.php
  - ClosureStrategy.php
  - DefaultStrategy.php
- StrategyEnabledInterface.php
- ArraySerializable.php
- HydratorPluginManager.php
- **NamingStrategy**
  - MapNamingStrategy.php
  - CompositeNamingStrategy.php
  - NamingStrategyInterface.php
  - UnderscoreNamingStrategy.php
  - IdentityNamingStrategy.php
  - ArrayMapNamingStrategy.php
- DelegatingHydratorFactory.php

HydratorInterface.php
NamingStrategyEnabledInterface.php
DelegatingHydrator.php
ClassMethods.php
Aggregate
HydratorListener.php
HydrateEvent.php
ExtractEvent.php
AggregateHydrator.php
HydrationInterface.php
HydratorAwareInterface.php
FilterEnabledInterface.php
Parameters.php
ResponseInterface.php
ArrayObject.php
Extractor
ExtractionInterface.php
Response.php
StringUtils.php
DispatchableInterface.php
ArrayUtils.php
SplQueue.php
DateTime.php
SplStack.php
RequestInterface.php
AbstractOptions.php
Message.php
CallbackHandler.php
LICENSE.md
paragonie
random_compat
psalm-autoload.php
psalm.xml
RATIONALE.md
lib
cast_to_int.php
random_bytes_dev_urandom.php
random_bytes_libsodium_legacy.php
random_bytes_com_dotnet.php
error_polyfill.php
random_bytes_mcrypt.php
random.php
random_bytes_libsodium.php
byte_safe_strings.php
random_bytes_openssl.php

- random_int.php
- composer.json
- LICENSE
- build-phar.sh
- **twig**
  - **twig**
    - **lib**
      - **Twig**
        - **Filter**
          - Method.php
          - Function.php
          - Node.php
        - **Profiler**
          - **Node**
            - LeaveProfile.php
            - EnterProfile.php
          - Profile.php
          - **Dumper**
            - Blackfire.php
            - Text.php
            - Html.php
          - **NodeVisitor**
            - Profiler.php
        - TemplateWrapper.php
        - CompilerInterface.php
        - SimpleTest.php
        - CacheInterface.php
        - FunctionCallableInterface.php
        - FilterCallableInterface.php
        - ParserInterface.php
        - **Function**
          - Method.php
          - Function.php
          - Node.php
        - TestInterface.php
        - FileExtensionEscapingStrategy.php
        - **Node**
          - Include.php
          - Do.php
          - Module.php
          - Body.php
          - SetTemp.php
          - SandboxedPrint.php
          - If.php
          - CheckSecurity.php

- AutoEscape.php
- Text.php
- Macro.php
- Print.php
- ForLoop.php
- Embed.php
- Block.php
- Set.php
- Flush.php
- With.php
- **Expression**
  - **Filter**
    - Default.php
  - AssignName.php
  - Array.php
  - Call.php
  - NullCoalesce.php
  - Name.php
  - Binary.php
  - Constant.php
  - ExtensionReference.php
  - Parent.php
  - MethodCall.php
  - **Unary**
    - Not.php
    - Neg.php
    - Pos.php
  - BlockReference.php
  - **Test**
    - Defined.php
    - Even.php
    - Divisibleby.php
    - Null.php
    - Constant.php
    - Odd.php
    - Sameas.php
  - Function.php
  - GetAttr.php
  - **Binary**
    - BitwiseXor.php
    - BitwiseAnd.php
    - EndsWith.php
    - Sub.php
    - Matches.php
    - Equal.php

NotIn.php
Power.php
BitwiseOr.php
Mul.php
Concat.php
FloorDiv.php
Mod.php
And.php
Or.php
Div.php
Range.php
StartsWith.php
GreaterEqual.php
Add.php
Less.php
Greater.php
In.php
LessEqual.php
NotEqual.php
Conditional.php
TempName.php
Unary.php
Filter.php
Test.php
BlockReference.php
For.php
Spaceless.php
Sandbox.php
Import.php
Expression.php
TokenParser.php
SourceContextLoaderInterface.php
Cache
Null.php
Filesystem.php
LexerInterface.php
NodeCaptureInterface.php
BaseNodeVisitor.php
ContainerRuntimeLoader.php
TokenParserBrokerInterface.php
Loader
Array.php
Chain.php
Filesystem.php
String.php

TokenParserInterface.php
ExistsLoaderInterface.php
Template.php
ExtensionInterface.php
Error.php
Autoloader.php
Parser.php
LoaderInterface.php
Extension
- InitRuntimeInterface.php
- Profiler.php
- GlobalsInterface.php
- Core.php
- Escaper.php
- Debug.php
- Optimizer.php
- Sandbox.php
- Staging.php
- StringLoader.php

FilterInterface.php
Compiler.php
RuntimeLoaderInterface.php
ExpressionParser.php
Error
- Runtime.php
- Loader.php
- Syntax.php

Sandbox
- SecurityNotAllowedFilterError.php
- SecurityPolicyInterface.php
- SecurityNotAllowedMethodError.php
- SecurityNotAllowedFunctionError.php
- SecurityError.php
- SecurityNotAllowedPropertyError.php
- SecurityPolicy.php
- SecurityNotAllowedTagError.php

SimpleFunction.php
TokenParser
- Include.php
- Do.php
- Use.php
- Extends.php
- From.php
- If.php
- AutoEscape.php

Macro.php
Embed.php
Block.php
Set.php
Flush.php
With.php
For.php
Spaceless.php
Sandbox.php
Import.php
Filter.php

Util

DeprecationCollector.php
TemplateDirIterator.php

FunctionInterface.php
NodeVisitorInterface.php
SimpleFilter.php
NodeInterface.php
Markup.php
Extension.php
TestCallableInterface.php

Test

Method.php
NodeTestCase.php
IntegrationTestCase.php
Function.php
Node.php

Function.php
TemplateInterface.php
TokenParserBroker.php
Environment.php
NodeTraverser.php

NodeVisitor

Escaper.php
Optimizer.php
Sandbox.php
SafeAnalysis.php

FactoryRuntimeLoader.php
Lexer.php
NodeOutputInterface.php
Node.php
Filter.php
Token.php
Test.php
TokenStream.php

Source.php
.php_cs.dist
composer.json
LICENSE
react
promise
composer.json
src
RejectedPromise.php
Exception
LengthException.php
FulfilledPromise.php
PromisorInterface.php
functions.php
CancellablePromiseInterface.php
Deferred.php
LazyPromise.php
Promise.php
ExtendedPromiseInterface.php
PromiseInterface.php
CancellationQueue.php
UnhandledRejectionException.php
functions_include.php
LICENSE
psr
log
Psr
Log
LoggerAwareInterface.php
LoggerInterface.php
AbstractLogger.php
NullLogger.php
LoggerAwareTrait.php
LoggerTrait.php
InvalidArgumentException.php
LogLevel.php
composer.json
LICENSE
s9e
text-formatter
composer.json
src
Parser
utils.js
Logger.js

- Logger.php
- Tag.js
- NullLogger.js
- Tag.php
- BuiltInFilters.php
- BuiltInFilters.js

render.js

Renderers

- Unformatted.php
- XSLT.php

Parser.js

Utils

- **Http**
  - Client.php
  - **Clients**
    - Native.php
    - Curl.php
- Http.php

Unparser.php

Configurator.php

Renderer.php

Parser.php

Bundle.php

Configurator

- **Validators**
  - TemplateParameterName.php
- JavaScript.php
- **TemplateNormalizations**
  - TransposeComments.php
  - ConvertCurlyExpressionsInText.php
  - MergeIdenticalConditionalBranches.php
  - SortAttributesByName.php
  - Custom.php
  - OptimizeNestedConditionals.php
  - MergeConsecutiveCopyOf.php
- **Items**
  - TemplateDocument.php
  - **AttributeFilters**
    - EmailFilter.php
    - IntFilter.php
    - FloatFilter.php
    - AlnumFilter.php
    - SimpletextFilter.php
    - NumberFilter.php
    - FontfamilyFilter.php

    - UintFilter.php
    - IpFilter.php
    - IpportFilter.php
    - IdentifierFilter.php
    - RangeFilter.php
    - FalseFilter.php
    - RegexpFilter.php
    - ColorFilter.php
    - MapFilter.php
    - HashmapFilter.php
    - Ipv6Filter.php
    - Ipv4Filter.php
    - ChoiceFilter.php
  - AttributePreprocessor.php
  - UnsafeTemplate.php
- Bundle.php
- **Collections**
  - TagList.php
  - MinifierList.php
  - AttributeList.php
- **JavaScript**
  - ConfigOptimizer.php
  - ConfigValue.php
  - RegexpConvertor.php
  - Minifier.php
  - OnlineMinifier.php
  - externs.application.js
  - Encoder.php
  - StylesheetCompressor.php
  - HintGenerator.php
  - Dictionary.php
  - **Minifiers**
    - HostedMinifier.php
    - MatthiasMullieMinify.php
    - RemoteCache.php
    - Noop.php
    - ClosureCompilerService.php
    - FirstAvailable.php
    - ClosureCompilerApplication.php
  - externs.service.js
  - CallbackGenerator.php
- **TemplateChecks**
  - RestrictFlashNetworking.php
  - DisallowElement.php
  - DisallowNodeByXPath.php

        DisallowFlashFullScreen.php
    Helpers
        RegexpParser.php
        ContextSafeness.php
    RulesGenerators
        ManageParagraphs.php
        BlockElementsCloseFormattingElements.php
    RendererGenerators
        Unformatted.php
        XSLT
            Optimizer.php
        XSLT.php
    Bundles
        Forum.php
        MediaPack.php
        Fatdown.php
    Exceptions
        InvalidXslException.php
        UnsafeTemplateException.php
        InvalidTemplateException.php
Utils.php
Bundles
    Forum.php
    Fatdown
        Renderer.php
    MediaPack
        Renderer.php
    MediaPack.php
    Fatdown.php
    Forum
        Renderer.php
Plugins
    MediaEmbed
        Parser
            tagFilter.js
            hasNonDefaultAttribute.js
        Parser.js
        Configurator.php
        Parser.php
        Configurator
            Collections
                CachedDefinitionCollection.php
                SiteDefinitionCollection.php
                XmlFileDefinitionCollection.php
                SiteCollection.php

        - **TemplateGenerators**
            - Iframe.php
            - Choose.php
            - Flash.php
        - TemplateGenerator.php
        - TemplateBuilder.php
- **Autoimage**
    - Parser.js
    - Configurator.php
    - Parser.php
- **Keywords**
    - Parser.js
    - Configurator.php
    - Parser.php
- ParserBase.php
- **HTMLElements**
    - Parser.js
    - Configurator.php
    - Parser.php
- **Autovideo**
    - Parser.js
    - Configurator.php
    - Parser.php
- **Censor**
    - Parser.js
    - Configurator.php
    - Parser.php
    - Helper.php
- **HTMLEntities**
    - Parser.js
    - Configurator.php
    - Parser.php
- **BBCodes**
    - Parser.js
    - Configurator.php
    - Parser.php
    - **Configurator**
        - RepositoryCollection.php
        - AttributeValueCollection.php
        - BBCode.php
        - BBCodeMonkey.php
        - repository.xml
        - Repository.php
        - BBCodeCollection.php
- **Preg**

Parser.js
Configurator.php
Parser.php
Emoji
Parser.js
Configurator.php
Parser.php
Autoemail
Parser.js
Configurator.php
Parser.php
Escaper
Parser.js
Configurator.php
Parser.php
FancyPants
Parser.js
Configurator.php
Parser.php
HTMLComments
Parser.js
Configurator.php
Parser.php
Autolink
Parser.js
Configurator.php
Parser.php
PipeTables
Parser.js
Configurator.php
Parser.php
Emoticons
Parser.js
Configurator.php
Parser.php
Configurator
EmoticonCollection.php
ConfiguratorBase.php
Litedown
Parser.js
Configurator.php
Parser.php
LICENSE
guzzlehttp
ringphp

    - composer.json
    - **src**
        - **Future**
            - FutureInterface.php
            - BaseFutureTrait.php
            - FutureValue.php
            - FutureArray.php
            - FutureArrayInterface.php
            - CompletedFutureValue.php
            - CompletedFutureArray.php
            - MagicFutureTrait.php
        - **Exception**
            - CancelledException.php
            - CancelledFutureAccessException.php
            - ConnectException.php
            - RingException.php
        - Core.php
        - **Client**
            - Middleware.php
            - MockHandler.php
            - ClientUtils.php
            - StreamHandler.php
            - CurlMultiHandler.php
            - CurlFactory.php
            - CurlHandler.php
    - LICENSE
- **streams**
    - composer.json
    - **src**
        - AppendStream.php
        - AsyncReadStream.php
        - Stream.php
        - InflateStream.php
        - PumpStream.php
        - FnStream.php
        - StreamDecoratorTrait.php
        - **Exception**
            - CannotAttachException.php
            - SeekException.php
        - NoSeekStream.php
        - DroppingStream.php
        - LazyOpenStream.php
        - NullStream.php
        - GuzzleStreamWrapper.php
        - StreamInterface.php

Utils.php
BufferStream.php
CachingStream.php
MetadataStreamInterface.php
LimitStream.php
LICENSE
guzzle
composer.json
src
Query.php
Message
Request.php
AppliesHeadersInterface.php
MessageInterface.php
ResponseInterface.php
FutureResponse.php
Response.php
MessageFactoryInterface.php
MessageParser.php
AbstractMessage.php
MessageFactory.php
RequestInterface.php
Event
EmitterInterface.php
AbstractRequestEvent.php
ListenerAttacherTrait.php
AbstractTransferEvent.php
EventInterface.php
ErrorEvent.php
AbstractRetryableEvent.php
SubscriberInterface.php
BeforeEvent.php
EndEvent.php
Emitter.php
CompleteEvent.php
HasEmitterInterface.php
ProgressEvent.php
RequestEvents.php
HasEmitterTrait.php
AbstractEvent.php
HasDataTrait.php
Exception
ServerException.php
TransferException.php
RequestException.php

StateException.php
ClientException.php
CouldNotRewindStreamException.php
TooManyRedirectsException.php
ParseException.php
XmlParseException.php
ConnectException.php
BadResponseException.php

Cookie

CookieJar.php
SessionCookieJar.php
SetCookie.php
FileCookieJar.php
CookieJarInterface.php

QueryParser.php
RingBridge.php
Client.php
Pool.php

Subscriber

Prepare.php
Redirect.php
Mock.php
Cookie.php
HttpError.php
History.php

RequestFsm.php
UriTemplate.php
ToArrayInterface.php

Post

PostBody.php
PostBodyInterface.php
PostFile.php
MultipartBody.php
PostFileInterface.php

Transaction.php
Url.php
BatchResults.php
Utils.php
Collection.php
Mimetypes.php
ClientInterface.php

LICENSE

composer

ClassLoader.php
autoload_psr4.php

- autoload_real.php
- autoload_files.php
- autoload_namespaces.php
- autoload_classmap.php
- LICENSE
- installed.json

symfony

- **filesystem**
  - **Exception**
    - ExceptionInterface.php
    - IOException.php
    - FileNotFoundException.php
    - IOExceptionInterface.php
  - Filesystem.php
  - LockHandler.php
  - composer.json
  - LICENSE
- **debug**
  - ExceptionHandler.php
  - ErrorHandler.php
  - **Exception**
    - FlattenException.php
    - ClassNotFoundException.php
    - FatalThrowableError.php
    - ContextErrorException.php
    - DummyException.php
    - UndefinedFunctionException.php
    - UndefinedMethodException.php
    - FatalErrorException.php
    - OutOfMemoryException.php
  - DebugClassLoader.php
  - BufferingLogger.php
  - Debug.php
  - **Resources**
    - **ext**
      - php_symfony_debug.h
      - config.w32
      - README.md
      - symfony_debug.c
      - **tests**
        - 003.phpt
        - 002_1.phpt
        - 002.phpt
        - 001.phpt
      - config.m4

FatalErrorHandler
ClassNotFoundFatalErrorHandler.php
FatalErrorHandlerInterface.php
UndefinedMethodFatalErrorHandler.php
UndefinedFunctionFatalErrorHandler.php
composer.json
LICENSE
config
ResourceCheckerInterface.php
Exception
FileLoaderLoadException.php
FileLoaderImportCircularReferenceException.php
Loader
DelegatingLoader.php
FileLoader.php
LoaderResolver.php
LoaderInterface.php
Loader.php
LoaderResolverInterface.php
ConfigCacheFactoryInterface.php
FileLocatorInterface.php
FileLocator.php
ResourceCheckerConfigCache.php
Util
XmlUtils.php
ConfigCacheFactory.php
Resource
FileExistenceResource.php
ResourceInterface.php
SelfCheckingResourceChecker.php
FileResource.php
SelfCheckingResourceInterface.php
BCResourceInterfaceChecker.php
DirectoryResource.php
composer.json
ResourceCheckerConfigCacheFactory.php
ConfigCacheInterface.php
Definition
VariableNode.php
PrototypedArrayNode.php
PrototypeNodeInterface.php
ReferenceDumper.php
Exception
InvalidConfigurationException.php
ForbiddenOverwriteException.php

DuplicateKeyException.php
Exception.php
InvalidDefinitionException.php
UnsetKeyException.php
InvalidTypeException.php
ScalarNode.php
Processor.php
FloatNode.php
BaseNode.php
ArrayNode.php
Dumper
YamlReferenceDumper.php
XmlReferenceDumper.php
ConfigurationInterface.php
NumericNode.php
NodeInterface.php
IntegerNode.php
BooleanNode.php
Builder
NodeDefinition.php
ParentNodeDefinitionInterface.php
ExprBuilder.php
MergeBuilder.php
BooleanNodeDefinition.php
ValidationBuilder.php
NumericNodeDefinition.php
IntegerNodeDefinition.php
ArrayNodeDefinition.php
TreeBuilder.php
FloatNodeDefinition.php
NodeBuilder.php
NormalizationBuilder.php
ScalarNodeDefinition.php
EnumNodeDefinition.php
NodeParentInterface.php
VariableNodeDefinition.php
EnumNode.php
LICENSE
ConfigCache.php
http-foundation
Request.php
JsonResponse.php
RequestMatcher.php
BinaryFileResponse.php
Exception

- ConflictingHeadersException.php

AcceptHeader.php
IpUtils.php
RedirectResponse.php
RequestMatcherInterface.php
FileBag.php
Cookie.php
ApacheRequest.php
StreamedResponse.php
Session
- Session.php
- **Flash**
  - AutoExpireFlashBag.php
  - FlashBagInterface.php
  - FlashBag.php
- SessionBagInterface.php
- SessionInterface.php
- **Attribute**
  - AttributeBag.php
  - AttributeBagInterface.php
  - NamespacedAttributeBag.php
- **Storage**
  - **Handler**
    - PdoSessionHandler.php
    - NativeFileSessionHandler.php
    - MongoDbSessionHandler.php
    - LegacyPdoSessionHandler.php
    - WriteCheckSessionHandler.php
    - MemcachedSessionHandler.php
    - MemcacheSessionHandler.php
    - NullSessionHandler.php
    - NativeSessionHandler.php
  - MetadataBag.php
  - NativeSessionStorage.php
  - MockFileSessionStorage.php
  - PhpBridgeSessionStorage.php
  - SessionStorageInterface.php
  - MockArraySessionStorage.php
  - **Proxy**
    - AbstractProxy.php
    - NativeProxy.php
    - SessionHandlerProxy.php

File
- File.php
- **Exception**

```
      UploadException.php
      UnexpectedTypeException.php
      AccessDeniedException.php
      FileException.php
      FileNotFoundException.php
    MimeType
      ExtensionGuesser.php
      MimeTypeExtensionGuesser.php
      MimeTypeGuesser.php
      FileBinaryMimeTypeGuesser.php
      ExtensionGuesserInterface.php
      FileinfoMimeTypeGuesser.php
      MimeTypeGuesserInterface.php
    UploadedFile.php
  composer.json
  ResponseHeaderBag.php
  AcceptHeaderItem.php
  Response.php
  RequestStack.php
  HeaderBag.php
  ServerBag.php
  LICENSE
  ExpressionRequestMatcher.php
  ParameterBag.php
routing
  RouteCompiler.php
  RequestContextAwareInterface.php
  Exception
    MethodNotAllowedException.php
    ResourceNotFoundException.php
    RouteNotFoundException.php
    MissingMandatoryParametersException.php
    ExceptionInterface.php
    InvalidParameterException.php
  CompiledRoute.php
  Loader
    AnnotationClassLoader.php
    ClosureLoader.php
    schema
      routing
        routing-1.0.xsd
    AnnotationDirectoryLoader.php
    ObjectRouteLoader.php
    AnnotationFileLoader.php
    PhpFileLoader.php
```

YamlFileLoader.php
DependencyInjection
ServiceRouterLoader.php
XmlFileLoader.php
DirectoryLoader.php
RouterInterface.php
RouteCompilerInterface.php
RouteCollectionBuilder.php
RouteCollection.php
RequestContext.php
Matcher
RedirectableUrlMatcherInterface.php
RedirectableUrlMatcher.php
RequestMatcherInterface.php
Dumper
DumperRoute.php
DumperCollection.php
MatcherDumper.php
DumperPrefixCollection.php
MatcherDumperInterface.php
ApacheMatcherDumper.php
PhpMatcherDumper.php
ApacheUrlMatcher.php
TraceableUrlMatcher.php
UrlMatcherInterface.php
UrlMatcher.php
Generator
UrlGeneratorInterface.php
ConfigurableRequirementsInterface.php
Dumper
GeneratorDumper.php
GeneratorDumperInterface.php
PhpGeneratorDumper.php
UrlGenerator.php
composer.json
Route.php
LICENSE
Router.php
Annotation
Route.php
dependency-injection
ExpressionLanguageProvider.php
ContainerAwareTrait.php
ContainerBuilder.php
ExpressionLanguage.php

Variable.php
Alias.php
DefinitionDecorator.php
Exception
  ScopeWideningInjectionException.php
  BadMethodCallException.php
  ParameterCircularReferenceException.php
  ScopeCrossingInjectionException.php
  ServiceCircularReferenceException.php
  RuntimeException.php
  ParameterNotFoundException.php
  LogicException.php
  ExceptionInterface.php
  InactiveScopeException.php
  OutOfBoundsException.php
  ServiceNotFoundException.php
  InvalidArgumentException.php
Parameter.php
Loader
  ClosureLoader.php
  FileLoader.php
  schema
    dic
      services
        services-1.0.xsd
  PhpFileLoader.php
  YamlFileLoader.php
  IniFileLoader.php
  XmlFileLoader.php
  DirectoryLoader.php
Definition.php
Extension
  PrependExtensionInterface.php
  ExtensionInterface.php
  Extension.php
  ConfigurationExtensionInterface.php
ScopeInterface.php
Dumper
  YamlDumper.php
  XmlDumper.php
  GraphvizDumper.php
  Dumper.php
  PhpDumper.php
  DumperInterface.php
ContainerAware.php

TaggedContainerInterface.php
IntrospectableContainerInterface.php
LazyProxy
  Instantiator
    InstantiatorInterface.php
    RealServiceInstantiator.php
  PhpDumper
    NullDumper.php
    DumperInterface.php
ParameterBag
  ParameterBagInterface.php
  FrozenParameterBag.php
  ParameterBag.php
Scope.php
SimpleXMLElement.php
composer.json
LICENSE
ResettableContainerInterface.php
Container.php
Reference.php
Compiler
  CheckExceptionOnInvalidReferenceBehaviorPass.php
  ServiceReferenceGraphEdge.php
  DecoratorServicePass.php
  CheckDefinitionValidityPass.php
  ResolveDefinitionTemplatesPass.php
  AutowirePass.php
  RemoveUnusedDefinitionsPass.php
  ResolveReferencesToAliasesPass.php
  AutoAliasServicePass.php
  PassConfig.php
  ReplaceAliasByActualDefinitionPass.php
  CompilerPassInterface.php
  RepeatedPass.php
  Compiler.php
  ServiceReferenceGraphNode.php
  RemovePrivateAliasesPass.php
  InlineServiceDefinitionsPass.php
  MergeExtensionConfigurationPass.php
  CheckCircularReferencesPass.php
  LoggingFormatter.php
  ResolveParameterPlaceHoldersPass.php
  RemoveAbstractDefinitionsPass.php
  RepeatablePassInterface.php
  ResolveInvalidReferencesPass.php

        CheckReferenceValidityPass.php
        ExtensionCompilerPass.php
        AnalyzeServiceReferencesPass.php
        ServiceReferenceGraph.php
    ContainerAwareInterface.php
    ContainerInterface.php
polyfill-mbstring
    README.md
    Resources
        unidata
            upperCase.php
            lowerCase.php
    composer.json
    LICENSE
    bootstrap.php
    Mbstring.php
http-kernel
    Profiler
        SqliteProfilerStorage.php
        Profiler.php
        MysqlProfilerStorage.php
        BaseMemcacheProfilerStorage.php
        RedisProfilerStorage.php
        Profile.php
        PdoProfilerStorage.php
        MemcachedProfilerStorage.php
        ProfilerStorageInterface.php
        FileProfilerStorage.php
        MongoDbProfilerStorage.php
        MemcacheProfilerStorage.php
    TerminableInterface.php
    Event
        GetResponseEvent.php
        GetResponseForExceptionEvent.php
        FinishRequestEvent.php
        KernelEvent.php
        FilterControllerEvent.php
        FilterResponseEvent.php
        PostResponseEvent.php
        GetResponseForControllerResultEvent.php
    Exception
        FlattenException.php
        GoneHttpException.php
        PreconditionRequiredHttpException.php
        HttpException.php

UnprocessableEntityHttpException.php
HttpExceptionInterface.php
ConflictHttpException.php
NotFoundHttpException.php
UnauthorizedHttpException.php
NotAcceptableHttpException.php
AccessDeniedHttpException.php
BadRequestHttpException.php
LengthRequiredHttpException.php
TooManyRequestsHttpException.php
FatalErrorException.php
PreconditionFailedHttpException.php
UnsupportedMediaTypeHttpException.php
MethodNotAllowedHttpException.php
ServiceUnavailableHttpException.php

Bundle

Bundle.php
BundleInterface.php

Config

FileLocator.php
EnvParametersResource.php

Client.php

Debug

ExceptionHandler.php
ErrorHandler.php
TraceableEventDispatcher.php

CacheWarmer

CacheWarmer.php
CacheWarmerInterface.php
WarmableInterface.php
CacheWarmerAggregate.php

DataCollector

RequestDataCollector.php
MemoryDataCollector.php
RouterDataCollector.php
DataCollector.php
LateDataCollectorInterface.php
DumpDataCollector.php
ConfigDataCollector.php
DataCollectorInterface.php
LoggerDataCollector.php

Util

ValueExporter.php

AjaxDataCollector.php
EventDataCollector.php

- TimeDataCollector.php
- ExceptionDataCollector.php

HttpCache

- StoreInterface.php
- ResponseCacheStrategyInterface.php
- EsiResponseCacheStrategy.php
- ResponseCacheStrategy.php
- SurrogateInterface.php
- Esi.php
- Store.php
- HttpCache.php
- Ssi.php
- EsiResponseCacheStrategyInterface.php

HttpKernel.php

CacheClearer

- ChainCacheClearer.php
- CacheClearerInterface.php

KernelEvents.php

composer.json

HttpKernelInterface.php

UriSigner.php

Fragment

- EsiFragmentRenderer.php
- RoutableFragmentRenderer.php
- AbstractSurrogateFragmentRenderer.php
- SsiFragmentRenderer.php
- FragmentRendererInterface.php
- HIncludeFragmentRenderer.php
- InlineFragmentRenderer.php
- FragmentHandler.php

LICENSE

Log

- LoggerInterface.php
- DebugLoggerInterface.php
- NullLogger.php

EventListener

- TestSessionListener.php
- SurrogateListener.php
- ExceptionListener.php
- StreamedResponseListener.php
- DebugHandlersListener.php
- SaveSessionListener.php
- TranslatorListener.php
- SessionListener.php
- FragmentListener.php

        - ErrorsLoggerListener.php
        - ResponseListener.php
        - DumpListener.php
        - ProfilerListener.php
        - RouterListener.php
        - AddRequestFormatsListener.php
        - LocaleListener.php
        - EsiListener.php
        - ValidateRequestListener.php
    - **Controller**
        - TraceableControllerResolver.php
        - ControllerReference.php
        - ControllerResolverInterface.php
        - ControllerResolver.php
    - KernelInterface.php
    - Kernel.php
    - **DependencyInjection**
        - RegisterListenersPass.php
        - LazyLoadingFragmentHandler.php
        - FragmentRendererPass.php
        - AddClassesToCachePass.php
        - MergeExtensionConfigurationPass.php
        - Extension.php
        - ConfigurableExtension.php
        - ContainerAwareHttpKernel.php
- **event-dispatcher**
    - EventDispatcherInterface.php
    - Event.php
    - **Debug**
        - WrappedListener.php
        - TraceableEventDispatcher.php
        - TraceableEventDispatcherInterface.php
    - EventDispatcher.php
    - GenericEvent.php
    - ContainerAwareEventDispatcher.php
    - composer.json
    - EventSubscriberInterface.php
    - LICENSE
    - ImmutableEventDispatcher.php
    - **DependencyInjection**
        - RegisterListenersPass.php
- **polyfill-php55**
    - Php55ArrayColumn.php
    - README.md
    - composer.json

Php55.php
LICENSE
bootstrap.php
yaml
Inline.php
Exception
DumpException.php
RuntimeException.php
ParseException.php
ExceptionInterface.php
Unescaper.php
Escaper.php
Parser.php
Yaml.php
composer.json
Dumper.php
LICENSE
polyfill-php54
README.md
Php54.php
Resources
stubs
RecursiveCallbackFilterIterator.php
CallbackFilterIterator.php
SessionHandlerInterface.php
composer.json
LICENSE
bootstrap.php
console
Shell.php
Style
StyleInterface.php
SymfonyStyle.php
OutputStyle.php
Application.php
Event
ConsoleTerminateEvent.php
ConsoleExceptionEvent.php
ConsoleCommandEvent.php
ConsoleEvent.php
Exception
InvalidOptionException.php
CommandNotFoundException.php
RuntimeException.php
LogicException.php

- ExceptionInterface.php
- InvalidArgumentException.php

Helper

- QuestionHelper.php
- TableCell.php
- TableStyle.php
- DescriptorHelper.php
- DebugFormatterHelper.php
- TableSeparator.php
- DialogHelper.php
- Table.php
- ProgressIndicator.php
- SymfonyQuestionHelper.php
- FormatterHelper.php
- ProcessHelper.php
- ProgressHelper.php
- ProgressBar.php
- HelperInterface.php
- HelperSet.php
- TableHelper.php
- Helper.php
- InputAwareHelper.php

Descriptor

- JsonDescriptor.php
- MarkdownDescriptor.php
- XmlDescriptor.php
- Descriptor.php
- DescriptorInterface.php
- TextDescriptor.php
- ApplicationDescription.php

Formatter

- OutputFormatterStyleInterface.php
- OutputFormatter.php
- OutputFormatterInterface.php
- OutputFormatterStyleStack.php
- OutputFormatterStyle.php

Logger

- ConsoleLogger.php

Input

- InputArgument.php
- StringInput.php
- InputInterface.php
- ArgvInput.php
- InputDefinition.php
- InputOption.php

    - InputAwareInterface.php
    - ArrayInput.php
    - Input.php
  - **Question**
    - Question.php
    - ConfirmationQuestion.php
    - ChoiceQuestion.php
  - **Resources**
    - **bin**
      - hiddeninput.exe
  - composer.json
  - LICENSE
  - **Command**
    - Command.php
    - ListCommand.php
    - HelpCommand.php
  - **Tester**
    - ApplicationTester.php
    - CommandTester.php
  - **Output**
    - ConsoleOutput.php
    - ConsoleOutputInterface.php
    - StreamOutput.php
    - BufferedOutput.php
    - NullOutput.php
    - OutputInterface.php
    - Output.php
  - ConsoleEvents.php
- **finder**
  - Finder.php
  - **Adapter**
    - GnuFindAdapter.php
    - BsdFindAdapter.php
    - AbstractAdapter.php
    - AdapterInterface.php
    - AbstractFindAdapter.php
    - PhpAdapter.php
  - **Comparator**
    - DateComparator.php
    - Comparator.php
    - NumberComparator.php
  - Glob.php
  - **Exception**
    - AccessDeniedException.php
    - ShellCommandFailureException.php

    - OperationNotPermitedException.php
    - AdapterFailureException.php
    - ExceptionInterface.php
  - **Shell**
    - Shell.php
    - Command.php
  - **Iterator**
    - MultiplePcreFilterIterator.php
    - FilecontentFilterIterator.php
    - SizeRangeFilterIterator.php
    - DepthRangeFilterIterator.php
    - FileTypeFilterIterator.php
    - FilenameFilterIterator.php
    - DateRangeFilterIterator.php
    - FilePathsIterator.php
    - ExcludeDirectoryFilterIterator.php
    - RecursiveDirectoryIterator.php
    - FilterIterator.php
    - CustomFilterIterator.php
    - SortableIterator.php
    - PathFilterIterator.php
  - composer.json
  - **Expression**
    - ValueInterface.php
    - Glob.php
    - Regex.php
    - Expression.php
  - LICENSE
  - SplFileInfo.php
- **twig-bridge**
  - **Form**
    - TwigRendererEngine.php
    - TwigRendererEngineInterface.php
    - TwigRendererInterface.php
    - TwigRenderer.php
  - **Node**
    - TransDefaultDomainNode.php
    - FormThemeNode.php
    - SearchAndRenderBlockNode.php
    - StopwatchNode.php
    - TransNode.php
    - RenderBlockNode.php
    - DumpNode.php
    - FormEnctypeNode.php
  - TwigEngine.php

- AppVariable.php
- **DataCollector**
  - TwigDataCollector.php
- **Extension**
  - AssetExtension.php
  - CodeExtension.php
  - ProfilerExtension.php
  - DumpExtension.php
  - SecurityExtension.php
  - HttpKernelExtension.php
  - HttpFoundationExtension.php
  - LogoutUrlExtension.php
  - FormExtension.php
  - TranslationExtension.php
  - StopwatchExtension.php
  - YamlExtension.php
  - ExpressionExtension.php
  - RoutingExtension.php
- **Resources**
  - **views**
    - **Form**
      - bootstrap_3_horizontal_layout.html.twig
      - form_table_layout.html.twig
      - bootstrap_3_layout.html.twig
      - form_div_layout.html.twig
      - foundation_5_layout.html.twig
- **TokenParser**
  - TransTokenParser.php
  - StopwatchTokenParser.php
  - DumpTokenParser.php
  - TransChoiceTokenParser.php
  - TransDefaultDomainTokenParser.php
  - FormThemeTokenParser.php
- composer.json
- LICENSE
- **NodeVisitor**
  - TranslationDefaultDomainNodeVisitor.php
  - TranslationNodeVisitor.php
  - Scope.php
- **Command**
  - DebugCommand.php
  - LintCommand.php
- **Translation**
  - TwigExtractor.php

proxy-manager-bridge

- **LazyProxy**
  - **Instantiator**
    - RuntimeInstantiator.php
  - **PhpDumper**
    - ProxyDumper.php
- composer.json
- LICENSE

patchwork

- **utf8**
  - .gitignore
  - LICENSE-APACHE
  - appveyor.yml
  - composer.json
  - LICENSE-GPL
  - **src**
    - **Patchwork**
      - Utf8.php
      - TurkishUtf8.php
      - **PHP**
        - **Shim**
          - **charset**
            - to.zdingbat.ser
            - from.iso-8859-4.ser
            - from.iso-8859-13.ser
            - from.cp866.ser
            - from.cp949.ser
            - to.gsm0338.ser
            - from.x-mac-icelandic.ser
            - from.turkish.ser
            - from.windows-1253.ser
            - from.iso-8859-16.ser
            - from.x-mac-roman.ser
            - from.cp875.ser
            - from.iso-8859-14.ser
            - from.zdingbat.ser
            - from.iso-8859-10.ser
            - from.iso-8859-1.ser
            - from.iso-8859-5.ser
            - to.mazovia.ser
            - from.x-mac-greek.ser
            - from.windows-1252.ser
            - from.iso-8859-3.ser
            - from.cp1026.ser
            - from.windows-1254.ser
            - from.cp860.ser

from.big5.ser
from.cp950.ser
from.koi8-u.ser
from.windows-1257.ser
from.x-mac-cyrillic.ser
from.cp932.ser
from.cp037.ser
from.iso-8859-8.ser
from.cp852.ser
from.iso-8859-7.ser
from.iso-8859-6.ser
from.iso-8859-11.ser
from.us-ascii.ser
from.cp862.ser
from.mazovia.ser
from.cp1006.ser
from.cp437.ser
from.us-ascii-quotes.ser
from.cp865.ser
from.cp869.ser
from.cp500.ser
from.iso-8859-9.ser
from.cp874.ser
from.cp861.ser
from.windows-1255.ser
from.nextstep.ser
to.symbol.ser
from.cp855.ser
from.x-mac-ce.ser
from.cp936.ser
from.windows-1258.ser
from.cp864.ser
from.koi8-r.ser
translit.ser
from.cp850.ser
from.cp856.ser
from.windows-1256.ser
from.windows-1250.ser
from.cp737.ser
to.stdenc.ser
from.iso-8859-2.ser
from.cp863.ser
from.symbol.ser
from.cp424.ser
from.stdenc.ser

from.cp775.ser
from.windows-1251.ser
from.iso-8859-15.ser
from.cp857.ser
from.gsm0338.ser
unidata
canonicalComposition.ser
upperCase.ser
combiningClass.ser
lowerCase.ser
canonicalDecomposition.ser
compatibilityDecomposition.ser
Intl.php
Normalizer.php
Iconv.php
Xml.php
Mbstring.php
Utf8
Bootup.php
Bootup
mbstring.php
iconv.php
utf8_encode.php
intl.php
WindowsStreamWrapper.php
data
to.bestfit936.ser
caseFolding_full.ser
to.bestfit1251.ser
to.bestfit1253.ser
to.bestfit1258.ser
to.bestfit950.ser
translit_extra.ser
to.bestfit1257.ser
to.bestfit949.ser
to.bestfit1255.ser
to.bestfit1256.ser
to.bestfit1250.ser
to.bestfit1252.ser
to.bestfit932.ser
to.bestfit1254.ser
to.bestfit874.ser
BestFit.php
Normalizer.php
bantu

ini-get-wrapper
composer.json
src
IniGetWrapper.php
LICENSE
ocramius
proxy-manager
proxy-manager.svg
.travis.install.sh
html-docs
access-interceptor-value-holder-proxy.html
favicon.ico
img
block.png
enf.png
null-object.html
access-interceptor-scope-localizer-proxy.html
contributing.html
css
styles.css
copyright.html
download.html
production.html
virtual-proxy.html
ghost-object.html
index.html
remote-object.html
credits.html
README.md
proxy-manager.png
UPGRADE.md
tests
ProxyManagerTestAsset
ClassWithPrivateProperties.php
ClassWithByRefMagicMethods.php
ClassWithMagicMethods.php
AccessInterceptorValueHolderMock.php
ClassWithProtectedProperties.php
EmptyClass.php
ClassWithFinalMethods.php
ClassWithMethodWithDefaultParameters.php
BaseClass.php
ClassWithFinalMagicMethods.php
BaseInterface.php
CallableTypeHintClass.php

HydratedObject.php
FinalClass.php
NullObjectMock.php
ClassWithSelfHint.php
ProxyGenerator
LazyLoading
MethodGenerator
ClassWithTwoPublicProperties.php
ClassWithPublicArrayProperty.php
ClassWithMixedProperties.php
RemoteProxy
Foo.php
BazServiceInterface.php
FooServiceInterface.php
ClassWithPublicProperties.php
LazyLoadingMock.php
ClassWithAbstractProtectedMethod.php
language-feature-scripts
lazy-loading-ghost-denies-private-property-isset.phpt
access-interceptor-with-cache.phpt
access-interceptor-scope-localizer-denies-protected-property-unset.phpt
lazy-loading-value-holder-denies-protected-property-write.phpt
access-interceptor-denies-protected-property-unset.phpt
lazy-loading-value-holder-denies-private-property-write.phpt
access-interceptor-denies-protected-property-write.phpt
lazy-loading-ghost-allows-inexisting-magic-property-read.phpt
lazy-loading-ghost-denies-protected-property-unset.phpt
init.php
lazy-loading-ghost-denies-protected-property-write.phpt
lazy-loading-ghost-allows-inexisting-property-write.phpt
lazy-loading-ghost-denies-protected-property-isset.phpt
access-interceptor-scope-localizer-denies-private-property-unset.phpt
README.md
lazy-loading-ghost-denies-private-property-write.phpt
access-interceptor-denies-private-property-read.phpt
access-interceptor-scope-localizer-denies-protected-property-read.phpt
lazy-loading-value-holder-denies-protected-property-isset.phpt
access-interceptor-denies-protected-property-isset.phpt
lazy-loading-value-holder-denies-private-property-isset.phpt
lazy-loading-ghost-denies-private-property-read.phpt
access-interceptor-scope-localizer-denies-private-property-write.phpt
remote-object-json-adapter-denies-unknow-method.phpt
lazy-loading-ghost-with-cache.phpt
lazy-loading-ghost-denies-private-property-unset.phpt
lazy-loading-ghost-denies-protected-property-read.phpt

cache
README.md
null-object-public-function-empty.phpt
access-interceptor-scope-localizer-denies-private-property-isset.phpt
lazy-loading-value-holder-internal-php-classes.phpt
lazy-loading-value-holder-denies-protected-property-read.phpt
lazy-loading-value-holder-denies-private-property-unset.phpt
access-interceptor-denies-protected-property-read.phpt
lazy-loading-value-holder-with-cache.phpt
access-interceptor-denies-private-property-isset.phpt
access-interceptor-denies-private-property-write.phpt
lazy-loading-value-holder-denies-private-property-read.phpt
access-interceptor-scope-localizer-denies-protected-property-isset.phpt
access-interceptor-scope-localizer-denies-protected-property-write.phpt
lazy-loading-value-holder-denies-protected-property-unset.phpt
access-interceptor-denies-private-property-unset.phpt
lazy-loading-ghost-denies-inexisting-property-read.phpt
access-interceptor-scope-localizer-denies-private-property-read.phpt
null-object-public-property-empty.phpt
ProxyManagerTest
GeneratorStrategy
EvaluatingGeneratorStrategyTest.php
BaseGeneratorStrategyTest.php
FileWriterGeneratorStrategyTest.php
Exception
FileNotWritableExceptionTest.php
DisabledMethodExceptionTest.php
InvalidProxiedClassExceptionTest.php
UnsupportedProxiedClassExceptionTest.php
InvalidProxyDirectoryExceptionTest.php
Factory
AccessInterceptorScopeLocalizerFactoryTest.php
RemoteObjectFactoryTest.php
RemoteObject
Adapter
JsonRpcTest.php
SoapTest.php
XmlRpcTest.php
BaseAdapterTest.php
LazyLoadingValueHolderFactoryTest.php
AbstractBaseFactoryTest.php
LazyLoadingGhostFactoryTest.php
AccessInterceptorValueHolderFactoryTest.php
NullObjectFactoryTest.php
VersionTest.php

- **Inflector**
  - **Util**
    - ParameterEncoderTest.php
    - ParameterHasherTest.php
  - ClassNameInflectorTest.php
- ConfigurationTest.php
- **FileLocator**
  - FileLocatorTest.php
- **Generator**
  - ParameterGeneratorTest.php
  - MagicMethodGeneratorTest.php
  - MethodGeneratorTest.php
  - ClassGeneratorTest.php
  - **Util**
    - UniqueIdentifierGeneratorTest.php
    - ClassGeneratorUtilsTest.php
- **ProxyGenerator**
  - LazyLoadingGhostGeneratorTest.php
  - AccessInterceptorScopeLocalizerTest.php
  - **PropertyGenerator**
    - PublicPropertiesDefaultsTest.php
    - PublicPropertiesMapTest.php
    - AbstractUniquePropertyNameTest.php
  - AbstractProxyGeneratorTest.php
  - **RemoteObject**
    - **PropertyGenerator**
      - AdapterPropertyTest.php
    - **MethodGenerator**
      - RemoteObjectMethodTest.php
      - MagicSetTest.php
      - MagicIssetTest.php
      - ConstructorTest.php
      - MagicGetTest.php
      - MagicUnsetTest.php
  - LazyLoadingValueHolderGeneratorTest.php
  - **NullObject**
    - **MethodGenerator**
      - ConstructorTest.php
      - NullObjectMethodInterceptorTest.php
  - NullObjectGeneratorTest.php
  - **AccessInterceptor**
    - **PropertyGenerator**
      - MethodPrefixInterceptorsTest.php
      - MethodSuffixInterceptorsTest.php
    - **MethodGenerator**

        SetMethodPrefixInterceptorTest.php
        SetMethodSuffixInterceptorTest.php
        MagicWakeupTest.php
Assertion
    CanProxyAssertionTest.php
AccessInterceptorValueHolderTest.php
Util
    ProxiedMethodsFilterTest.php
    PublicScopeSimulatorTest.php
RemoteObjectGeneratorTest.php
AccessInterceptorScopeLocalizer
    MethodGenerator
        MagicCloneTest.php
        MagicSetTest.php
        MagicSleepTest.php
        MagicIssetTest.php
        Util
            InterceptorGeneratorTest.php
        ConstructorTest.php
        MagicGetTest.php
        InterceptedMethodTest.php
        MagicUnsetTest.php
LazyLoadingGhost
    PropertyGenerator
        InitializerPropertyTest.php
        InitializationTrackerTest.php
    MethodGenerator
        LazyLoadingMethodInterceptorTest.php
        GetProxyInitializerTest.php
        MagicCloneTest.php
        IsProxyInitializedTest.php
        MagicSetTest.php
        InitializeProxyTest.php
        MagicSleepTest.php
        MagicIssetTest.php
        MagicGetTest.php
        MagicUnsetTest.php
        CallInitializerTest.php
        SetProxyInitializerTest.php
AccessInterceptorValueHolder
    LazyLoading
        MethodGenerator
            ConstructorTest.php
    MethodGenerator
        MagicCloneTest.php

- MagicSetTest.php
- MagicIssetTest.php
- **Util**
  - InterceptorGeneratorTest.php
- ConstructorTest.php
- MagicGetTest.php
- InterceptedMethodTest.php
- MagicUnsetTest.php

LazyLoadingValueHolder

- **PropertyGenerator**
  - InitializerPropertyTest.php
  - ValueHolderPropertyTest.php
- **MethodGenerator**
  - LazyLoadingMethodInterceptorTest.php
  - GetProxyInitializerTest.php
  - MagicCloneTest.php
  - IsProxyInitializedTest.php
  - MagicSetTest.php
  - InitializeProxyTest.php
  - MagicSleepTest.php
  - MagicIssetTest.php
  - MagicGetTest.php
  - MagicUnsetTest.php
  - SetProxyInitializerTest.php

ValueHolder

- **MethodGenerator**
  - GetWrappedValueHolderValueTest.php
  - MagicSleepTest.php

Autoloader

- AutoloaderTest.php

Signature

- SignatureCheckerTest.php
- **Exception**
  - MissingSignatureExceptionTest.php
  - InvalidSignatureExceptionTest.php
- SignatureGeneratorTest.php
- ClassSignatureGeneratorTest.php

Functional

- LazyLoadingValueHolderPerformanceTest.php
- FatalPreventionFunctionalTest.php
- LazyLoadingValueHolderFunctionalTest.php
- AccessInterceptorScopeLocalizerFunctionalTest.php
- RemoteObjectFunctionalTest.php
- MultipleProxyGenerationTest.php
- LazyLoadingGhostPerformanceTest.php

LazyLoadingGhostFunctionalTest.php
AccessInterceptorValueHolderFunctionalTest.php
BaseLazyLoadingPerformanceTest.php
BasePerformanceTest.php
NullObjectFunctionalTest.php
phpdox.xml.dist
.gitignore
docs
tuning-for-production.md
access-interceptor-scope-localizer.md
remote-object.md
access-interceptor-value-holder.md
null-object.md
generator-strategies.md
lazy-loading-ghost-object.md
lazy-loading-value-holder.md
STABILITY.md
examples
virtual-proxy.php
remote-proxy.php
remote-proxy
remote-proxy-server.php
access-interceptor-scope-localizer.php
ghost-object.php
smart-reference.php
composer.json
index.html
src
ProxyManager
GeneratorStrategy
FileWriterGeneratorStrategy.php
BaseGeneratorStrategy.php
EvaluatingGeneratorStrategy.php
GeneratorStrategyInterface.php
Exception
InvalidProxyDirectoryException.php
FileNotWritableException.php
ExceptionInterface.php
InvalidProxiedClassException.php
UnsupportedProxiedClassException.php
DisabledMethodException.php
Configuration.php
Factory
NullObjectFactory.php
RemoteObject

    - **Adapter**
      - Soap.php
      - JsonRpc.php
      - XmlRpc.php
      - BaseAdapter.php
    - AdapterInterface.php
  - AbstractLazyFactory.php
  - LazyLoadingValueHolderFactory.php
  - AbstractBaseFactory.php
  - AccessInterceptorValueHolderFactory.php
  - LazyLoadingGhostFactory.php
  - RemoteObjectFactory.php
  - AccessInterceptorScopeLocalizerFactory.php
- **Inflector**
  - **Util**
    - ParameterHasher.php
    - ParameterEncoder.php
  - ClassNameInflector.php
  - ClassNameInflectorInterface.php
- **FileLocator**
  - FileLocatorInterface.php
  - FileLocator.php
- **Generator**
  - ClassGenerator.php
  - MagicMethodGenerator.php
  - ParameterGenerator.php
  - **Util**
    - ClassGeneratorUtils.php
    - UniqueIdentifierGenerator.php
  - MethodGenerator.php
- **ProxyGenerator**
  - LazyLoadingGhostGenerator.php
  - ProxyGeneratorInterface.php
  - **PropertyGenerator**
    - PublicPropertiesDefaults.php
    - PublicPropertiesMap.php
  - **RemoteObject**
    - **PropertyGenerator**
      - AdapterProperty.php
    - **MethodGenerator**
      - MagicGet.php
      - MagicUnset.php
      - RemoteObjectMethod.php
      - Constructor.php
      - MagicIsset.php

MagicSet.php
LazyLoadingValueHolderGenerator.php
NullObject
MethodGenerator
Constructor.php
NullObjectMethodInterceptor.php
AccessInterceptor
PropertyGenerator
MethodSuffixInterceptors.php
MethodPrefixInterceptors.php
MethodGenerator
SetMethodSuffixInterceptor.php
SetMethodPrefixInterceptor.php
MagicWakeup.php
Assertion
CanProxyAssertion.php
LazyLoading
MethodGenerator
Constructor.php
Util
PublicScopeSimulator.php
ProxiedMethodsFilter.php
AccessInterceptorScopeLocalizer
MethodGenerator
MagicSleep.php
MagicGet.php
MagicUnset.php
Constructor.php
MagicClone.php
MagicIsset.php
Util
InterceptorGenerator.php
MagicSet.php
InterceptedMethod.php
LazyLoadingGhost
PropertyGenerator
InitializerProperty.php
InitializationTracker.php
MethodGenerator
SetProxyInitializer.php
MagicSleep.php
InitializeProxy.php
MagicGet.php
MagicUnset.php
MagicClone.php

MagicIsset.php
GetProxyInitializer.php
CallInitializer.php
MagicSet.php
IsProxyInitialized.php
LazyLoadingMethodInterceptor.php
AccessInterceptorScopeLocalizerGenerator.php
AccessInterceptorValueHolder
MethodGenerator
MagicGet.php
MagicUnset.php
Constructor.php
MagicClone.php
MagicIsset.php
Util
InterceptorGenerator.php
MagicSet.php
InterceptedMethod.php
AccessInterceptorValueHolderGenerator.php
RemoteObjectGenerator.php
LazyLoadingValueHolder
PropertyGenerator
ValueHolderProperty.php
InitializerProperty.php
MethodGenerator
SetProxyInitializer.php
MagicSleep.php
InitializeProxy.php
MagicGet.php
MagicUnset.php
MagicClone.php
MagicIsset.php
GetProxyInitializer.php
MagicSet.php
IsProxyInitialized.php
LazyLoadingMethodInterceptor.php
NullObjectGenerator.php
ValueHolder
MethodGenerator
MagicSleep.php
GetWrappedValueHolderValue.php
Autoloader
AutoloaderInterface.php
Autoloader.php
Signature

- ClassSignatureGenerator.php
- ClassSignatureGeneratorInterface.php
- **Exception**
  - MissingSignatureException.php
  - InvalidSignatureException.php
  - ExceptionInterface.php
- SignatureChecker.php
- SignatureCheckerInterface.php
- SignatureGenerator.php
- SignatureGeneratorInterface.php

Version.php

Proxy

- ProxyInterface.php
- **Exception**
  - RemoteObjectException.php
- AccessInterceptorInterface.php
- RemoteObjectInterface.php
- SmartReferenceInterface.php
- LazyLoadingInterface.php
- GhostObjectInterface.php
- NullObjectInterface.php
- FallbackValueHolderInterface.php
- ValueHolderInterface.php
- VirtualProxyInterface.php

LICENSE
.travis.yml
CONTRIBUTING.md
.scrutinizer.yml
phpmd.xml.dist
phpunit.xml.dist

google

- **recaptcha**
  - composer.json
  - **src**
    - **ReCaptcha**
      - ReCaptcha.php
      - **RequestMethod**
        - Post.php
        - SocketPost.php
        - Socket.php
        - CurlPost.php
        - Curl.php
      - RequestParameters.php
      - RequestMethod.php
      - Response.php

```
      autoload.php
    LICENSE
ircmaxell
  password-compat
    lib
      password.php
    composer.json
    LICENSE.md
    version-test.php
marc1706
  fast-image-size
    composer.lock
    lib
      Type
        TypeWbmp.php
        TypePsd.php
        TypeBmp.php
        TypeJpeg.php
        TypeIff.php
        TypeInterface.php
        TypeTif.php
        TypeIco.php
        TypeGif.php
        TypeJp2.php
        TypeBase.php
        TypePng.php
      FastImageSize.php
    composer.json
    LICENSE
autoload.php
lusitanian
  oauth
    composer.lock
    composer.json
    src
      OAuth
        ServiceFactory.php
        OAuth1
          Token
            StdOAuth1Token.php
            TokenInterface.php
          Signature
            Signature.php
            Exception
              UnsupportedHashAlgorithmException.php
```

SignatureInterface.php

Service

Tumblr.php

ServiceInterface.php

FiveHundredPx.php

AbstractService.php

Flickr.php

Redmine.php

BitBucket.php

Xing.php

Etsy.php

Twitter.php

Yahoo.php

ScoopIt.php

FitBit.php

QuickBooks.php

OAuth2

Token

StdOAuth2Token.php

TokenInterface.php

Service

Buffer.php

GitHub.php

Mailchimp.php

SoundCloud.php

RunKeeper.php

ServiceInterface.php

Exception

MissingRefreshTokenException.php

InvalidScopeException.php

InvalidAuthorizationStateException.php

InvalidAccessTypeException.php

Dailymotion.php

Yammer.php

Nest.php

Ustream.php

AbstractService.php

Mondo.php

Salesforce.php

Google.php

Pinterest.php

Amazon.php

Foursquare.php

Instagram.php

Box.php

    - Dropbox.php
    - EveOnline.php
    - DeviantArt.php
    - Hubic.php
    - Paypal.php
    - Yahoo.php
    - Strava.php
    - Reddit.php
    - ParrotFlowerPower.php
    - BattleNet.php
    - Facebook.php
    - Spotify.php
    - Deezer.php
    - Pocket.php
    - Bitly.php
    - Delicious.php
    - Vkontakte.php
    - Harvest.php
    - JawboneUP.php
    - Microsoft.php
    - Linkedin.php
    - Heroku.php
    - Vimeo.php
    - Netatmo.php
    - Bitrix24.php
- bootstrap.php
- **Common**
  - AutoLoader.php
  - **Exception**
    - Exception.php
  - **Http**
    - **Exception**
      - TokenResponseException.php
    - **Uri**
      - UriFactory.php
      - Uri.php
      - UriInterface.php
      - UriFactoryInterface.php
    - **Client**
      - AbstractClient.php
      - CurlClient.php
      - StreamClient.php
      - ClientInterface.php
  - **Token**
    - **Exception**

                - ExpiredTokenException.php
              - AbstractToken.php
              - TokenInterface.php
            - **Storage**
              - Session.php
              - Redis.php
              - **Exception**
                - TokenNotFoundException.php
                - StorageException.php
                - AuthorizationStateNotFoundException.php
              - SymfonySession.php
              - TokenStorageInterface.php
              - Memory.php
            - **Service**
              - ServiceInterface.php
              - AbstractService.php
            - **Consumer**
              - CredentialsInterface.php
              - Credentials.php
      - LICENSE
- composer.json
- **ext**
  - **phpbb**
    - **viglink**
      - composer.lock
      - **config**
        - cron.yml
        - services.yml
      - ext.php
      - **styles**
        - **all**
          - **template**
            - **event**
              - acp_help_phpbb_stats_after.html
              - overall_footer_after.html
              - acp_help_phpbb_stats_before.html
          - **theme**
            - **images**
              - VigLink_logo.png
            - viglink.css
      - **event**
        - acp_listener.php
        - listener.php
      - .gitignore
      - **language**

- **en**
  - viglink_module_acp.php
  - info_acp_viglink.php
- **migrations**
  - viglink_cron.php
  - viglink_data.php
  - viglink_ask_admin_wait.php
  - viglink_data_v2.php
  - viglink_ask_admin.php
- composer.json
- license.txt
- **adm**
  - **style**
    - acp_viglink.html
- **cron**
  - viglink.php
- **acp**
  - viglink_helper.php
  - viglink_info.php
  - viglink_module.php

index.htm

cron.php

includes

- functions_messenger.php
- **hooks**
  - index.php
- bbcode.php
- functions_compress.php
- functions_admin.php
- functions_transfer.php
- **utf**
  - utf_tools.php
  - **data**
    - search_indexer_20.php
    - search_indexer_26.php
    - search_indexer_95.php
    - search_indexer_32.php
    - search_indexer_36.php
    - search_indexer_58.php
    - search_indexer_448.php
    - search_indexer_21.php
    - recode_basic.php
    - case_fold_f.php
    - search_indexer_9.php
    - search_indexer_64.php

case_fold_s.php
search_indexer_2.php
recode_cjk.php
search_indexer_1.php
search_indexer_5.php
confusables.php
search_indexer_4.php
search_indexer_3.php
search_indexer_19.php
search_indexer_84.php
search_indexer_6.php
search_indexer_33.php
search_indexer_0.php
case_fold_c.php
search_indexer_31.php
functions_download.php
compatibility_globals.php
ucp
ucp_pm_viewmessage.php
ucp_resend.php
ucp_groups.php
ucp_zebra.php
ucp_notifications.php
info
ucp_groups.php
ucp_zebra.php
ucp_notifications.php
ucp_pm.php
ucp_auth_link.php
ucp_main.php
ucp_profile.php
ucp_attachments.php
ucp_prefs.php
ucp_confirm.php
ucp_pm.php
ucp_auth_link.php
ucp_main.php
ucp_profile.php
ucp_remind.php
ucp_register.php
ucp_login_link.php
ucp_pm_viewfolder.php
ucp_attachments.php
ucp_pm_compose.php
ucp_activate.php

ucp_pm_options.php
ucp_prefs.php
questionnaire
questionnaire.php
functions_jabber.php
functions_acp.php
.htaccess
startup.php
diff
engine.php
diff.php
renderer.php
functions_mcp.php
message_parser.php
mcp
mcp_pm_reports.php
mcp_front.php
mcp_warn.php
info
mcp_pm_reports.php
mcp_warn.php
mcp_logs.php
mcp_notes.php
mcp_reports.php
mcp_ban.php
mcp_main.php
mcp_queue.php
mcp_logs.php
mcp_post.php
mcp_topic.php
mcp_notes.php
mcp_reports.php
mcp_forum.php
mcp_ban.php
mcp_main.php
mcp_queue.php
functions_module.php
functions_privmsgs.php
functions_compatibility.php
functions_user.php
functions.php
sphinxapi.php
functions_convert.php
functions_content.php
index.htm

constants.php
functions_display.php
functions_database_helper.php
acp

- acp_words.php
- acp_reasons.php
- acp_forums.php
- acp_permissions.php
- acp_styles.php
- acp_update.php
- acp_php_info.php
- acp_bots.php
- acp_contact.php
- auth.php
- acp_icons.php
- acp_prune.php
- acp_ban.php
- **info**
  - acp_words.php
  - acp_reasons.php
  - acp_forums.php
  - acp_permissions.php
  - acp_styles.php
  - acp_update.php
  - acp_php_info.php
  - acp_bots.php
  - acp_contact.php
  - acp_icons.php
  - acp_prune.php
  - acp_ban.php
  - acp_logs.php
  - acp_captcha.php
  - acp_inactive.php
  - acp_jabber.php
  - acp_permission_roles.php
  - acp_modules.php
  - acp_attachments.php
  - acp_ranks.php
  - acp_disallow.php
  - acp_bbcodes.php
  - acp_language.php
  - acp_database.php
  - acp_users.php
  - acp_search.php
  - acp_help_phpbb.php

            acp_email.php
            acp_profile.php
            acp_extensions.php
            acp_board.php
            acp_groups.php
            acp_main.php
        acp_logs.php
        acp_captcha.php
        acp_inactive.php
        acp_jabber.php
        acp_permission_roles.php
        acp_modules.php
        acp_attachments.php
        acp_ranks.php
        acp_disallow.php
        acp_bbcodes.php
        acp_language.php
        acp_database.php
        acp_users.php
        acp_search.php
        acp_help_phpbb.php
        acp_email.php
        acp_profile.php
        acp_extensions.php
        acp_board.php
        acp_groups.php
        acp_main.php
    functions_posting.php
app.php
files
    .htaccess
    index.htm
faq.php
common.php
adm
    images
        icon_folder_lock.gif
        bg_header.jpg
        bg_hash2.gif
        no_image.png
        bg_hash3.gif
        loading.gif
        file_new.gif
        progress_bar.gif
        cellpic3.gif

icon_delete.gif
spacer.gif
file_not_modified.gif
icon_subfolder.gif
icon_up.gif
icon_edit_disabled.gif
bg_hash1.gif
no_avatar.gif
bg_hash4.gif
arrow_up.gif
icon_folder_link.gif
icon_trace.gif
arrow_right.gif
icon_up_disabled.gif
icon_delete_disabled.gif
arrow_left.gif
gradient2b.gif
alert_close.png
bg_button.gif
bg_tabs_alt1.gif
file_new_conflict.gif
icon_down_disabled.gif
file_up_to_date.gif
icon_folder.gif
arrow_down.gif
icon_sync.gif
file_conflict.gif
bg_tabs_alt2.gif
icon_edit.gif
phpbb_logo.png
bg_header.gif
icon_sync_disabled.gif
icon_down.gif
file_modified.gif
innerbox_bg.gif

style

captcha_gd_acp.html
acp_users_feedback.html
installer_update.html
permissions.js
simple_body.html
acp_language.html
acp_bots.html
acp_prune_users.html
permission_trace.html

acp_reasons.html
acp_jabber.html
acp_ext_delete_data.html
acp_avatar_options_gravatar.html
permission_roles_mask.html
acp_search.html
installer_header.html
acp_styles.html
installer_convert.html
acp_words.html
acp_attachments.html
acp_logs.html
installer_main.html
acp_icons.html
acp_ban.html
ajax.js
acp_profile.html
admin.js
permission_mask.html
acp_users_prefs.html
captcha_recaptcha.html
acp_avatar_options_remote.html
acp_users_avatar.html
acp_php_info.html
acp_groups_position.html
acp_database.html
acp_update.html
acp_avatar_options_local.html
captcha_default_acp_demo.html
progress_bar.html
overall_header.html
acp_ext_list.html
acp_forums_copy_perm.html
confirm_bbcode.html
acp_captcha.html
tooltip.js
acp_disallow.html
admin.css
acp_users.html
acp_board.html
acp_users_profile.html
overall_footer.html
acp_email.html
acp_users_signature.html
acp_users_overview.html

acp_contact.html
confirm_body_prune.html
acp_avatar_options_upload.html
acp_inactive.html
auth_provider_ldap.html
profilefields
string.html
int.html
url.html
text.html
bool.html
dropdown.html
date.html
acp_ext_disable.html
acp_modules.html
acp_users_warnings.html
acp_ext_enable.html
installer_footer.html
acp_forums.html
auth_provider_oauth.html
captcha_qa_acp_demo.html
acp_permission_roles.html
installer_update_file_status.html
installer_form.html
acp_posting_buttons.html
acp_groups.html
acp_permissions.html
timezone.js
installer_install.html
acp_ranks.html
acp_ext_details.html
confirm_body.html
pagination.html
permission_forum_copy.html
captcha_recaptcha_acp.html
acp_help_phpbb.html
acp_prune_forums.html
acp_bbcodes.html
acp_main.html
simple_header.html
captcha_qa_acp.html
message_body.html
timezone_option.html
simple_footer.html
index.php

viewtopic.php

images

skin2

background.jpg

down.png

face.jpg

mediaelement

play-white.png

spinner-grey.gif

controls-grey.png

play-grey.png

controls-white.png

spinner-white.gif

skin6

background.png

pe3d

bt3ah.png

pe_11.jpg

bt2.png

bolha.png

bt3bh.png

bt5ah.png

pe_03.jpg

pe_12.jpg

xicon.png

bt2h.png

bt3b.png

superior.png

bt5a.png

bt1.png

bt1h.png

pe_02.jpg

pe_08.jpg

inferior.png

bt3a.png

pe_10.jpg

bolhape.jpg

bt4a.png

base.jpg

bt4ah.png

pe_07.jpg

pe_01.jpg

pe_09.jpg

pe_04.jpg

pe_06.jpg

pe_05.jpg
saedd2.psd
flags
it.png
es.png
foot.png
br.png
de.png
tr.png
en.png
fr.png
list_icons.png
alert
success.png
notice.png
error.png
warning.png
small_icons.png
loading.gif
query.png
invert_selection.png
sound_over.png
huge_icons.png
woocommerce_icons
credit-cards
visa.png
diners.png
mastercard.png
maestro.png
jcb.png
discover.png
laser.png
amex.png
cursor.png
small_paste.png
back.png
complete.gif
bt_hover.png
update.png
bt_normal.png
up.png
avatar.png
background.png
select_all.png
skin5

background.png
adown.png
logos
logo_en_us.png
logo_pt_br.png
nosound_bt.png
file_delete.png
check.png
small_cut.png
copy.png
paste.png
portfolio_header.jpg
globe.png
pass-icon.png
folder_add.png
sound_bt.png
frames
f3.png
f1.png
f2.png
user-icon.png
small_copy.png
try.png
ocorrencias
rigidez.jpg
CoraÃ§Ã£o.gif
Rim.png
xicon.png
item2.jpg
item3.png
item5.png
item4.png
disfuncao.jpg
autonomica.jpg
item1.jpg
item6.png
dentes.jpg
item7.png
t2.png
face1.jpg
social.png
link.png
tile_icons.png
exit.png
audios

Abauar o pÃ©_Cod_39_39.1_39.2.mp3
Alongamento da planta do pÃ©_3.mp3
Alongamento da musculatura posterior da coxa_1.mp3
beijos.mp3
oioioi.mp3
zip_extract.png
refresh.png
aup.png
skin9
background.png
ruler.png
face-6.jpg
face-luciano.jpg
logo-en.png
medium_icons.png
hd.png
zip_add.png
exercicio
sorriso.png
lapis.png
toalha.png
algodao.png
levelup.png
tenis.png
right.png
igual.png
thera.png
bexiga.png
man.png
unselect_all.png
avatar0.png
back.ico
trans.png
folder.png
target.png
coin.png
file_add.png
date.png
signature.png
saedd.psd
labimph.png
avatar
texto.png
fa_02.png
fa_05.png

- fa_10.png
- fa_09.png
- fa_09.jpg
- fa_10.jpg
- fa_08.jpg
- fa_01.png
- fa_04.jpg
- fa_03.png
- fa_06.jpg
- fa_03.jpg
- fa_07.jpg
- fa_05.jpg
- fa_06.png
- fa_08.png
- fa_04.png
- fa_02.jpg
- fa_01.jpg
- fa_07.png

face-8.jpg
large_icon.png
backbt.png
bg.png
cut.png
skin11
- background.png

close.png
rename.png
lattes.png
exercicios
- emotion_smile.png
- lock.png
- ex28.png
- ex8.png
- clock.png
- emotion_amazing.png
- exercicio_01.png
- lock2.png
- emotion_question.png
- ex2.png
- btfimh.png
- ex13.png
- ex19.png
- emotion_cool.png
- emotion_sweat.png
- exercicio_03.png

tenis.png
emotion_confuse.png
saude.png
ex7.png
emotion_beaten.png
btfim.png
ex17.png
exercicio_02.png
star.png
face-7.jpg
face.png
t1.png
medal.png
icons
female_avatar.png
back.png
Help.png
cobrar.png
sair.png
male_avatar.png
folder_go.png
check.png
bug.png
foot.png
app_go.png
op5.png
database_go.png
app.png
op2.png
database.png
web.png
folder.png
web_go.png
op3.png
op6.png
trophy.png
videos_sem_reducao
Vídeos novos
Pegar objetos com os p+©s - bolinha.MOV
11.0 Alternar o apoio do 1-Ý e do 5-Ý dedos sentado- 02.MOV
11.2 Apertar os dedos contra um separador - 01.MOV
MVI_8471.MOV
Mudan+§a de dire+§+£o - teste.MOV
3.0 Alongamento da planta do p+©.MOV
Enrugar toalha com os dedos dos p+©s.MOV

Alongamento de isquitibial - 01.MOV
3.4 Movimento de parafuso em cada dedo.MOV
Pegar objeto com os p+©s - bexiga com gr+£os de feij+£o.MOV
Theraband.MOV
3.1 Massagem nos p+©s com as m+£os.MOV
7.0 Abrir e fechar o h+¡lux e o segundo dedo - 02.MOV
11.0 Alternar o apoio do 1-Ý e do 5-Ý dedos sentado - 01.MOV
11.3 Andar apertando os dedos dos p+©s contra o ch+£o - 03.MOV
11.3 Andar apertando os dedos dos p+©s contra o ch+£o - 02.MOV
11.1 Dedilhar dos dedos - do 5-Ý ao 1-Ý - em p+©.MOV
5.1 Movimentar os p+©s para frente e para tr+¡s 02.MOV
7.0 Abrir e fechar o h+¡lux e o segundo dedo - 01.MOV
11.0 Alternar o apoio dos dedos (1-Ý e 5-Ý) em p+© - 01.MOV
5.2 Escrever nomes com os p+©s.MOV
Alongamento de isquitibial - 02.MOV
5.0 Apoio com a borda medial e lateral do p+© - sentado - 02.MOV
Andar com o calcanhar.MOV
11.2 Apertar os dedos contra um separador - 02.MOV
Passo para frente e para tr+¡s.MOV
Movimentos circulares com os p+©s.MOV
Apertar a bolinha com o antep+©.MOV
Pegar objetos com os p+©s - L+¡pis.MOV
11.1 Dedilhar dos dedos - do 5-Ý ao 1-Ý.MOV
11.3 Andar apertando os dedos dos p+©s contra o ch+£o - 01.MOV
7.2 Andar dedos abertos - 01.MOV
5.1 Movimentar os p+©s para frente e para tr+¡s 03.MOV
11.1Dedilhar dos dedos - do 1-Ý ao 5-Ý - em p+©.MOV
Flex+£o dos dedos com theraband.MOV
MVI_8536.MOV
5.0 Apoio com a borda medial e lateral do p+© - sentado - 01.MOV
Pegar objeto com os p+©s - algod+£o.MOV
3.2 Entrela+§ar dos dedos - movimentos circulares.MOV
5.0 Apoio com a borda medial e lateral do p+© - em p+©.MOV
Andar para frente e para tr+¡s em linha reta com um p+© atr+¡s do outro.MOV
Apoiar em um s+Ý p+©.MOV
5.1 Movimentar os p+©s para frente e para tr+¡s 01.MOV
Bater o antep+© no ch+£o - tapping.MOV
7.1 Abrir e fechar dos dedos (do 2-Ý ao 5-Ý) - 02.MOV
3.3 Massagear a planta do p+© com a bolinha.MOV
7.2 Andar dedos abertos -02.MOV
5.3 Ponta dos p+©s alternado - sentado.MOV
MVI_8543.MOV
11.1 Dedilhar dos dedos - do 1-Ý ao 5-Ý.MOV
Apoiar-se em um s+Ý p+©, pisando num objeto inst+¡vel.MOV
Ponta dos p+©s alternado - em p+©.MOV

7.1 Abrir e fechar dos dedos (do 2-Ý ao 5-Ý) - 01.MOV
11.0 Alternar o apoio dos dedos (1-Ý e 5-Ý) em p+© - 02.MOV
Mudan+§a de dire+§+£o.MOV
Fortalecimento da musculatura lateral do p+©.MOV
Fortalecimento da musculatura medial do p+©.MOV
5.1 Movimentos circulares com os p+©s - para fora e para dentro.MOV

videos

43.mp4
41.4.mp4
11.mp4
17.mp4
1 NAO .mp4
Alternar o apoio dos dedos (1-Ý e 5-Ý) em p+© - 01.mp4
57.3.mp4
33 NAO .mp4
39.mp4
15 NAO .mp4
15 NAO v4 .mp4
37.mp4
49.mp4
21.3.mp4
7.mp4
41.mp4
41.2.mp4
53.3.mp4
53.mp4
33.mp4
21.3 - v2.mp4
13 - v2.mp4
31.mp4
3.mp4
21 NAO .mp4
29.mp4
45.mp4
41.6.mp4
Mudan+§a de dire+§+£o - teste.mp4
27.mp4
Movimentos circulares.mp4
video.mp4
69.mp4
47.mp4
audio39.mp3
51.mp4
Apoio com a borda medial e lateral do p+© - em p+©.mp4
35.mp4

67.mp4
27 NAO .mp4
55.mp4
9.mp4
59.mp4
21.mp4
15.6.mp4
Alternar o apoio dos dedos (1-Ý e 5-Ý) em p+© - 02.mp4
13.mp4
25 NAO .mp4
25.mp4
audio39.wav
Dedilhar dos dedos - do 5-Ý ao 1-Ý - em p+©.mp4
15.3.mp4
15.mp4
1.mp4
61.mp4
33 NAO v2.mp4
Dedilhar dos dedos - do 1-Ý ao 5-Ý - em p+©.mp4
63.mp4
31 NAO .mp4
5.mp4
29.3 .mp4
.htaccess
css
woocommerce
rt-woocommerce-rtl.css
rt-woocommerce.css
fonts
star.ttf
WooCommerce.woff
star.eot
WooCommerce.ttf
star.svg
WooCommerce.eot
WooCommerce.svg
star.woff
rt-woocommerce-rtl.min.css
rt-woocommerce.min.css
introjs-rtl.css
myform.css
ie9.min.css
themes
.DS_Store
dark

loading.gif
dark.css
.DS_Store
bullets.png
arrows.png
bar
loading.gif
bar.css
bullets.png
arrows.png
light
loading.gif
bullets.png
arrows.png
light.css
default
loading.gif
bullets.png
default.css
arrows.png
ie9.css
blue.css
upload.css
mejs-skin.min.css
preview-style.css
preview-style.min.css
mejs-skin.css
owl-carousel.css
bootstrap.css
login.css
owl-carousel.min.css
introjs.css
fontello
css
fontello-ie7-codes.css
fontello-ie7.css
fontello.css
fontello-embedded.css
animation.css
fontello-codes.css
LICENSE.txt
README.txt
config.json
demo.html
font

- fontello.woff
- fontello.ttf
- fontello.svg
- fontello.eot

forms.css

nivo-slider.css

layout1

- style.css
- style.min.css
- app.min.css
- rtl.min.css
- rtl.css

mytable.css

sass

- **woocommerce**
  - rt-woocommerce.scss
  - **utility**
    - _mixins.scss
  - rt-woocommerce-rtl.scss
- ie9.scss
- **partials**
  - _testimonials.scss
  - _portfolio.scss
  - _media_queries.scss
  - _social_media.scss
  - _pagination.scss
  - _global_structure.scss
  - _page_loading.scss
  - _design_elements.scss
  - _404_page.scss
  - _tables.scss
  - **layout1**
    - _widgets.scss
    - _media_queries.scss
    - _navigation.scss
    - _structure.scss
  - _animations.scss
  - _helpers.scss
  - _typography.scss
  - _forms.scss
  - _carousels.scss
  - _breadcrumb_menu.scss
  - _team.scss
  - **layout2**
    - _widgets.scss

_media_queries.scss
_navigation.scss
_structure.scss
_shortcodes.scss
_products.scss
_blog.scss

bootstrap

_type.scss
_grid.scss
_modals.scss
_progress-bars.scss
_dropdowns.scss
_navbar.scss
_theme.scss
_wells.scss
_button-groups.scss
_list-group.scss
_close.scss
_thumbnails.scss
_breadcrumbs.scss
_popovers.scss
_jumbotron.scss
_normalize.scss
_print.scss
_pagination.scss
_code.scss
_component-animations.scss
_responsive-embed.scss
_scaffolding.scss
_badges.scss
_panels.scss
_navs.scss
_media.scss
_tables.scss
_pager.scss
_carousel.scss

mixins

_grid.scss
_background-variant.scss
_tab-focus.scss
_image.scss
_resize.scss
_list-group.scss
_border-radius.scss
_reset-filter.scss

_text-emphasis.scss
_pagination.scss
_panels.scss
_hide-text.scss
_opacity.scss
_grid-framework.scss
_responsive-visibility.scss
_text-overflow.scss
_center-block.scss
_nav-vertical-align.scss
_size.scss
_progress-bar.scss
_table-row.scss
_forms.scss
_gradients.scss
_nav-divider.scss
_alerts.scss
_vendor-prefixes.scss
_buttons.scss
_labels.scss
_clearfix.scss
_variables.scss
_mixins.scss
_input-groups.scss
_tooltip.scss
_responsive-utilities.scss
_glyphicons.scss
_forms.scss
_alerts.scss
_buttons.scss
_labels.scss
_utilities.scss
bootstrap.scss
utility
_imports.scss
_layout1_vars.scss
_layout2_vars.scss
_mixins.scss
_vars.scss
layout1
rtl.scss
style.scss
layout2
rtl.scss
style.scss

bootstrap.min.css
pink.css
layout2
style.css
style.min.css
app.min.css
rtl.min.css
rtl.css
system.css
mysql
index.php
chat
sounds
sound_2.ogg
sound_2.wav
sound_6.wav
sound_6.mp3
sound_5.mp3
sound_5.ogg
sound_3.ogg
sound_7.mp3
sound_5.wav
sound_4.wav
sound_2.mp3
sound_3.wav
index.html
sound_1.ogg
sound_4.ogg
license.txt
sound_4.mp3
sound_1.wav
sound_8.mp3
sound_6.ogg
sound_1.mp3
sound_3.mp3
img
pixel.gif
buttons-sprite.png
broken-image.png
index.html
license.txt
emoticons
confused.png
wink.png
grin.png

cool.png
important.png
smile-big.png
idea.png
monkey.png
glasses.png
devilish.png
plain.png
angel.png
smile.png
error.png
crying.png
warning.png
index.html
razz.png
favorite.png
help.png
eek.png
sad.png
kiss.png
surprise.png
delete.png
loading-sprite.png
install.php
flash
FABridge.swf
index.html
css
Plum.css
vBulletin.css
black.css
XenForo.css
pine_images
chatlist.jpg
print.css
prosilver.css
grey.css
Lithium.css
Oxygen.css
Core.css
fonts.css
custom.css
beige.css
Pine.css
Uranium.css

shoutbox.css
index.html
MyBB.css
plum_images
plum.png
plum2.png
Cobalt.css
global.css
Mercury.css
Sulfur.css
socket
server
.htaccess
server.rb
server.conf
js
lang
th.js
el.js
ar.js
no.js
ro.js
kr.js
nl.js
pt-br.js
ca.js
pt-pt.js
gl.js
sr.js
fr.js
he.js
hu.js
ja.js
et.js
sl.js
es.js
uk.js
en.js
tr.js
fi.js
pl.js
index.html
it.js
cz.js
ka.js

bg.js
sk.js
mk.js
hr.js
ru.js
sv.js
in.js
zh.js
de.js
da.js
cy.js
nl-be.js
zh-tw.js
custom.js
logs.js
shoutbox.js
config.js
index.html
FABridge.js
chat.js
lib
lang
ar.php
no.php
tr.php
he.php
sk.php
ka.php
cy.php
et.php
pt-br.php
sl.php
sr.php
nl-be.php
th.php
zh-tw.php
hu.php
hr.php
fi.php
it.php
ro.php
ja.php
kr.php
in.php
cz.php

de.php
mk.php
ca.php
bg.php
nl.php
fr.php
da.php
es.php
en.php
el.php
uk.php
pt-pt.php
fa.php
sv.php
ru.php
zh.php
gl.php
pl.php

class

CustomAJAXChatInterface.php
AJAXChatEncoding.php
CustomAJAXChatShoutBox.php
CustomAJAXChat.php
AJAXChatHTTPHeader.php
AJAXChatTemplate.php
AJAXChatMySQLQuery.php
AJAXChatMySQLiDataBase.php
AJAXChatFileSystem.php
AJAXChatLanguage.php
AJAXChatDataBase.php
AJAXChatMySQLDataBase.php
AJAXChat.php
AJAXChatString.php
AJAXChatMySQLiQuery.php

classes.php
custom.php
.htaccess
config.php

template

loggedIn.html
shoutbox.html
logs.html
loggedOut.html

data

channels.php

users.php
index.php
readme.html
src
FABridge.as
index.html
EmptySwf.as
chat.sql
license.txt
changelog.txt
audio
25,1.mp3
25.mp3
31,1.mp3
29,3.mp3
13.mp3
29,1.mp3
3.mp3
45.mp3
7.wav
53.mp3
51,2.mp3
17,1.mp3
67,1.mp3
41,4.mp3
7.mp3
17,2.mp3
15,5.mp3
37.mp3
47.mp3
35,2.mp3
15,2.mp3
31.mp3
59,2.mp3
49.mp3
57,3.mp3
53,4.mp3
37,1.mp3
57,5.mp3
69,2.mp3
41,1.mp3
21,4.mp3
21,3.mp3
53,3.mp3
33,1.mp3

41,7.mp3
49,2.mp3
57,2.mp3
61,1.mp3
27.mp3
15,7.mp3
39,1.mp3
49,1.mp3
43.mp3
41,5.mp3
39,2.mp3
9.mp3
13,2.mp3
47,1.mp3
21,1.mp3
29,2.mp3
35.mp3
17.mp3
69.mp3
15,1.mp3
55,1.mp3
63,2.mp3
57.mp3
29,4.mp3
41.mp3
29.mp3
57.1.mp3
15.mp3
67.mp3
33,2.mp3
63,1.mp3
41,6.mp3
27,1.mp3
51,3.mp3
39.mp3
27,2.mp3
59.mp3
55.mp3
51.mp3
45,2.mp3
41,2.mp3
43,1.mp3
53,2.mp3
57,4.mp3
13,1.mp3

21.mp3
15,6.mp3
35,1.mp3
67,2.mp3
63.mp3
29,5.mp3
11.mp3
61,2.mp3
15,4.mp3
47,2.mp3
31,2.mp3
43,2.mp3
21,2.mp3
15,3.mp3
45,1.mp3
61.mp3
33.mp3
1.mp3
69,1.mp3
59,1.mp3
21,5.mp3
41,3.mp3
25,2.mp3
53,5.mp3
53,1.mp3

fonts

glyphicons-halflings-regular.svg
glyphicons-halflings-regular.ttf
glyphicons-halflings-regular.eot
glyphicons-halflings-regular.woff

js

us.control.js
us.forms.js

tinymce

themes

modern

theme.min.js

inlite

theme.min.js

jquery.tinymce.min.js

skins

lightgray

ImagePanel.less
TabPanel.less
Progress.less

Reset.less
Iframe.less
Icons.less
Content.Inline.less
ColorButton.less
skin.ie7.less
FitLayout.less
img
  anchor.gif
  object.gif
  trans.gif
  loader.gif
Mixins.less
FloatPanel.less
Notification.less
MenuButton.less
SplitButton.less
Animations.less
Content.Objects.less
TinyMCE.less
ComboBox.less
skin.ie7.min.css
skin.dev.less
Window.less
ResizeHandle.less
Spacer.less
ToolTip.less
skin.ie7.dev.less
Menu.less
Path.less
Sidebar.less
ButtonGroup.less
CropRect.less
skin.less
StackLayout.less
Button.less
fonts
  tinymce.ttf
  tinymce.woff
  tinymce.eot
  tinymce.svg
  tinymce-small.svg
  tinymce-small.eot
  tinymce-small.woff
  tinymce-small.ttf

- content.min.css
    - MenuBar.less
    - Label.less
    - ColorPicker.less
    - SelectBox.less
    - Icons.Ie7.less
    - Content.less
    - Variables.less
    - skin.min.css
    - Panel.less
    - Checkbox.less
    - Container.less
    - Scrollable.less
    - MenuItem.less
    - Throbber.less
    - content.inline.min.css
    - InfoBox.less
    - FieldSet.less
    - AbsoluteLayout.less
    - TextBox.less
    - Slider.less
    - ColorBox.less
    - ListBox.less
    - Arrows.less
    - Radio.less
    - FlowLayout.less
- **plugins**
  - **preview**
    - plugin.min.js
  - **table**
    - plugin.dev.js
    - plugin.min.js
  - **bbcode**
    - plugin.min.js
  - **visualblocks**
    - **css**
      - visualblocks.css
    - plugin.min.js
  - **link**
    - plugin.min.js
  - **contextmenu**
    - plugin.min.js
  - **advlist**
    - plugin.min.js
  - **autolink**

plugin.min.js

image

plugin.min.js

autosave

plugin.min.js

layer

plugin.min.js

importcss

plugin.min.js

legacyoutput

plugin.min.js

tabfocus

plugin.min.js

fullpage

plugin.min.js

anchor

plugin.min.js

searchreplace

plugin.min.js

code

plugin.min.js

visualchars

plugin.min.js

directionality

plugin.min.js

wordcount

plugin.min.js

media

plugin.min.js

lists

plugin.min.js

textcolor

plugin.min.js

codesample

plugin.dev.js

css

prism.css

plugin.min.js

charmap

plugin.min.js

hr

plugin.min.js

example

dialog.html

plugin.min.js

paste
  plugin.dev.js
  plugin.min.js
template
  plugin.min.js
fullscreen
  plugin.min.js
noneditable
  plugin.min.js
insertdatetime
  plugin.min.js
toc
  plugin.min.js
print
  plugin.min.js
pagebreak
  plugin.min.js
save
  plugin.min.js
example_dependency
  plugin.min.js
textpattern
  plugin.min.js
imagetools
  plugin.min.js
emoticons
  img
    smiley-tongue-out.gif
    smiley-surprised.gif
    smiley-sealed.gif
    smiley-innocent.gif
    smiley-cry.gif
    smiley-yell.gif
    smiley-foot-in-mouth.gif
    smiley-wink.gif
    smiley-smile.gif
    smiley-cool.gif
    smiley-laughing.gif
    smiley-embarassed.gif
    smiley-money-mouth.gif
    smiley-frown.gif
    smiley-undecided.gif
    smiley-kiss.gif
  plugin.min.js
spellchecker

plugin.dev.js
plugin.min.js
autoresize
plugin.min.js
colorpicker
plugin.min.js
nonbreaking
plugin.min.js
tinymce.min.js
license.txt
langs
pt_BR.js
readme.md
fancybox
helpers
jquery.fancybox-thumbs.css
jquery.fancybox-buttons.js
fancybox_buttons.png
jquery.fancybox-buttons.css
jquery.fancybox-thumbs.js
jquery.fancybox-media.js
fancybox_sprite.png
fancybox_overlay.png
jquery.fancybox.js
jquery.fancybox.pack.js
blank.gif
fancybox_loading@2x.gif
fancybox_loading.gif
jquery.fancybox.css
fancybox_sprite@2x.png
pixi.min.js
sideslider
jquery.side-slider.js
side-slider.css
main.js
alert
success.png
notice.png
error.png
warning.png
alert-box.css
waypoints.min.js
jquery.maskedinput.js
placeholders.min.js
comment-reply.min.js

jquery.nivo.slider.pack.js
pace.js
mediaelement-and-player.min.js
bootstrap.min.js
jflickrfeed.min.js
introducao.js
jquery.min2.js
jquery.js
jquery.nivo.slider.js
video.js
plupload
  jquery.ui.plupload
    img
      loading.gif
      plupload.png
    jquery.ui.plupload.js
    css
      jquery.ui.plupload.css
    jquery.ui.plupload.min.js
  plupload.full.min.js
  jquery.plupload.queue
    img
      done.gif
      backgrounds.gif
      error.gif
      delete.gif
      transp50.png
      throbber.gif
      buttons.png
      buttons-disabled.png
    css
      jquery.plupload.queue.css
    jquery.plupload.queue.js
    jquery.plupload.queue.min.js
  plupload.dev.js
  plupload.min.js
  i18n
    kk.js
    km.js
    el.js
    ar.js
    ro.js
    nl.js
    uk_UA.js
    ca.js

hy.js
sr.js
fr.js
ms.js
be_BY.js
he.js
hu.js
vi.js
ja.js
pt_BR.js
az.js
th_TH.js
et.js
es.js
en.js
id.js
tr.js
bs.js
fi.js
pl.js
it.js
lv.js
ko.js
ka.js
bg.js
cs.js
sk.js
sq.js
hr.js
ru.js
fa.js
sv.js
zh_TW.js
zh_CN.js
de.js
da.js
ku_IQ.js
cy.js
lt.js
moxie.min.js
Moxie.xap
moxie.js
upload.php
Moxie.swf
us.main.js

ruler_video.js
scripts.js
modernizr.min.js
customselect.min.js
lightbox
- **img**
  - video_poster.jpg
  - audio_poster.jpg
  - **graphics**
    - thumbs_show.png
    - hover_magnify.png
    - info.png
    - panel_right_over.png
    - thumb_right.png
    - thumbs_hide.png
    - fullscreen.png
    - thumb_left.png
    - ie_preloader.gif
    - panel_left_over.png
    - panel_right.png
    - right_arrow.png
    - ie_bg.png
    - hover_play.png
    - exit.png
    - normalscreen.png
    - **video**
      - fullscreen.png
      - vid_pause.png
      - vid_play.png
      - normalscreen.png
      - vid_total.png
      - preloader.gif
      - vid_progress.png
      - vid_volume.png
      - **retina**
        - vid_progress@2x.png
        - vid_play@2x.png
        - vid_volume@2x.png
        - vid_pause@2x.png
        - preloader@2x.gif
        - vid_mute@2x.png
        - normalscreen@2x.png
        - vid_total@2x.png
        - fullscreen@2x.png
      - vid_mute.png

- pixel.png
- hover_document.png
- **music**
  - ie_btn.png
  - ie_ctrl.png
- **retina**
  - panel_right_over@2x.png
  - thumbs_show@2x.png
  - info@2x.png
  - panel_left_over@2x.png
  - right_arrow@2x.png
  - normalscreen@2x.png
  - thumb_left@2x.png
  - hover_magnify@2x.png
  - thumbs_hide@2x.png
  - panel_right@2x.png
  - panel_left@2x.png
  - fullscreen@2x.png
  - hover_document@2x.png
  - left_arrow@2x.png
  - exit@2x.png
  - thumb_right@2x.png
  - hover_play@x2.png
- **social**
  - facebook.png
  - facebook_mac.png
  - twitter.png
  - google.png
  - twitter_webkit.png
  - pinterest.png
  - twitter_mac.png
- left_arrow.png
- panel_left.png

bg.png

thumbs

- default.jpg
- music.jpg

css

- jackbox-video.min.css
- jackbox-ie8.css
- jackbox.min.css
- jackbox-audio.min.css
- jackbox-ie9.css

modules

- jackbox_social.php

    jackbox_video.html
    jackbox_audio.html
    jackbox_swf.html
  js
    libs
      modernizr-2.5.3.min.js
    jackbox-effects.min.js
    jackbox-audio.min.js
    jackbox-packed.min.js
    jackbox-video.min.js
  swf
    video_fallback.swf
    audio_fallback.swf
scripts.min.js
maps.js
respond.min.js
isotope.pkgd.min.js
language
  pt_br.js
  en_us.js
sound.js
html5shiv.min.js
admin_complicacoes.js
jquery-migrate.min.js
input-mask.js
intro.js
ruler.js
remodal
  remodal-default-theme.css
  remodal.css
  remodal.js
complicacoes.js
app.min.js
owl.carousel.min.js
jquery.vide.min.js
pikaday
  moment.min.js
  pikaday.css
  pikaday.js
avatar.js
filetree
  jqueryFileTree.css
  images
    file.png
    php.png

doc.png
directory.png
java.png
ruby.png
spinner.gif
folder_open.png
html.png
zip.png
pdf.png
txt.png
flash.png
xls.png
css.png
psd.png
picture.png
ppt.png
code.png
script.png
film.png
application.png
music.png
linux.png
db.png
jquery.js
jqueryFileTree.js
jquery.easing.js
connectors
jqueryFileTree.php
test.php
select2
css
select2.custom.css
select2.css
select2.min.css
js
select2.js
select2.full.js
select2.min.js
i18n
dsb.js
nb.js
km.js
th.js
el.js
ar.js

sr-Cyrl.js
ps.js
ro.js
nl.js
ca.js
hy.js
gl.js
sr.js
fr.js
ms.js
he.js
zh-TW.js
hu.js
tk.js
zh-CN.js
vi.js
ja.js
az.js
et.js
sl.js
es.js
uk.js
en.js
id.js
pt-BR.js
tr.js
bs.js
fi.js
pl.js
it.js
lv.js
is.js
ko.js
bg.js
cs.js
sk.js
mk.js
hr.js
ru.js
fa.js
sv.js
de.js
da.js
hi.js
af.js

pt.js
eu.js
hsb.js
lt.js
select2.full.min.js
how.txt
imagesloaded.min.js
sweetalert2
sweetalert2.all.min.js
sweetalert2.min.css
index.php
ftp
server.php
images
down.png
list_icons.png
alert
success.png
notice.png
error.png
warning.png
small_icons.png
query.png
invert_selection.png
huge_icons.png
small_paste.png
back.png
update.png
up.png
select_all.png
adown.png
file_delete.png
small_cut.png
copy.png
paste.png
pass-icon.png
folder_add.png
logo.png
user-icon.png
small_copy.png
link.png
tile_icons.png
exit.png
zip_extract.png
refresh.png

aup.png
medium_icons.png
hd.png
zip_add.png
unselect_all.png
folder.png
file_add.png
large_icon.png
backbt.png
bg.png
cut.png
close.png
rename.png
icons
folder_go.png
app_go.png
database_go.png
app.png
database.png
web.png
folder.png
web_go.png
css
upload.css
login.css
forms.css
system.css
js
jquery.js
plupload
jquery.ui.plupload
img
loading.gif
plupload.png
jquery.ui.plupload.js
css
jquery.ui.plupload.css
jquery.ui.plupload.min.js
plupload.full.min.js
jquery.plupload.queue
img
done.gif
backgrounds.gif
error.gif
delete.gif

transp50.png
throbber.gif
buttons.png
buttons-disabled.png
css
jquery.plupload.queue.css
jquery.plupload.queue.js
jquery.plupload.queue.min.js
plupload.dev.js
plupload.min.js
i18n
kk.js
km.js
el.js
ar.js
ro.js
nl.js
uk_UA.js
ca.js
hy.js
sr.js
fr.js
ms.js
be_BY.js
he.js
hu.js
vi.js
ja.js
pt_BR.js
az.js
th_TH.js
et.js
es.js
en.js
id.js
tr.js
bs.js
fi.js
pl.js
it.js
lv.js
ko.js
ka.js
bg.js
cs.js

sk.js
sq.js
hr.js
ru.js
fa.js
sv.js
zh_TW.js
zh_CN.js
de.js
da.js
ku_IQ.js
cy.js
lt.js
moxie.min.js
Moxie.xap
moxie.js
upload.php
Moxie.swf
filetree
jqueryFileTree.css
images
file.png
php.png
doc.png
directory.png
java.png
ruby.png
spinner.gif
folder_open.png
html.png
zip.png
pdf.png
txt.png
flash.png
xls.png
css.png
psd.png
picture.png
ppt.png
code.png
script.png
film.png
application.png
music.png
linux.png

db.png
jquery.js
jqueryFileTree.js
jquery.easing.js
connectors
jqueryFileTree.php
test.php
index.php
php
actions.php
plugins
revslider
release_log.html
revslider.php
languages
revslider-de_DE.mo
revslider-de_DE.po
revslider-ru_RU.po
revslider-hu_HU.po
revslider.pot
revslider-hu_HU.mo
revslider-pl_PL.mo
revslider-en_US.mo
index.php
revslider-zh_CN.po
revslider-it_IT.po
revslider-pl_PL.po
revslider-it_IT.mo
revslider-ru_RU.mo
revslider-zh_CN.mo
revslider-en_US.po
public
revslider-front.class.php
index.php
assets
info.cfg
css
index.php
openhand.cur
closedhand.cur
settings.css
captions-original.css
fonts
pe-icon-7-stroke
css

- helper.css
- pe-icon-7-stroke.css
- index.php
- **fonts**
  - Pe-icon-7-stroke.ttf
  - Pe-icon-7-stroke.woff
  - Pe-icon-7-stroke.eot
  - index.php
  - Pe-icon-7-stroke.svg
- index.php

revicons

- revicons.eot
- revicons.svg
- index.php
- revicons.ttf
- revicons.woff

font-awesome

- **css**
  - font-awesome.css
  - font-awesome.min.css
  - index.php
- **fonts**
  - fontawesome-webfont.woff
  - fontawesome-webfont.svg
  - FontAwesome.otf
  - index.php
  - fontawesome-webfont.eot
  - fontawesome-webfont.ttf
- index.php

index.php

js

- jquery.themepunch.revolution.min.js
- **source**
  - jquery.themepunch.tools.min.js
  - jquery.themepunch.enablelog.js
  - index.php
  - jquery.themepunch.revolution.js
- jquery.themepunch.tools.min.js
- jquery.themepunch.enablelog.js
- index.php
- **extensions**
  - revolution.extension.navigation.min.js
  - revolution.extension.parallax.min.js
  - revolution.extension.actions.min.js
  - **source**

```
            revolution.extension.layeranimation.js
            revolution.extension.migration.js
            revolution.extension.slideanims.js
            revolution.extension.actions.js
            revolution.extension.video.js
            revolution.extension.parallax.js
            revolution.extension.carousel.js
            index.php
            revolution.extension.navigation.js
            revolution.extension.kenburn.js
          revolution.extension.kenburn.min.js
          revolution.extension.carousel.min.js
          index.php
          revolution.extension.slideanims.min.js
          revolution.extension.video.min.js
          revolution.extension.migration.min.js
          revolution.extension.layeranimation.min.js
      index.php
      assets
        gridtile_white.png
        coloredbg.png
        gridtile.png
        gridtile_3x3.png
        sources
          vm.png
          ig.png
          post.png
          yt.png
          fb.png
          tw.png
          index.php
          revolution_slide1.png
          wc.png
          revolution_slide2.png
          fr.png
        index.php
        gridtile_3x3_white.png
        loader.gif
backup
  index.php
index.php
uninstall.php
admin
  views
    slide-editor.php
```

- slider-overview.php
- slider-editor.php
- navigation-editor.php
- index.php
- **system**
  - dialog-video.php
  - validation.php
  - index.php
  - dialog-copy-move.php
  - dialog-global-settings.php
- slide-overview.php
- master-view.php
- **templates**
  - slides.php
  - create-slider.php
  - slider-main-options.php
  - slide-selector.php
  - slides-list.php
  - template-selector.php
  - edit-slider.php
  - index.php
  - sliders-list.php
  - wpml-selector.php
  - template-slider-selector.php
  - slide-stage.php
  - slide-general-settings.php

revslider-admin.class.php
index.php
assets

- **images**
  - icon-not-activated.png
  - tp_newsletter_bg.png
  - dash-plugin.png
  - slidebg.jpg
  - new_slider.png
  - macbook.png
  - gridtile_white.png
  - dash-light.png
  - i_addlayer_video.png
  - i_addlayer_text.png
  - newsletter-figure.png
  - visitstore.png
  - preview.png
  - icon_vimeo.png
  - icon-no-problem-found.png

trans_tile2.png
quickslider.png
dotted_vertical.png
icon-plus.png
i_addlayer_image.png
imac.png
dash-diamond.png
icon-text-layer.png
add_template.png
gridtile.png
dash-gift.png
gridtile_3x3.png
icon-shape-layer.png
tooltips
  progressbar.png
  slide_placeholder.png
  slide_placeholder_cut.png
  index.php
icon-button-layer.png
iphone.png
sliderpresets
  thumb_auto1.png
  flat_carousel.png
  thumbs_left_auto.png
  carousel_thumbs_right_fullscreen.png
  flat_carousel_thumbs.png
  cover_carousel_endless.png
  wide_fullscreen.png
  thumbs_right_auto.png
  cover_carousel_thumbs.png
  index.php
  flat_carousel_thumbs_left.png
  carousel_full_rounded.png
  scroll_fullscreen.png
  slideshow_auto_layout.png
dash-download.png
dash-refresh.png
dash-ticket.png
icon_youtube.png
toolbar
  icon-chooser-2-light.png
  icon-italic.png
  schematic_out.png
  icon-alignleft-dark.png
  droplet_black.png

largearrow_black.png
icon-scalex.png
icon-mode-laptop-light.png
icon-skewx.png
animate_out.png
icon-save-big.png
icon-fontsize.png
visibility_black.png
icon-close.png
schematic_in.png
icon-color.png
plus_black.png
icon-add-video.png
icon-mode-desktop-light.png
advancedcss.png
eye_black.png
icon-maxheight.png
icon-aligntop-light.png
conflict.png
icon-wrap.png
icon-add-layer.png
icon-slide-published.png
icon-splittext-delay.png
icon-mode-tablet-dark.png
icon-rotation-end.png
icon-add-duplicate.png
icon-edit_dark.png
icon-rotationz.png
icon-alignbottom-light.png
newlinear-horizontal.png
in_white.png
icon-chooser-1-dark.png
icon-aligncenterh-dark.png
icon-skewy.png
icon-parallax.png
tiled-lightgrey.png
morestyles_black.png
icon-originx.png
icon-link-light.png
icon-rev-logo-mini-2.png
tools_white.png
icon-fontfamily.png
morestyles_white.png
plus_white.png
icon-add-image.png

lighttrash.png
icon-padding.png
icon-opacity.png
icon-alignright-dark.png
toolbar-bg.png
icon-wrap-light.png
backtoidle.png
icon-borderwidth.png
icon-bg.png
lightpencil.png
icon-rotationx.png
icon-aligncenterv-dark.png
icon-aligntop-dark.png
tiled-darkgrey.png
icon-fonttemplate.png
icon-perspective.png
plus_grey.png
tools_black.png
icon-alignleft-light.png
icon-chooser-3-dark.png
icon-mode-desktop-dark.png
icon-splittext.png
icon-alignright-light.png
icon-save-light.png
icon-bordercolor.png
droplet_white.png
animate_in.png
icon-globe.png
icon-edit.png
icon-mode-phone-light.png
icon-decoration.png
icon-mode-phone-dark.png
icon-scaley.png
layers_bg_tile.png
icon-2drotation.png
index.php
icon-slide-unpublished.png
icon-borderradius.png
icon-easing.png
icon-clock.png
out_white.png
newlinear-vertical.png
icon-rotationy.png
icon-chooser-1-light.png
icon-close-big.png

icon-zoffset.png
icon-basketball2.png
icon-maxwidth.png
icon-fontweight.png
icon-borderstyle.png
icon-shortcode.png
icon-aligncenterh-light.png
icon-link.png
icon-originy.png
icon-xoffset.png
icon-rotation-start.png
arrow_white.png
icon-mode-laptop-dark.png
lightcopy.png
icon-mode-tablet-light.png
icon-align.png
tiled-red.png
icon-chooser-3-light.png
eye_white.png
newlinear.png
icon-aligncenterv-light.png
icon-save-dark.png
out_black.png
visibility_white.png
icon-rev-logo-mini.png
in_black.png
icon-lineheight.png
addlayer.png
icon-chooser-2-dark.png
icon-alignbottom-dark.png
icon-yoffset.png
icon-transition.png
arrow_grey.png
icon-delete.png
sliderselector
type_slider.png
index.php
type_hero.png
type_carousel.png
dummy.png
add_template_bg.jpg
mainoptions
tp_uparrow.png
jqueryicon.png
showimage.png

fullwidth.png
imageicon.png
fullscreen.png
openclose.png
bullets.png
thumbnails.png
cssicon.png
linktoslide.png
mini-editslide.jpg
index.php
arrows.png
mini-implement.jpg
add_preset.png
mini-customizeslide.jpg
auto.png
tabs.png
logo_small.png
rs_rotate.png
icon-edit.png
sources
vm.png
ig.png
post.png
yt.png
fb.png
tw.png
index.php
revolution_slide1.png
notice.txt
wc.png
revolution_slide2.png
fr.png
topbar.png
tp-brokenimage.png
slidersources
tp_source_face.png
tp_source_yout.png
tp_source_inst.png
tp_source_post.png
tp_source_flic.png
tp_source_twit.png
index.php
tp_source_wooc.png
tp_source_vime.png
tp_source_defa.png

- index.php
- dash-copy.png
- icon_html5.png
- icon-video-layer.png
- icon-image-layer.png
- transparent.png
- rs_center.png
- ipadair.png
- tipsy.gif
- icon-trash.png
- icon-problem-found.png
- dash-speaker.png
- wp-arrows.png
- icon-update-refresh.png
- dash-smile.png
- icon-not-registered.png
- dash-notregistered.png
- trans_tile.png
- i_addlayer_shape.png
- install_slider.png
- dash-template-bg.jpg
- i_addlayer_button.png
- import_slider.png
- **navigationeditor**
  - tp_thumb.jpg
  - index.php
- dash-credit.png
- icon-all.png
- gridtile_3x3_white.png
- dash-buket.png
- dotted_horizontal.png
- loader.gif

css

- tipsy.css
- admin.css
- index.php
- **font**
  - egfont.svg
  - index.php
  - egfont.ttf
  - egfont.eot
  - egfont.woff
- global.css
- edit_layers.css

js

- **codemirror**
  - codemirror.js
  - **util**
    - simple-hint.js
    - formatting.js
    - runmode-standalone.js
    - xml-hint.js
    - dialog.js
    - closetag.js
    - searchcursor.js
    - search.js
    - simple-hint.css
    - loadmode.js
    - multiplex.js
    - runmode.js
    - index.php
    - dialog.css
    - javascript-hint.js
    - foldcode.js
    - pig-hint.js
    - match-highlighter.js
    - overlay.js
  - xml.js
  - codemirror.css
  - css.js
  - index.php
- admin.js
- vc.js
- jquery.tipsy.js
- css_editor.js
- oldvideo.js
- rev_admin.js
- tinymce-shortcode-script.js
- edit_layers.js
- index.php
- edit_layers_timeline.js
- newvideo.js
- settings.js

index.php

imports

- **showcasecarousel**
  - slide6.jpg
  - slide9.jpg
  - slide5.jpg
  - index.php

slide7.jpg
slide8.jpg
slide3.jpg
slide4.jpg
slide2.jpg
slider.jpg
slide1.jpg
web-product-dark
index.php
slide3.jpg
slide4.jpg
slide2.jpg
slider.jpg
slide1.jpg
news-bg-video
index.php
slider.jpg
slide1.jpg
mainfeature
slide6.jpg
slide5.jpg
index.php
slide7.jpg
slide3.jpg
slide4.jpg
slide2.jpg
slider.jpg
slide1.jpg
photography-carousel
slide10.jpg
slide6.jpg
slide9.jpg
slide5.jpg
index.php
slide7.jpg
slide8.jpg
slide3.jpg
slide4.jpg
slide2.jpg
slider.jpg
slide1.jpg
slidingoverlays
index.php
slide3.jpg
slide2.jpg

slide1.jpg

concept

index.php

slide3.jpg

slide2.jpg

slide1.jpg

sportshero

index.php

slider.jpg

slide1.jpg

web-product-dark-hero

index.php

slider.jpg

slide1.jpg

news-gallery-post-based

index.php

slider.jpg

slide1.jpg

gym

index.php

slide3.jpg

slide2.jpg

slider.jpg

slide1.jpg

youtubehero

index.php

slider.jpg

slide1.jpg

highlight-showcase

index.php

slide3.jpg

slide4.jpg

slide2.jpg

slider.jpg

slide1.jpg

vimeohero

index.php

slider.jpg

slide1.jpg

news-gallery

index.php

slide3.jpg

slide4.jpg

slide2.jpg

slider.jpg

slide1.jpg

media-gallery-two

slide6.jpg
slide5.jpg
index.php
slide3.jpg
slide4.jpg
slide2.jpg
slider.jpg
slide1.jpg

imagehero

index.php
slider.jpg
slide1.jpg

travel-static-captions

index.php
slide3.jpg
slide4.jpg
slide2.jpg
slide1.jpg

highlight-carousel

index.php
slide3.jpg
slide4.jpg
slide2.jpg
slider.jpg
slide1.jpg

classicslider

slide5.jpg
index.php
slide3.jpg
slide4.jpg
slide2.jpg
slider.jpg
slide1.jpg

notgeneric

slide5.jpg
index.php
slide3.jpg
slide4.jpg
slide2.jpg
slider.jpg
slide1.jpg

news-hero

index.php

slider.jpg
slide1.jpg
web-product-light-hero
index.php
slider.jpg
slide1.jpg
twitter-feed
index.php
slider.jpg
slide1.jpg
woobig
index.php
slide1.jpg
index.php
flickr-gallery
index.php
slider.jpg
slide1.jpg
classic-carousel
slide5.jpg
index.php
slide3.jpg
slide4.jpg
slide2.jpg
slider.jpg
slide1.jpg
creativefreedom
index.php
slide3.jpg
slide2.jpg
slide1.jpg
fashion
slide5.jpg
index.php
slide3.jpg
slide4.jpg
slide2.jpg
slider.jpg
slide1.jpg
levanorestaurantbar
slide5.jpg
index.php
slide3.jpg
slide4.jpg
slide2.jpg

slider.jpg
slide1.jpg
insta-gallery
index.php
slider.jpg
slide1.jpg
facebook-feed
index.php
slider.jpg
slide1.jpg
agency-intro
index.php
slide1.jpg
youtube-gallery
index.php
slider.jpg
slide1.jpg
finedining
index.php
slide1.jpg
contenttabs
index.php
slide3.jpg
slide2.jpg
slider.jpg
slide1.jpg
newsletter-hero
index.php
slider.jpg
slide1.jpg
media-carousel-autoplay
slide6.jpg
slide5.jpg
index.php
slide3.jpg
slide4.jpg
slide2.jpg
slider.jpg
slide1.jpg
vimeo-gallery
index.php
slider.jpg
slide1.jpg
foodcarousel
slide6.jpg

slide5.jpg
index.php
slide7.jpg
slide3.jpg
slide4.jpg
slider_cover.jpg
slide2.jpg
slide1.jpg

photography

slide10.jpg
slide6.jpg
slide9.jpg
slide5.jpg
index.php
slide7.jpg
slide8.jpg
slide3.jpg
slide4.jpg
slide2.jpg
slider.jpg
slide1.jpg

woocommercesmall

index.php
slide1.jpg

parallaxscene

index.php
slide1.jpg

news-video

index.php
slider.jpg
slide1.jpg

webproductlight

index.php
slide3.jpg
slide4.jpg
slide2.jpg
slider.jpg
slide1.jpg

search-form-hero

index.php
slider.jpg
slide1.jpg

fullscreen-button

slide5.jpg
index.php

slide3.jpg
slide4.jpg
slide2.jpg
slide1.jpg
web-product-light-hero-3d
index.php
slide1.jpg
includes
widget.class.php
template.class.php
slider.class.php
framework
woocommerce.class.php
include-framework.php
wpml.class.php
update.class.php
db.class.php
em-integration.class.php
aq-resizer.class.php
plugin-update.class.php
cssparser.class.php
functions-wordpress.class.php
functions.class.php
elements-base.class.php
base-admin.class.php
base.class.php
base-front.class.php
index.php
newsletter.class.php
output.class.php
external-sources.class.php
operations.class.php
globals.class.php
index.php
slide.class.php
googlefonts.php
extension.class.php
navigation.class.php
tinybox.class.php
rt19-extentions
css
admin.min.css
rt19-extentions.php
widgets
latest_posts.php

contact_info.php
product_categories.php
social_media.php
index.php
portfolio_categories.php
flickr.php
popular_posts.php

inc

shortcodes.php
metaboxes.php
post-types.php

editor

google_maps.php
portfolio_carousel.php
portfolio.php
column.php
content_icon_box.php
product_carousel.php
latest_news.php
quote.php
woo_product_carousel.php
blog.php
tab.php
testimonials.php
woo_products.php
accordion.php
product_categories.php
pricing_table.php
contact_form.php
divider.php
chained_contents.php
info_box.php
numbers.js
timeline.php
compare_table.php
multiple-select.js
image_gallery.php
image_carousel.php
slider.php
blog_carousel.php
testimonial_carousel.php
banner.php
counter.php
heading.php
button.php

icon_lists.php
products.php
content_box.php
row.php
staff_box.php
fallback_functions.php
metaboxes
single_product_custom_fields.php
portfolio_custom_fields.php
design_custom_fields.php
testimonial_custom_fields.php
index.php
staff_custom_fields.php
product_custom_fields.php
post_custom_fields.php
vc_functions.php
shortcodes
google_maps.php
portfolio_carousel.php
rt_social_media_share.php
rt_columns.php
portfolio_box.php
content_icon_box.php
product_carousel.php
space_box.php
rt_column_text.php
rt_chained_contents.php
woo_product_carousel.php
testimonials.php
woo_products.php
pullquote.php
rt_social_media_icons.php
rt_image_carousel.php
rt_scroll.php
rt_icon_list.php
rt_quote.php
contact_form.php
rt_row.php
divider.php
product_box.php
info_box.php
rt_column.php
rt_image_gallery.php
rt_tooltip.php
rt_product_categories.php

- pricing_tables.php
- rt_slider.php
- rt_get_commnets_template.php
- rt_tabs.php
- video_embed.php
- rt_timeline.php
- blog_box.php
- widget_caller.php
- blog_carousel.php
- testimonial_carousel.php
- rt_counter.php
- banner.php
- rt_heading.php
- button.php
- rt_highlight.php
- rt_latest_news.php
- rt_accordion.php
- content_box.php
- staff_box.php
- icon.php

shortcode_helper.php
rt_resize.php
metabox-gallery.php
visual_composer_config.php
helper-functions.php

js

jflickrfeed.min.js
app.min.js

vc_templates

vc_separator.php
vc_tab.php
vc_accordion_tab.php
vc_column_inner.php
vc_column.php
vc_accordion.php
vc_row_inner.php
vc_tabs.php
vc_row.php

js_composer

changes.txt
vc_classmap.json.php

locale

default.mo
js_composer-cs_CZ.po
js_composer-zh_TW.mo

js_composer-es_ES.mo
js_composer-zh_TW.po
js_composer-ru_RU.mo
js_composer-ja_JP.po
js_composer-de_DE.mo
js_composer-ja_JP.mo
js_composer-it_IT.mo
js_composer-cs_CZ.mo
js_composer-pl_PL.po
js_composer-it_IT.po
js_composer-nl_NL.mo
js_composer-nl_NL.po
js_composer-ru_RU.po
js_composer-pl_PL.mo
js_composer-fa_IR.po
js_composer-pt_BR.mo
js_composer-fr_FR.mo
js_composer-de_DE.po
default.po
js_composer-fa_IR.mo
readme.txt
js_composer-bg_BG.po
js_composer-bg_BG.mo
js_composer-pt_BR.po
js_composer-es_ES.po
js_composer-fr_FR.po

config

wp

shortcode-vc-wp-rss.php
shortcode-vc-wp-custommenu.php
shortcode-vc-wp-categories.php
shortcode-vc-wp-calendar.php
shortcode-vc-wp-posts.php
shortcode-vc-wp-meta.php
shortcode-vc-wp-tagcloud.php
shortcode-vc-wp-pages.php
shortcode-vc-wp-links.php
shortcode-vc-wp-text.php
shortcode-vc-wp-recentcomments.php
shortcode-vc-wp-search.php
shortcode-vc-wp-archives.php

tta

shortcode-vc-tta-pageable.php
shortcode-vc-tta-accordion.php
shortcode-vc-tta-section.php

- shortcode-vc-tta-tour.php
- shortcode-vc-tta-tabs.php

containers

- shortcode-vc-row-inner.php
- shortcode-vc-column.php
- shortcode-vc-row.php
- shortcode-vc-column-inner.php

grids

- shortcode-vc-masonry-media-grid.php
- shortcode-vc-media-grid.php
- shortcode-vc-basic-grid.php
- vc-grids-functions.php
- vc-grids-common.php
- shortcode-vc-masonry-grid.php

buttons

- shortcode-vc-btn.php
- shortcode-vc-cta.php

content

- shortcode-vc-icon.php
- shortcode-vc-images-carousel.php
- shortcode-vc-toggle.php
- shortcode-vc-gallery.php
- vc-custom-heading-element.php
- shortcode-vc-column-text.php
- shortcode-vc-line-chart.php
- shortcode-vc-flickr.php
- shortcode-vc-custom-heading.php
- shortcode-vc-round-chart.php
- shortcode-vc-single-image.php
- shortcode-vc-message.php
- vc-icon-element.php
- shortcode-vc-pie.php
- shortcode-vc-posts-slider.php
- shortcode-vc-separator.php
- shortcode-vc-progress-bar.php
- shortcode-vc-video.php
- shortcode-vc-text-separator.php
- shortcode-vc-gmaps.php
- shortcode-vc-empty-space.php

templates.php

lean-map.php

deprecated

- shortcode-vc-accordion-tab.php
- shortcode-vc-cta-button.php
- shortcode-vc-posts-grid.php

        shortcode-vc-accordion.php
        shortcode-vc-tabs.php
        shortcode-vc-button2.php
        shortcode-vc-tour.php
        shortcode-vc-tab.php
        shortcode-vc-carousel.php
        shortcode-vc-button.php
        shortcode-vc-cta-button2.php
    structure
        shortcode-vc-raw-html.php
        shortcode-vc-widget-sidebar.php
        shortcode-vc-raw-js.php
    social
        shortcode-vc-pinterest.php
        shortcode-vc-googleplus.php
        shortcode-vc-facebook.php
        shortcode-vc-tweetmeme.php
index.php
assets
    images
        toggle_close.png
        spinner.gif
        flickr.png
        toggle_open.png
        icons
            balloon.png
            fire.png
            balloon-buzz.png
            blue-document-excel.png
            plus-shield.png
            address-book.png
            application-image.png
            chart.png
            binocular.png
            bookmark.png
            battery-full.png
            camcorder.png
            clock.png
            anchor.png
            blue-document-word.png
            blue-document-image.png
            video.png
            blue-document-pdf.png
            camera.png
            blue-document-music.png

- auction-hammer.png
- chart-pie.png
- balloon-twitter.png
- blue-document-powerpoint.png
- mail.png
- alarm-clock.png
- arrow.png
- heart.png
- asterisk.png
- blue-document-office.png
- control.png
- balloon-facebook.png

css

- js_composer_settings.min.css
- vc-ie8.min.css
- js_composer_frontend_editor_iframe.min.css
- **ui-custom-theme**
  - **images**
    - ui-icons_1982d1_256x240.png
    - ui-bg_highlight-soft_100_f6f6f6_1x100.png
    - ui-bg_flat_55_ffffff_40x100.png
    - ui-icons_333333_256x240.png
    - ui-bg_highlight-soft_25_eaf2fa_1x100.png
    - ui-icons_454545_256x240.png
    - ui-bg_highlight-soft_50_dddddd_1x100.png
    - ui-bg_flat_75_ffffff_40x100.png
    - ui-bg_flat_0_eeeeee_40x100.png
    - ui-bg_glass_65_ffffff_1x400.png
    - ui-bg_flat_0_aaaaaa_40x100.png
    - ui-resize-icon.png
    - ui-icons_ff0046_256x240.png
  - jquery-ui-less.custom.min.css
- **lib**
  - **vc-open-iconic**
    - vc_openiconic.min.css
    - **fonts**
      - vc_openiconic.svg
      - vc_openiconic.eot
      - vc_openiconic.woff
      - vc_openiconic.ttf
  - **typicons**
    - **src**
      - **font**
        - typicons.min.css
        - typicons.svg

typicons.eot
LICENCE.md
typicons.woff
typicons.ttf
vc-entypo
vc_entypo.min.css
fonts
vc_entypo.svg
vc_entypo.eot
vc_entypo.ttf
vc_entypo.woff
vc-linecons
vc_linecons_icons.min.css
fonts
vc_linecons.svg
vc_linecons.woff
vc_linecons.eot
vc_linecons.ttf
isotope.min.css
js_composer.min.css
js_composer_backend_editor.min.css
vc_lte_ie9.min.css
js_composer_frontend_editor.min.css
less
wp
accordion.less
form.less
js_composer_settings.less
params
iconpicker.less
options_field.less
hidden.less
vc_grid_item
navbar.less
shortcodes
vc_gitem_zone.less
vc_gitem_zone_admin.less
vc_gitem_row.less
vc_gitem_zone_a.less
vc_gitem_zone_b.less
vc_gitem_animated_block_admin.less
vc_gitem_row_admin.less
vc_gitem_gitem_zone_height_mode.less
vc_gitem_zone_c.less
vc_gitem_col.less

        vc_gitem_animated_block.less
        vc_gitem_post_data.less
      vc_grid_item.less
      preview.less
      vc_gitem_editor.less
    param_group.less
    css_editor.less
    loop.less
    autocomplete.less
    column_offset.less
    dropdown.less
    vc_grid.less
    colorpicker.less
    vc_link.less
    sorted_list.less
    vc_grid_element.less
    vc_message_color.less
config
    variables_common.less
    variables_colors.less
    variables.less
    admin_variables.less
shortcodes
    vc_tta.less
    vc_pie.less
    vc_button3_editform.less
    vc_social_btns.less
    vc_carousel.less
    vc_widgetised_column.less
    tta
      vc_tta-colors.less
      vc_tta-core.less
      vc_tta-variables.less
      vc_tta-mixins.less
      vc_tta-frontend-editor.less
      vc_tta-options.less
      vc_tta-icon-controls.less
    vc_progress_bar.less
    vc_post_slider.less
    vc_flickr.less
    vc_zoom.less
    vc_alert.less
    vc_custom_heading.less
    vc_teaser_grid.less
    vc_separator.less

- vc_toggle.less
- vc_button.less
- vc_tabs_tour_accordion.less
- vc_icon_element.less
- vc_google_maps.less
- vc_call_to_action3.less
- vc_single_image.less
- vc_pagination.less
- vc_charts.less
- vc_button3.less
- vc_responsive.less
- vc_cta3_editform.less
- **vc_basic_grid**
  - vc_grid_loading.less
  - vc_grid_style.less
  - vc_grid_pagination_dots.less
  - vc_grid_arrows.less
  - **filter_styles**
    - filter_default_less_rounded.less
    - filter_bordered.less
    - filter_dropdown.less
    - filter_filled.less
    - filter_default.less
  - vc_grid_filter.less
  - vc_grid_pagination_numbers.less
  - vc_grid_gap.less
  - vc_grid.less
  - vc_grid_carousel.less
- vc_image_gallery.less
- **vc_message_box**
  - vc_message_box_front.less
  - vc_message_box_2.less
  - vc_message_box_mixins.less
  - vc_message_box_variables.less
- _shortcodes_container.less
- vc_video_widget.less
- vc_call_to_action.less

modules

- vc_messages.less
- vc_panels.less
- vc_buttons.less
- vc_placeholder.less
- vc_welcome.less
- vc_teaser_box.less
- vc_table.less

- vc_license_activation_notice.less
- vc_modals.less
- vc_preloader.less
- vc_helper.less
- vc_switcher.less
- vc_navbar.less
- vc_fullscreen.less
- **ui**
  - **vc_ui-panel**
    - vc_ui-panel-footer.less
    - vc_ui-panel-window.less
    - vc_ui-panel-header.less
    - vc_ui-panel.less
    - vc_ui-panel-content.less
  - vc_ui-flex-bootstrap.less
  - vc_ui-button.less
  - vc_ui-panel-row-layout.less
  - vc_ui-fonts.less
  - vc_ui-panel-edit-element.less
  - vc_ui-tabs-line.less
  - vc_ui-helper.less
  - vc_ui-panel-templates.less
  - vc_ui-prompt.less
  - vc_ui-dropdown.less
  - vc_ui-gaps.less
  - vc_ui-icon-pixel.less
  - vc_ui-loaders.less
  - vc_ui-list-bar.less

js_composer.less

js_composer_frontend_editor.less

lib

- frontend_vc_elements.less
- vc_mixins.less
- elements_icons.less
- vc_pointer.less
- frontend_draganddrop.less
- parallax.less
- vc_row.less
- responsive-utilities.less
- controls.less
- bootstrap-components.less
- css3_animations.less
- frontend_vc_row.less
- panel_preview.less
- grid.less

wpb_icon.less
utils.less
editor.less
backend_draganddrop.less
frontend_editor_controls.less
front.less
media-gallery.less
pixel_icons.less
resizable_modal.less
backend_grid_element_pointers.less
backend_controls.less
vc_font.less

vendor

rev_slider.less
jwplayer.less
woocommerce.less
gravity_form.less
contact_form_7.less
layerslider.less

backend_shortcodes

vc_gallery.less
vc_gmaps.less
vc_tta.less
vc_pie.less
vc_tab.less
vc_empty_space.less
vc_tweetmeme.less
empty_containers.less
vc_progress_bar.less
content_block.less
vc_flickr.less
vc_accordion_tab.less
vc_googleplus.less
vc_raw_html.less
vc_raw_js.less
vc_teaser_grid.less
vc_separator.less
vc_widget_sidebar.less
rows_columns.less
vc_toggle.less
vc_button.less
vc_wp_widget.less
vc_posts_slider.less
vc_call_to_action3.less
vc_single_image.less

vc_column_text.less
vc_charts.less
vc_button3.less
vc_tabs.less
vc_tour.less
vc_cta_button.less
vc_twitter.less
vc_message_box.less
vc_images_carousel.less
vc_accordion.less
vc_facebook.less
vc_pinterest.less
vc_video.less

settings_tabs

automapper.less
element_css.less
tabs_ui.less
product_license.less
general_settings.less
vc_roles.less
design_options.less
user-group.less
custom_css.less

js_composer_backend_editor.less

pages

vc_welcome.less

vc_lte_ie9.less

popups

panel_edit_layout.less
panel_edit_form.less
panel_post_settings.less
modal_add_element.less

js_composer_frontend_editor_iframe.less

fonts

vc_grid

vc_grid_v1.ttf
vc_grid_v1.svg
vc_grid_v1.eot
vc_grid_v1.woff

js

vendors

woocommerce.js
yoast.js
qtranslate_backend.js
advanced_custom_fields.js

    - woocommerce-add-to-cart.js
    - qtranslate_frontend.js
  - **frontend_editor**
    - **vendors**
      - **plugins**
        - jwplayer.js
  - **editors**
    - templates-preview.js
  - **dist**
    - backend.min.js
    - backend-actions.min.js
    - grid-builder.min.js
    - vc_grid.min.js
    - settings.min.js
    - frontend-editor.min.js
    - edit-form.min.js
    - js_composer_front.min.js
    - page_editable.min.js
- **vc**
  - pattern.gif
  - no_image.png
  - **vc-welcome**
    - 01_49.png
    - screenshot49.png
    - 03_49.png
    - 02_49.png
  - info.png
  - rowbg.png
  - search.png
  - **fe**
    - expand.gif
    - js_icon.png
    - **screen_sizes**
      - laptop.png
      - iphone_h.png
      - ipad_h.png
      - iphone_v.png
      - ipad_v.png
    - move.png
    - close_panel.png
    - controls.png
    - preloader.gif
    - eye_ico.png
    - layout_sprite.png
    - visual_composer_32.png

- **social**
  - **tw**
    - none.png
  - **pinterest**
    - horizontal.png
    - vertical.png
    - none.png
  - **fb**
    - standart.png
    - box_count.png
    - button_count.png
  - **gp**
    - tall_desc.png
    - small_desc.png
    - standard_desc.png
    - small.png
    - medium.png
    - small_bubble.png
    - tall.png
    - medium_bubble.png
    - standard.png
    - tall_bubble.png
    - medium_desc.png
    - standard_bubble.png
- modal_minimize.png
- empty_row_sprite.png

minus.png

row_layouts

- 56_16.gif
- 16_16_16_16_16_16.gif
- 14_12_14.gif
- 1.gif
- 13_23.gif
- 14_14_14_14.gif
- 14_46_16.gif
- 23_13.gif
- 16_16_16_12.gif
- 14_34.gif
- 13_13_13.gif
- 12_12.gif

navbar_controls.png

exclamation.png

elements_icons_sprite.png

tick.png

gb_additional_content.png

- **editors**
  - **ui**
    - **vc_ui-header-graphics**
      - vc_ui-header-graphic.png
      - vc_ui-icon-pixel-search.png
    - **vc_ui-icon-pixel**
      - controls.png
      - vc_ui-sprite.png
- separator.gif
- visual_composer.png
- plus.png
- **loaders**
  - spinner.svg
  - spinner-light.svg
- blank.gif
- controls.png
- **3rd-party**
  - jwplayer.png
- **logo**
  - 64x64.png
- remove.png
- blur.svg
- atm_controls.png
- empty_row.png
- drag_handler.gif
- column_offset_screen_size.png
- item_icon.png
- logo_ce.png
- visual_composer_32.png
- add.png
- vc_gitem_image.png
- alert.png

lib

- **php.default**
  - php.default.min.js
- **vc-tta-autoplay**
  - vc-tta-autoplay.min.js
- **vc_image_zoom**
  - vc_image_zoom.min.js
- **vc_line_chart**
  - vc_line_chart.min.js
- **vc_accordion**
  - vc-accordion.min.js
- **vc_carousel**
  - **css**

- vc_carousel.min.css
- **fonts**
  - glyphicons-halflings-regular.svg
  - glyphicons-halflings-regular.ttf
  - glyphicons-halflings-regular.eot
  - glyphicons-halflings-regular.woff
- **js**
  - vc_carousel.min.js
  - transition.min.js
- LICENSE

waypoints
- waypoints.min.js

vc_tabs
- vc-tabs.min.js

vc_round_chart
- vc_round_chart.min.js

owl-carousel2-dist
- **assets**
  - owl.min.css
  - owl.video.play.png
- LICENSE
- owl.carousel.min.js

bower
- **bootstrap3**
  - **less**
    - navbar.less
    - responsive-embed.less
    - scaffolding.less
    - tooltip.less
    - progress-bars.less
    - tables.less
    - navs.less
    - list-group.less
    - buttons.less
    - carousel.less
    - type.less
    - code.less
    - variables.less
    - breadcrumbs.less
    - theme.less
    - forms.less
    - responsive-utilities.less
    - popovers.less
    - wells.less
    - jumbotron.less

modals.less
close.less
pager.less
normalize.less
grid.less
mixins
- responsive-visibility.less
- nav-divider.less
- list-group.less
- buttons.less
- opacity.less
- clearfix.less
- text-emphasis.less
- image.less
- forms.less
- text-overflow.less
- reset-filter.less
- vendor-prefixes.less
- nav-vertical-align.less
- grid-framework.less
- background-variant.less
- grid.less
- tab-focus.less
- alerts.less
- border-radius.less
- size.less
- center-block.less
- resize.less
- pagination.less
- hide-text.less
- gradients.less
- progress-bar.less
- labels.less
- panels.less
- table-row.less

alerts.less
button-groups.less
badges.less
dropdowns.less
component-animations.less
input-groups.less
utilities.less
pagination.less
bootstrap.less
glyphicons.less

      labels.less
      panels.less
      media.less
      thumbnails.less
      mixins.less
      print.less
   js
      modal.min.js
      popover.min.js
      tab.min.js
      alert.min.js
      affix.min.js
      dropdown.min.js
      collapse.min.js
      tooltip.min.js
      scrollspy.min.js
      carousel.min.js
      button.min.js
      transition.min.js
   dist
      js
         bootstrap.min.js
nivoslider
   jquery.nivo.slider.pack.js
   themes
      default
         loading.gif
         bullets.png
         arrows.png
         default.min.css
   nivo-slider.min.css
   license.txt
ace-builds
   src-min-noconflict
      ace.js
      theme-chrome.js
      mode-css.js
      worker-css.js
isotope
   README.mdown
   dist
      isotope.pkgd.min.js
font-awesome
   css
      font-awesome.min.css

fonts
fontawesome-webfont.woff
fontawesome-webfont.svg
FontAwesome.otf
fontawesome-webfont.eot
fontawesome-webfont.ttf
scrollTo
jquery.scrollTo.min.js
LICENSE
imagesloaded
imagesloaded.pkgd.min.js
zoom
jquery.zoom.min.js
readme.md
chartjs
README.md
Chart.min.js
masonry
README.mdown
dist
masonry.pkgd.min.js
json-js
README
json2.min.js
vcIconPicker
themes
grey-theme
jquery.fonticonpicker.vcgrey.min.css
css
jquery.fonticonpicker.min.css
jquery.fonticonpicker.min.js
progress-circle
README.md
ProgressCircle.min.js
twbs-pagination
jquery.twbsPagination.min.js
flexslider
images
bg_play_pause.png
flexslider.min.css
jquery.flexslider-min.js
fonts
flexslider-icon.woff
flexslider-icon.eot
flexslider-icon.svg

flexslider-icon.ttf
README.mdown
lessjs
dist
less.min.js
skrollr
README.md
dist
skrollr.min.js
animate-css
animate.min.css
jquery-ui-tabs-rotate
README
jquery-ui-tabs-rotate.min.js
vc_chart
jquery.vc_chart.min.js
prettyphoto
README
images
prettyPhoto
facebook
contentPatternBottom.png
contentPatternTop.png
default_thumbnail.gif
btnNext.png
btnPrevious.png
sprite.png
contentPatternLeft.png
loader.gif
contentPatternRight.png
light_square
default_thumbnail.gif
btnNext.png
btnPrevious.png
sprite.png
loader.gif
light_rounded
default_thumbnail.gif
btnNext.png
btnPrevious.png
sprite.png
loader.gif
dark_rounded
contentPattern.png
default_thumbnail.gif

btnNext.png
btnPrevious.png
sprite.png
loader.gif
dark_square
contentPattern.png
default_thumbnail.gif
btnNext.png
btnPrevious.png
sprite.png
loader.gif
default
sprite_prev.png
sprite_y.png
sprite.png
default_thumb.png
sprite_next.png
sprite_x.png
loader.gif
css
prettyPhoto.min.css
js
jquery.prettyPhoto.min.js
include
helpers
helpers.php
helpers_factory.php
filters.php
class-vc-color-helper.php
helpers_api.php
params
iconpicker
iconpicker.php
href
href.php
el_id
el_id.php
sorted_list
sorted_list.php
custom_markup
custom_markup.php
font_container
font_container.php
options
options.php

textarea_html

   textarea_html.php

css_editor

   css_editor.php

params.php

params_preset

   params_preset.php

vc_grid_item

   shortcodes.php

   editor

      class-vc-grid-item-preview.php

      navbar

         class-vc-navbar-grid-item.php

      class-vc-grid-item-editor.php

      popups

         class-vc-add-element-box-grid-item.php

         class-vc-templates-editor-grid-item.php

   attributes.php

   templates.php

   class-wpb-map-grid-item.php

   class-vc-grid-item.php

google_fonts

   google_fonts.php

hidden

   hidden.php

autocomplete

   autocomplete.php

tab_id

   tab_id.php

loop

   loop.php

vc_grid_element

   vc_grid_id

      vc_grid_id.php

   vc_grid_element.php

param_group

   param_group.php

vc_link

   vc_link.php

load.php

colorpicker

   colorpicker.php

default_params.php

column_offset

   column_offset.php

animation_style
animation_style.php
classes
vendors
plugins
class-vc-vendor-revslider.php
woocommerce
grid-item-shortcodes.php
grid-item-filters.php
class-vc-gitem-woocommerce-shortcode.php
grid-item-attributes.php
acf
grid-item-shortcodes.php
class-vc-gitem-acf-shortcode.php
grid-item-attributes.php
class-vc-vendor-ninja-forms.php
class-vc-vendor-advanced-custom-fields.php
class-vc-vendor-qtranslate.php
class-vc-vendor-contact-form7.php
class-vc-vendor-woocommerce.php
class-vc-vendor-layerslider.php
class-vc-vendor-wpml.php
class-vc-vendor-mqtranslate.php
class-vc-vendor-yoast_seo.php
class-vc-vendor-jwplayer.php
class-vc-vendors-manager.php
editors
class-vc-frontend-editor.php
class-vc-edit-form-fields.php
navbar
class-vc-navbar.php
class-vc-navbar-frontend.php
class-vc-backend-editor.php
popups
class-vc-edit-layout.php
class-vc-post-settings.php
class-vc-templates-panel-editor.php
class-vc-shortcode-edit-form.php
class-vc-templates-editor.php
class-vc-add-element-box.php
shortcodes
vc-posts-slider.php
vc-posts-grid.php
shortcodes.php
vc-column-inner.php

wordpress-widgets.php
vc-tta-tour.php
vc-masonry-grid.php
vc-icon.php
vc-media-grid.php
vc-empty-space.php
vc-btn.php
vc-raw-html.php
vc-gitem-zone-a.php
vc-tabs.php
vc-button2.php
vc-googleplus.php
vc-tta-tabs.php
vc-row-inner.php
vc-masonry-media-grid.php
vc-gitem-post-excerpt.php
vc-row.php
vc-text-separator.php
vc-custom-field.php
vc-pie.php
vc-carousel.php
vc-gitem.php
vc-images-carousel.php
vc-button.php
vc-line-chart.php
vc-toggle.php
vc-facebook.php
vc-tta-pageable.php
vc-gitem-image.php
vc-tour.php
vc-separator.php
vc-twitter.php
vc-widget-sidebar.php
vc-column-text.php
vc-tweetmeme.php
vc-column.php
vc-cta-button.php
vc-gitem-col.php
rev-slider-vc.php
vc-basic-grid.php
vc-gitem-row.php
example.php
vc-cta.php
vc-gitem-zone-c.php
vc-raw-js.php

- vc-cta-button2.php
- vc-pinterest.php
- vc-gitem-zone-b.php
- vc-tab.php
- vc-tta-section.php
- vc-gitem-post-title.php
- vc-gitem-zone.php
- layerslider-vc.php
- vc-progress-bar.php
- vc-gallery.php
- vc-message.php
- vc-gitem-post-data.php
- vc-round-chart.php
- vc-gmaps.php
- vc-gitem-animated-block.php
- vc-gitem-post-date.php
- vc-tta-accordion.php
- vc-flickr.php
- vc-custom-heading.php
- vc-video.php
- vc-accordion-tab.php
- vc-accordion.php
- vc-single-image.php
- vc-gitem-post-meta.php
- **paginator**
  - class-vc-pageable.php

settings

- class-vc-settings.php
- class-vc-automapper.php
- class-vc-license.php
- class-vc-roles.php

updaters

- class-vc-updater.php
- class-vc-updating-manager.php

core

- class-wpb-map.php
- class-vc-mapper.php
- class-vc-sort.php
- **access**
  - abstract-class-vc-access.php
  - class-vc-role-access.php
  - class-vc-current-user-access.php
  - class-vc-current-user-access-controller.php
  - class-vc-role-access-controller.php
- class-vc-base.php

- class-vc-page.php
- class-vc-pages-group.php
- class-vc-shared-library.php
- interfaces.php
- class-vc-post-admin.php

templates
- teaser.html.php
- **params**
  - **options**
    - templates.html
  - **vc_grid_item**
    - **editor**
      - **partials**
        - vc_grid_item_editor_footer.tpl.php
      - vc_grid_item_editor.tpl.php
      - vc_ui-template-preview.tpl.php
    - **shortcodes**
      - vc_btn.php
      - vc_single_image.php
      - vc_icon.php
      - vc_custom_heading.php
      - vc_button2.php
    - preview.tpl.php
    - **attributes**
      - vc_btn.php
      - featured_image.php
  - **google_fonts**
    - template.php
  - **loop**
    - templates.html
  - **param_group**
    - inner_content.tpl.php
    - content.tpl.php
    - add.tpl.php
  - **column_offset**
    - template.tpl.php
- **editors**
  - frontend_editor.tpl.php
  - frontend_template.tpl.php
  - **partials**
    - vc_ui-presets-dropdown.tpl.php
    - vc_ui-templates-tabs.tpl.php
    - vc_settings-image-block.tpl.php
    - frontend_controls.tpl.php
    - access-manager-js.tpl.php

templates_search.tpl.php
backend_editor_footer.tpl.php
backend_controls.tpl.php
prompt.tpl.php
backend-shortcodes-templates.tpl.php
backend_controls_tab.tpl.php
vc_welcome_block.tpl.php
settings_presets_popup.tpl.php
post_shortcodes.tpl.php
add_element_tabs.tpl.php
add_element_search.tpl.php
navbar
navbar.tpl.php
backend_editor.tpl.php
vc_ui-template-preview.tpl.php
popups
panel_templates_editor.tpl.php
vc_ui-panel-post-settings.tpl.php
modal_edit_element.tpl.php
panel_shortcode_edit_form.tpl.php
vc_ui-panel-add-element.tpl.php
vc_ui-panel-row-layout.tpl.php
vc_ui-header.tpl.php
panel_templates.tpl.php
panel_edit_layout.tpl.php
vc_ui-panel-edit-element.tpl.php
panel_post_settings.tpl.php
vc_ui-footer.tpl.php
vc_ui-panel-templates.tpl.php
panel_multi_shortcode_edit_form.tpl.php
shortcodes
vc_btn.php
vc_pie.php
vc_line_chart.php
vc_wp_links.php
vc_cta_button2.php
vc_gitem_post_meta.php
vc_wp_posts.php
vc_wp_meta.php
vc_gallery.php
vc_separator.php
vc_gitem_post_data.php
vc_flickr.php
vc_carousel.php
vc_video.php

vc_tab.php
vc_wp_categories.php
vc_tta_section.php
vc_accordion_tab.php
vc_text_separator.php
vc_wp_calendar.php
vc_custom_field.php
vc_gitem_col.php
vc_message.php
vc_gitem_animated_block.php
vc_wp_custommenu.php
vc_column_inner.php
vc_button.php
vc_single_image.php
vc_icon.php
layerslider_vc.php
vc_cta_button.php
vc_widget_sidebar.php
vc_gitem.php
vc_column.php
vc_toggle.php
vc_accordion.php
vc_posts_slider.php
vc_tweetmeme.php
vc_items.php
vc_round_chart.php
vc_wp_recentcomments.php
vc_wp_archives.php
vc_gitem_zone.php
vc_basic_grid.php
rev_slider_vc.php
vc_gitem_image.php
vc_googleplus.php
vc_wp_text.php
vc_gitem_zone_c.php
vc_custom_heading.php
vc_progress_bar.php
vc_gitem_row.php
vc_wp_search.php
vc_raw_html.php
vc_empty_space.php
vc_images_carousel.php
vc_row_inner.php
vc_wp_tagcloud.php
vc_wp_pages.php

    vc_item.php
    vc_basic_grid_filter.php
    vc_tta_global.php
    vc_tabs.php
    vc_tta_pageable_section.php
    vc_gitem_block.php
    vc_posts_grid.php
    vc_wp_rss.php
    vc_column_text.php
    vc_row.php
    vc_facebook.php
    vc_button2.php
    vc_pinterest.php
    post_block
      _item.php
    vc_cta.php
    vc_gmaps.php
  pages
    vc-welcome
      vc-welcome.php
      index.php
      vc-faq.php
      vc-resources.php
    vc-settings
      tab.php
      tab-vc-roles.php
      index.php
      vc-automapper.php
    partials
      _settings_tabs.php
      vc-roles-parts
        _grid_builder.tpl.php
        _presets.tpl.php
        _frontend_editor.tpl.php
        _post_types.tpl.php
        _part.tpl.php
        _post_settings.tpl.php
        _settings.tpl.php
        _templates.tpl.php
        _backend_editor.tpl.php
        _shortcodes.tpl.php
      _tabs.php
autoload
  class-vc-settings-presets.php
  params-to-init.php

hook-vc-wp-text.php
hook-vc-iconpicker-param.php
vc-grid-item-editor.php
hook-vc-message.php
vendors
qtranslate.php
acf.php
cf7.php
wp_customize.php
wpml.php
revslider.php
gravity_forms.php
mqtranslate.php
yoast_seo.php
woocommerce.php
layerslider.php
ninja_forms.php
jwplayer.php
components.json
params
vc_grid_item.php
hidden.php
ui-vc-pointers.php
hook-vc-progress-bar.php
frontend-editor-ie.php
bc-access-rules-4.8.php
vc-shortcode-autoloader.php
vc-image-filters.php
hook-vc-pie.php
class-vc-vendor-presets.php
vc-pointers-backend-editor.php
vc-pointers-frontend-editor.php
backend-editor-ie.php
vc-settings-presets.php
vc-pages
pages.php
settings-tabs.php
welcome-screen.php
page-role-manager.php
page-design-options.php
automapper.php
page-custom-css.php
hook-vc-grid.php
vc-single-image.php
vendor

mmihey
PHP-Instagram-effects
README.md
LICENCE
src
Image
Filter.php
js_composer.php
credits.txt
files
ruler
images
cursor.png
bt_hover.png
bt_normal.png
ruler.png
js
pixi.min.js
main.js
index.php
questions
5a7c6b56d2fbf.docx
app.html
users
57.jpg
55.jpg
61.jpg
36.jpg
99.jpg
30.jpg
1.jpg
62.jpg
14.jpg
9.jpg
33.jpg
38.jpg
47.jpg
39.jpg
41.jpg
40.jpg
59.jpg
86.jpg
87.jpg
public
39.MOV

    - ruler.psd
    - convertido.zip
    - 59.MOV
  - soped_videos.zip
  - soped_full.zip
  - pezinho.html
  - **3d_occorency**
    - dedo_martelo.png
    - pus.png
    - bolha.png
    - liquido.png
    - branco.png
    - rachadura.png
    - dor.png
    - preto.png
    - calo.png
    - garras.png
    - superior.png
    - posterior.png
    - ferida.png
    - halux.png
    - olho_peixe.png
    - inferior.png
    - unha.png
    - sangramento.png
    - micose.png
    - vermelho.png
  - **pages**
    - como_Chegar_03.png
  - **noticias**
    - noticias.jpg
  - **convertido**
  - termo.pdf
  - **equipe**
    - ricardo.jpg
    - cristina.jpg
    - isabel.jpg
    - viviane.jpg
    - no-avatar2.jpg
    - lucas.jpg
    - jane.jpg
    - elena.jpg
    - pedro.jpg
    - no-avatar.jpg
- **php**

- top_content.php
- us.utils.php
- top_content-en.php
- footer-en.php
- **PHPMailer**
  - **extras**
    - htmlfilter.php
    - class.html2text.php
    - ntlm_sasl_client.php
    - EasyPeasyICS.php
  - README.md
  - **test**
    - runfakepopserver.sh
    - fakepopserver.sh
    - test_callback.php
    - fakesendmail.sh
    - phpmailerTest.php
    - bootstrap.php
    - testbootstrap-dist.php
    - phpmailerLangTest.php
  - travis.phpunit.xml.dist
  - **docs**
    - Callback_function_notes.txt
    - pop3_article.txt
    - Note_for_SMTP_debugging.txt
    - DomainKeys_notes.txt
    - generatedocs.sh
    - faq.html
    - extending.html
  - PHPMailerAutoload.php
  - **examples**
    - mailing_list.phps
    - **images**
      - phpmailer.png
      - phpmailer_mini.png
    - code_generator.phps
    - **scripts**
      - shCore.js
      - shBrushPhp.js
      - shAutoloader.js
      - XRegExp.js
      - shLegacy.js
    - pop_before_smtp.phps
    - contents.html
    - smtp_no_auth.phps

styles

- shThemeEclipse.css
- shThemeMDUltra.css
- shThemeVisualStudio.css
- shCoreDjango.css
- shCoreEmacs.css
- shCoreDefault.css
- shThemeDjango.css
- shCoreRDark.css
- shCoreMDUltra.css
- shThemeDefault.css
- shThemeMidnight.css
- shCore.css
- shThemeAppleScript.css
- shThemeEmacs.css
- shThemeFadeToGrey.css
- shCoreMidnight.css
- shCoreEclipse.css
- shCoreFadeToGrey.css
- shThemeRDark.css
- wrapping.png

index.html
mail.phps
smtp.phps
sendmail.phps
LGPLv3.txt
exceptions.phps
gmail.phps

language

phpmailer.lang-br.php
phpmailer.lang-vi.php
phpmailer.lang-ch.php
phpmailer.lang-be.php
phpmailer.lang-uk.php
phpmailer.lang-zh.php
phpmailer.lang-nl.php
phpmailer.lang-no.php
phpmailer.lang-lt.php
phpmailer.lang-hu.php
phpmailer.lang-ja.php
phpmailer.lang-tr.php
phpmailer.lang-zh_cn.php
phpmailer.lang-fo.php
phpmailer.lang-pt.php
phpmailer.lang-lv.php

        phpmailer.lang-de.php
        phpmailer.lang-et.php
        phpmailer.lang-eo.php
        phpmailer.lang-fa.php
        phpmailer.lang-hr.php
        phpmailer.lang-he.php
        phpmailer.lang-ar.php
        phpmailer.lang-ka.php
        phpmailer.lang-fi.php
        phpmailer.lang-fr.php
        phpmailer.lang-it.php
        phpmailer.lang-el.php
        phpmailer.lang-dk.php
        phpmailer.lang-sk.php
        phpmailer.lang-ca.php
        phpmailer.lang-se.php
        phpmailer.lang-gl.php
        phpmailer.lang-pl.php
        phpmailer.lang-cz.php
        phpmailer.lang-ro.php
        phpmailer.lang-es.php
        phpmailer.lang-ru.php
        phpmailer.lang-sr.php
    class.smtp.php
    class.phpmailer.php
    composer.json
    LICENSE
    changelog.md
    class.pop3.php
responsive-menu.php
menu-en.php
menu.php
languages
    en_us.php
    pt_br.php
head.php
us.class.messages.php
us.class.database.php
admin
    cidades.php
slide.php
footer.php
pages
    contato.php
    register.php

polineuropatia.php
privacy.php
compartilhar.php
cuidados.php
start.php
diabetes.php
protocolo.php
video.php
comunicacao.php
compartilhar2.php
colaboradores.php
internal.php
video_avaliacao.php
adm.php
projeto.php
reasons.php
checklist.php
tutorial.php
protocolo_final.php
lojinha.php
intro2.php
admin_complicacoes.php
program.php
protocolo_final2.php
teste.php
introducao.php
old_user_start.php
secret.php
occurrence.php
register_new.php
pontuacao.php
terms.php
nouse
- user_auto03.php
- user_auto04.php
- user_auto01_register.php
- user_auto02.php

exercicios.php
solicitar.php
questions.php
administrador.php
user_start.php
questionarios.php
noticias.php
program2.php

avatar.php
intro_soped.php
eventos.php
social.php
equipe.php
register2.php
publicacoes.php
recompensas.php
back.php
questionario.php
apresentacao.php
perguntar.php
registration.php
user_auto.php
email.php
MysqlDB.php
us.control.php
